# Supplementary material for: Abiotic and past climatic conditions drive protein abundance variation among natural populations of the caddisfly Crunoecia irrorata
Source: Sci Rep. 2020 Sep 23;10:15538. doi: 10.1038/s41598-020-72569-4 (PMC7512004; doi:10.1038/s41598-020-72569-4)
Supplement: Supplementary file 2 — Supplementary Information 2. [file 41598_2020_72569_MOESM2_ESM.zip › SI3_artMS_QC/QC-ID-Overlap.pdf]

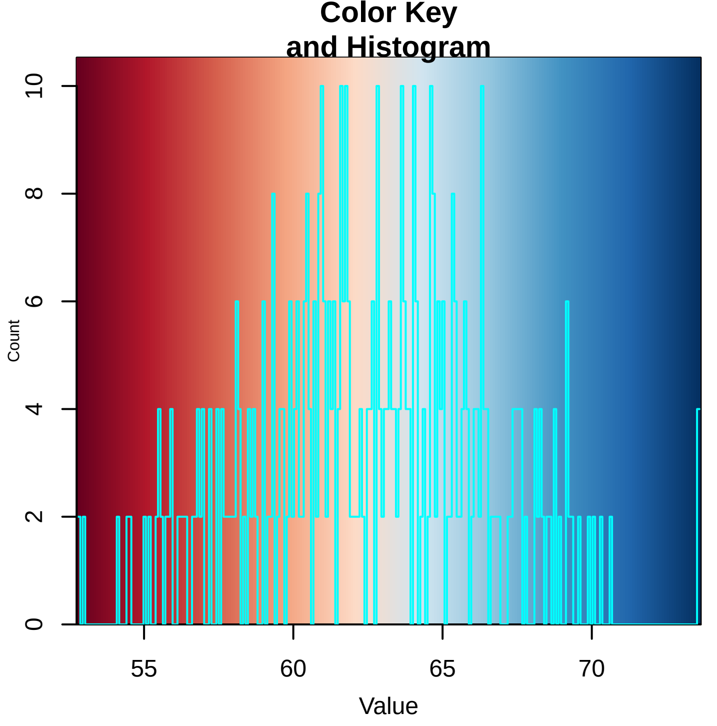

Pairwise peptide identification overlap (only peptides with at least 1 peptide detected and identified)

Harz−1		Harz−3		Harz−5		Harz−7		Rhoen−1		Rhoen−3		Rhoen−5		Rhoen−7		SW−1		SW−3		SW−5		SW−7		
	59.5	65.7	57	63.2	66.4	61.7	66.6	66.9	61.7	67.4	61.2	62.9	67.8	65.3	63.7	65.3	66.1	69.1	61.6	68.1	64.9	65.6	58	
59.5		55.2	63.9	65.4	64.1	67.4	60.6	57	55	59.3	55.9	63.6	58.1	62.6	60.4	56.6	61.6	59	55.8	60.2	58	55.6	59.2	Harz−2
65.7	55.2		59.3	63.7	68.2	60.5	66.2	64	68.3	63.2	64.5	64.9	64.4	58.5	61	68.1	64.1	64.1	63.8	59.9	58.6	60	55.9	
57	63.9	59.3		65.5	65.1	67.2	61.6	55.5	56.3	58.2	57.2	64.6	58.1	57.7	63.1	59.8	61.7	58.4	60.8	57.2	56.4	52.8	59.7	Harz−4
63.2	65.4	63.7	65.5		70.6	67.5	65.6	61.3	60.7	62.7	61.9	64.6	62.3	62.7	62.8	63.7	66.1	63.3	60.9	62.4	61.2	57.8	61.7	
66.4	64.1	68.2	65.1	70.6		67.6	66.3	64.7	63.4	65.8	62.9	66.2	64.6	64.9	65.8	66.7	68.5	67.5	62.7	63.8	62.5	60.9	58.8	Harz−6
61.7	67.4	60.5	67.2	67.5	67.6		69.2	58.2	57.4	59.6	56.8	64.4	60.3	62.3	63.6	63.8	66.5	64.9	62.9	63.9	63	60.9	63.4	
66.6	60.6	66.2	61.6	65.6	66.3	69.2		61.6	60.9	60.7	60.1	65.1	61.8	60.4	62.1	70.4	69.3	69.6	67.3	66.3	65.3	64.7	61.3	Harz−8
66.9	57	64	55.5	61.3	64.7	58.2	61.6		68.6	73.6	65.8	66	68.8	68.8	60.9	63.6	62.9	64.7	54.5	61.7	64.6	61	55.4	
61.7	55	68.3	56.3	60.7	63.4	57.4	60.9	68.6		66.4	69.4	64.7	65.8	59.6	61.3	63.2	62.2	59.7	58.1	57.4	56	58.6	54.1	Rhoen−2
67.4	59.3	63.2	58.2	62.7	65.8	59.6	60.7	73.6	66.4		66.9	66.5	73.6	69	65	61	62.5	63.3	55.5	62	63.6	60.6	57.6	
61.2	55.9	64.5	57.2	61.9	62.9	56.8	60.1	65.8	69.4	66.9		64.8	66.3	60.2	61.6	61.3	59.4	59.3	56.7	56.8	56.9	56.3	54.4	Rhoen−4
62.9	63.6	64.9	64.6	64.6	66.2	64.4	65.1	66	64.7	66.5	64.8		65.4	63.8	66.3	64	63.7	61.6	61	61.8	61.2	59.2	60.1	
67.8	58.1	64.4	58.1	62.3	64.6	60.3	61.8	68.8	65.8	73.6	66.3	65.4		67.7	64.3	61.6	63.2	64.8	56.2	62.8	62.6	61.8	57.9	Rhoen−6
65.3	62.6	58.5	57.7	62.7	64.9	62.3	60.4	68.8	59.6	69	60.2	63.8	67.7		59.9	59	60.4	62.1	53	63.2	65.4	59.9	57.6	
63.7	60.4	61	63.1	62.8	65.8	63.6	62.1	60.9	61.3	65	61.6	66.3	64.3	59.9		60.9	62.9	61.8	61.3	60.8	58.7	58.7	60	Rhoen−8
65.3	56.6	68.1	59.8	63.7	66.7	63.8	70.4	63.6	63.2	61	61.3	64	61.6	59	60.9		69.2	68.3	66.3	65.8	64.2	64.7	59	
66.1	61.6	64.1	61.7	66.1	68.5	66.5	69.3	62.9	62.2	62.5	59.4	63.7	63.2	60.4	62.9	69.2		70.1	64	67.5	63.4	64	60.5	SW−2
69.1	59	64.1	58.4	63.3	67.5	64.9	69.6	64.7	59.7	63.3	59.3	61.6	64.8	62.1	61.8	68.3	70.1		65.2	69.9	67.5	66	60.5	
61.6	55.8	63.8	60.8	60.9	62.7	62.9	67.3	54.5	58.1	55.5	56.7	61	56.2	53	61.3	66.3	64	65.2		63.1	60.9	60.5	61.1	SW−4
68.1	60.2	59.9	57.2	62.4	63.8	63.9	66.3	61.7	57.4	62	56.8	61.8	62.8	63.2	60.8	65.8	67.5	69.9	63.1		68.2	65.5	61.2	
64.9	58	58.6	56.4	61.2	62.5	63	65.3	64.6	56	63.6	56.9	61.2	62.6	65.4	58.7	64.2	63.4	67.5	60.9	68.2		65.5	61.8	SW−6
65.6	55.6	60	52.8	57.8	60.9	60.9	64.7	61	58.6	60.6	56.3	59.2	61.8	59.9	58.7	64.7	64	66	60.5	65.5	65.5		60	
58	59.2	55.9	59.7	61.7	58.8	63.4	61.3	55.4	54.1	57.6	54.4	60.1	57.9	57.6	60	59	60.5	60.5	61.1	61.2	61.8	60		SW−8

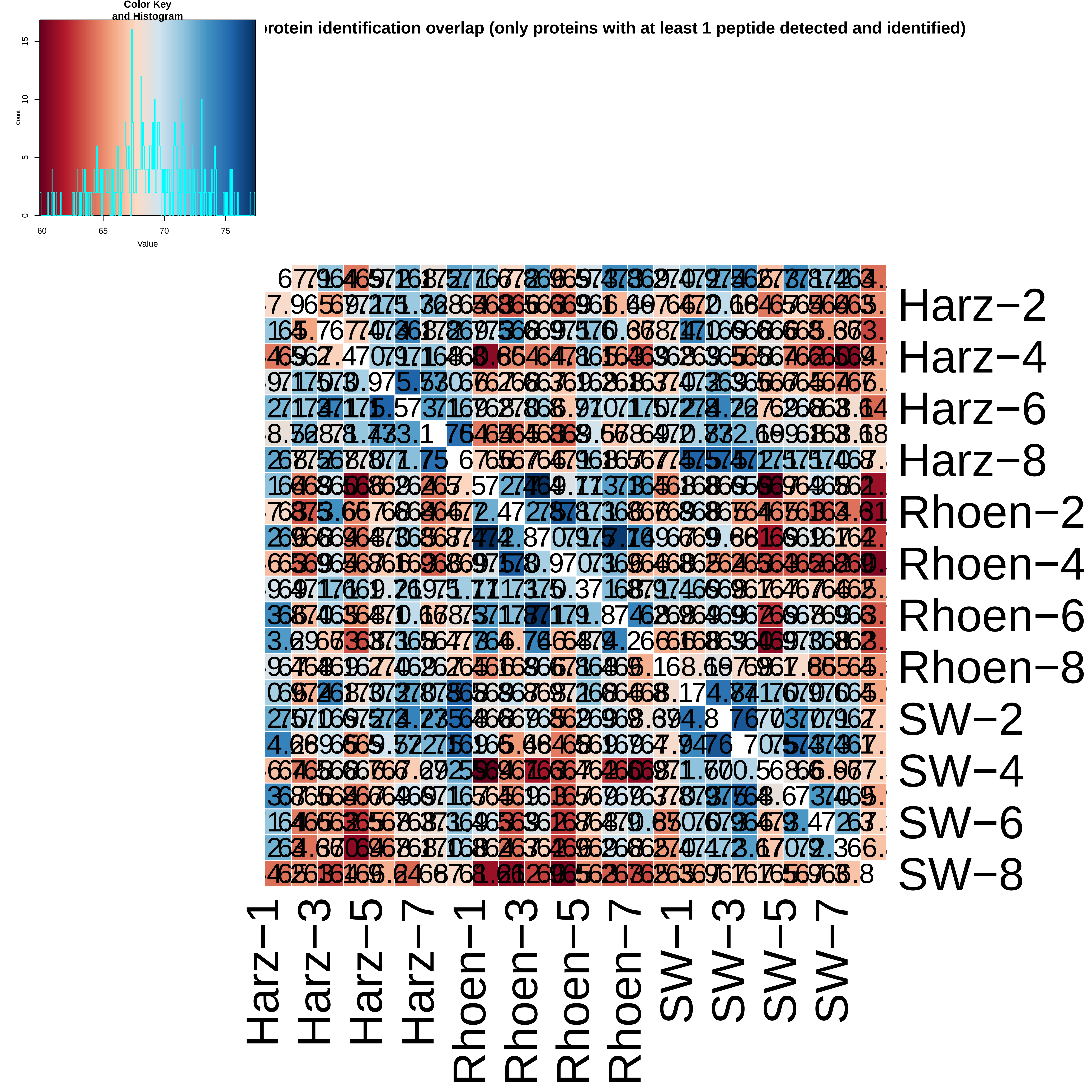

Color Key
and Histogram
Count
Value
60
65
70
75
0
5
10
15
rotein identification overlap (only proteins with at least 1 peptide detected and identified)
Harz−2
Harz−4
Harz−6
Harz−8
Rhoen−2
Rhoen−4
Rhoen−6
Rhoen−8
SW−2
SW−4
SW−6
SW−8
Harz−1
Harz−3
Harz−5
Harz−7
Rhoen−1
Rhoen−3
Rhoen−5
Rhoen−7
SW−1
SW−3
SW−5
SW−7
