# Supplementary material for: Abiotic and past climatic conditions drive protein abundance variation among natural populations of the caddisfly Crunoecia irrorata
Source: Sci Rep. 2020 Sep 23;10:15538. doi: 10.1038/s41598-020-72569-4 (PMC7512004; doi:10.1038/s41598-020-72569-4)

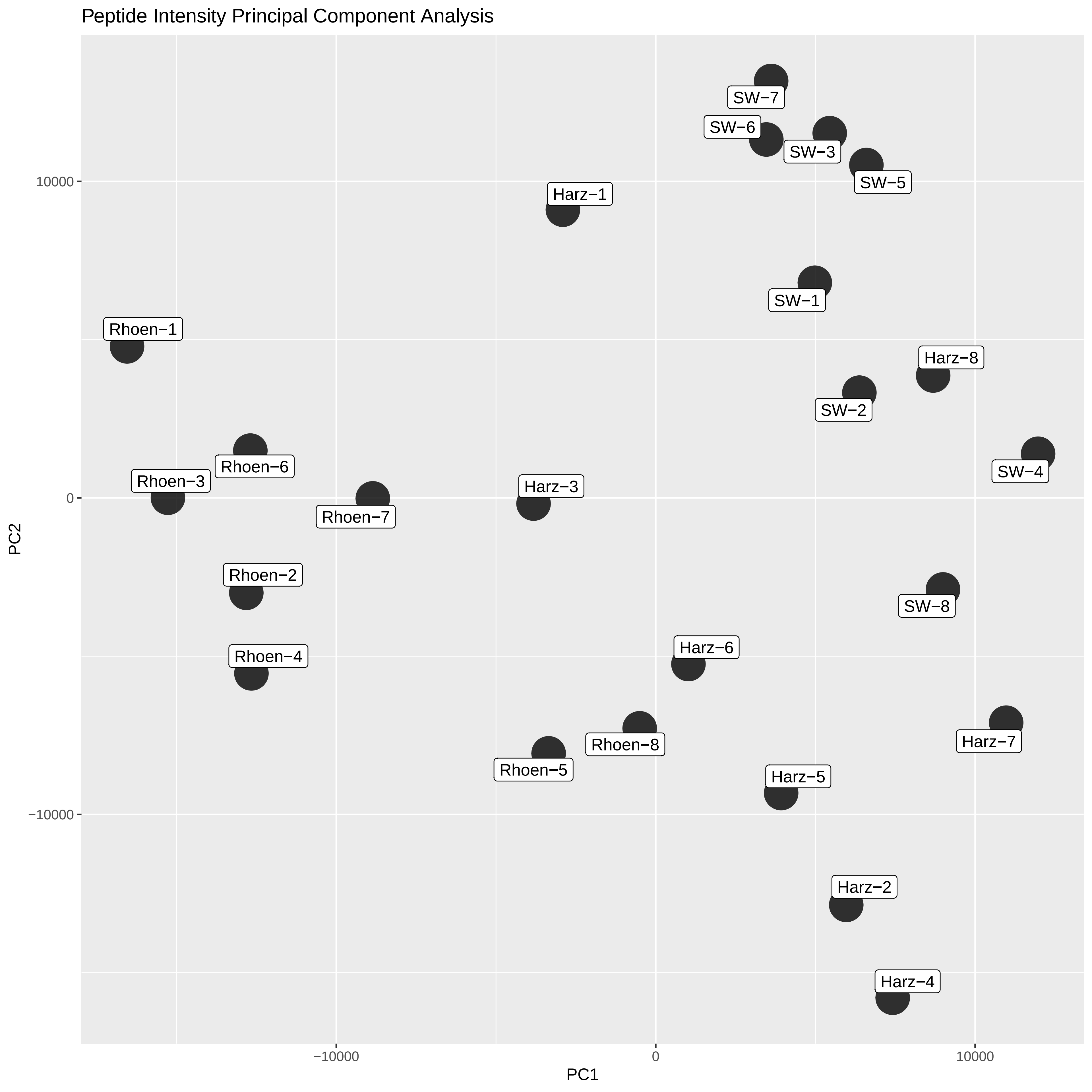

Peptide Intensity Principal Component Analysis
PC1
PC2
10000
0
−10000
−10000
0
10000
SW−7
SW−6
SW−3
SW−5
Harz−1
SW−1
Rhoen−1
Harz−8
SW−2
SW−4
Rhoen−6
Rhoen−3
Rhoen−7
Harz−3
Rhoen−2
SW−8
Harz−6
Rhoen−4
Harz−7
Rhoen−8
Rhoen−5
Harz−5
Harz−2
Harz−4

Protein Intensity Principal Component Analysis

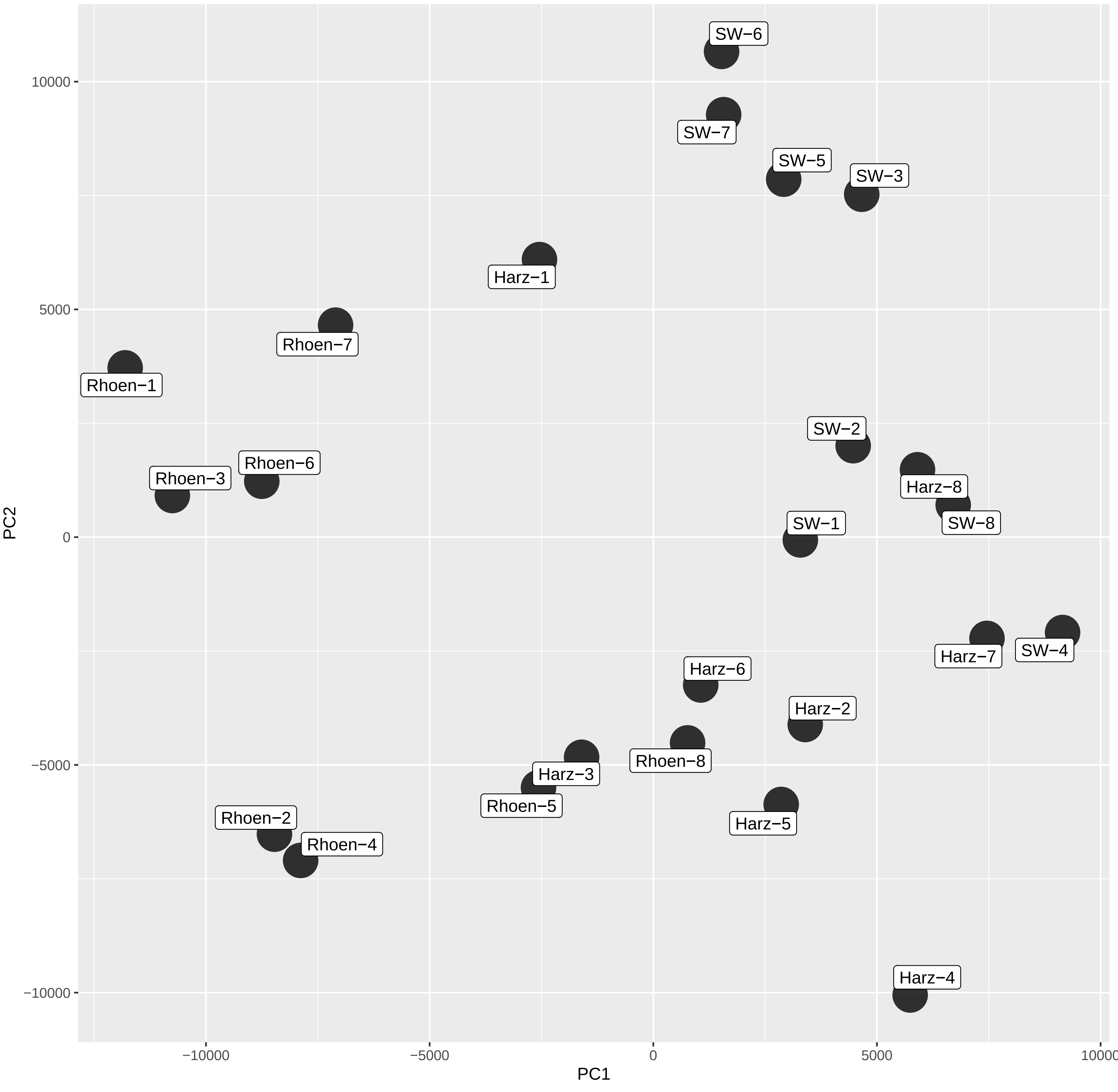

Supplement: Supplementary file 2 — Supplementary Information 2. [file 41598_2020_72569_MOESM2_ESM.zip › SI3_artMS_QC/QC-IntCorrelation.pdf]
