# Supplementary material for: Abiotic and past climatic conditions drive protein abundance variation among natural populations of the caddisfly Crunoecia irrorata
Source: Sci Rep. 2020 Sep 23;10:15538. doi: 10.1038/s41598-020-72569-4 (PMC7512004; doi:10.1038/s41598-020-72569-4)
Supplement: Supplementary file 2 — Supplementary Information 2. [file 41598_2020_72569_MOESM2_ESM.zip › SI3_artMS_QC/QC_Plots_IONS.pdf]

Number of unique Peptide Ions:

bottom = Potential contaminants; top = non−contaminants

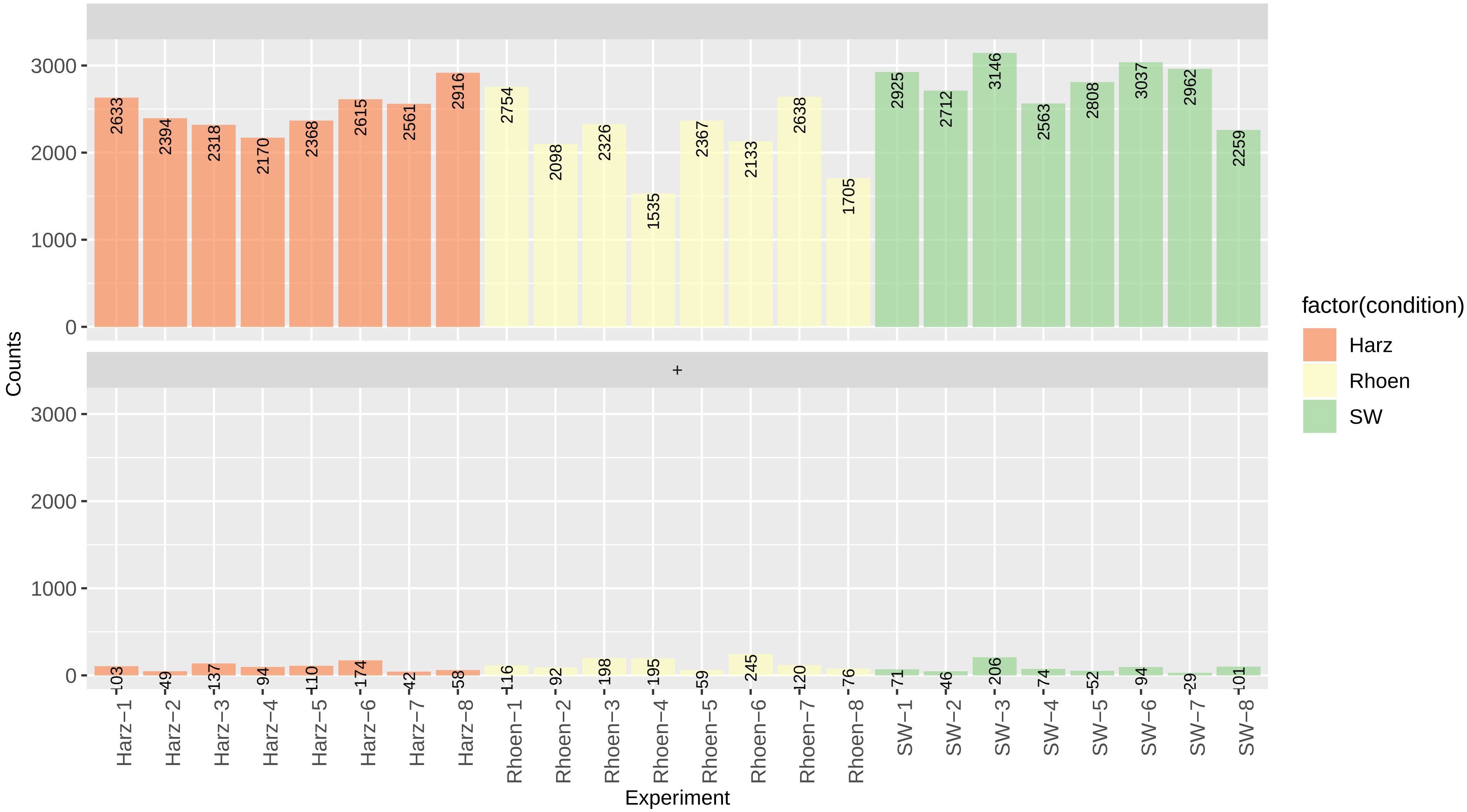

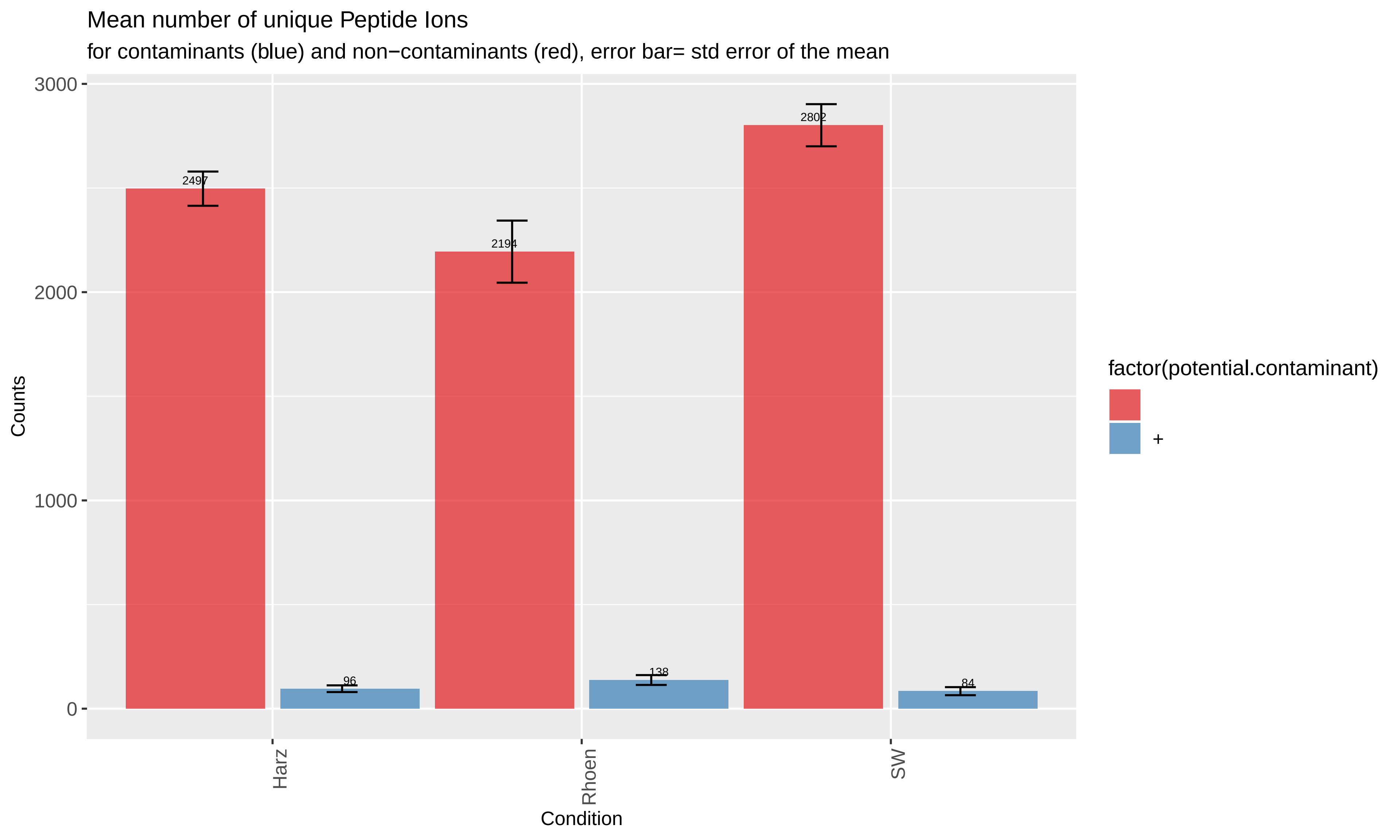

Mean number of unique Peptide Ions
for contaminants (blue) and non−contaminants (red), error bar= std error of the mean
3000
2000
1000
0
Counts
2497
96
2194
138
2802
84
Harz
Rhoen
SW
Condition
factor(potential.contaminant)
+
