# Supplementary material for: Abiotic and past climatic conditions drive protein abundance variation among natural populations of the caddisfly Crunoecia irrorata
Source: Sci Rep. 2020 Sep 23;10:15538. doi: 10.1038/s41598-020-72569-4 (PMC7512004; doi:10.1038/s41598-020-72569-4)
Supplement: Supplementary file 2 — Supplementary Information 2. [file 41598_2020_72569_MOESM2_ESM.zip › SI3_artMS_QC/QC_Plots_PEPINT.pdf]

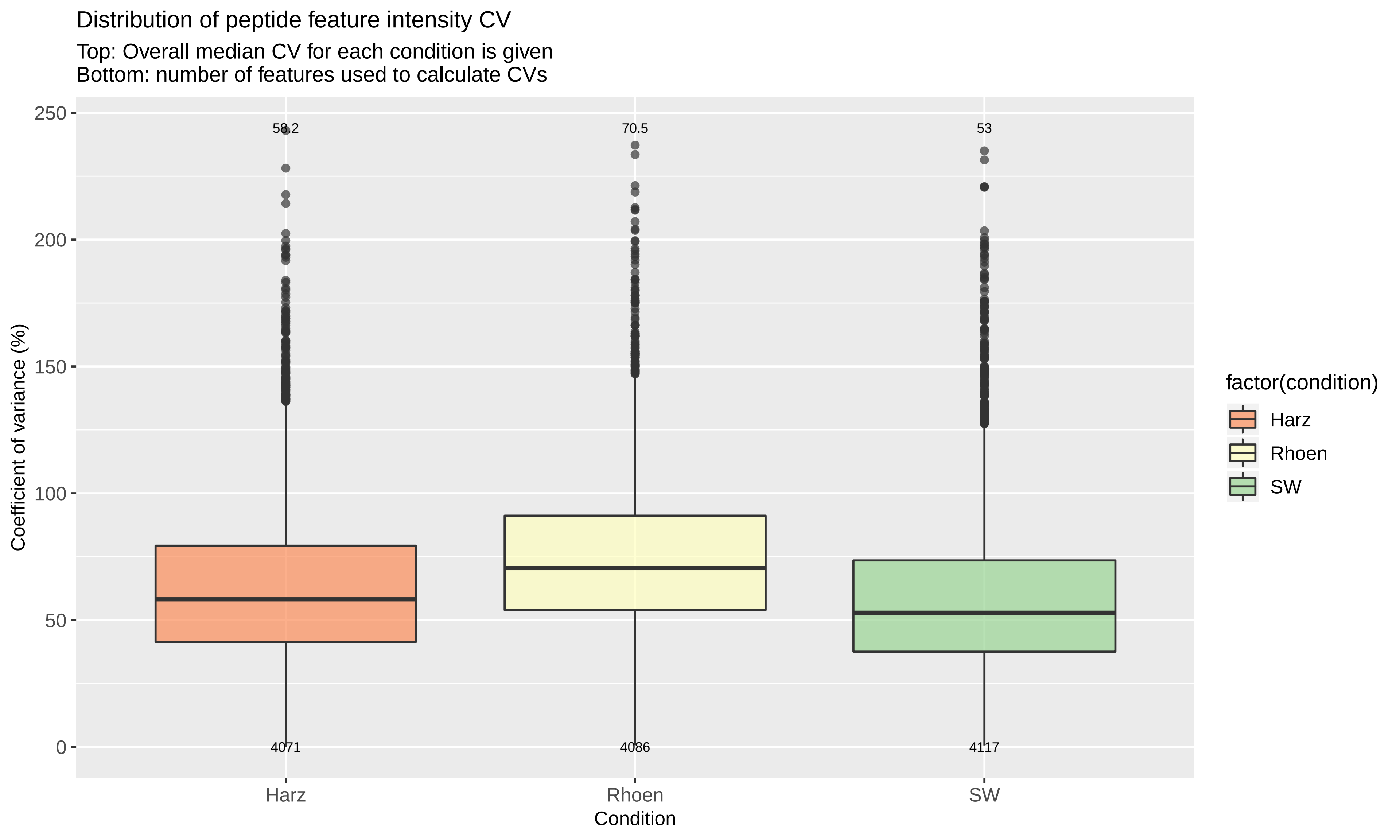
Distribution of peptide feature intensity CV
Top: Overall median CV for each condition is given
Bottom: number of features used to calculate CVs
Coefficient of variance (%)
250
200
150
100
50
0
58.2
70.5
53
4071
4086
4117
Harz
Rhoen
SW
Condition
factor(condition)
Harz
Rhoen
SW

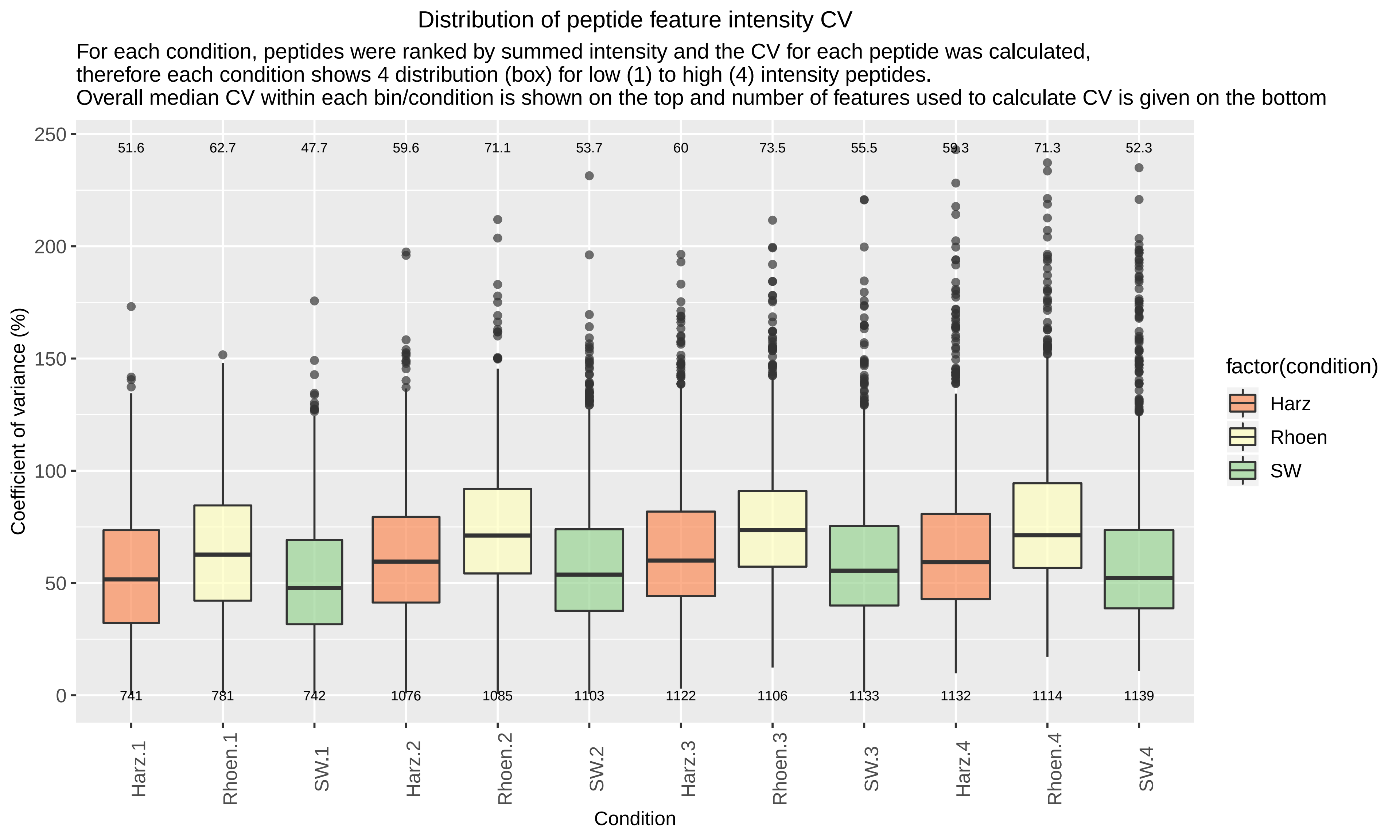
Distribution of peptide feature intensity CV
For each condition, peptides were ranked by summed intensity and the CV for each peptide was calculated,
therefore each condition shows 4 distribution (box) for low (1) to high (4) intensity peptides.
Overall median CV within each bin/condition is shown on the top and number of features used to calculate CV is given on the bottom
Coefficient of variance (%)
0
50
100
150
200
250
51.6
62.7
47.7
59.6
71.1
53.7
60
73.5
55.5
59.3
71.3
52.3
741
781
742
1076
1085
1103
1122
1106
1133
1132
1114
1139
Harz.1
Rhoen.1
SW.1
Harz.2
Rhoen.2
SW.2
Harz.3
Rhoen.3
SW.3
Harz.4
Rhoen.4
SW.4
Condition
factor(condition)
Harz
Rhoen
SW
