# Supplementary material for: Abiotic and past climatic conditions drive protein abundance variation among natural populations of the caddisfly Crunoecia irrorata
Source: Sci Rep. 2020 Sep 23;10:15538. doi: 10.1038/s41598-020-72569-4 (PMC7512004; doi:10.1038/s41598-020-72569-4)
Supplement: Supplementary file 2 — Supplementary Information 2. [file 41598_2020_72569_MOESM2_ESM.zip › SI3_artMS_QC/QC_Plots_PepIonOversampling.pdf]

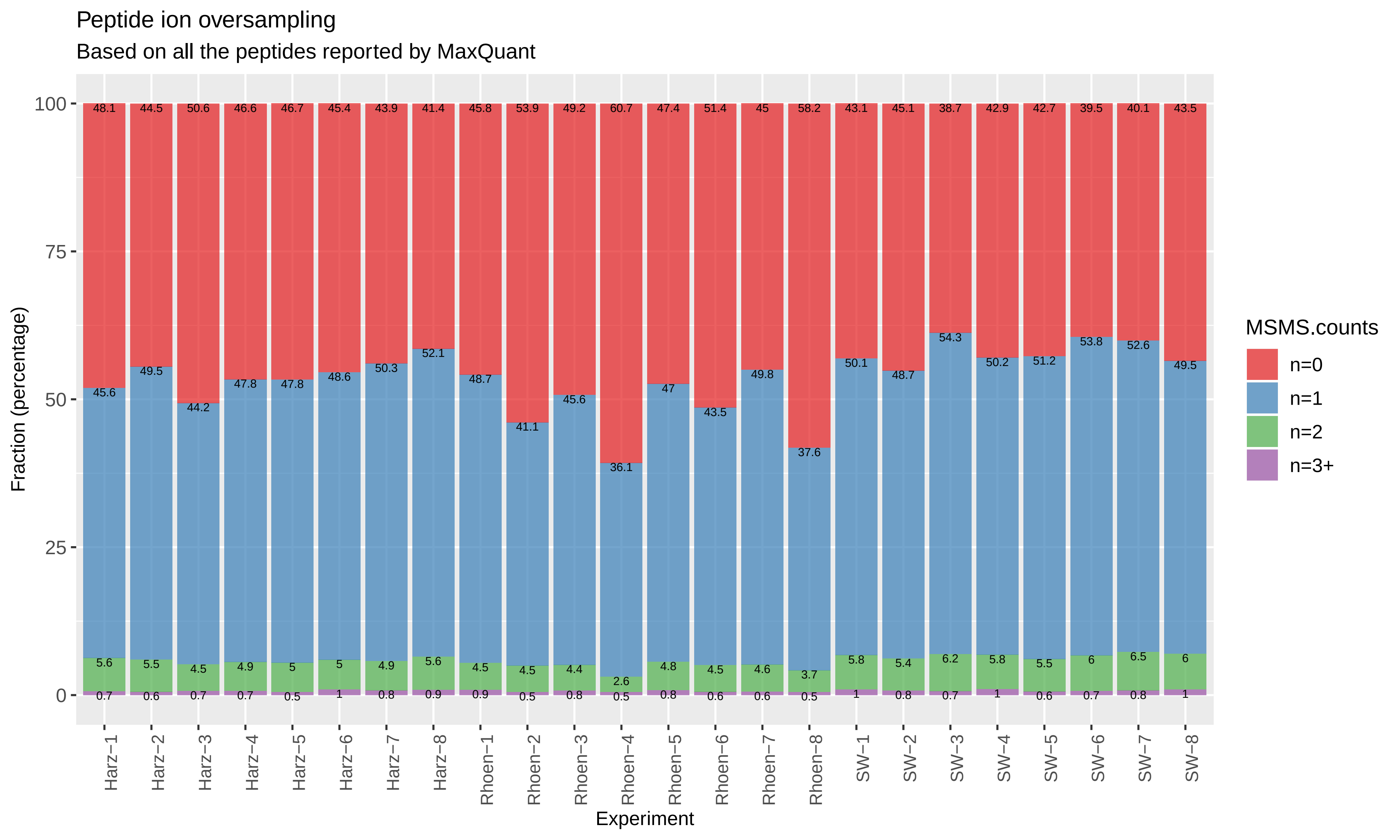
Peptide ion oversampling
Based on all the peptides reported by MaxQuant
Fraction (percentage)
Experiment
MSMS.counts
n=0
n=1
n=2
n=3+
100
75
50
25
0
Harz−1
Harz−2
Harz−3
Harz−4
Harz−5
Harz−6
Harz−7
Harz−8
Rhoen−1
Rhoen−2
Rhoen−3
Rhoen−4
Rhoen−5
Rhoen−6
Rhoen−7
Rhoen−8
SW−1
SW−2
SW−3
SW−4
SW−5
SW−6
SW−7
SW−8
48.1
44.5
50.6
46.6
46.7
45.4
43.9
41.4
45.8
53.9
49.2
60.7
47.4
51.4
45
58.2
43.1
45.1
38.7
42.9
42.7
39.5
40.1
43.5
45.6
49.5
44.2
47.8
47.8
48.6
50.3
52.1
48.7
41.1
45.6
36.1
47
43.5
49.8
37.6
50.1
48.7
54.3
50.2
51.2
53.8
52.6
49.5
5.6
5.5
4.5
4.9
5
5
4.9
5.6
4.5
4.5
4.4
2.6
4.8
4.5
4.6
3.7
5.8
5.4
6.2
5.8
5.5
6
6.5
6
0.7
0.6
0.7
0.7
0.5
1
0.8
0.9
0.9
0.5
0.8
0.5
0.8
0.6
0.6
0.5
1
0.8
0.7
1
0.6
0.7
0.8
1

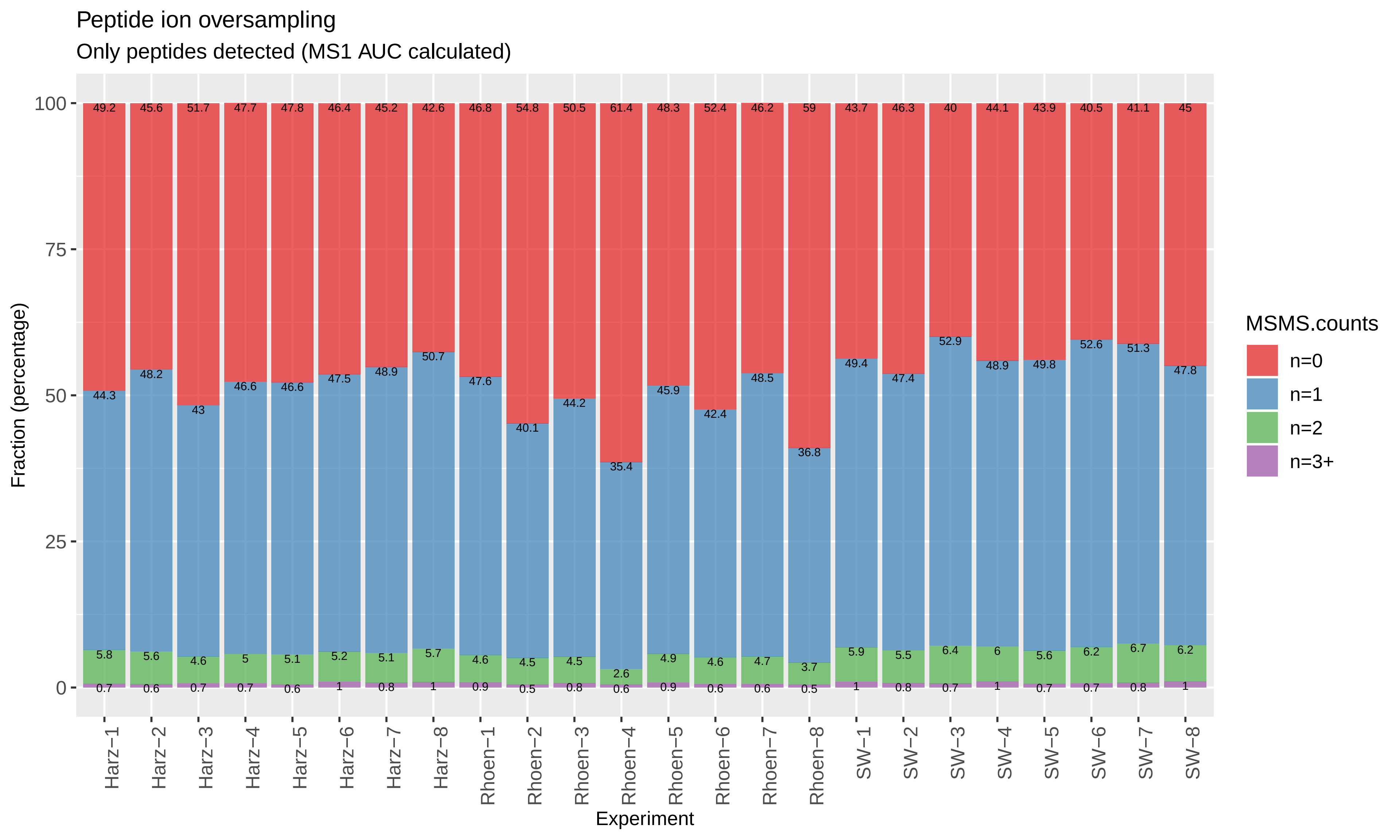

Peptide ion oversampling
Only peptides detected (MS1 AUC calculated)
Fraction (percentage)
Experiment
MSMS.counts
n=0
n=1
n=2
n=3+
Harz−1
Harz−2
Harz−3
Harz−4
Harz−5
Harz−6
Harz−7
Harz−8
Rhoen−1
Rhoen−2
Rhoen−3
Rhoen−4
Rhoen−5
Rhoen−6
Rhoen−7
Rhoen−8
SW−1
SW−2
SW−3
SW−4
SW−5
SW−6
SW−7
SW−8

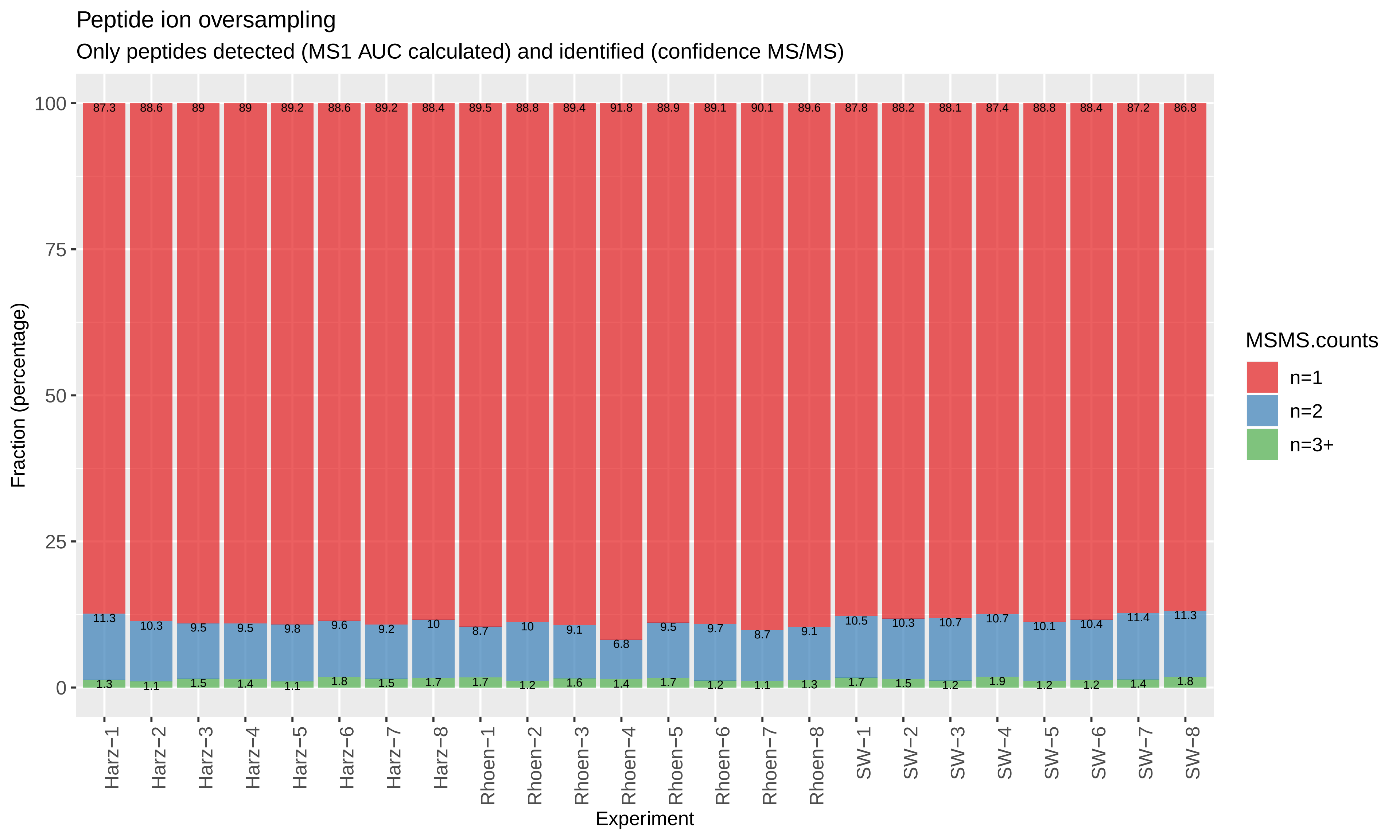

Peptide ion oversampling
Only peptides detected (MS1 AUC calculated) and identified (confidence MS/MS)
Fraction (percentage)
Experiment
MSMS.counts
n=1
n=2
n=3+
Harz−1 Harz−2 Harz−3 Harz−4 Harz−5 Harz−6 Harz−7 Harz−8
Rhoen−1 Rhoen−2 Rhoen−3 Rhoen−4 Rhoen−5 Rhoen−6 Rhoen−7 Rhoen−8
SW−1 SW−2 SW−3 SW−4 SW−5 SW−6 SW−7 SW−8
87.3 88.6 89 89 89.2 88.6 89.2 88.4 89.5 88.8 89.4 91.8 88.9 89.1 90.1 89.6 87.8 88.2 88.1 87.4 88.8 88.4 87.2 86.8
11.3 10.3 9.5 9.5 9.8 9.6 9.2 10 8.7 10 9.1 6.8 9.5 9.7 8.7 9.1 10.5 10.3 10.7 10.7 10.1 10.4 11.4 11.3
1.3 1.1 1.5 1.4 1.1 1.8 1.5 1.7 1.7 1.2 1.6 1.4 1.7 1.2 1.1 1.3 1.7 1.5 1.2 1.9 1.2 1.2 1.4 1.8
