# Supplementary material for: Abiotic and past climatic conditions drive protein abundance variation among natural populations of the caddisfly Crunoecia irrorata
Source: Sci Rep. 2020 Sep 23;10:15538. doi: 10.1038/s41598-020-72569-4 (PMC7512004; doi:10.1038/s41598-020-72569-4)
Supplement: Supplementary file 2 — Supplementary Information 2. [file 41598_2020_72569_MOESM2_ESM.zip › SI3_artMS_QC/QC_Plots_PEPTIDES.pdf]

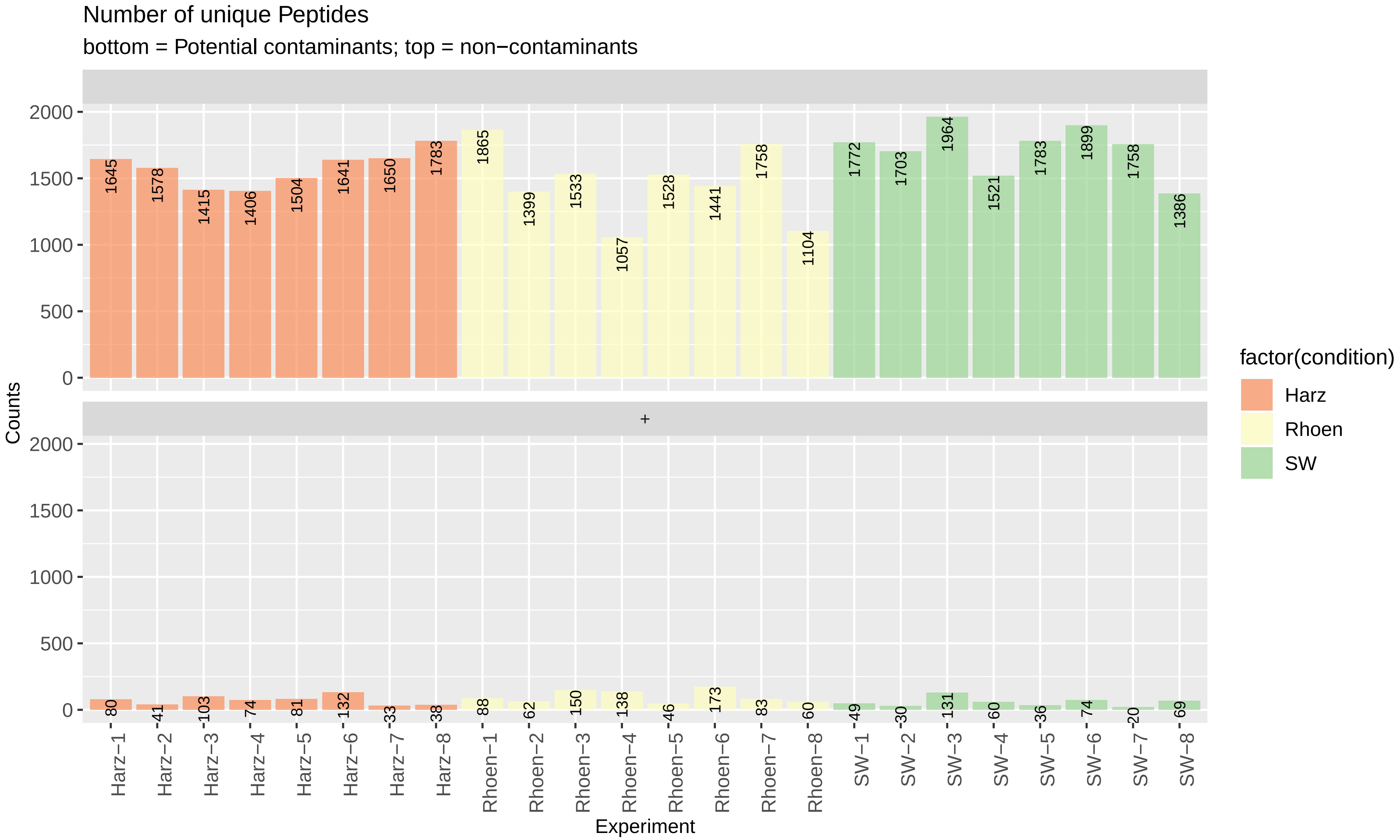
Number of unique Peptides
bottom = Potential contaminants; top = non−contaminants
Counts
Experiment
+
factor(condition)
Harz
Rhoen
SW
Harz−1 Harz−2 Harz−3 Harz−4 Harz−5 Harz−6 Harz−7 Harz−8
Rhoen−1 Rhoen−2 Rhoen−3 Rhoen−4 Rhoen−5 Rhoen−6 Rhoen−7 Rhoen−8
SW−1 SW−2 SW−3 SW−4 SW−5 SW−6 SW−7 SW−8
1645 1578 1415 1406 1504 1641 1650 1783
1865 1399 1533 1057 1528 1441 1758 1104
1772 1703 1964 1521 1783 1899 1758 1386
80 41 103 74 81 132 33 38
88 62 150 138 46 173 83 60
49 30 131 60 36 74 20 69

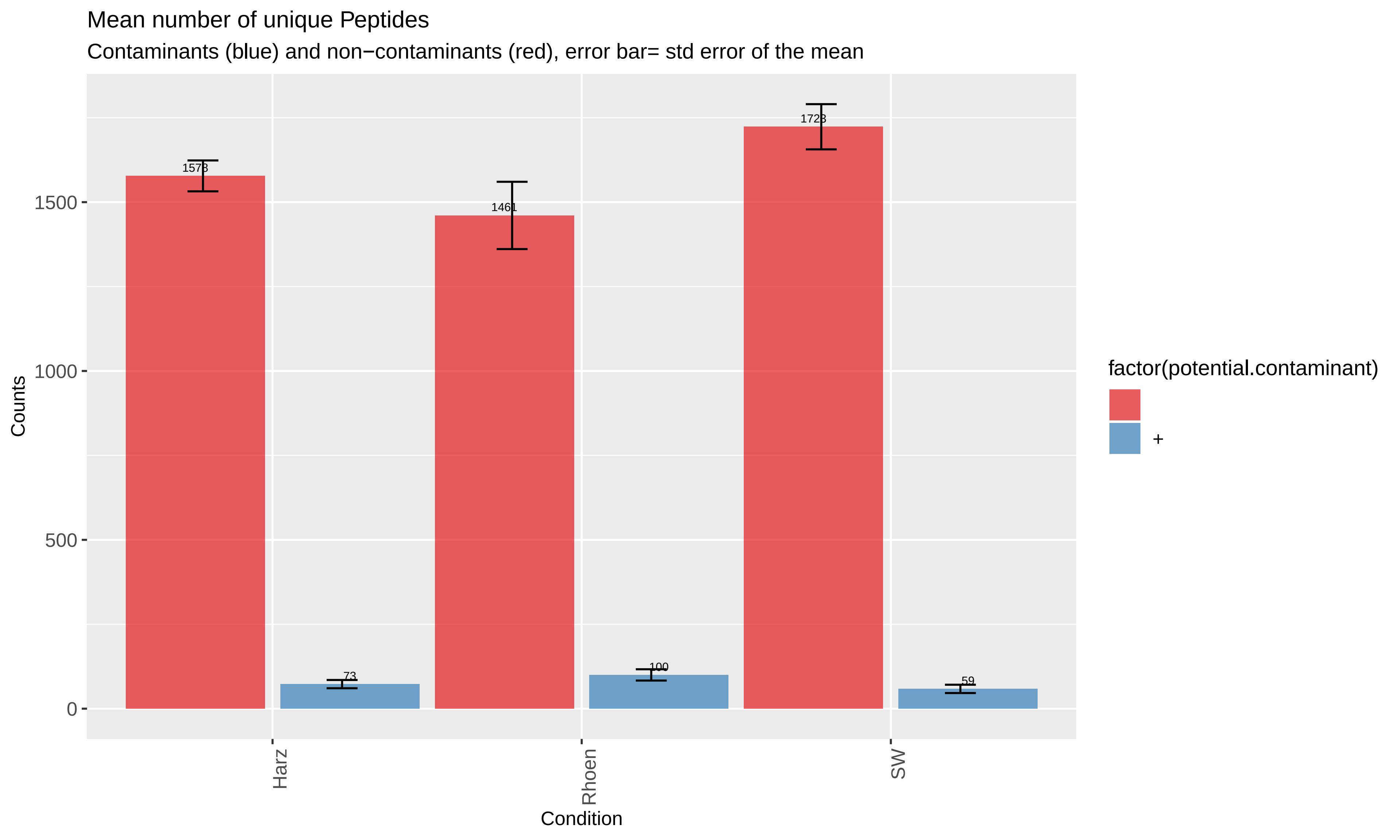
Mean number of unique Peptides
Contaminants (blue) and non−contaminants (red), error bar= std error of the mean
1578
1461
1723
73
100
59
1500
1000
500
0
Counts
Harz
Rhoen
SW
Condition
factor(potential.contaminant)
+
