# Supplementary material for: Abiotic and past climatic conditions drive protein abundance variation among natural populations of the caddisfly Crunoecia irrorata
Source: Sci Rep. 2020 Sep 23;10:15538. doi: 10.1038/s41598-020-72569-4 (PMC7512004; doi:10.1038/s41598-020-72569-4)
Supplement: Supplementary file 2 — Supplementary Information 2. [file 41598_2020_72569_MOESM2_ESM.zip › SI3_artMS_QC/QC_Plots_ProtDetect.pdf]

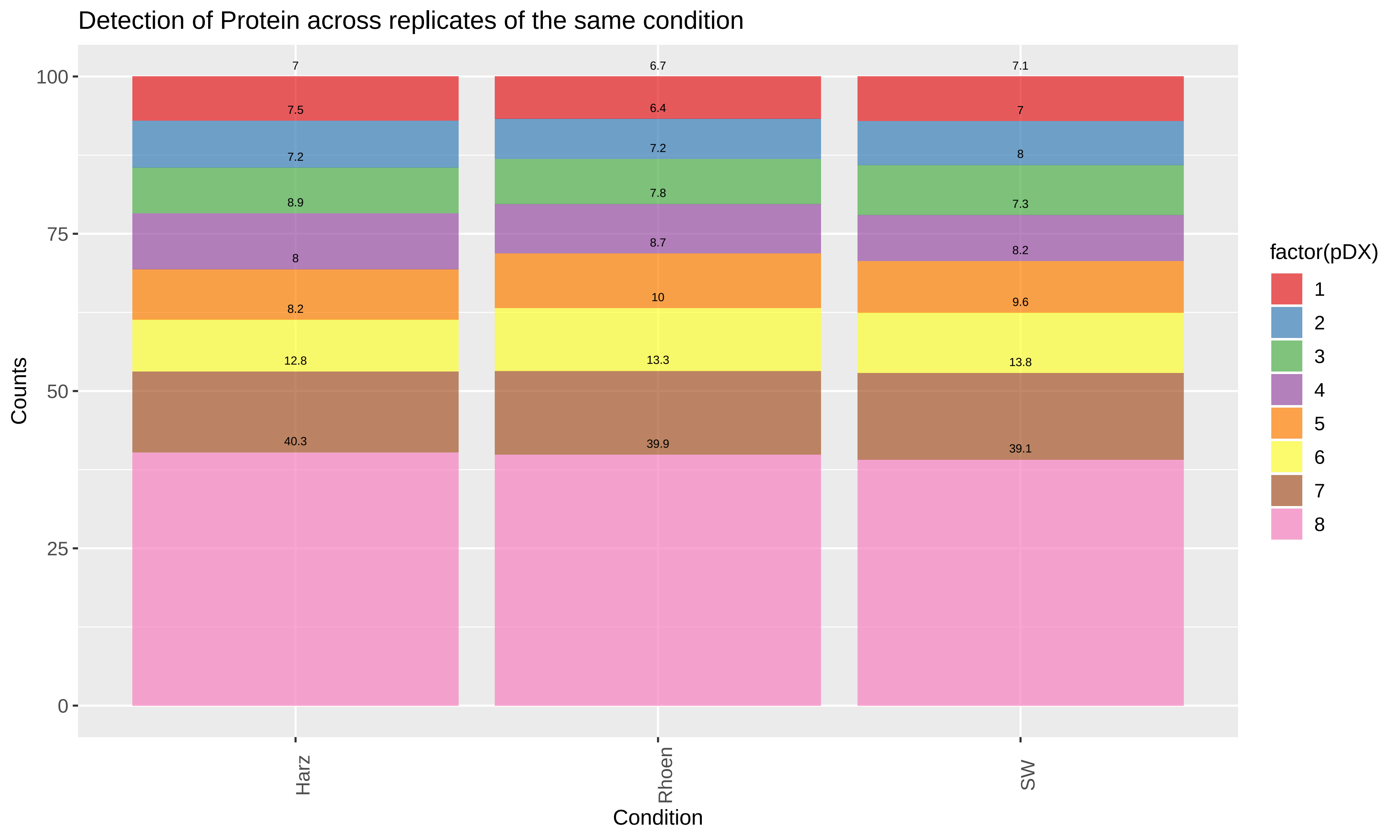
Detection of Protein across replicates of the same condition
Counts
Condition
Harz
Rhoen
SW
factor(pDX)
1
2
3
4
5
6
7
8
0
25
50
75
100
7
7.5
7.2
8.9
8
8.2
12.8
40.3
6.7
6.4
7.2
7.8
8.7
10
13.3
39.9
7.1
7
8
7.3
8.2
9.6
13.8
39.1

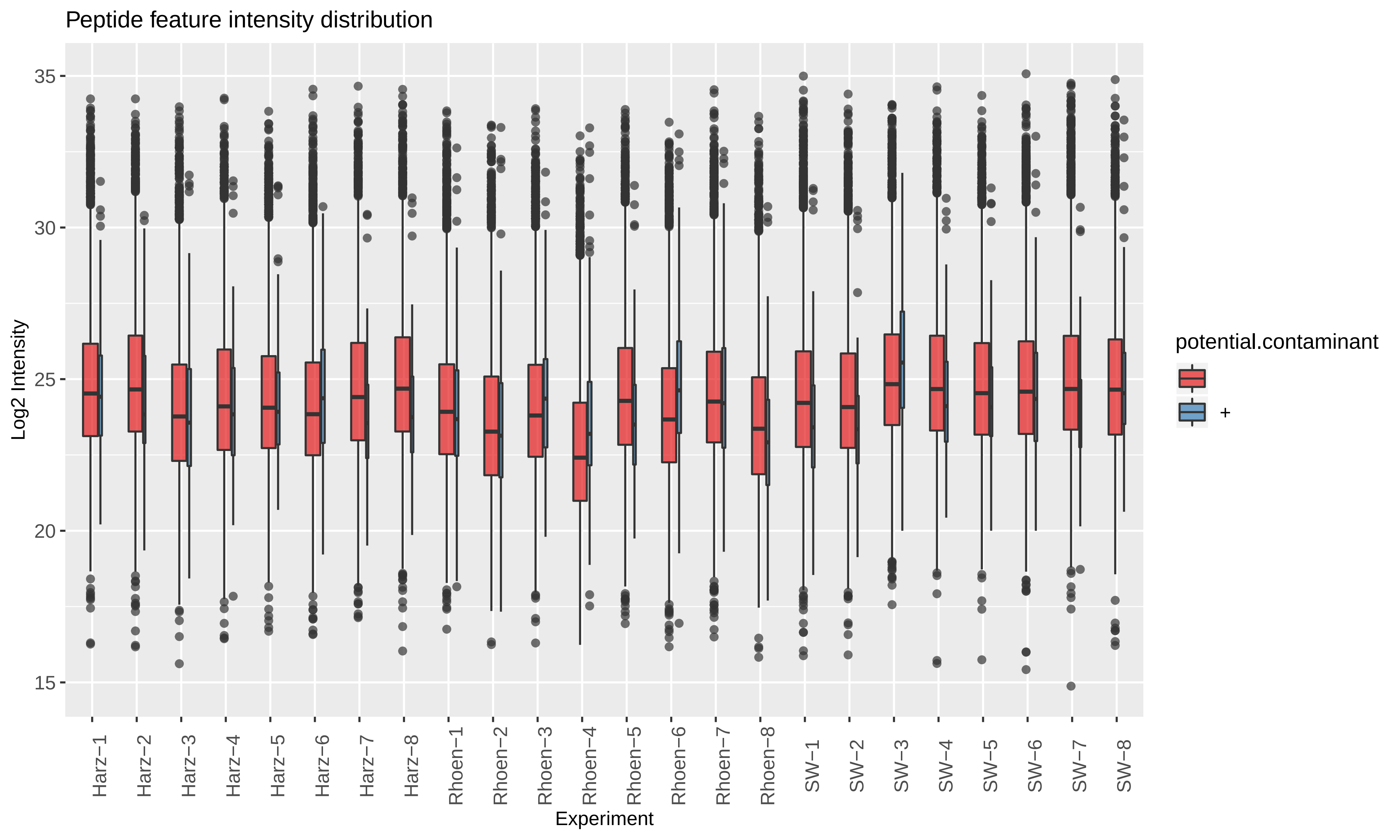

Peptide feature intensity distribution
Log2 Intensity
35
30
25
20
15
Harz−1
Harz−2
Harz−3
Harz−4
Harz−5
Harz−6
Harz−7
Harz−8
Rhoen−1
Rhoen−2
Rhoen−3
Rhoen−4
Rhoen−5
Rhoen−6
Rhoen−7
Rhoen−8
SW−1
SW−2
SW−3
SW−4
SW−5
SW−6
SW−7
SW−8
Experiment
potential.contaminant
+

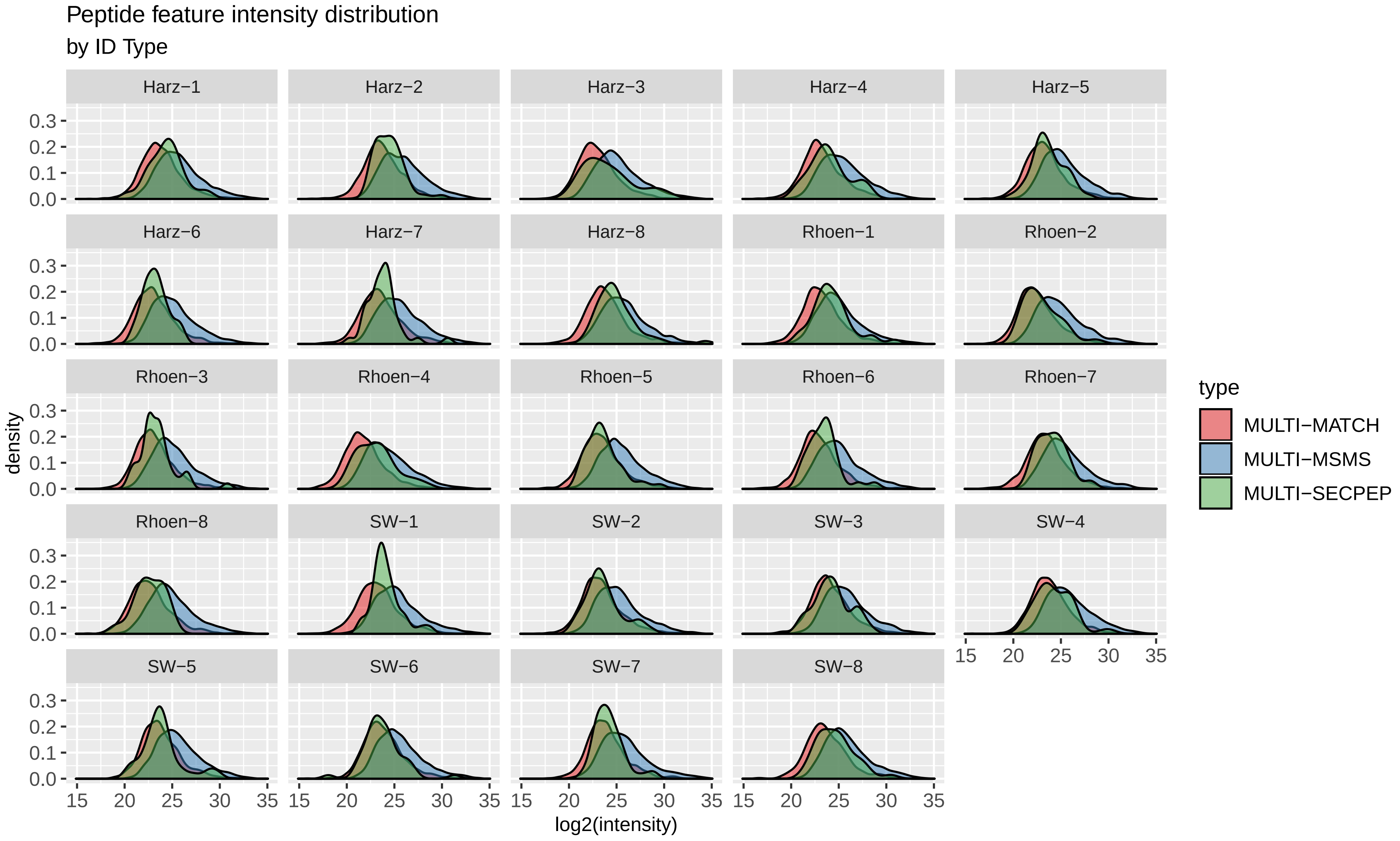
Peptide feature intensity distribution
by ID Type
Harz−1
Harz−2
Harz−3
Harz−4
Harz−5
Harz−6
Harz−7
Harz−8
Rhoen−1
Rhoen−2
Rhoen−3
Rhoen−4
Rhoen−5
Rhoen−6
Rhoen−7
Rhoen−8
SW−1
SW−2
SW−3
SW−4
SW−5
SW−6
SW−7
SW−8
density
log2(intensity)
type
MULTI−MATCH
MULTI−MSMS
MULTI−SECPEP
