# Supplementary material for: Abiotic and past climatic conditions drive protein abundance variation among natural populations of the caddisfly Crunoecia irrorata
Source: Sci Rep. 2020 Sep 23;10:15538. doi: 10.1038/s41598-020-72569-4 (PMC7512004; doi:10.1038/s41598-020-72569-4)
Supplement: Supplementary file 2 — Supplementary Information 2. [file 41598_2020_72569_MOESM2_ESM.zip › SI3_artMS_QC/QC_Plots_PROTEINS.pdf]

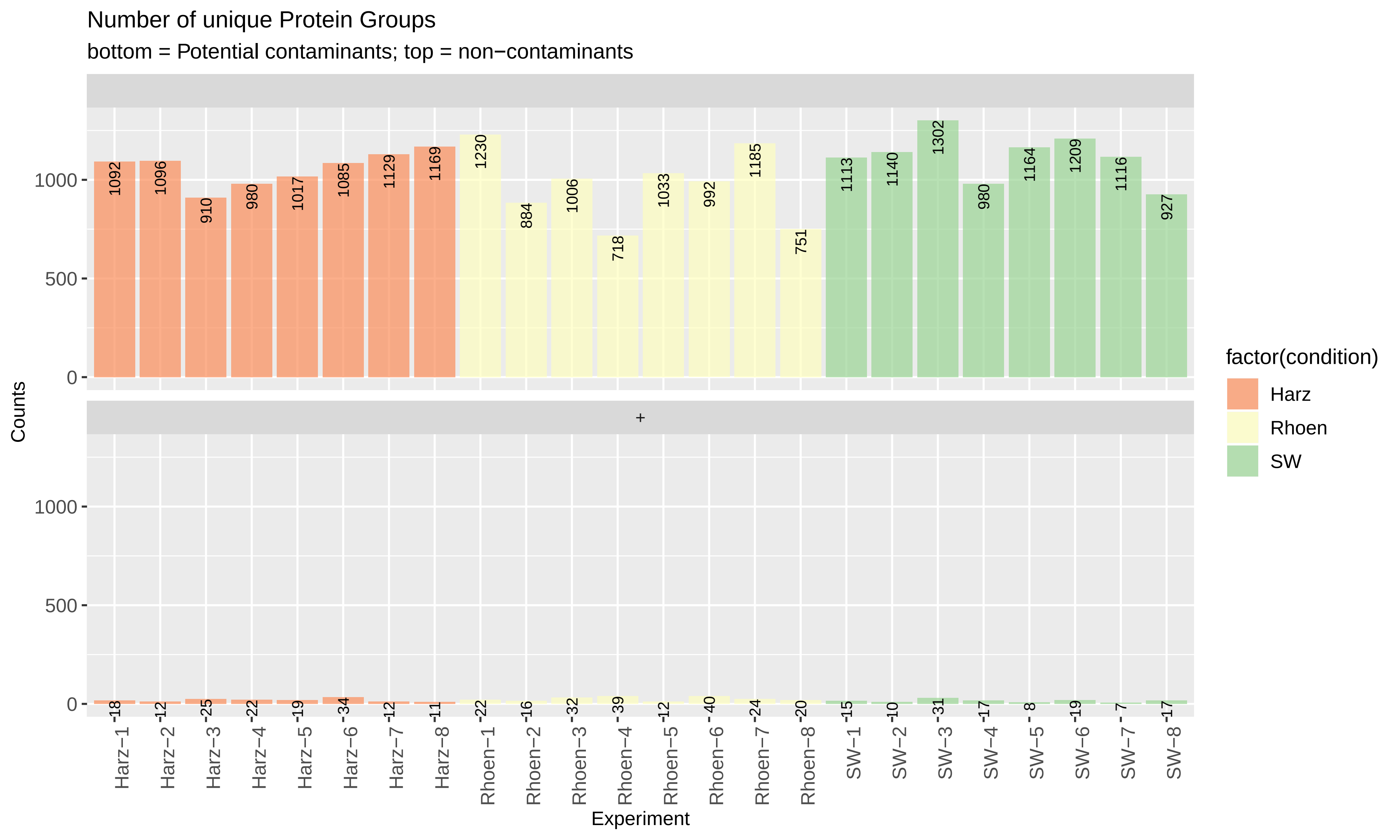

Number of unique Protein Groups
bottom = Potential contaminants; top = non−contaminants
+
Counts
Experiment
factor(condition)
Harz
Rhoen
SW
1092
1096
910
980
1017
1085
1129
1169
1230
884
1006
718
1033
992
1185
751
1113
1140
1302
980
1164
1209
1116
927
-18
-12
-25
-22
-19
-34
-12
-11
-22
-16
-32
-39
-12
-40
-24
-20
-15
-10
-31
-17
-8
-19
-7
-17
Harz−1
Harz−2
Harz−3
Harz−4
Harz−5
Harz−6
Harz−7
Harz−8
Rhoen−1
Rhoen−2
Rhoen−3
Rhoen−4
Rhoen−5
Rhoen−6
Rhoen−7
Rhoen−8
SW−1
SW−2
SW−3
SW−4
SW−5
SW−6
SW−7
SW−8

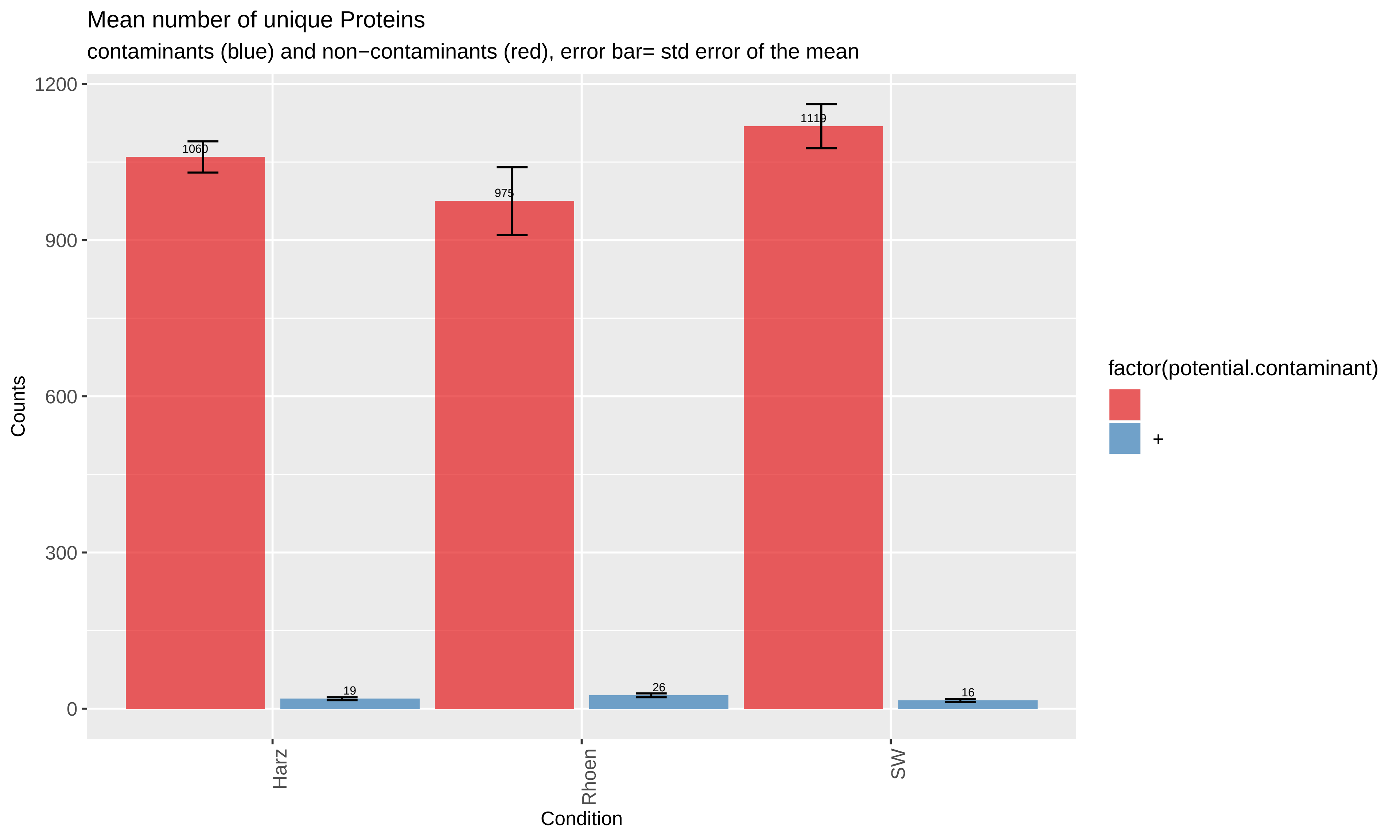

Mean number of unique Proteins
contaminants (blue) and non−contaminants (red), error bar= std error of the mean
1200
900
600
300
0
Counts
1060
19
975
26
1119
16
Harz
Rhoen
SW
Condition
factor(potential.contaminant)
+
