# Supplementary material for: Abiotic and past climatic conditions drive protein abundance variation among natural populations of the caddisfly Crunoecia irrorata
Source: Sci Rep. 2020 Sep 23;10:15538. doi: 10.1038/s41598-020-72569-4 (PMC7512004; doi:10.1038/s41598-020-72569-4)
Supplement: Supplementary file 2 — Supplementary Information 2. [file 41598_2020_72569_MOESM2_ESM.zip › SI3_artMS_QC/QC_Plots_ProtInt.pdf]

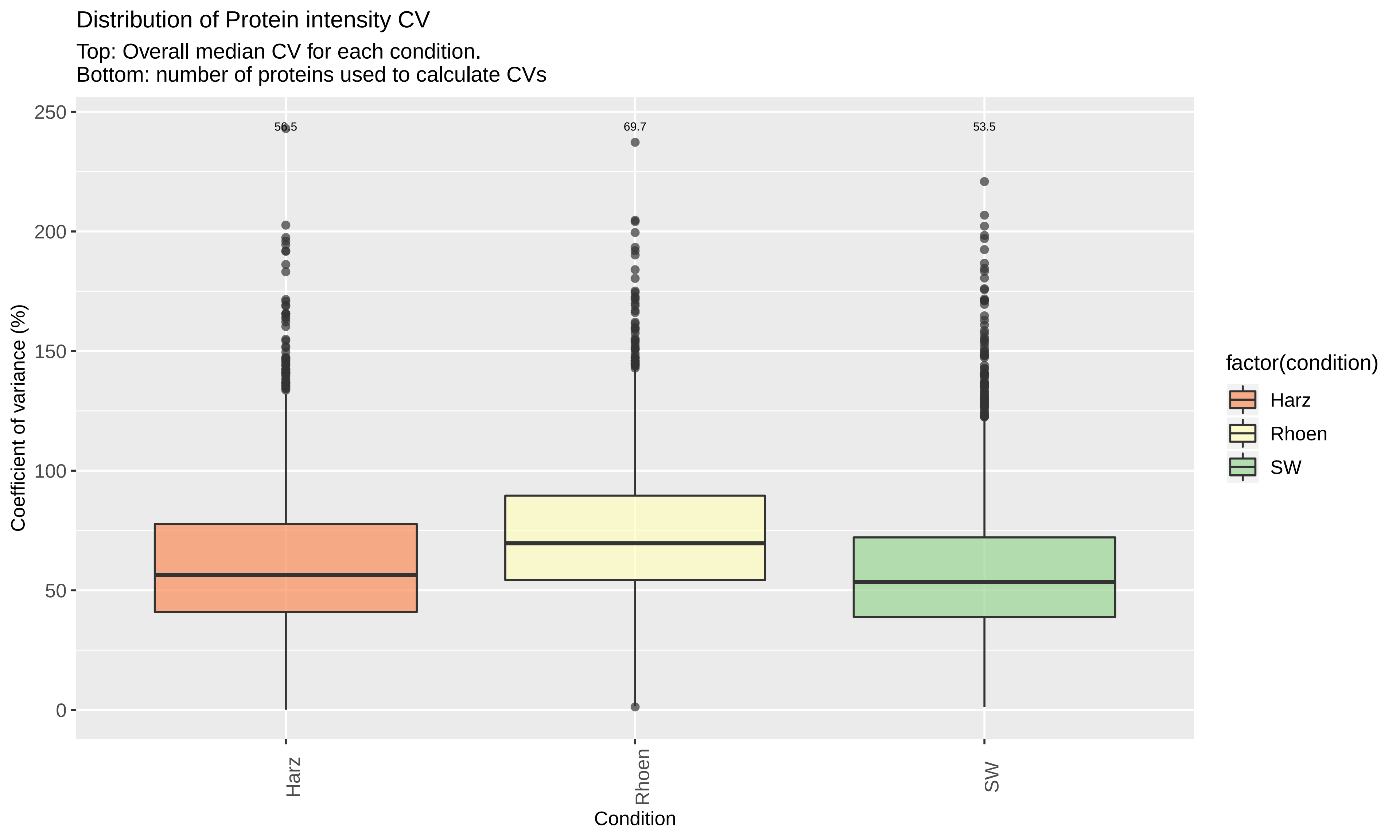

Distribution of Protein intensity CV
Top: Overall median CV for each condition.
Bottom: number of proteins used to calculate CVs
56.5
69.7
53.5
250
200
150
100
50
0
Coefficient of variance (%)
Harz
Rhoen
SW
Condition
factor(condition)
Harz
Rhoen
SW

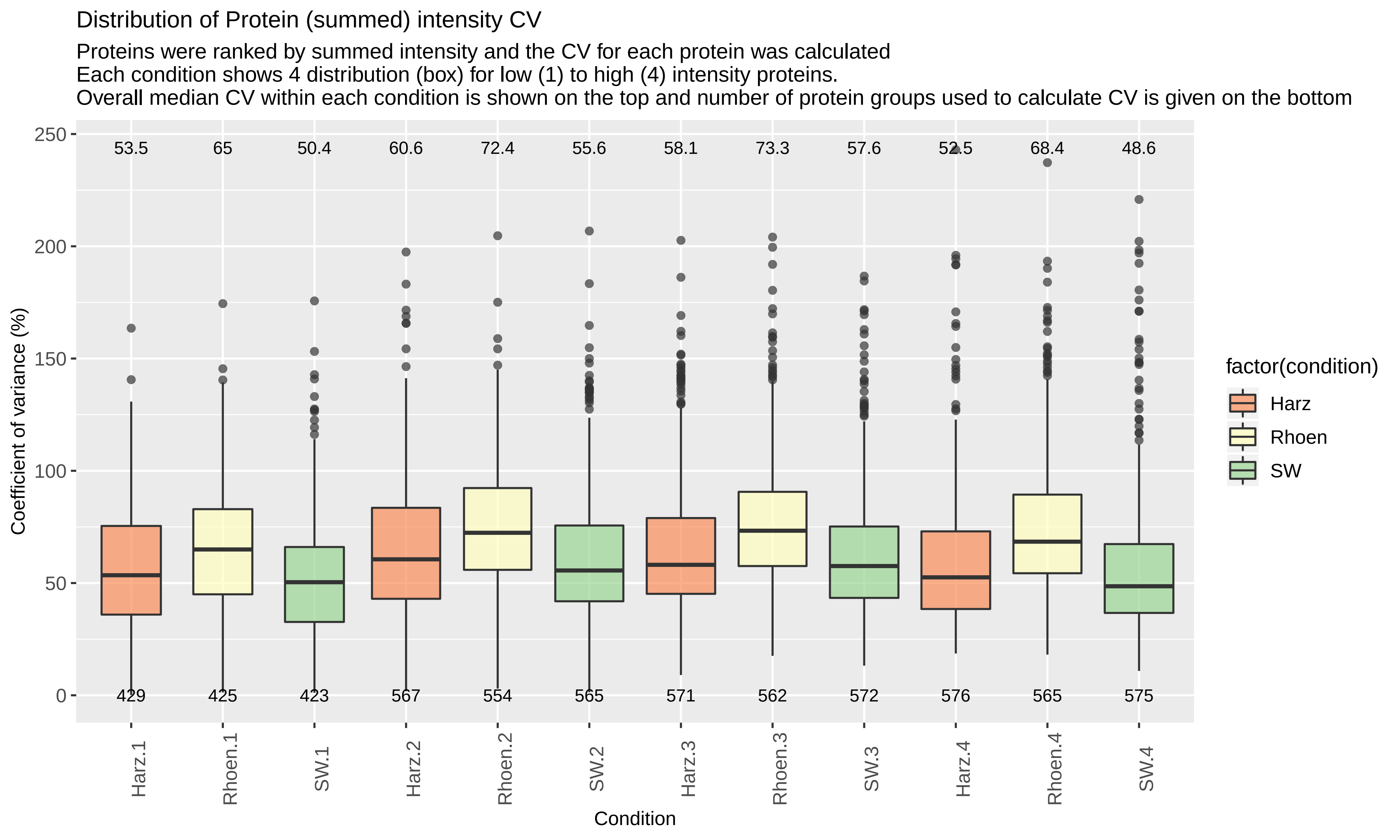
Distribution of Protein (summed) intensity CV
Proteins were ranked by summed intensity and the CV for each protein was calculated
Each condition shows 4 distribution (box) for low (1) to high (4) intensity proteins.
Overall median CV within each condition is shown on the top and number of protein groups used to calculate CV is given on the bottom
Coefficient of variance (%)
250
200
150
100
50
0
53.5
65
50.4
60.6
72.4
55.6
58.1
73.3
57.6
52.5
68.4
48.6
429
425
423
567
554
565
571
562
572
576
565
575
Harz.1
Rhoen.1
SW.1
Harz.2
Rhoen.2
SW.2
Harz.3
Rhoen.3
SW.3
Harz.4
Rhoen.4
SW.4
Condition
factor(condition)
Harz
Rhoen
SW
