# Supplementary material for: Abiotic and past climatic conditions drive protein abundance variation among natural populations of the caddisfly Crunoecia irrorata
Source: Sci Rep. 2020 Sep 23;10:15538. doi: 10.1038/s41598-020-72569-4 (PMC7512004; doi:10.1038/s41598-020-72569-4)
Supplement: Supplementary file 2 — Supplementary Information 2. [file 41598_2020_72569_MOESM2_ESM.zip › SI3_artMS_QC/QC_Plots_PSM.pdf]

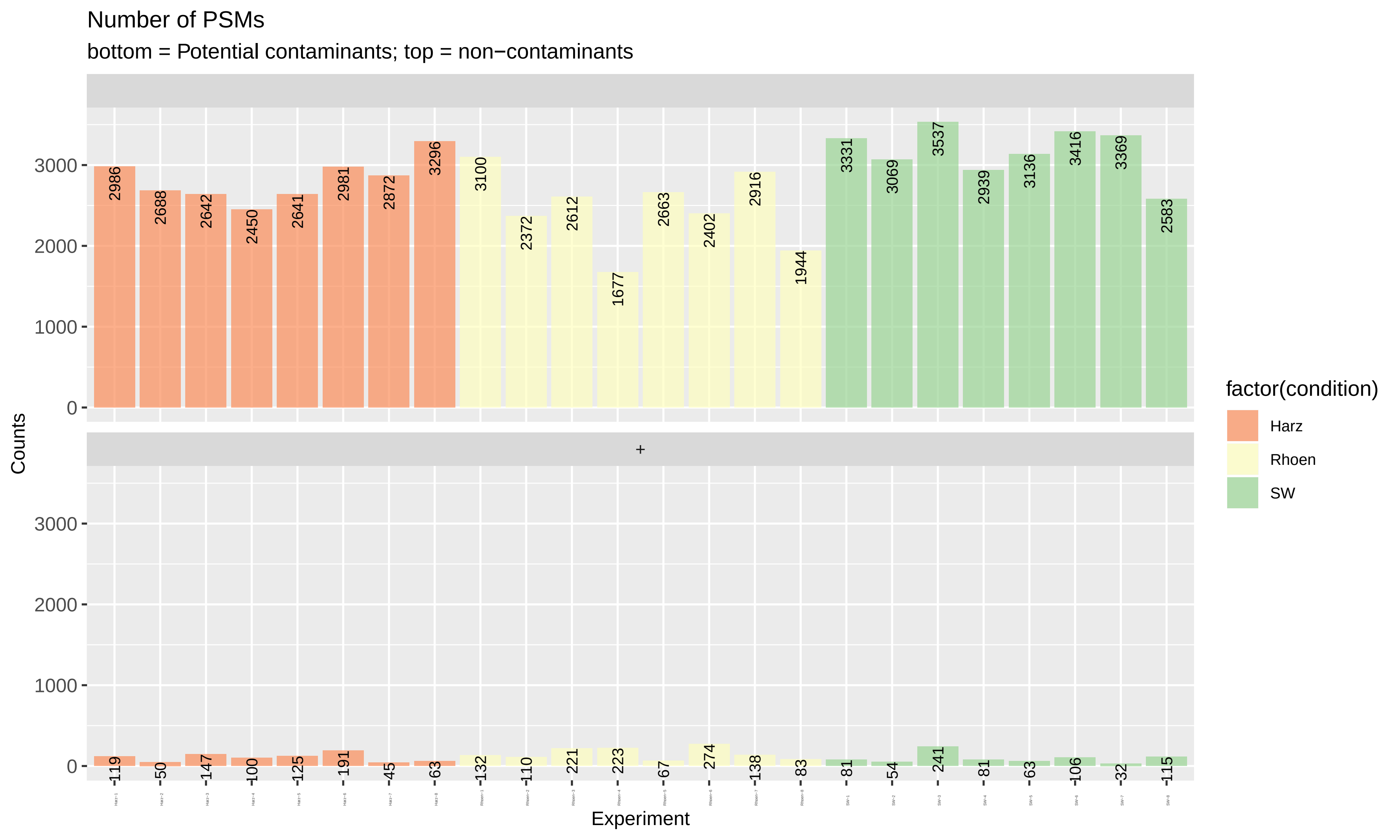

Number of PSMs
bottom = Potential contaminants; top = non−contaminants
Counts
Experiment
factor(condition)
Harz
Rhoen
SW
+
0
1000
2000
3000
2986
2688
2642
2450
2641
2981
2872
3296
3100
2372
2612
1677
2663
2402
2916
1944
3331
3069
3537
2939
3136
3416
3369
2583
119
50
147
100
125
191
45
63
132
110
221
223
67
274
138
83
81
54
241
81
63
106
32
115

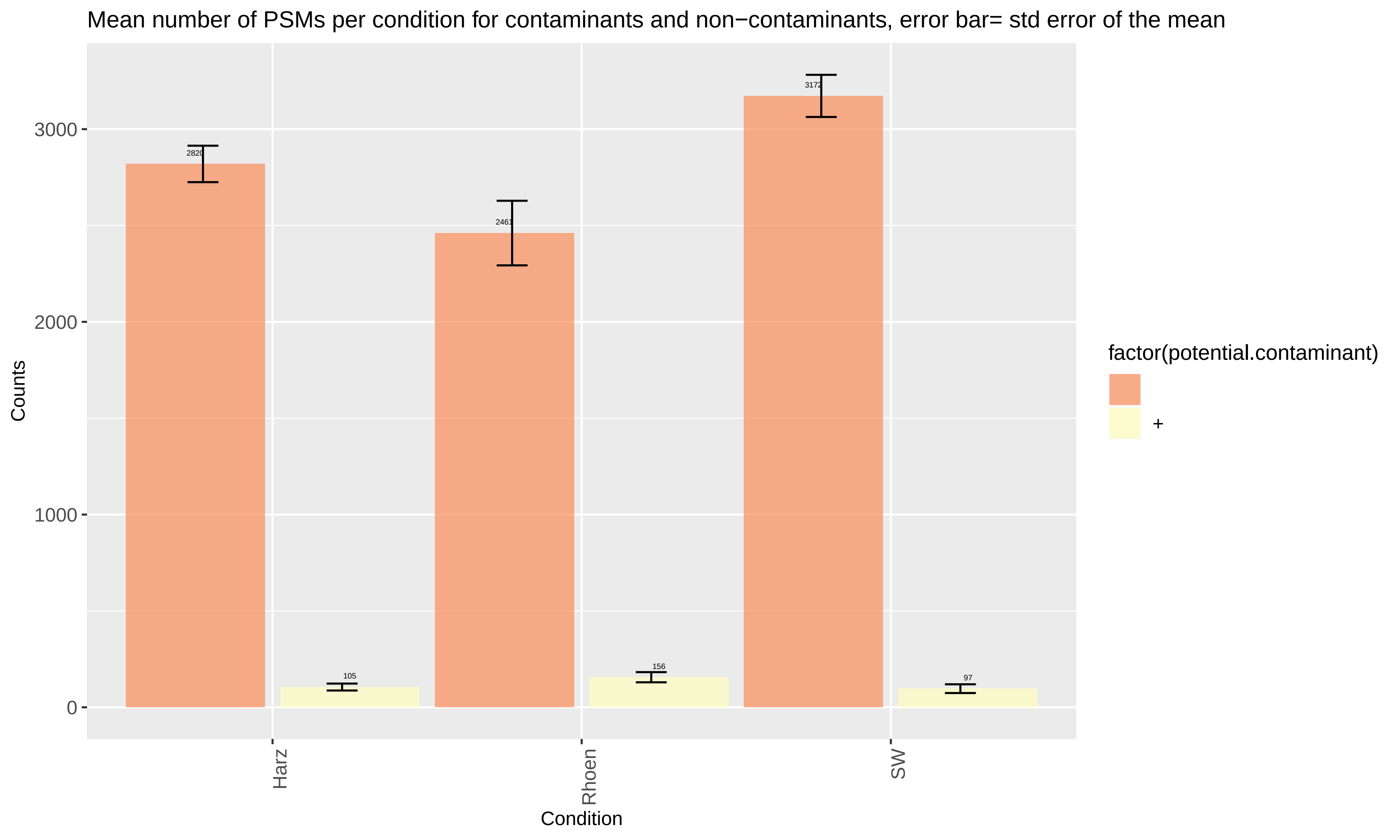
Mean number of PSMs per condition for contaminants and non−contaminants, error bar= std error of the mean
Counts
3000
2000
1000
0
2820
105
2461
156
3172
97
Harz
Rhoen
SW
Condition
factor(potential.contaminant)
+
