# Supplementary material for: Abiotic and past climatic conditions drive protein abundance variation among natural populations of the caddisfly Crunoecia irrorata
Source: Sci Rep. 2020 Sep 23;10:15538. doi: 10.1038/s41598-020-72569-4 (PMC7512004; doi:10.1038/s41598-020-72569-4)

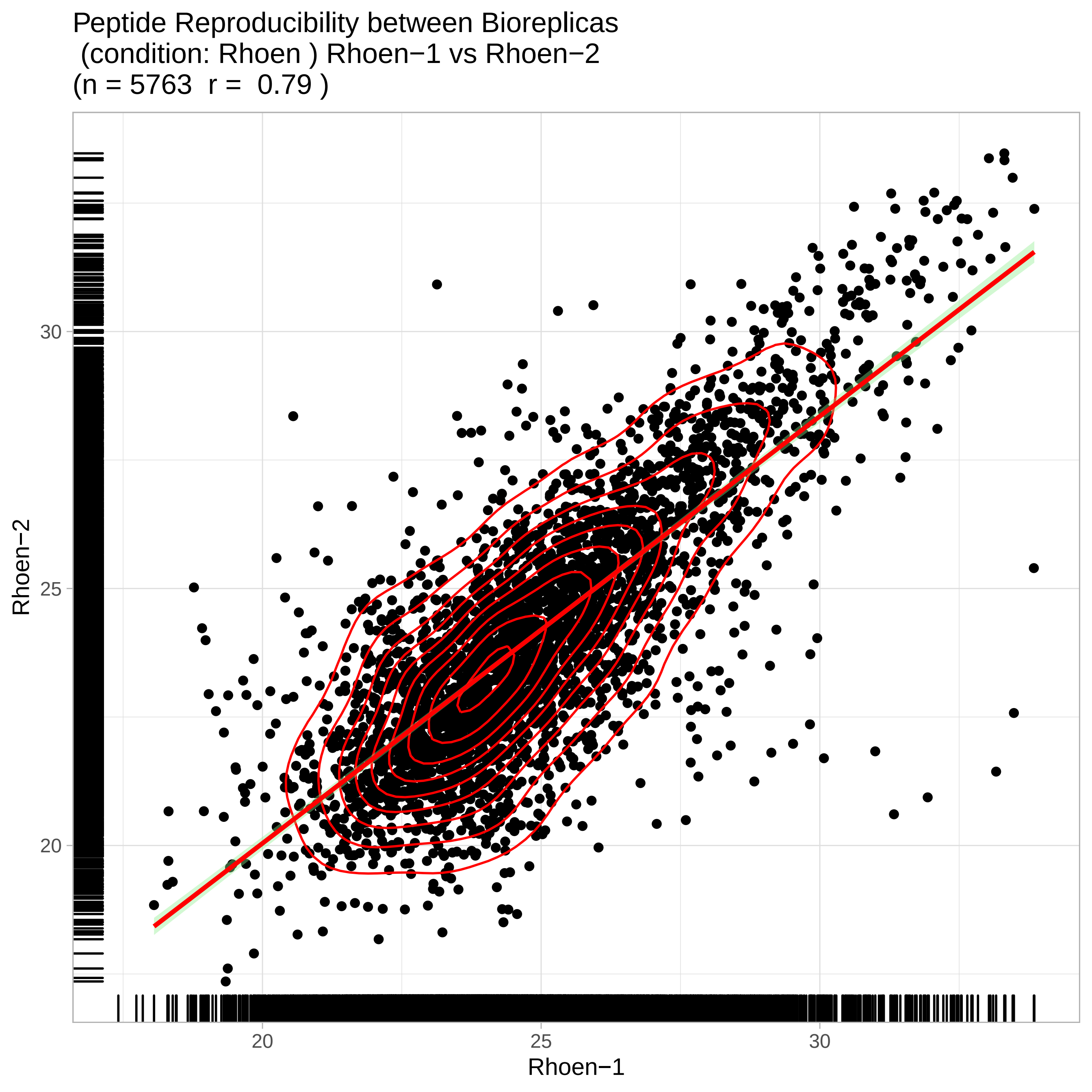
Peptide Reproducibility between Bioreplicas
(condition: Rhoen) Rhoen−1 vs Rhoen−2
(n = 5763 r = 0.79)
Rhoen−2
Rhoen−1
20
25
30
20
25
30

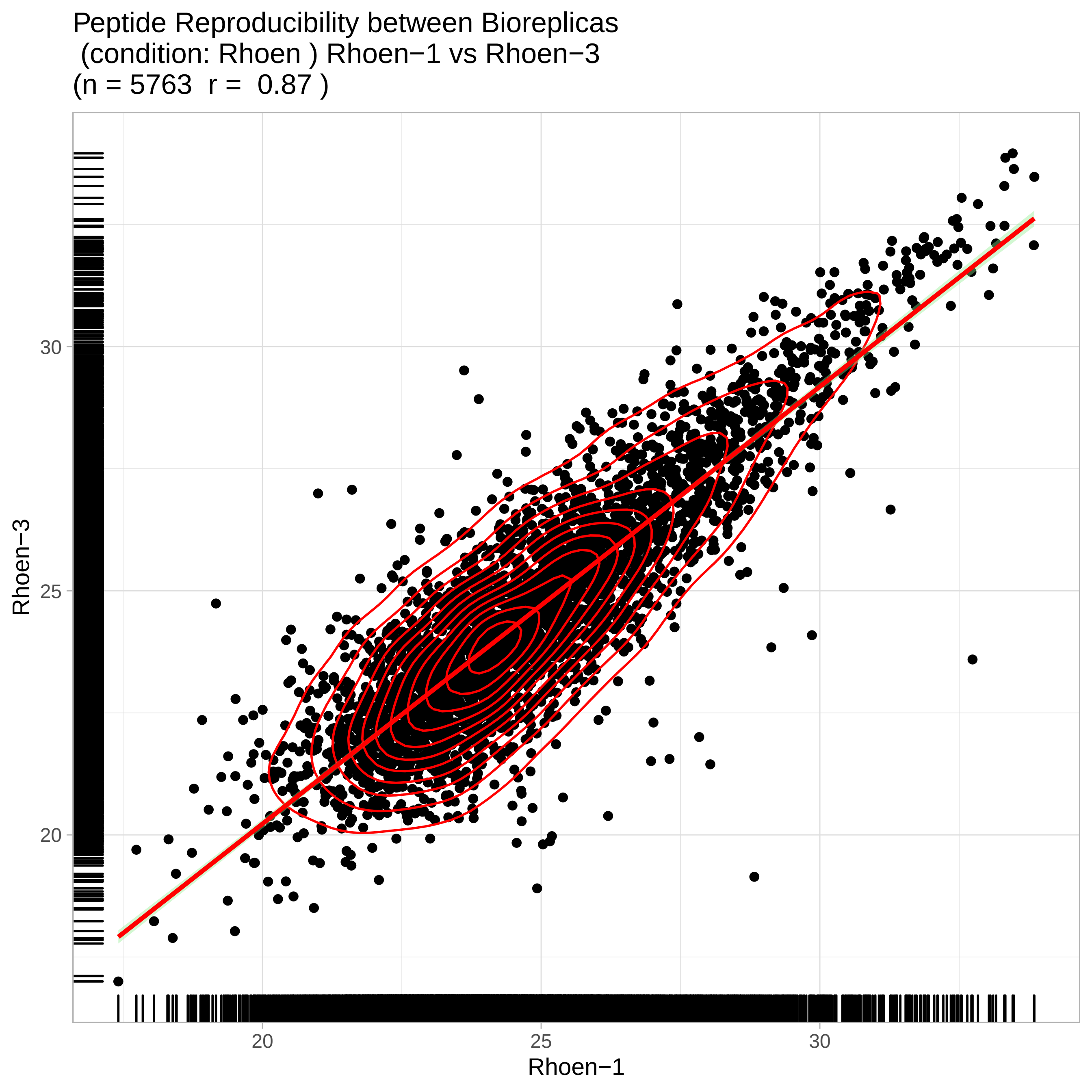
Peptide Reproducibility between Bioreplicas
(condition: Rhoen) Rhoen−1 vs Rhoen−3
(n = 5763 r = 0.87)
Rhoen−3
30
25
20
20
25
30
Rhoen−1

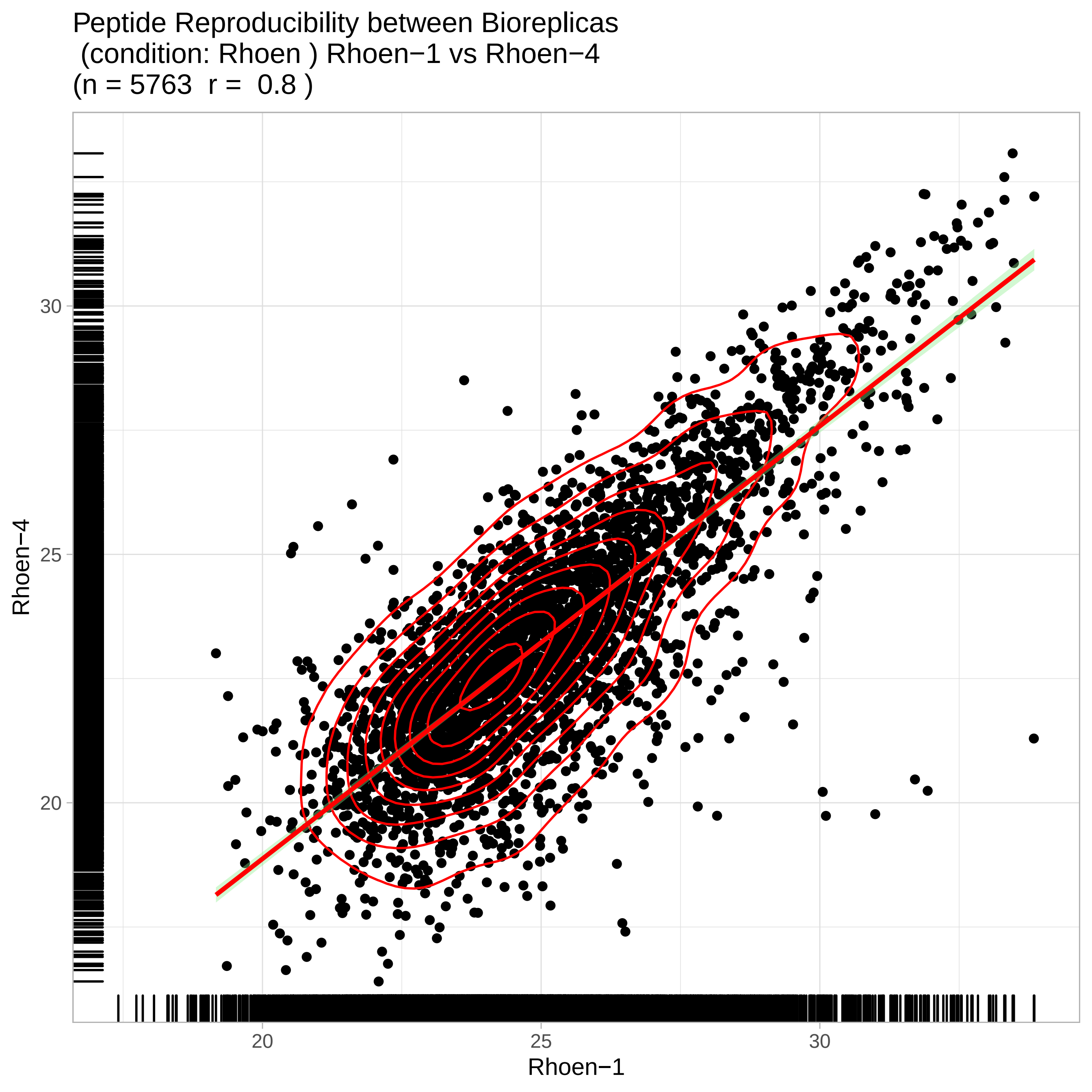
Peptide Reproducibility between Bioreplicas
(condition: Rhoen) Rhoen−1 vs Rhoen−4
(n = 5763 r = 0.8)
Rhoen−4
30
25
20
20
25
30
Rhoen−1

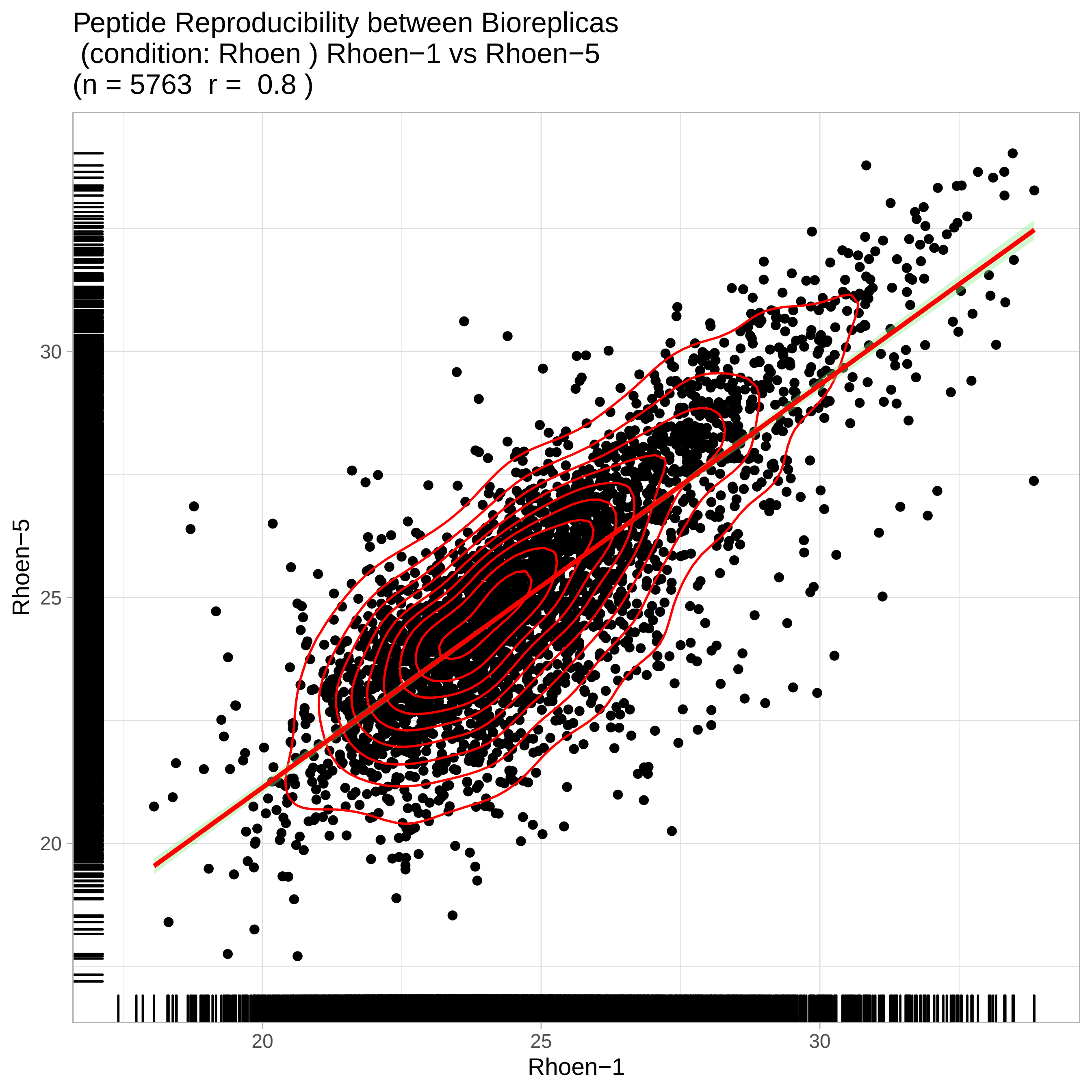
Peptide Reproducibility between Bioreplicas
(condition: Rhoen) Rhoen−1 vs Rhoen−5
(n = 5763 r = 0.8)
Rhoen−5
Rhoen−1
20
25
30
20
25
30

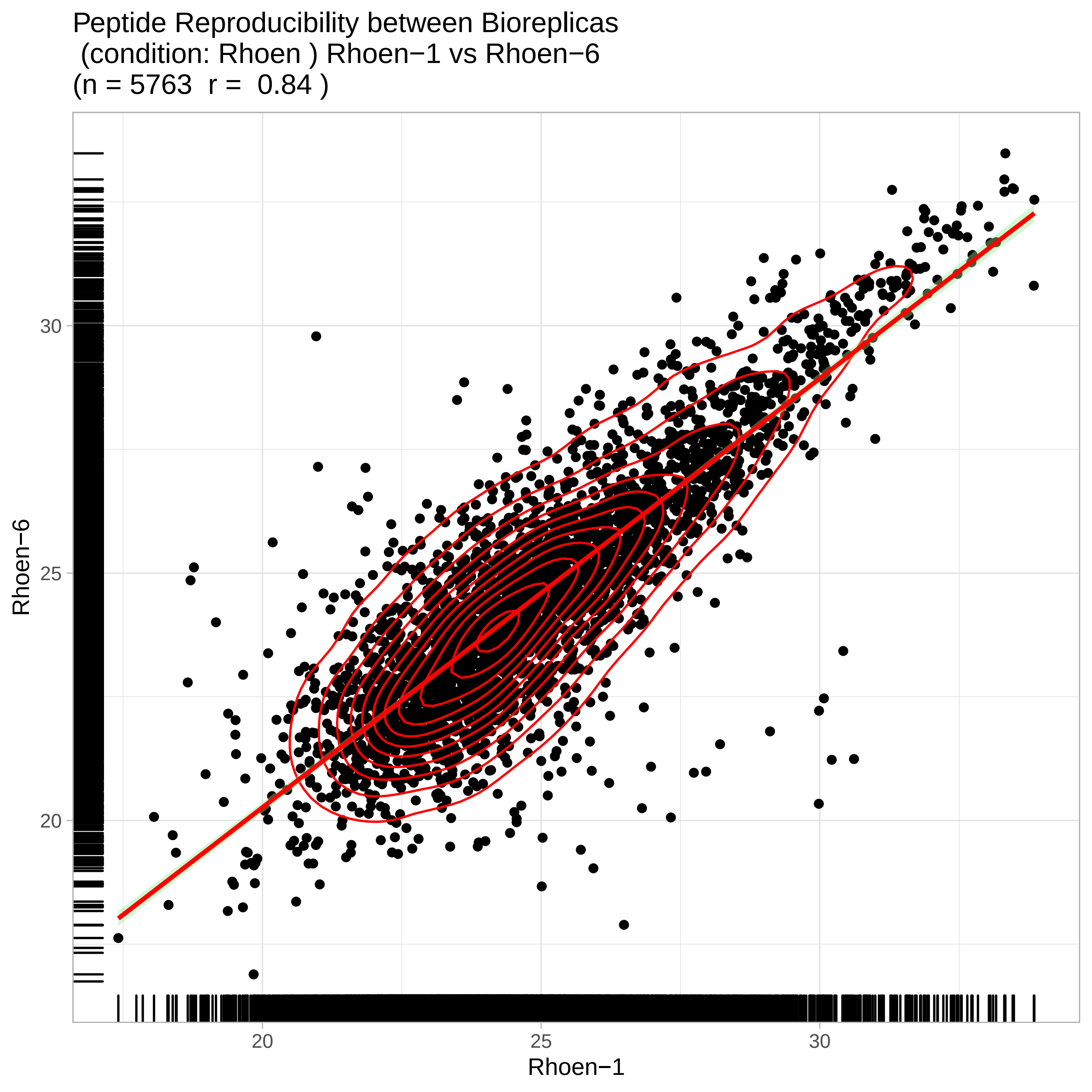

Peptide Reproducibility between Bioreplicas
(condition: Rhoen) Rhoen−1 vs Rhoen−6
(n = 5763 r = 0.84)
30
25
20
Rhoen−6
20
25
30
Rhoen−1

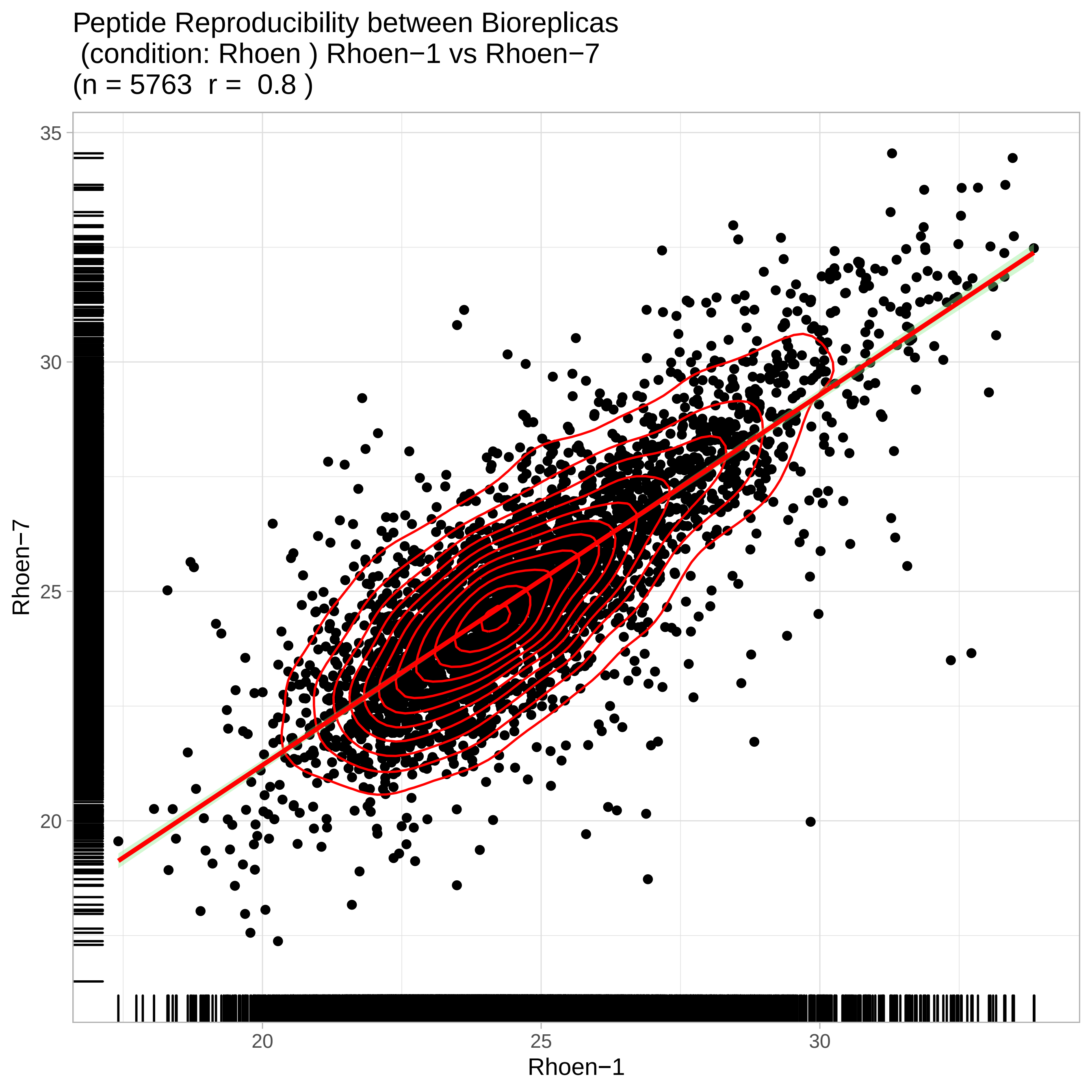

Peptide Reproducibility between Bioreplicas
(condition: Rhoen) Rhoen−1 vs Rhoen−7
(n = 5763 r = 0.8)
35
30
25
20
Rhoen−7
20
25
30
Rhoen−1

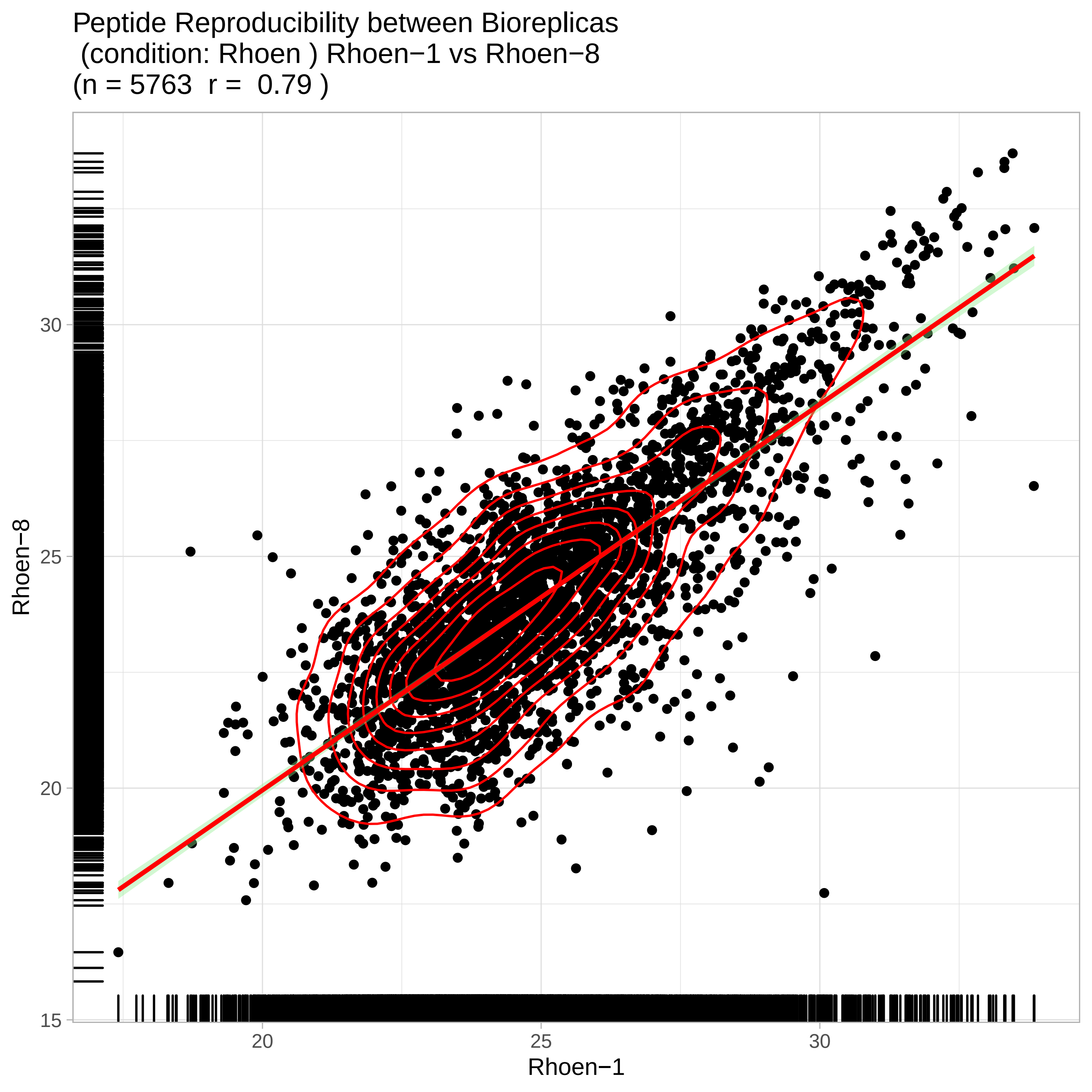
Peptide Reproducibility between Bioreplicas
(condition: Rhoen) Rhoen−1 vs Rhoen−8
(n = 5763 r = 0.79)
Rhoen−8
Rhoen−1
15
20
25
30
20
25
30

Peptide Reproducibility between Bioreplicas
(condition: Rhoen) Rhoen−2 vs Rhoen−3
(n = 5763 r = 0.78)

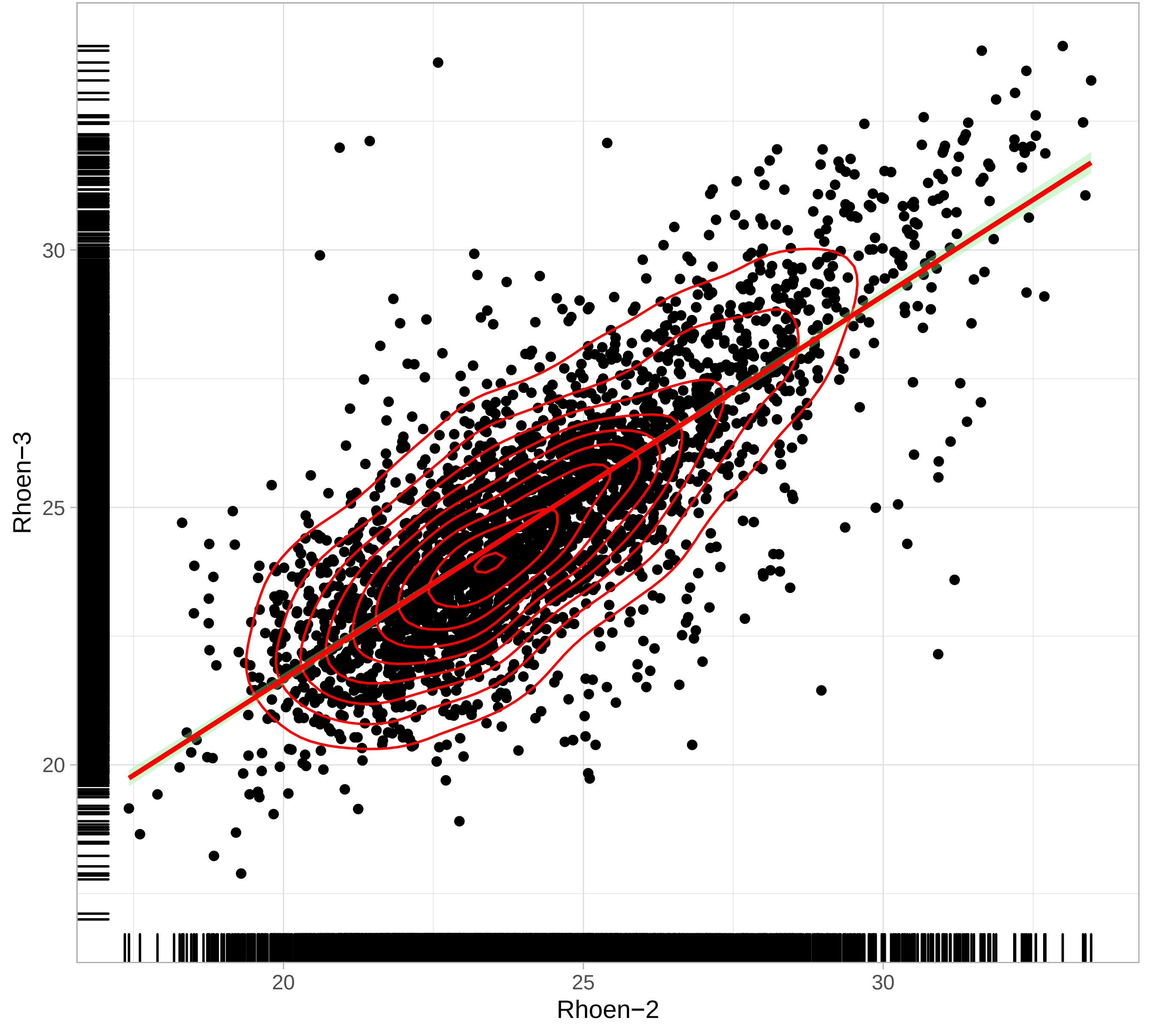

Peptide Reproducibility between Bioreplicas
(condition: Rhoen) Rhoen−2 vs Rhoen−4
(n = 5763 r = 0.86)

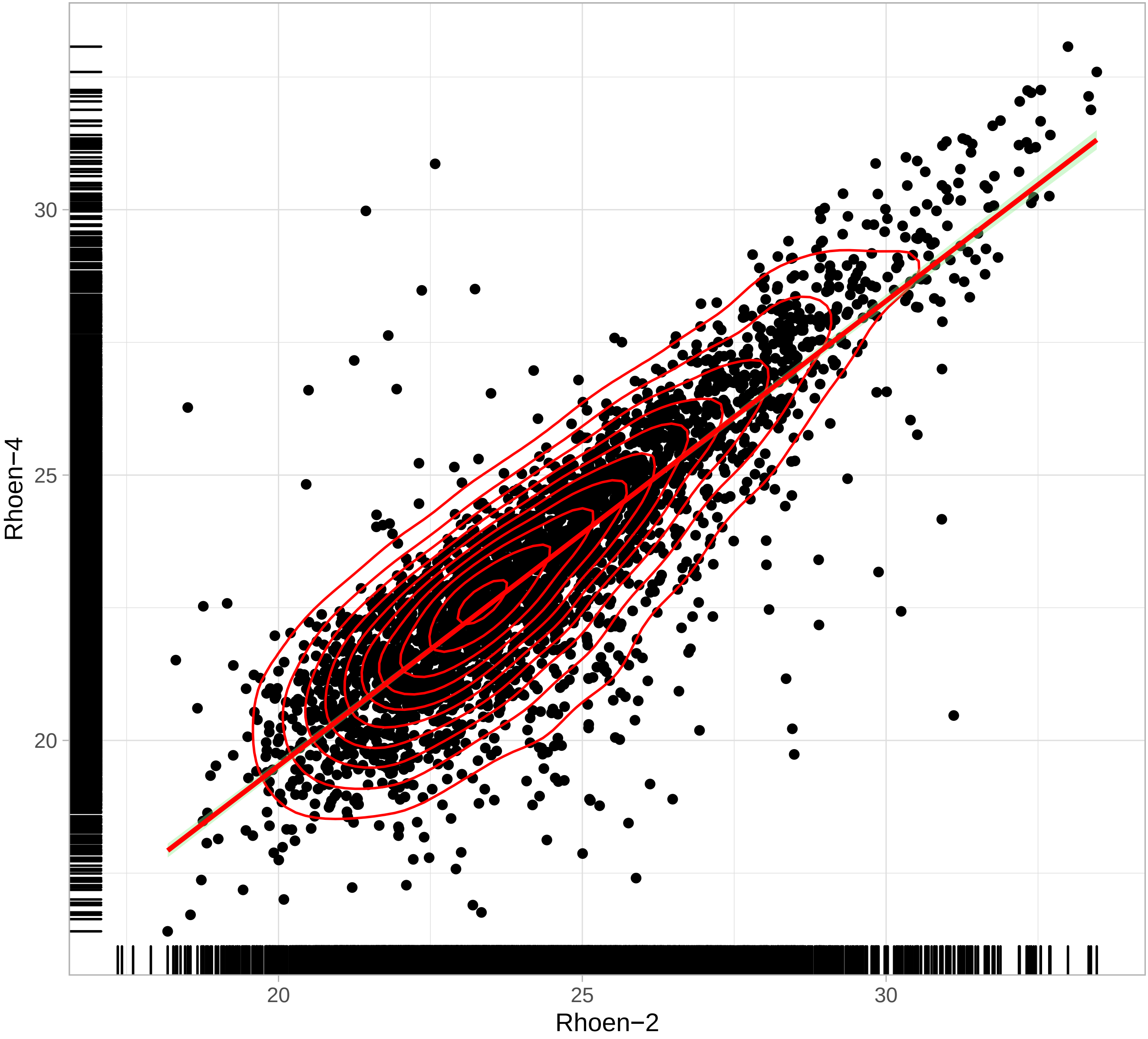

Peptide Reproducibility between Bioreplicas
(condition: Rhoen) Rhoen−2 vs Rhoen−5
(n = 5763 r = 0.8)

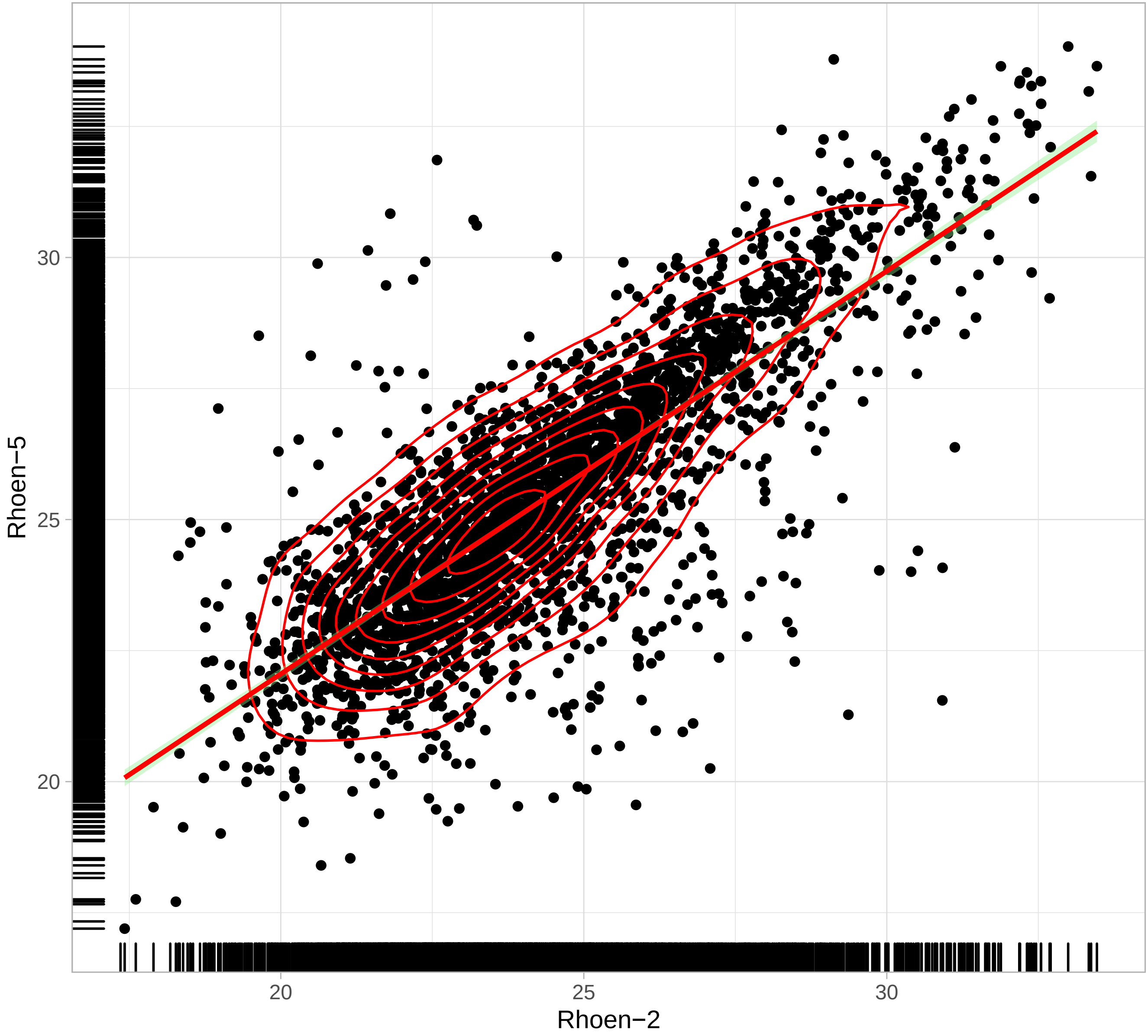

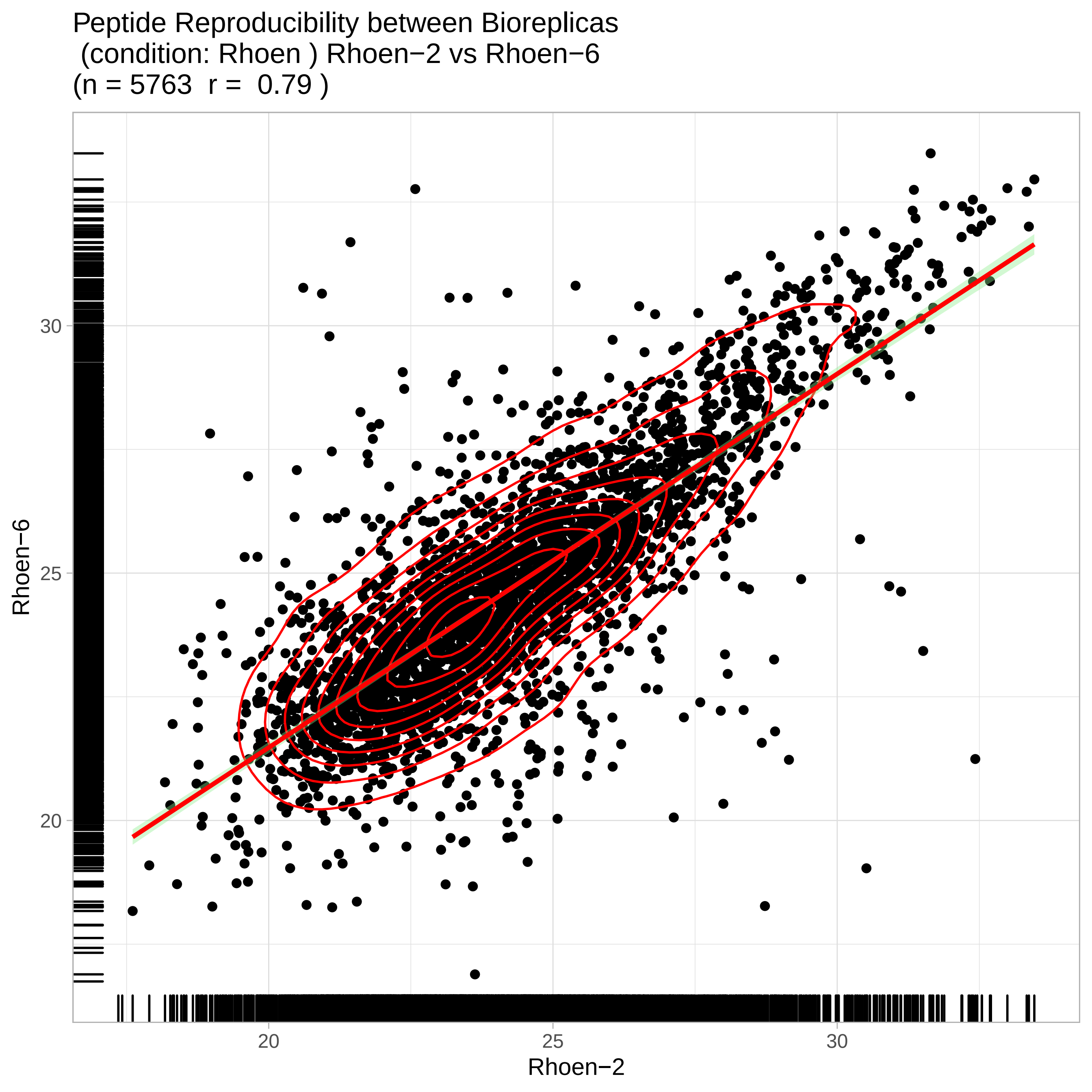

Peptide Reproducibility between Bioreplicas
(condition: Rhoen) Rhoen−2 vs Rhoen−6
(n = 5763 r = 0.79)
Rhoen−6
30
25
20
Rhoen−2
20
25
30

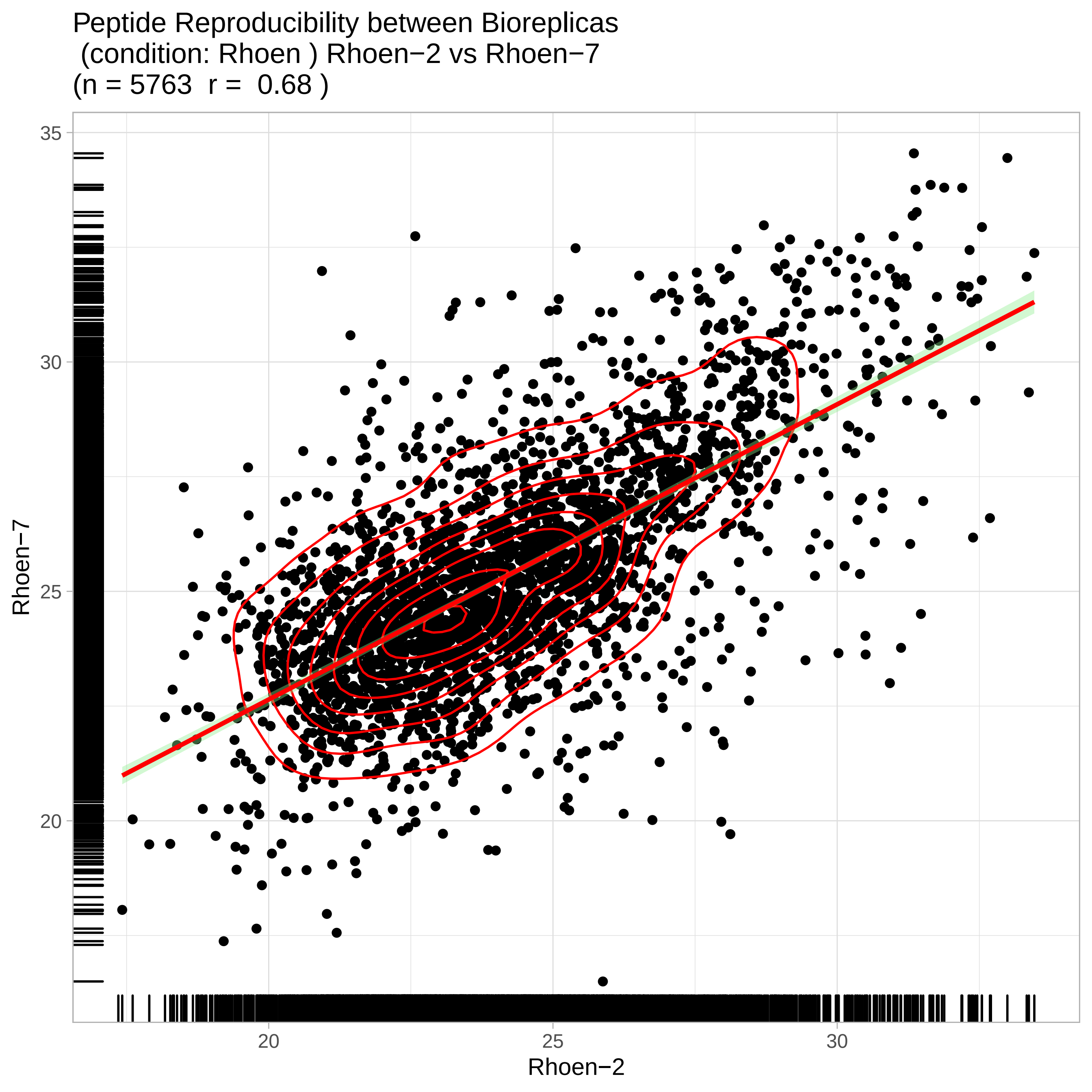

Peptide Reproducibility between Bioreplicas
(condition: Rhoen) Rhoen−2 vs Rhoen−7
(n = 5763 r = 0.68)
Rhoen−7
35
30
25
20
Rhoen−2
20
25
30

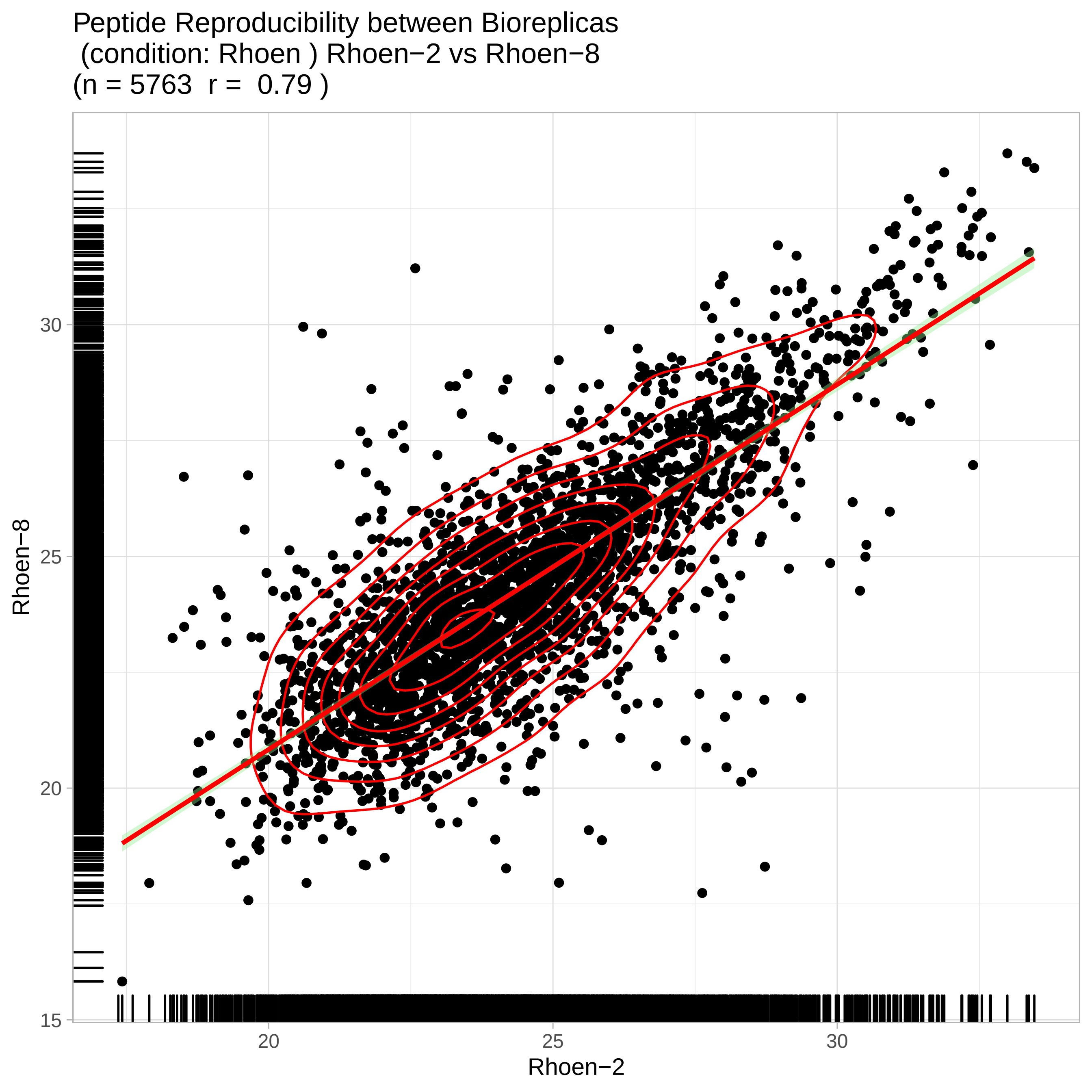
Peptide Reproducibility between Bioreplicas
(condition: Rhoen) Rhoen−2 vs Rhoen−8
(n = 5763 r = 0.79)
Rhoen−8
Rhoen−2
15
20
25
30
20
25
30

Peptide Reproducibility between Bioreplicas
(condition: Rhoen) Rhoen−3 vs Rhoen−4
(n = 5763 r = 0.79)

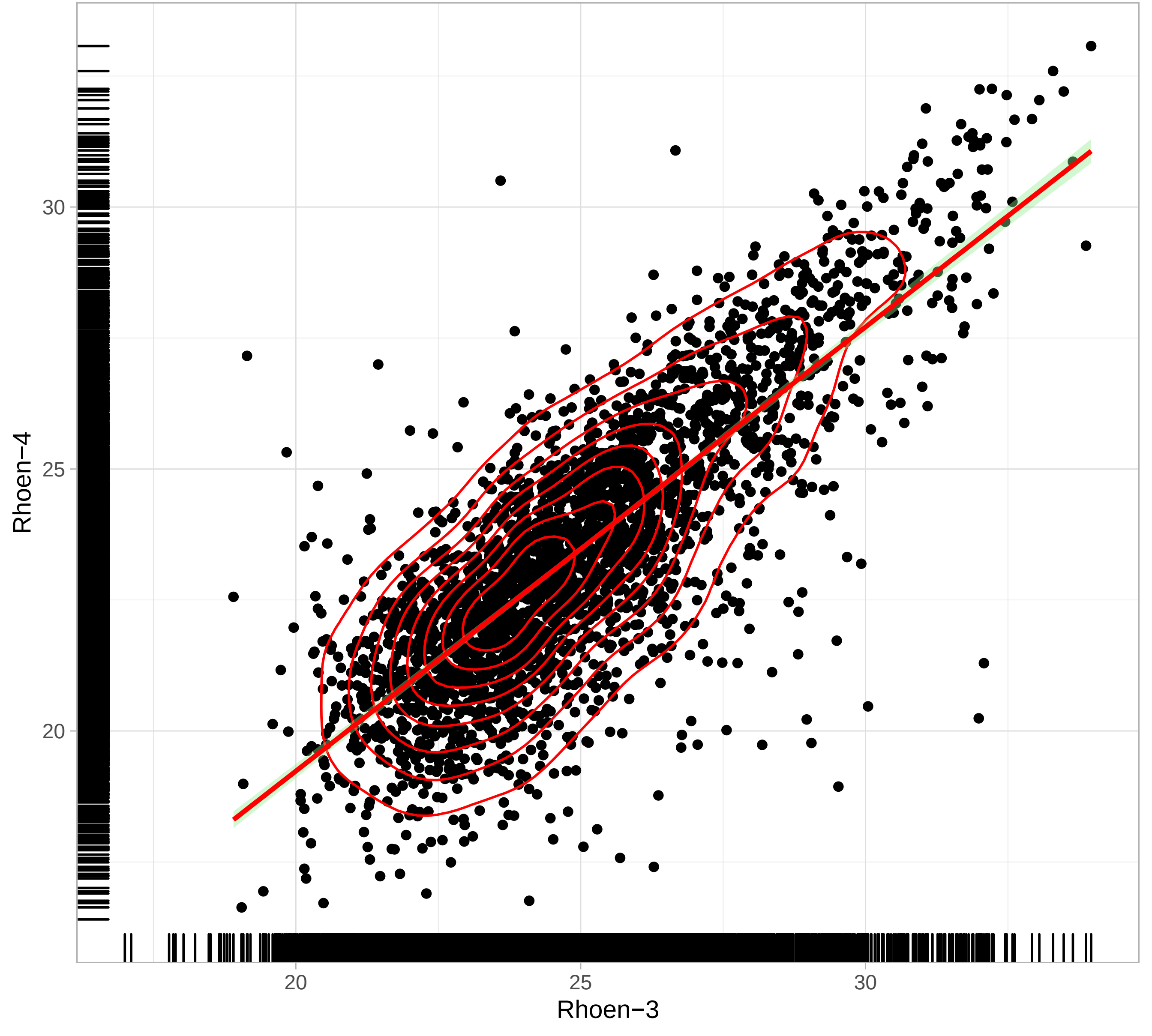

Peptide Reproducibility between Bioreplicas
 (condition: Rhoen) Rhoen−3 vs Rhoen−5
(n = 5763 r = 0.8)

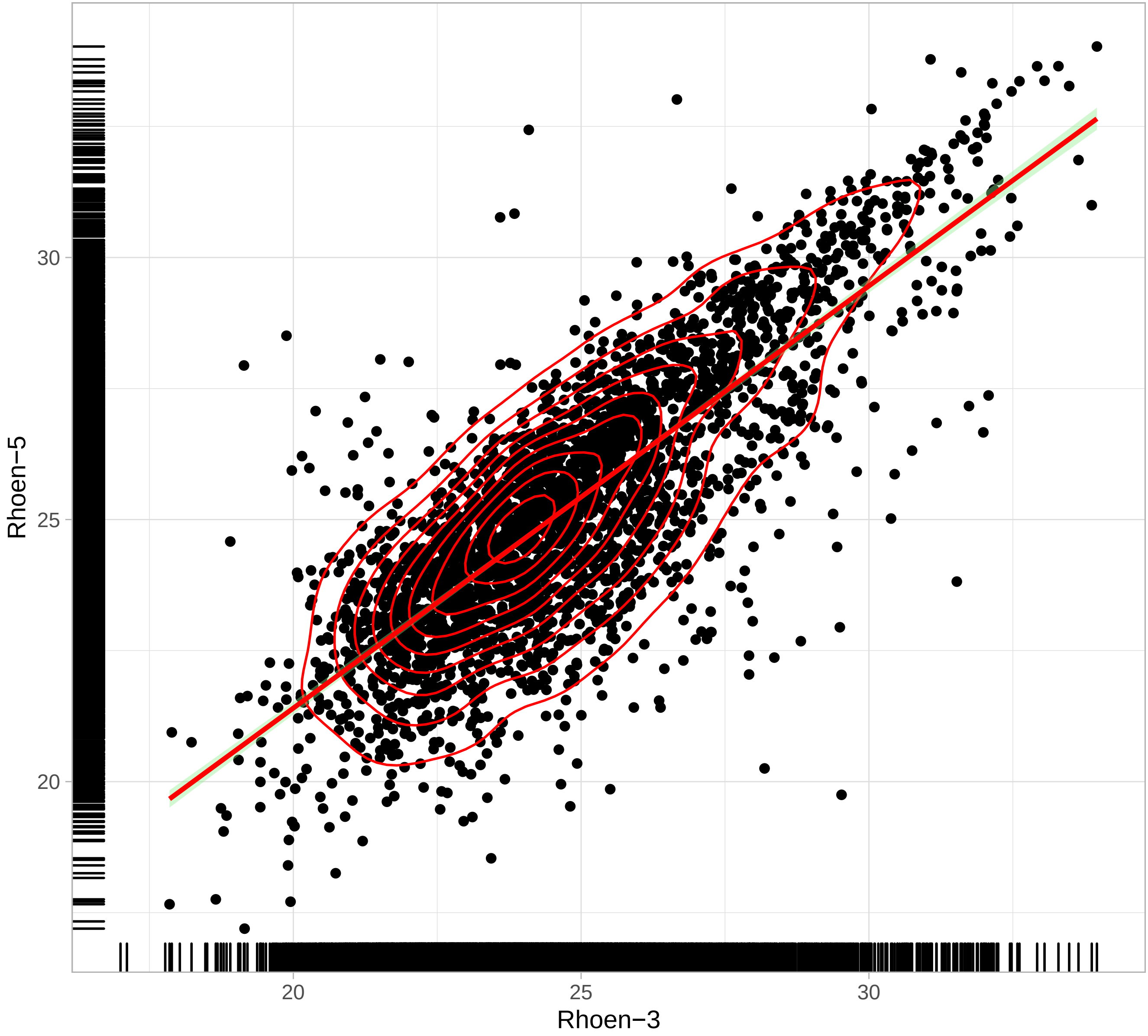

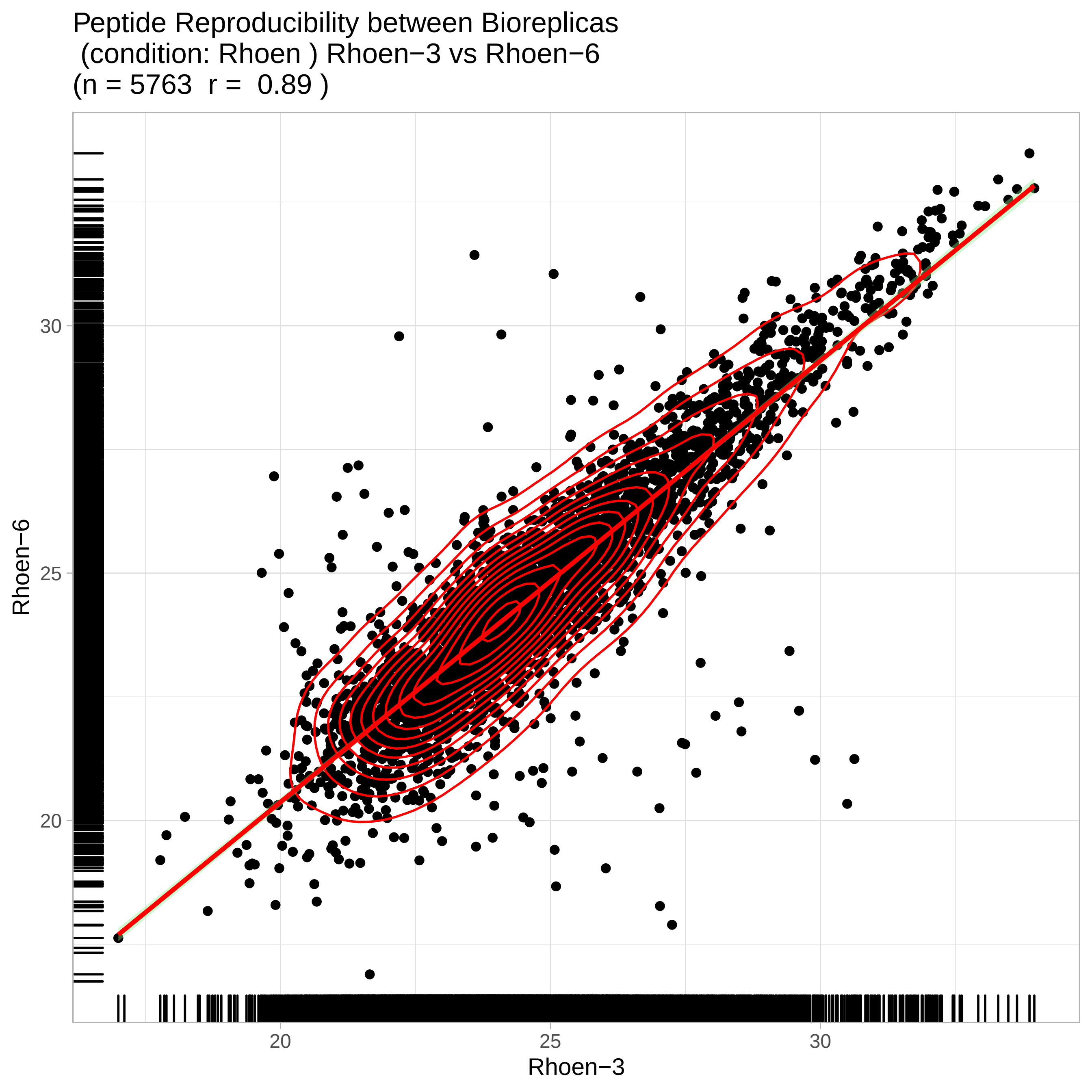
Peptide Reproducibility between Bioreplicas
(condition: Rhoen) Rhoen−3 vs Rhoen−6
(n = 5763 r = 0.89)
Rhoen−6
30
25
20
20
25
30
Rhoen−3

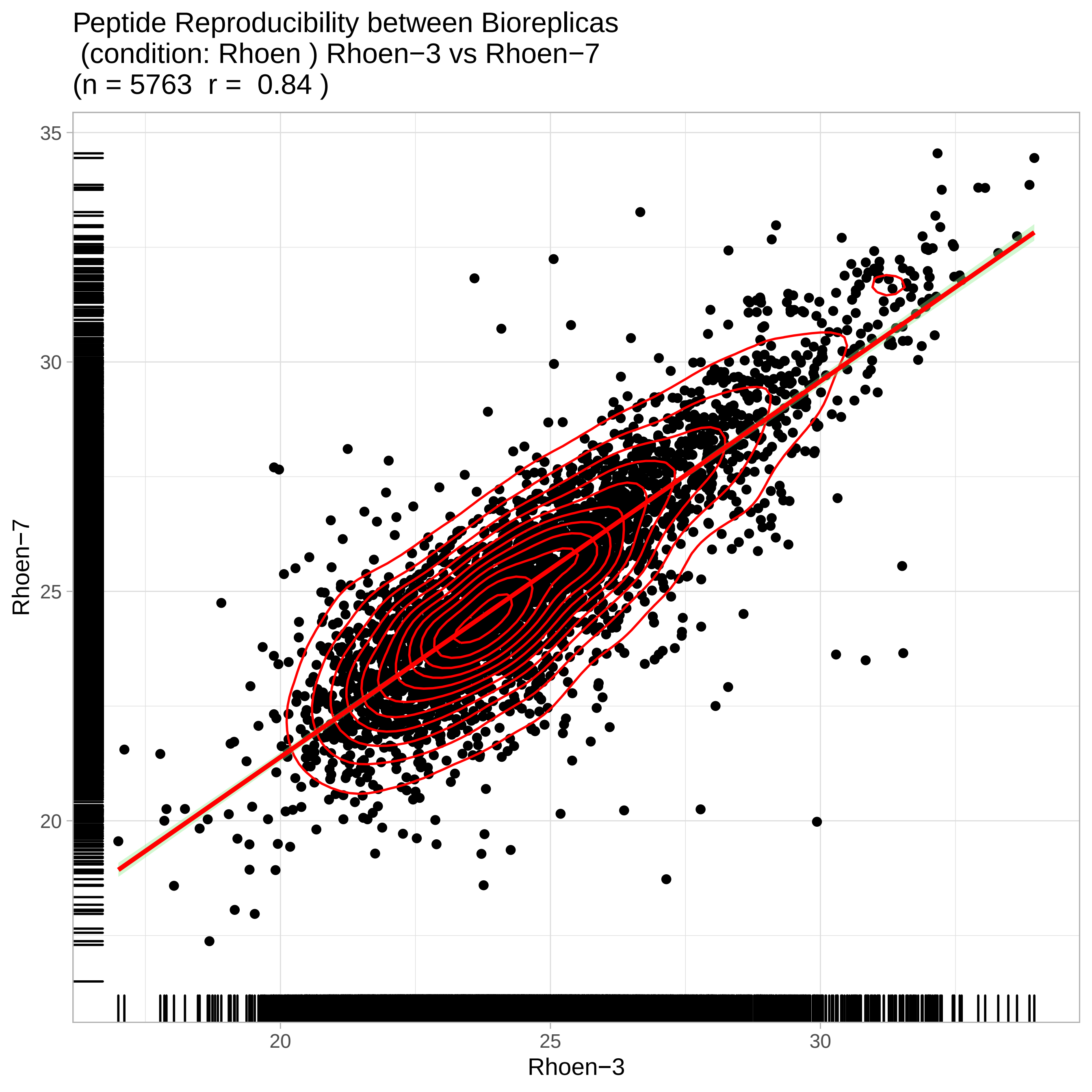
Peptide Reproducibility between Bioreplicas
(condition: Rhoen) Rhoen−3 vs Rhoen−7
(n = 5763 r = 0.84)
Rhoen−7
35
30
25
20
20
25
30
Rhoen−3

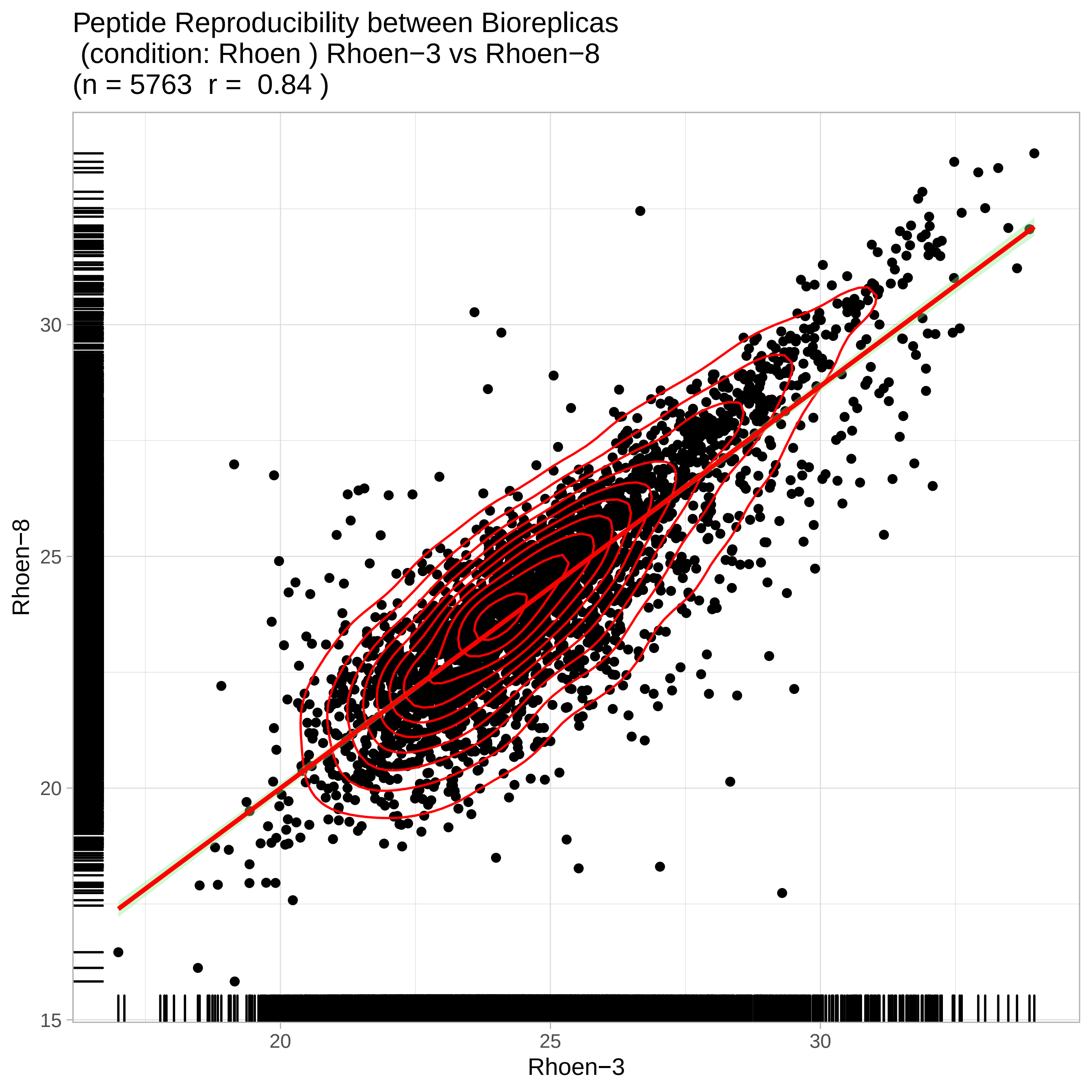

Peptide Reproducibility between Bioreplicas
(condition: Rhoen) Rhoen−3 vs Rhoen−8
(n = 5763 r = 0.84)
Rhoen−8
30
25
20
15
20
25
30
Rhoen−3

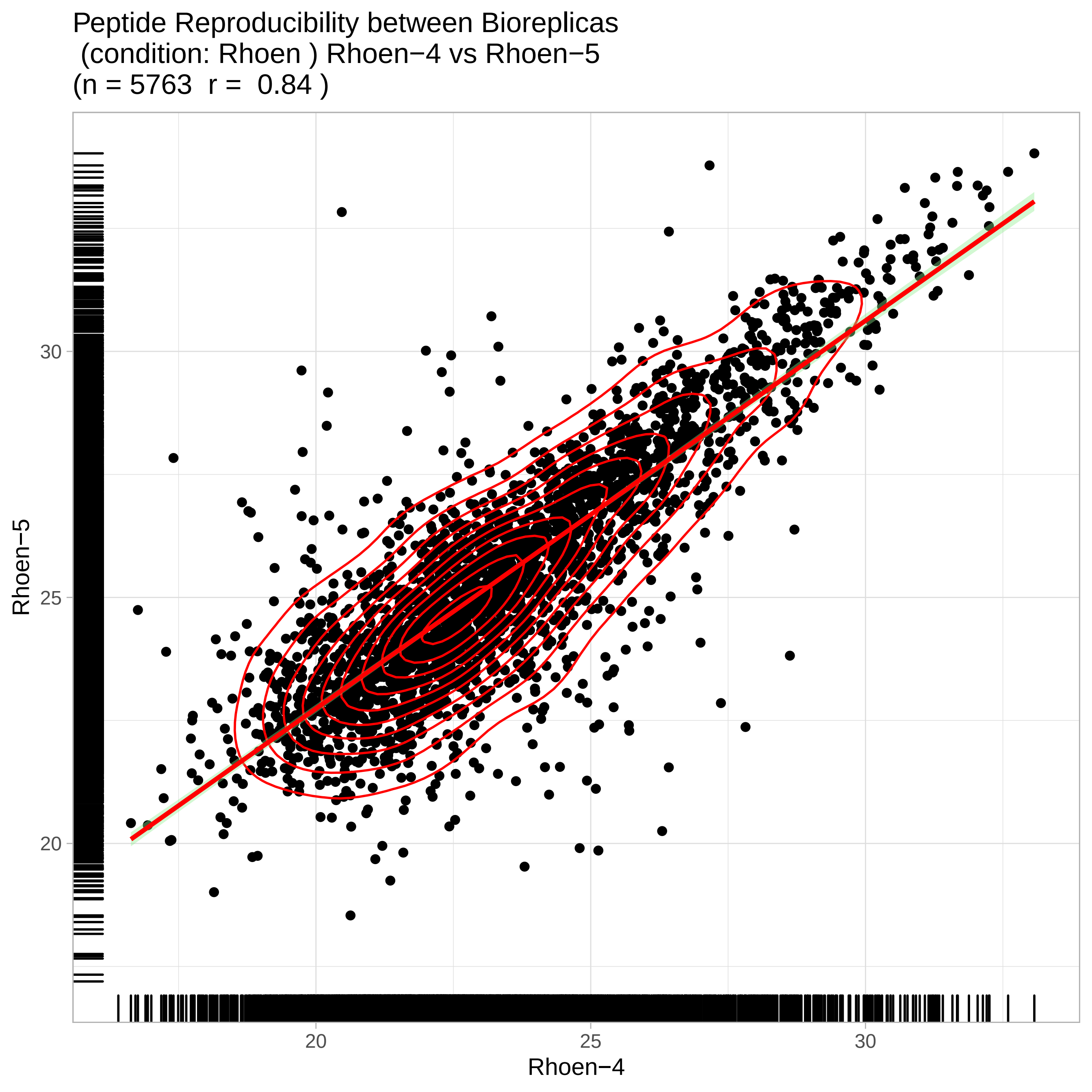
Peptide Reproducibility between Bioreplicas
(condition: Rhoen) Rhoen−4 vs Rhoen−5
(n = 5763 r = 0.84)
Rhoen−5
30
25
20
20
25
30
Rhoen−4

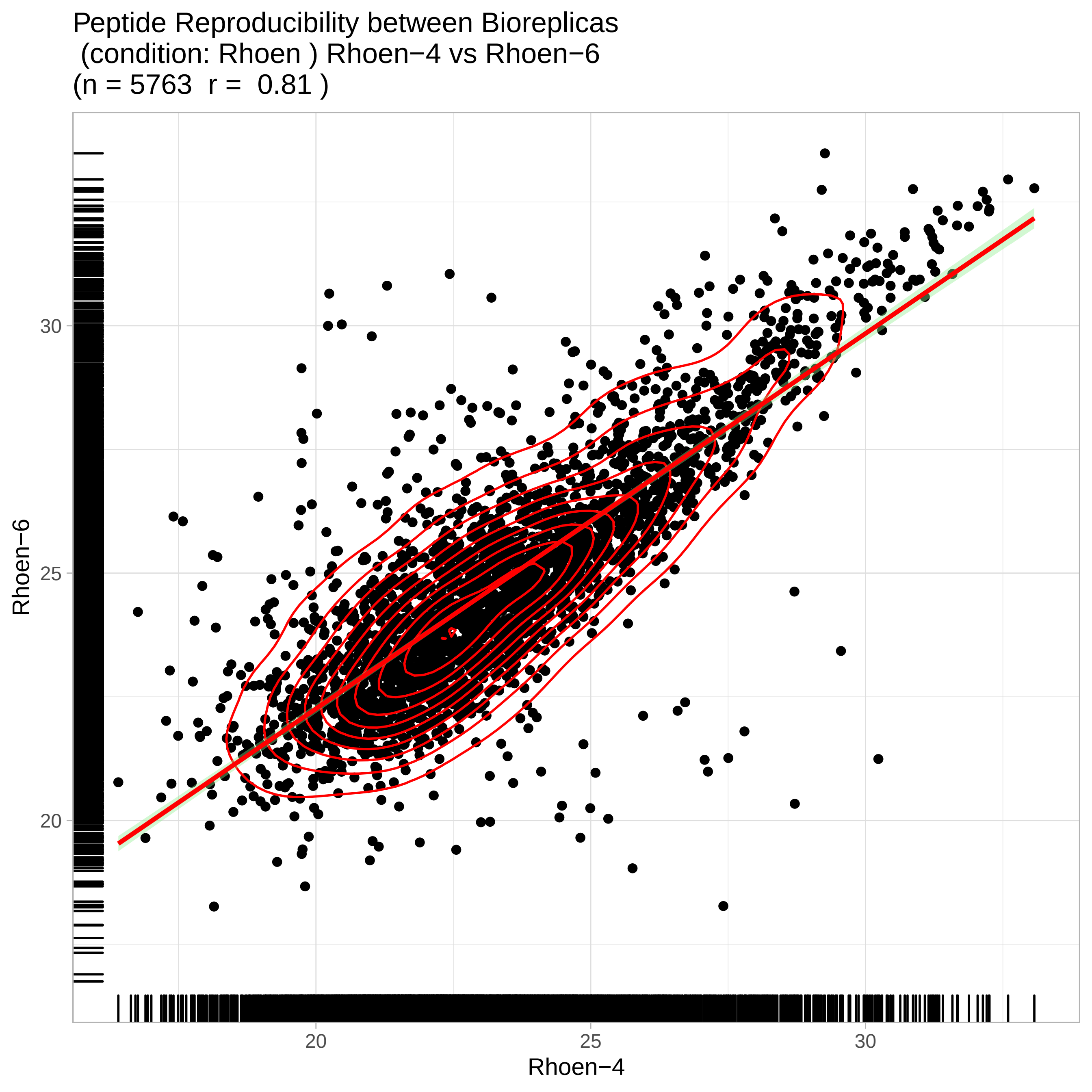
Peptide Reproducibility between Bioreplicas
(condition: Rhoen) Rhoen−4 vs Rhoen−6
(n = 5763 r = 0.81)
Rhoen−6
30
25
20
20
25
30
Rhoen−4

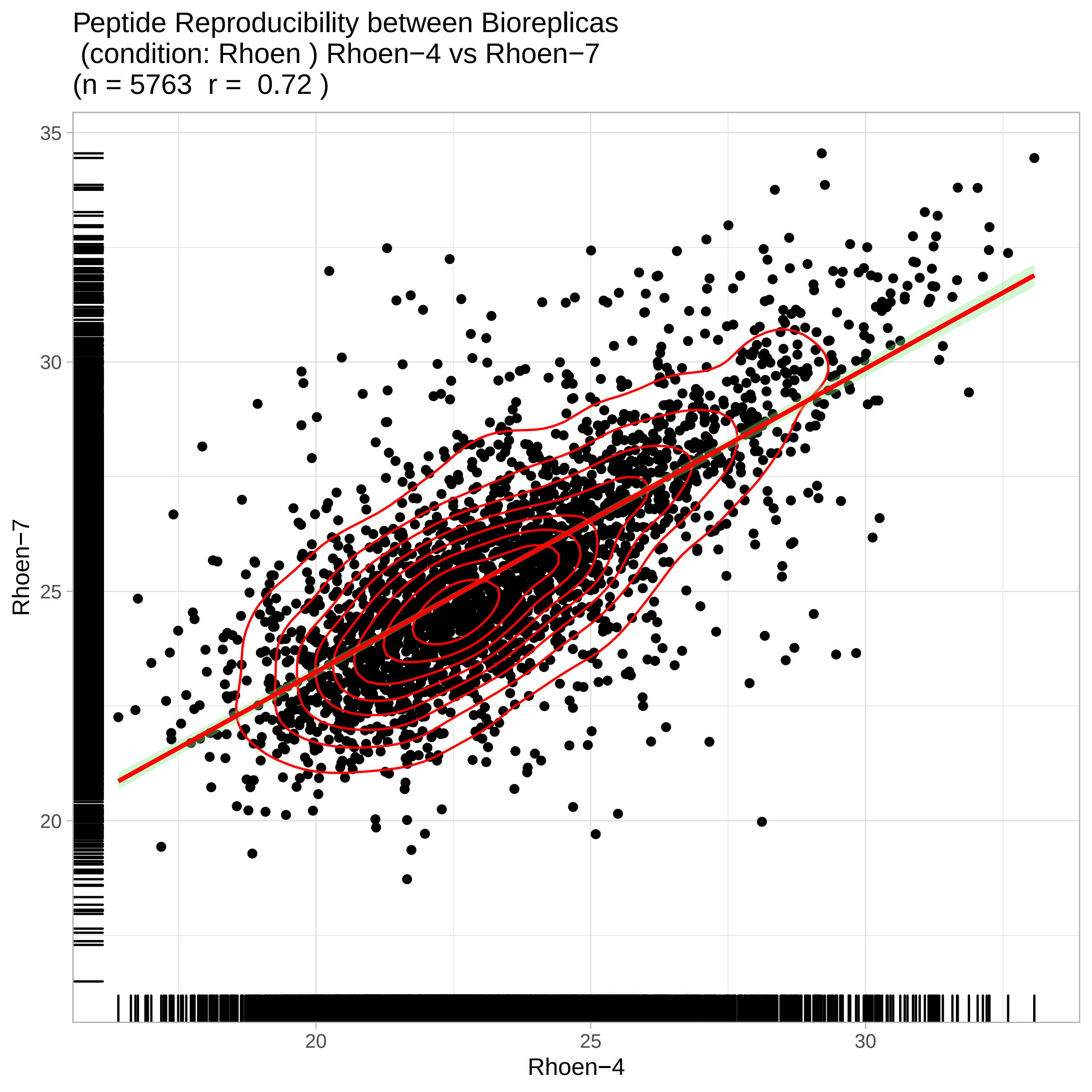

Peptide Reproducibility between Bioreplicas
(condition: Rhoen) Rhoen−4 vs Rhoen−7
(n = 5763 r = 0.72)
Rhoen−7
35
30
25
20
20
25
30
Rhoen−4

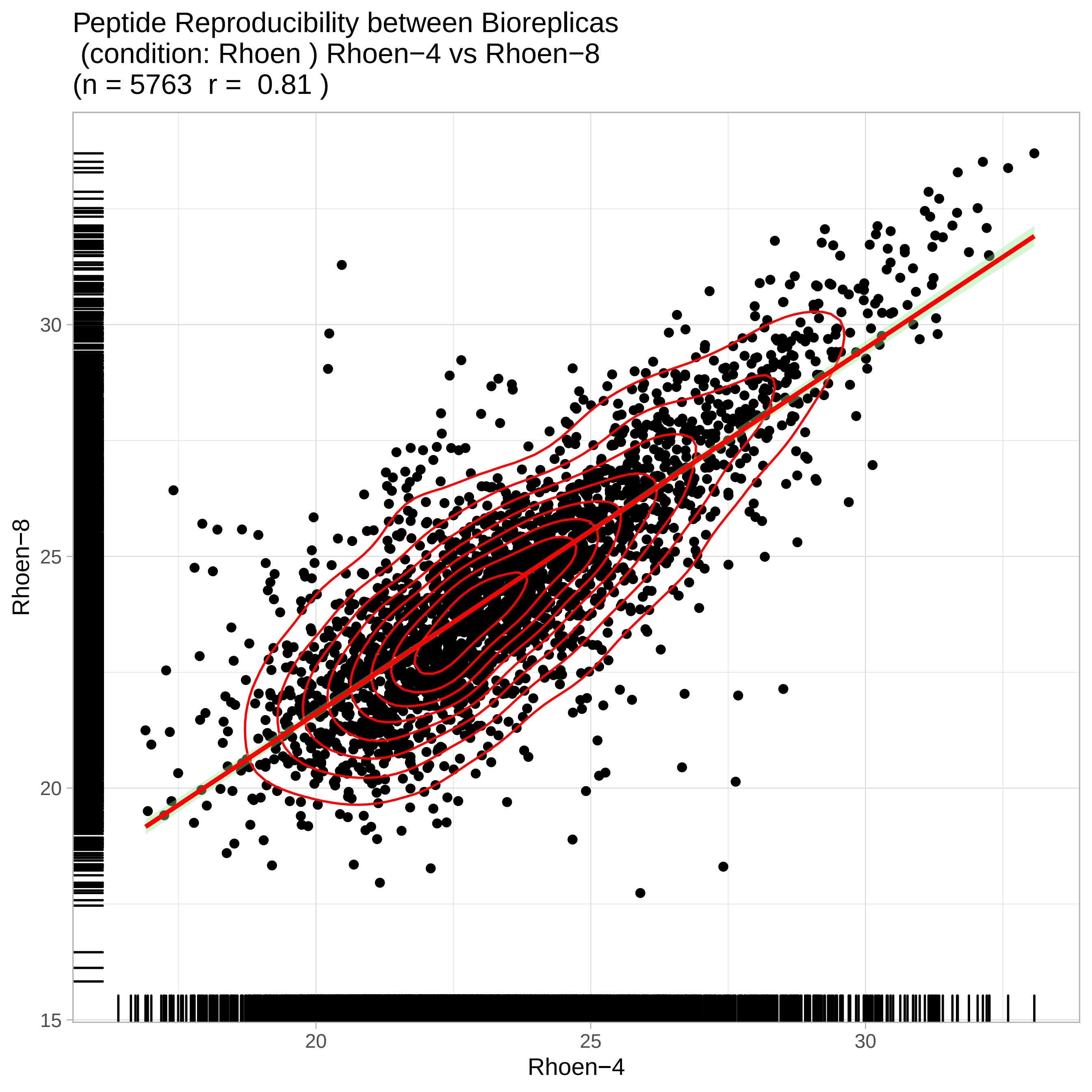
Peptide Reproducibility between Bioreplicas
(condition: Rhoen) Rhoen−4 vs Rhoen−8
(n = 5763 r = 0.81)
Rhoen−8
30
25
20
15
20
25
30
Rhoen−4

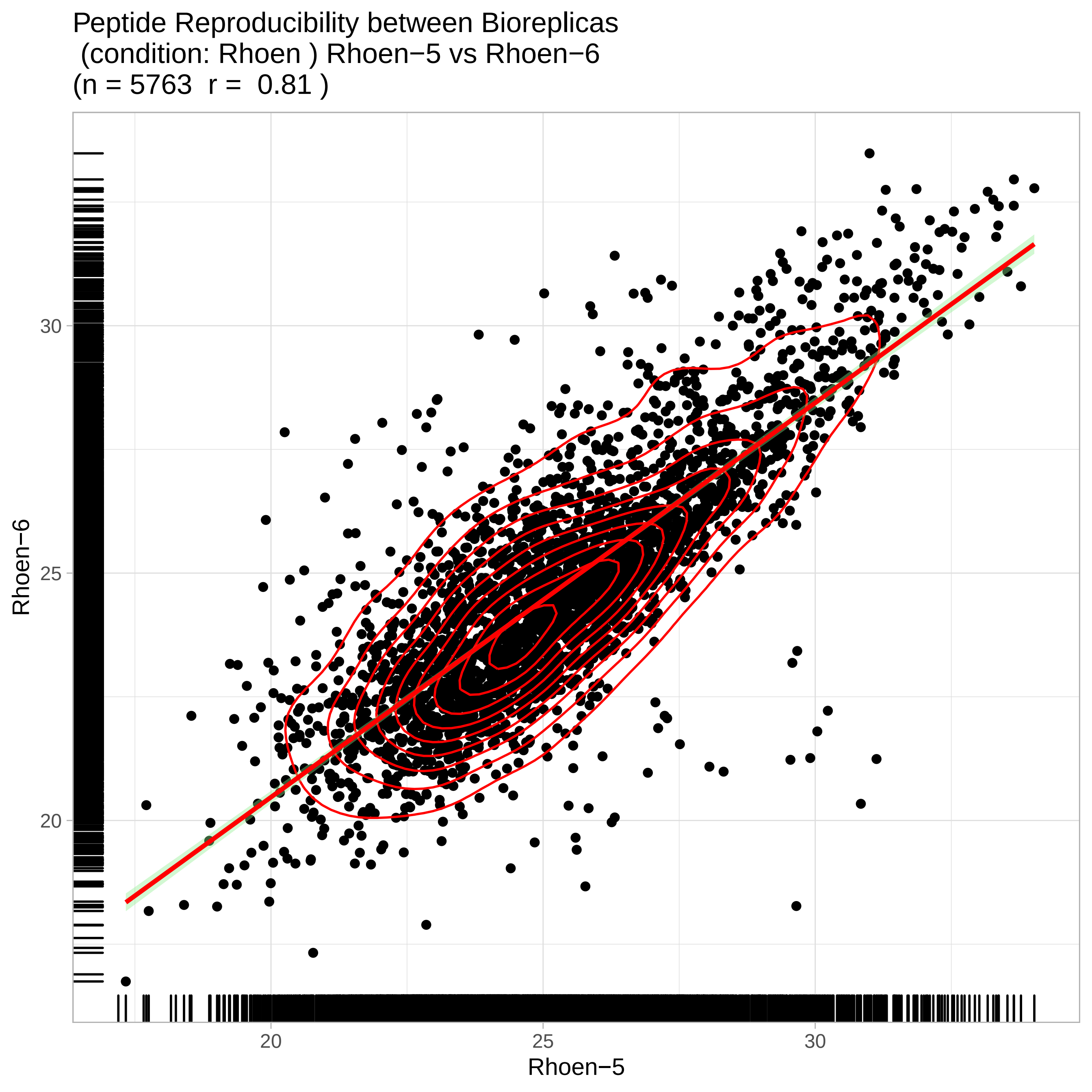

Peptide Reproducibility between Bioreplicas
(condition: Rhoen) Rhoen−5 vs Rhoen−6
(n = 5763 r = 0.81)
Rhoen−6
30
25
20
Rhoen−5
20
25
30

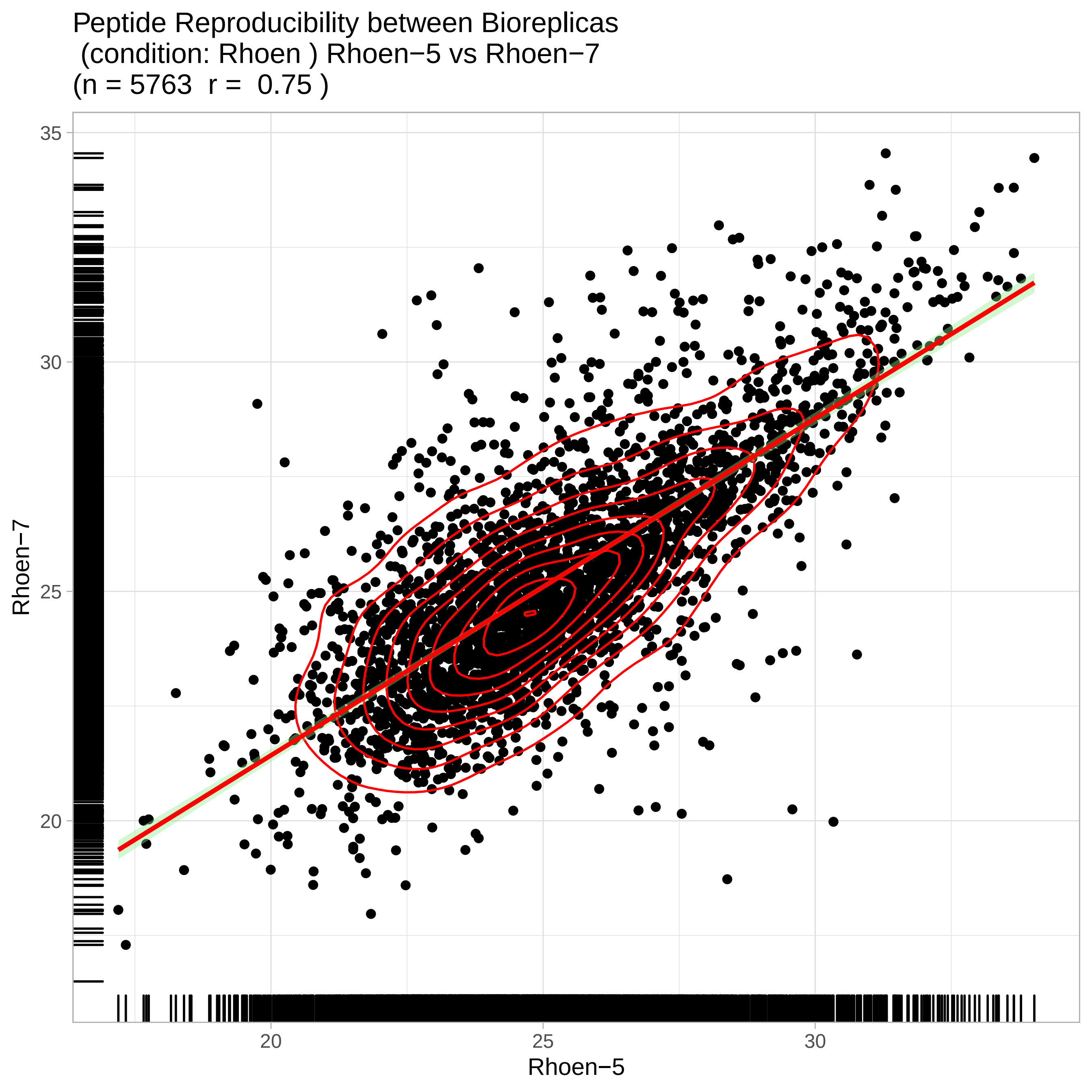
Peptide Reproducibility between Bioreplicas
(condition: Rhoen) Rhoen−5 vs Rhoen−7
(n = 5763 r = 0.75)
Rhoen−7
35
30
25
20
20
25
30
Rhoen−5

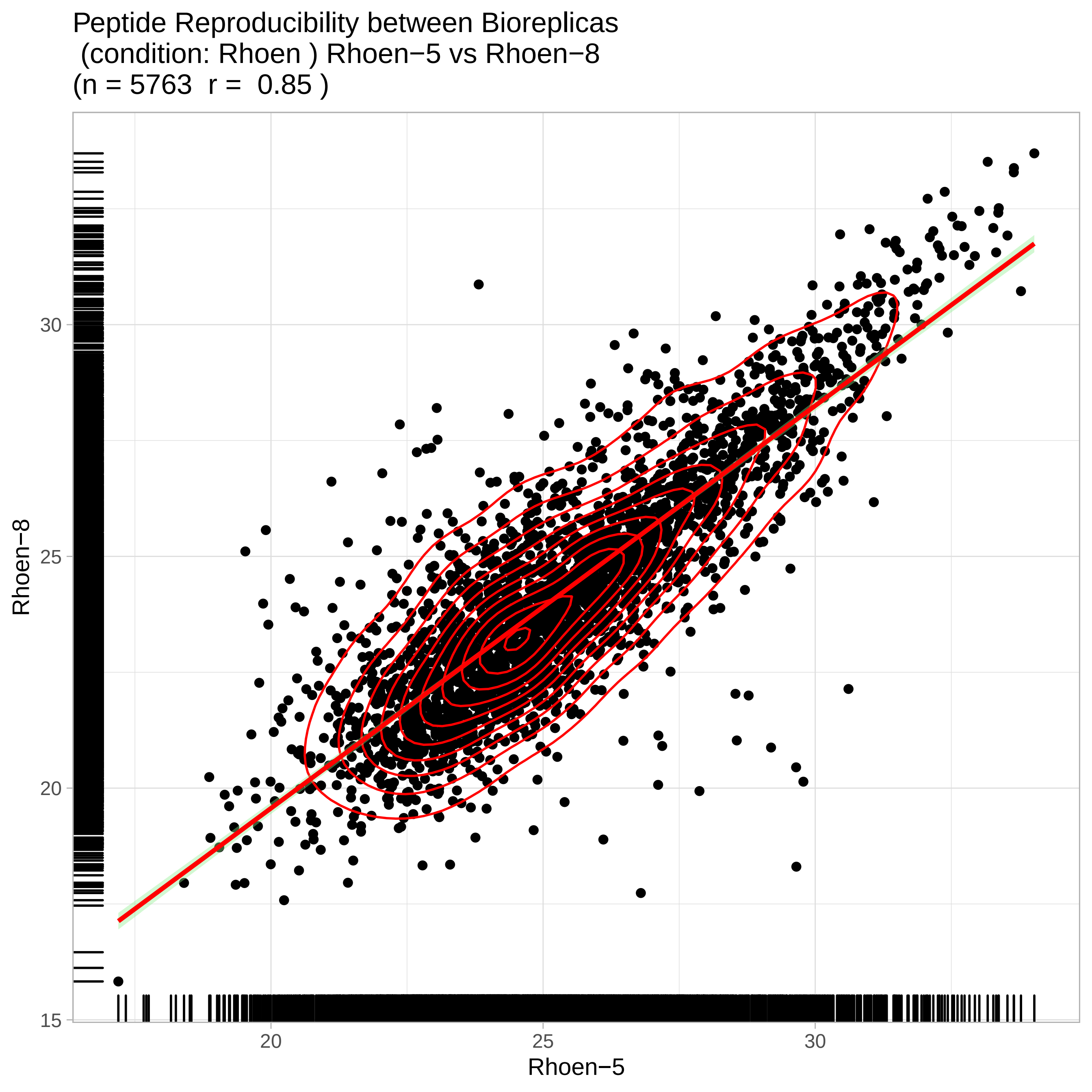
Peptide Reproducibility between Bioreplicas
(condition: Rhoen) Rhoen−5 vs Rhoen−8
(n = 5763 r = 0.85)
Rhoen−8
Rhoen−5
30
25
20
15
20
25
30

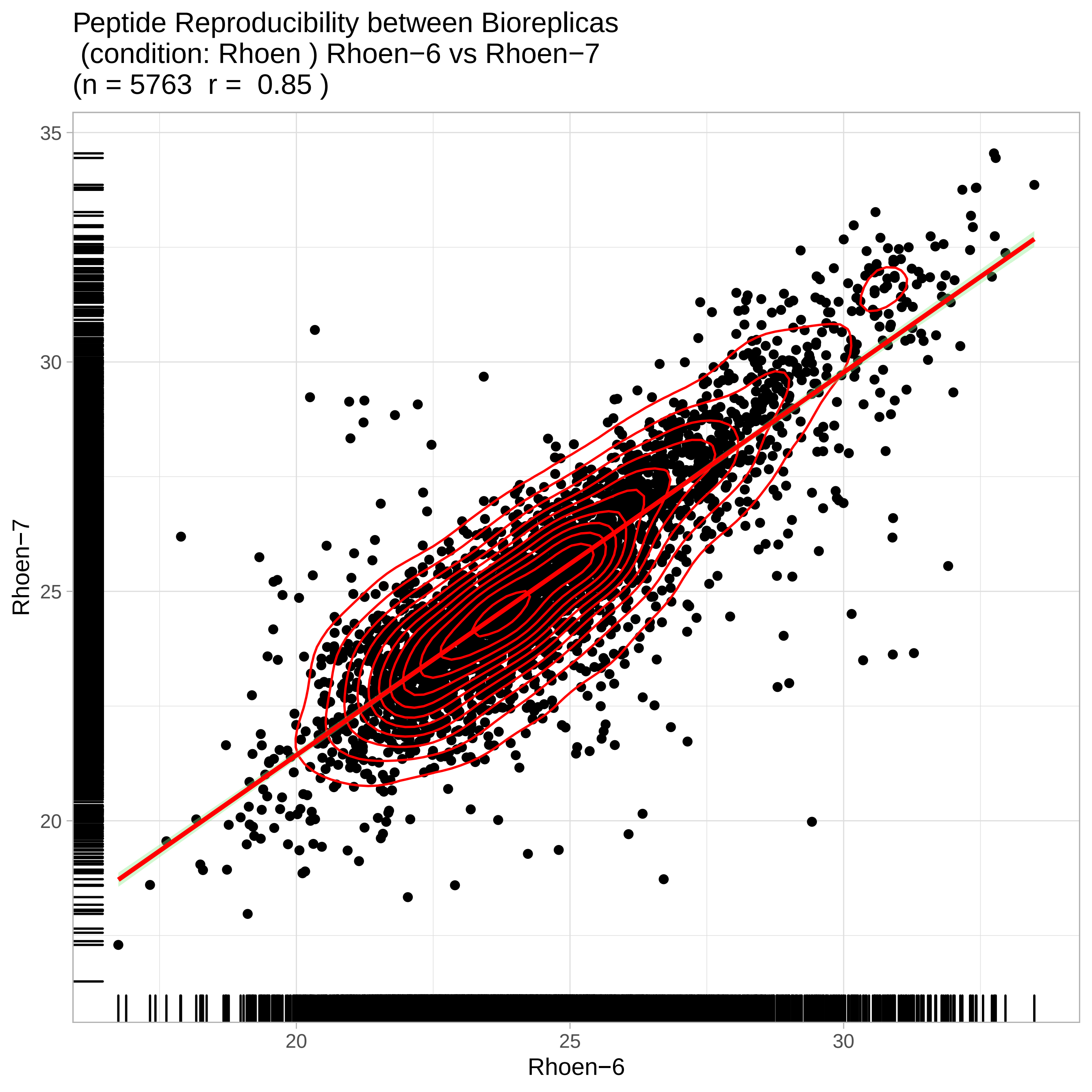
Peptide Reproducibility between Bioreplicas
(condition: Rhoen) Rhoen−6 vs Rhoen−7
(n = 5763 r = 0.85)
Rhoen−7
35
30
25
20
Rhoen−6
20
25
30

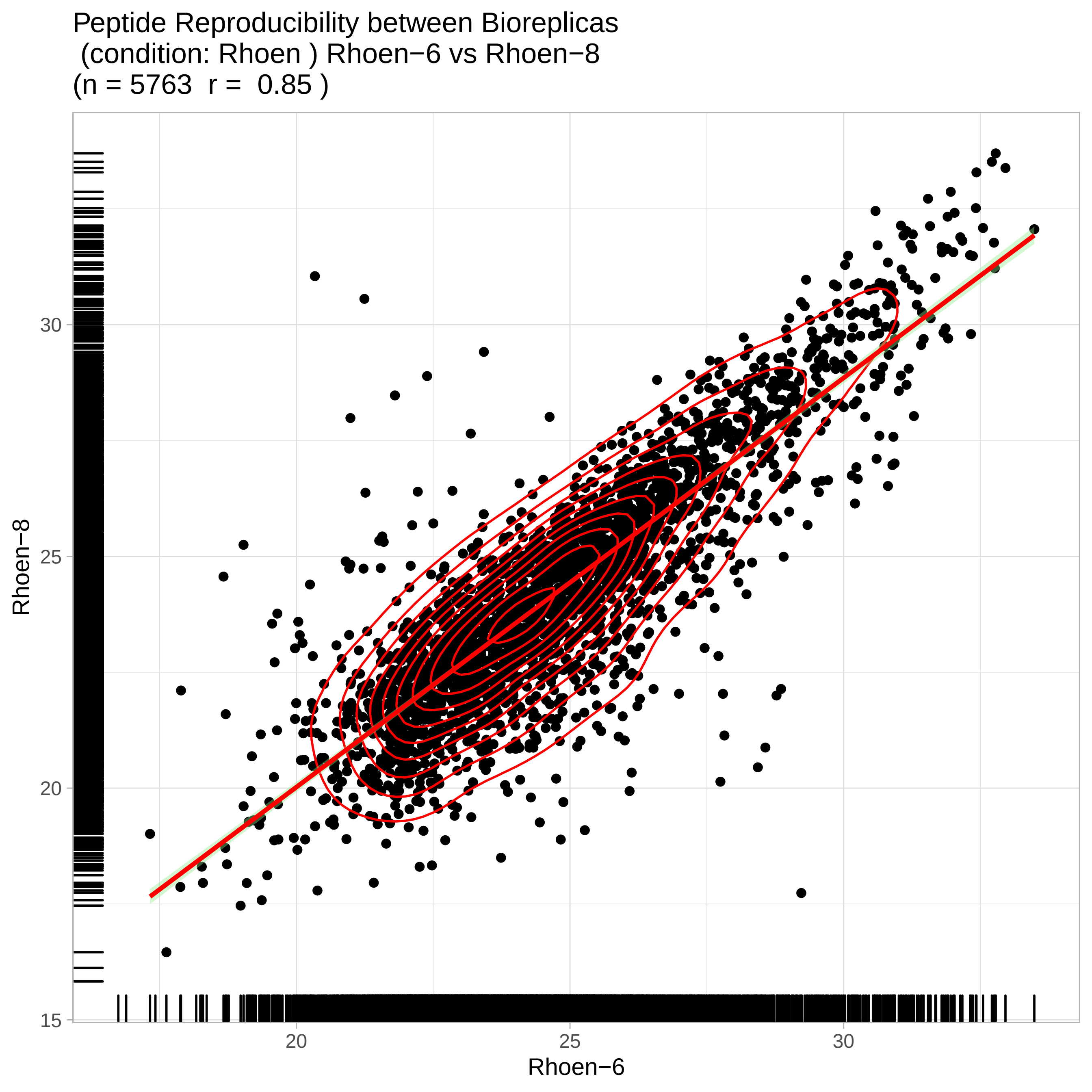

Peptide Reproducibility between Bioreplicas
(condition: Rhoen) Rhoen−6 vs Rhoen−8
(n = 5763 r = 0.85)
Rhoen−8
Rhoen−6
30
25
20
15
20
25
30

Peptide Reproducibility between Bioreplicas
(condition: Rhoen) Rhoen−7 vs Rhoen−8
(n = 5763 r = 0.78)

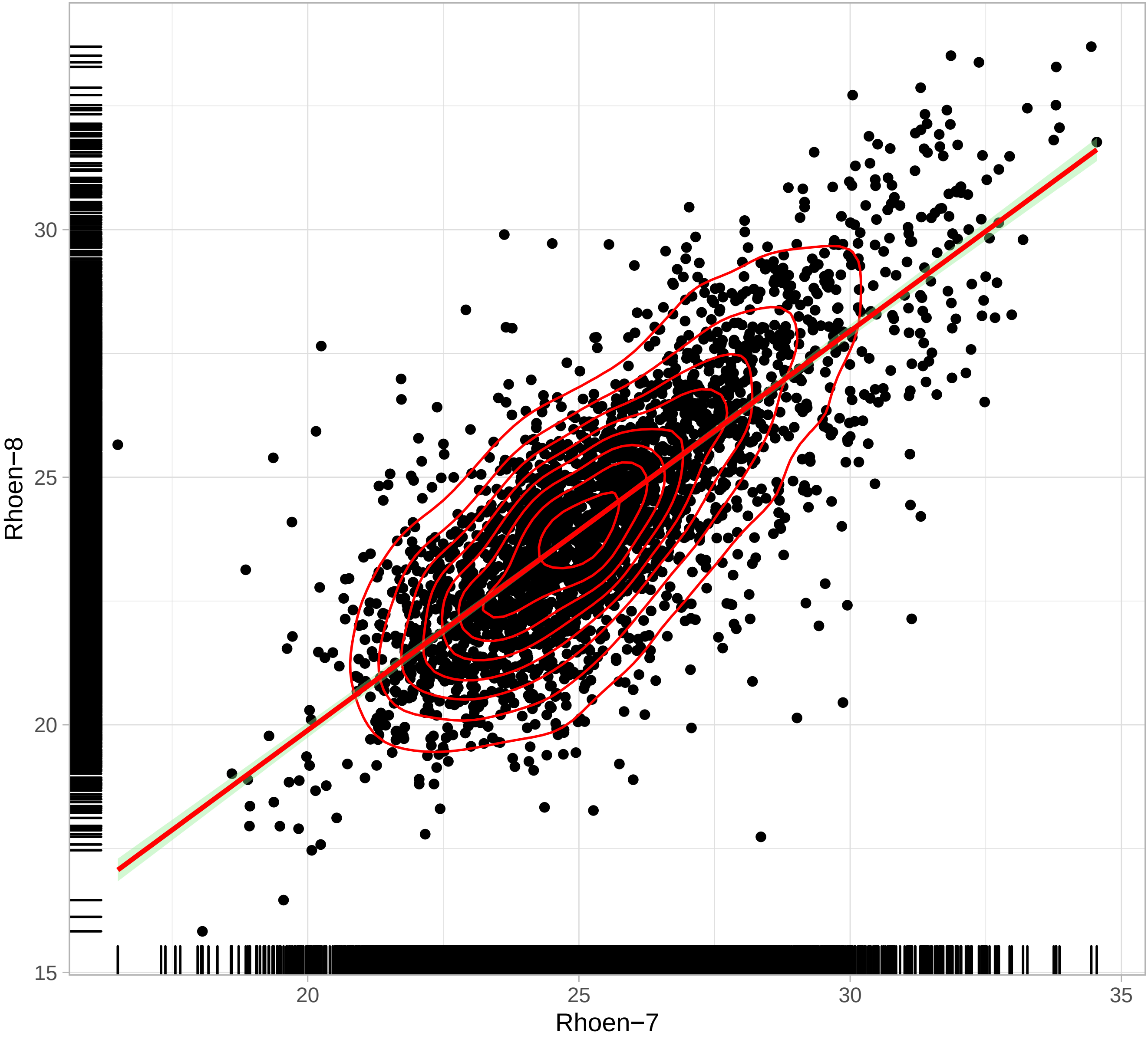

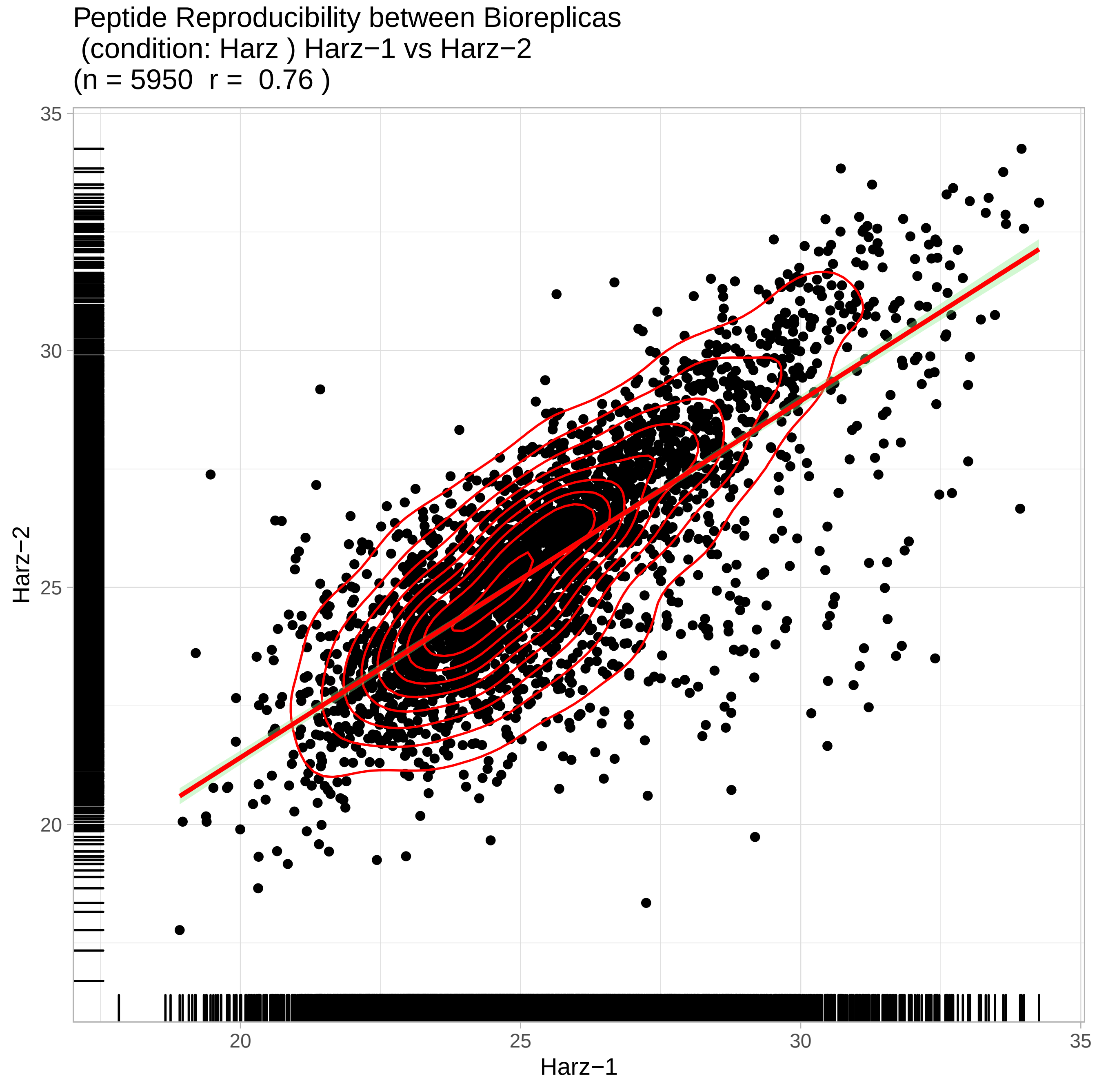

Peptide Reproducibility between Bioreplicas
(condition: Harz) Harz−1 vs Harz−2
(n = 5950 r = 0.76)
Harz−2
Harz−1
35
30
25
20
20
25
30
35

Peptide Reproducibility between Bioreplicas
(condition: Harz) Harz−1 vs Harz−3
(n = 5950 r = 0.8)

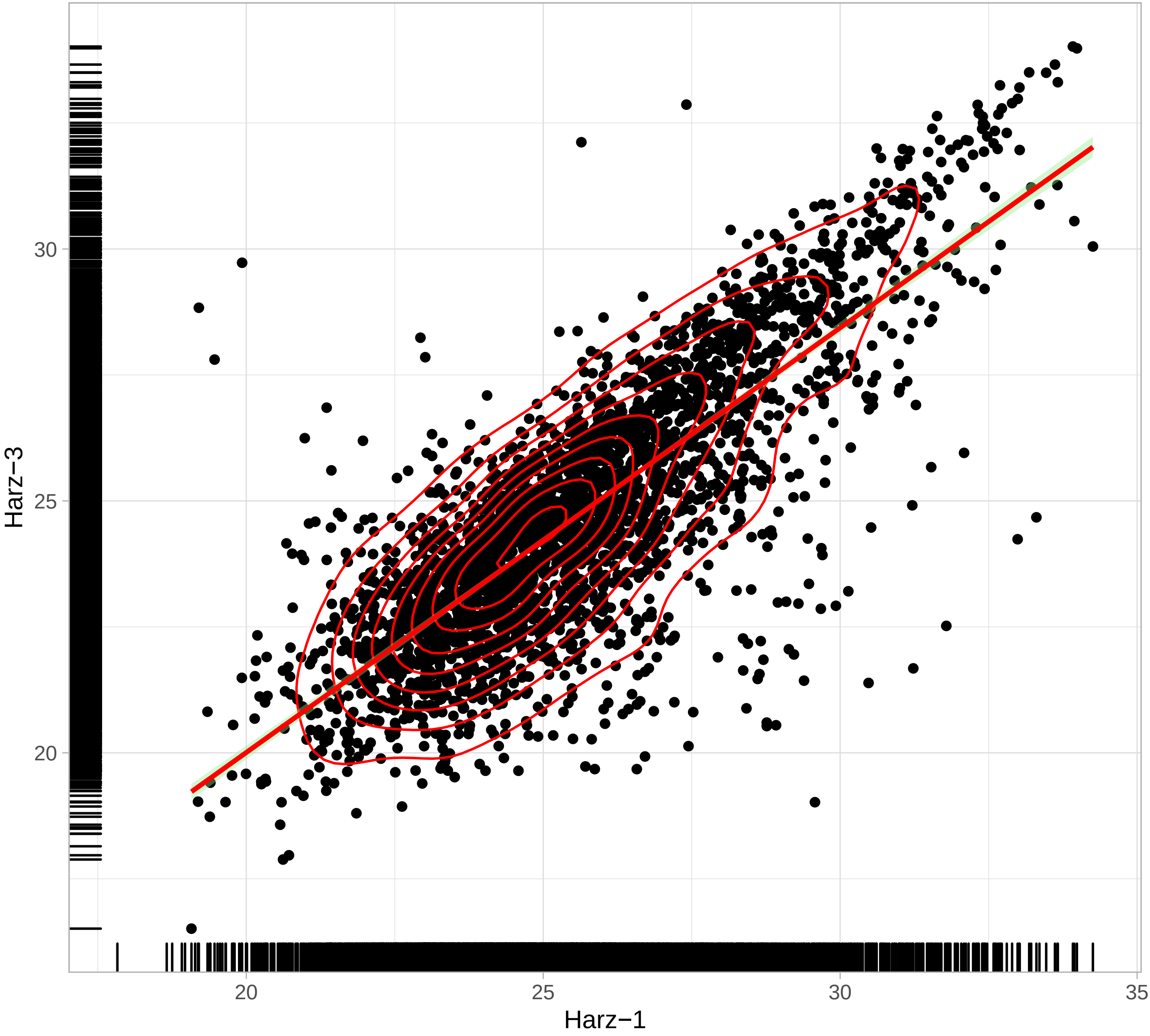

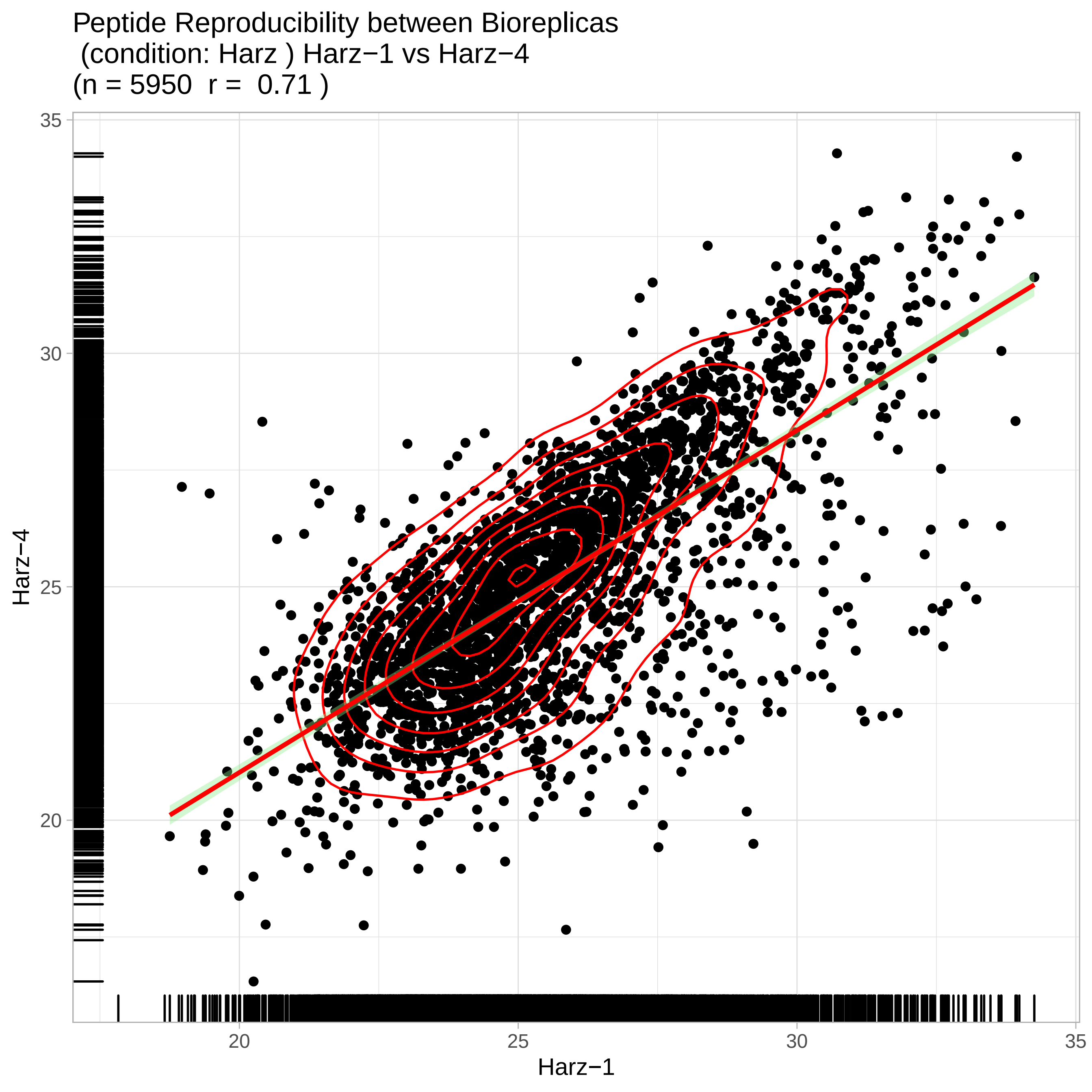
Peptide Reproducibility between Bioreplicas
(condition: Harz) Harz−1 vs Harz−4
(n = 5950 r = 0.71)
Harz−4
35
30
25
20
20
25
30
35
Harz−1

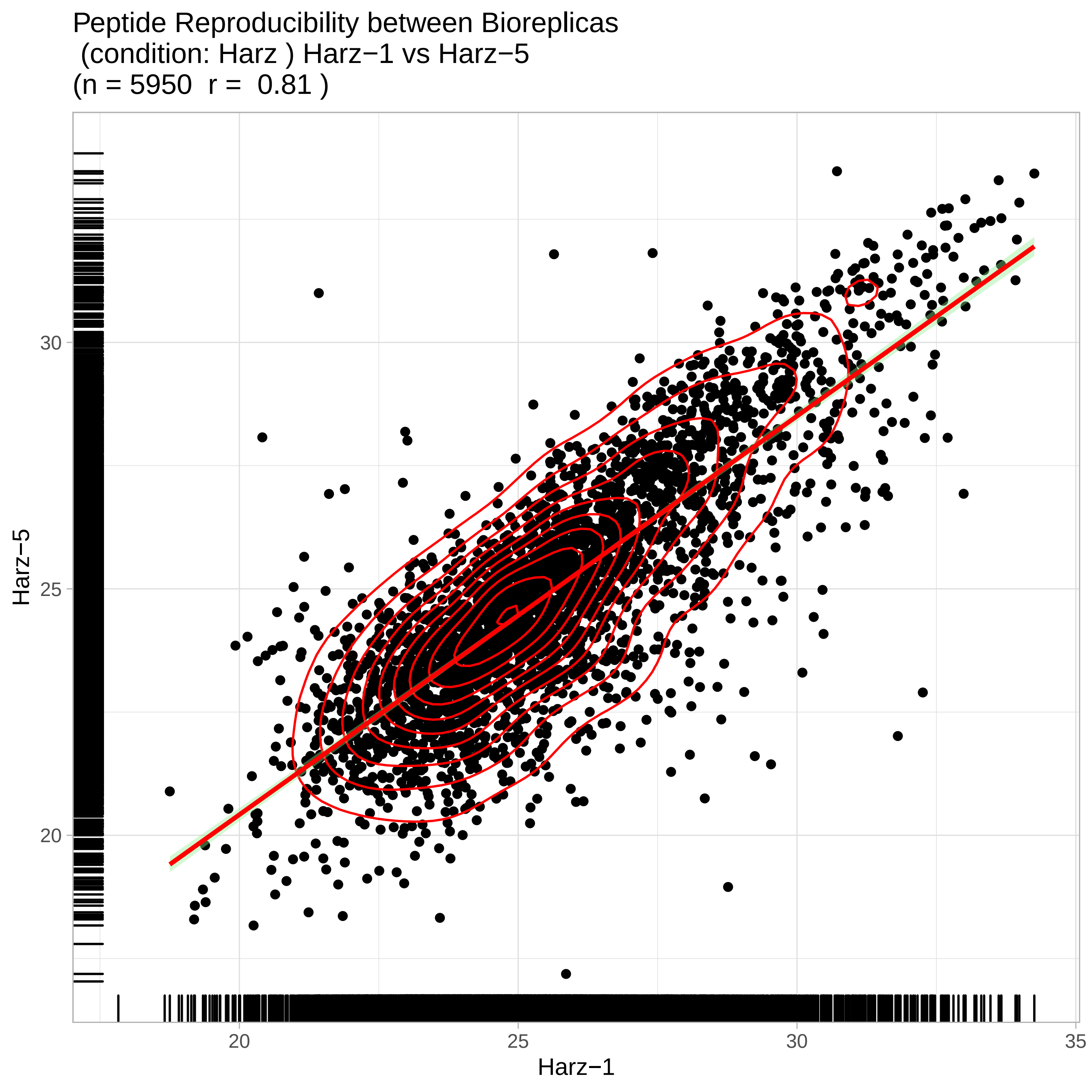
Peptide Reproducibility between Bioreplicas
(condition: Harz) Harz−1 vs Harz−5
(n = 5950 r = 0.81)
Harz−5
30
25
20
20
25
30
35
Harz−1

Peptide Reproducibility between Bioreplicas
(condition: Harz) Harz−1 vs Harz−6
(n = 5950 r = 0.83)

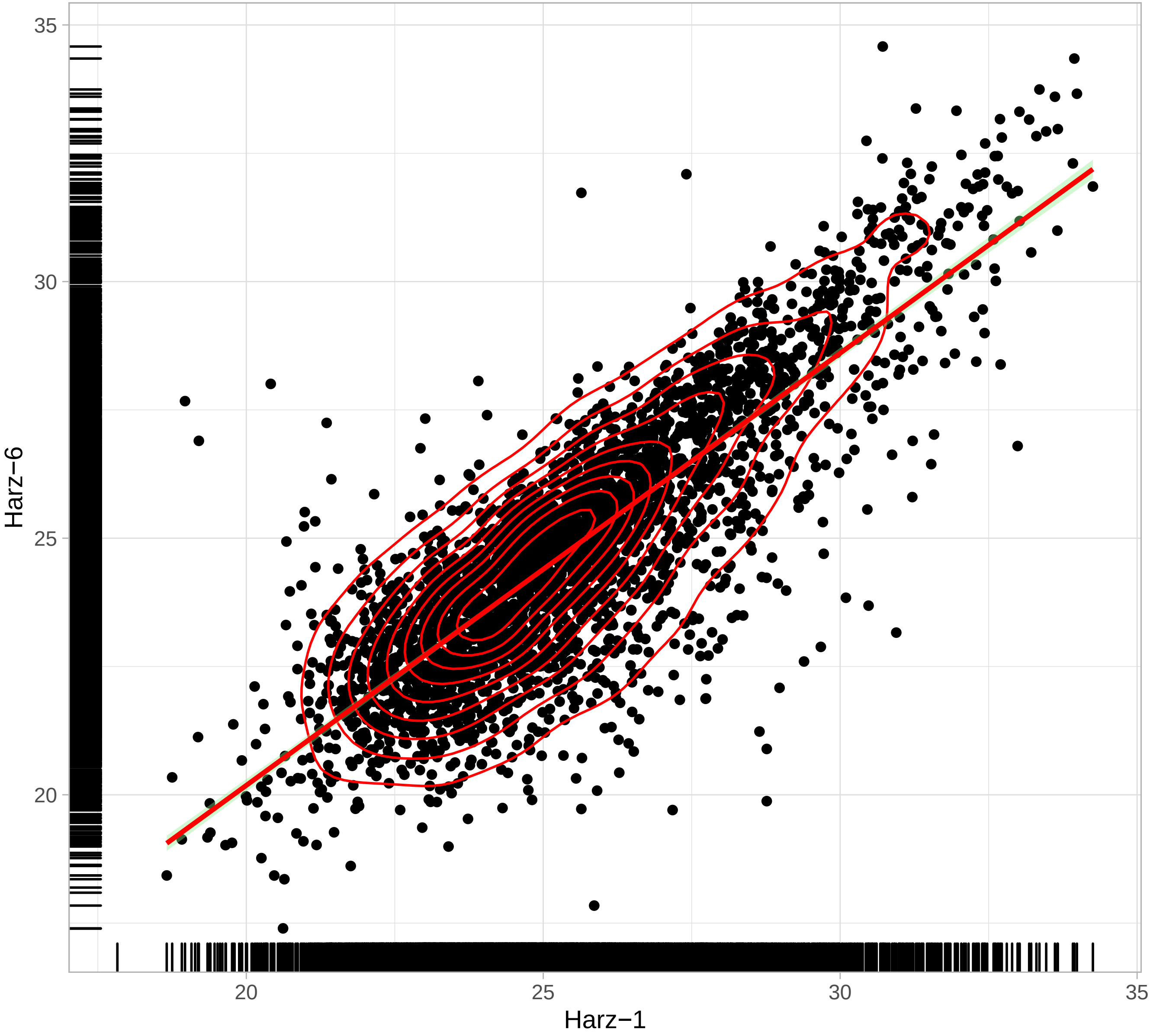

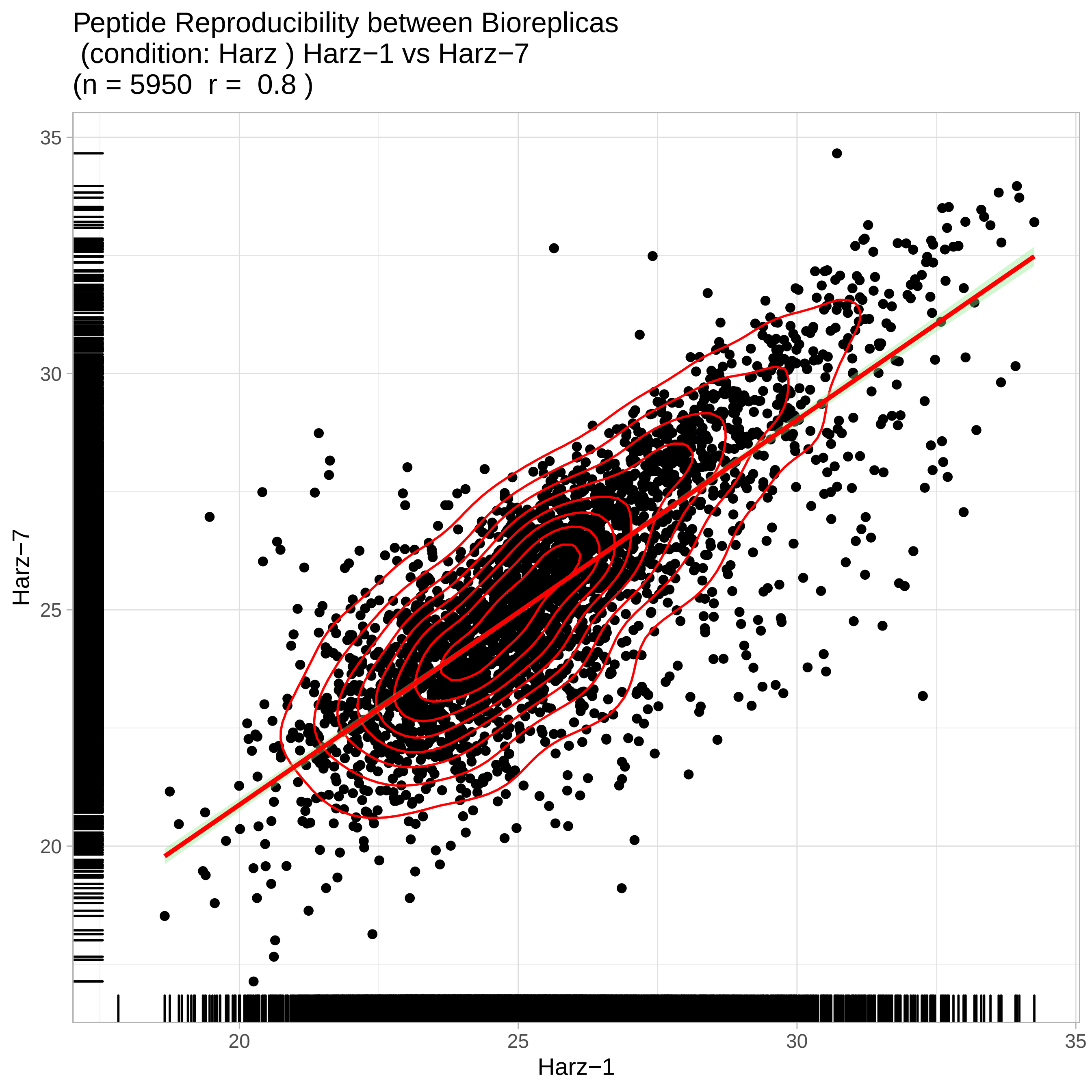

Peptide Reproducibility between Bioreplicas
(condition: Harz) Harz−1 vs Harz−7
(n = 5950 r = 0.8)
35
30
25
20
Harz−7
20
25
30
35
Harz−1

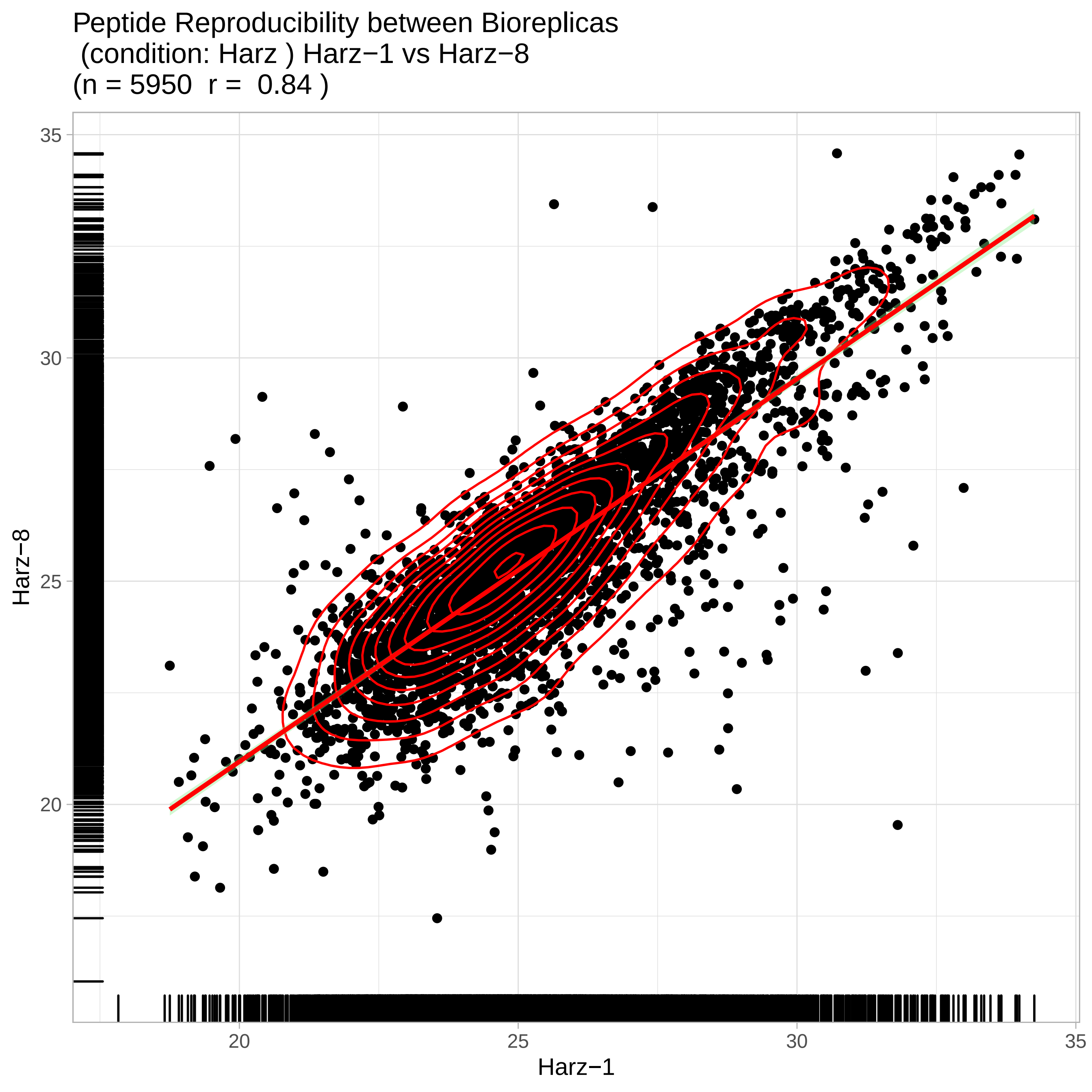
Peptide Reproducibility between Bioreplicas
(condition: Harz) Harz−1 vs Harz−8
(n = 5950 r = 0.84)
Harz−8
35
30
25
20
20
25
30
35
Harz−1

Peptide Reproducibility between Bioreplicas
(condition: Harz) Harz−2 vs Harz−3
(n = 5950 r = 0.7)

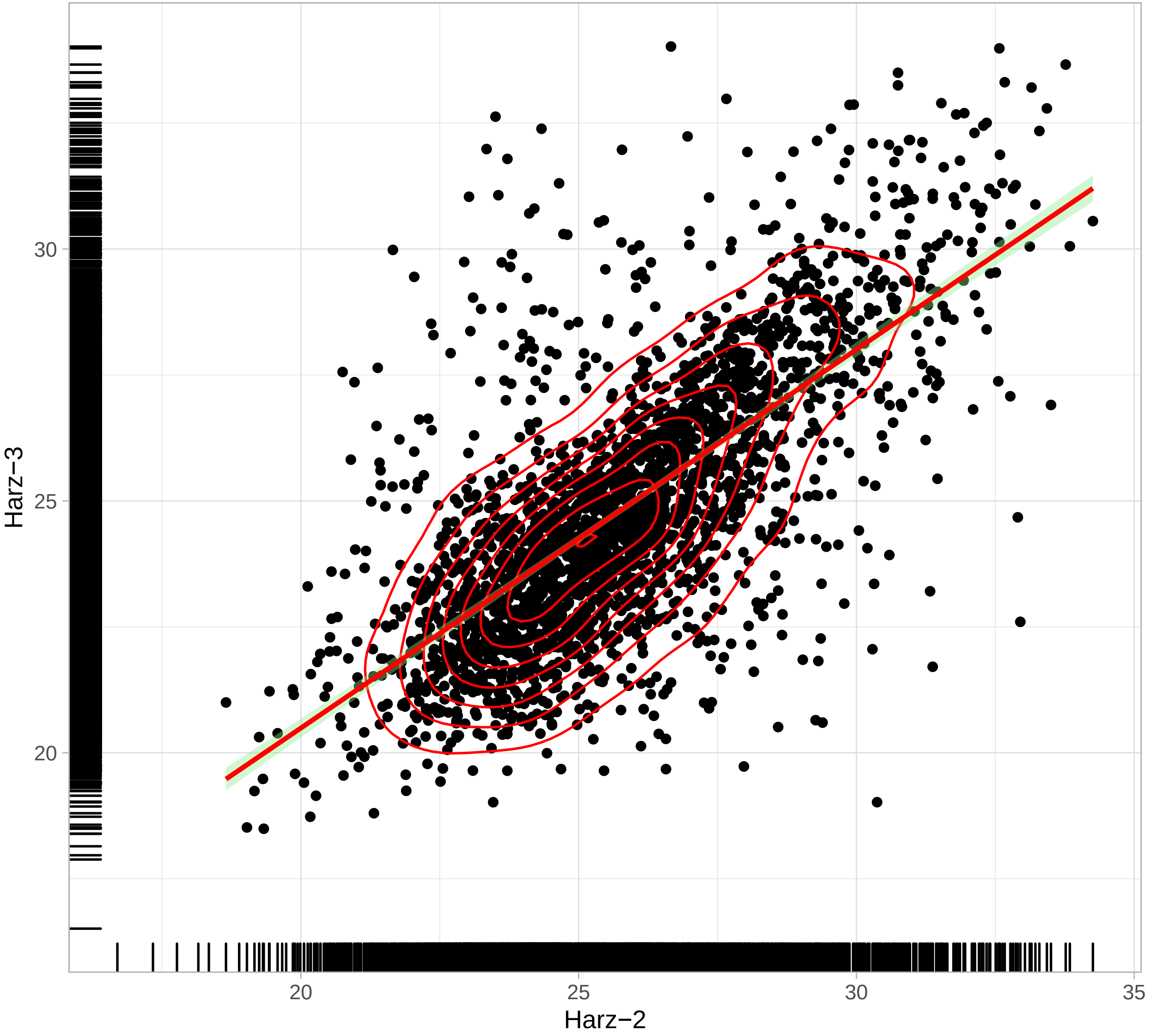

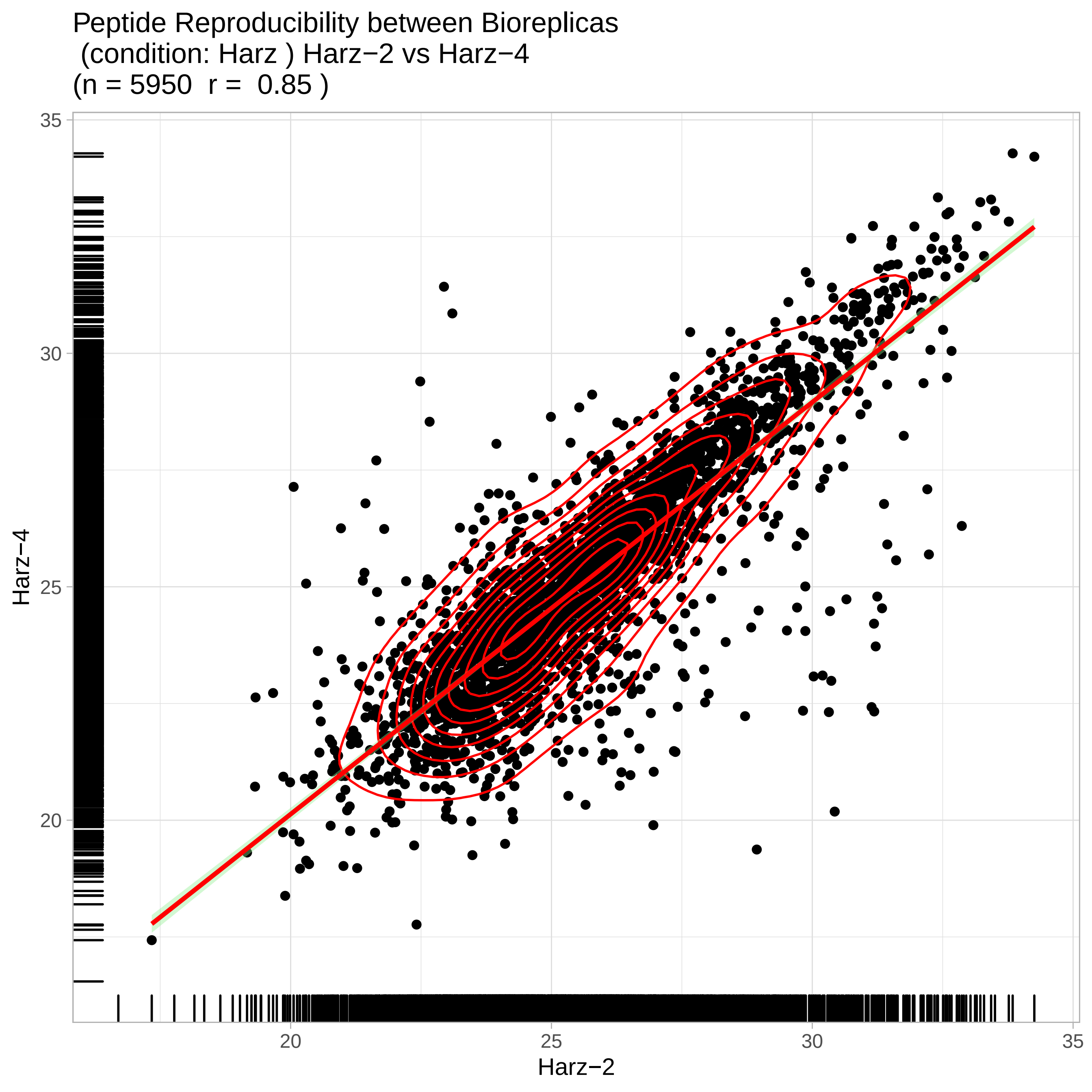
Peptide Reproducibility between Bioreplicas
(condition: Harz) Harz−2 vs Harz−4
(n = 5950 r = 0.85)
Harz−4
35
30
25
20
20
25
30
35
Harz−2

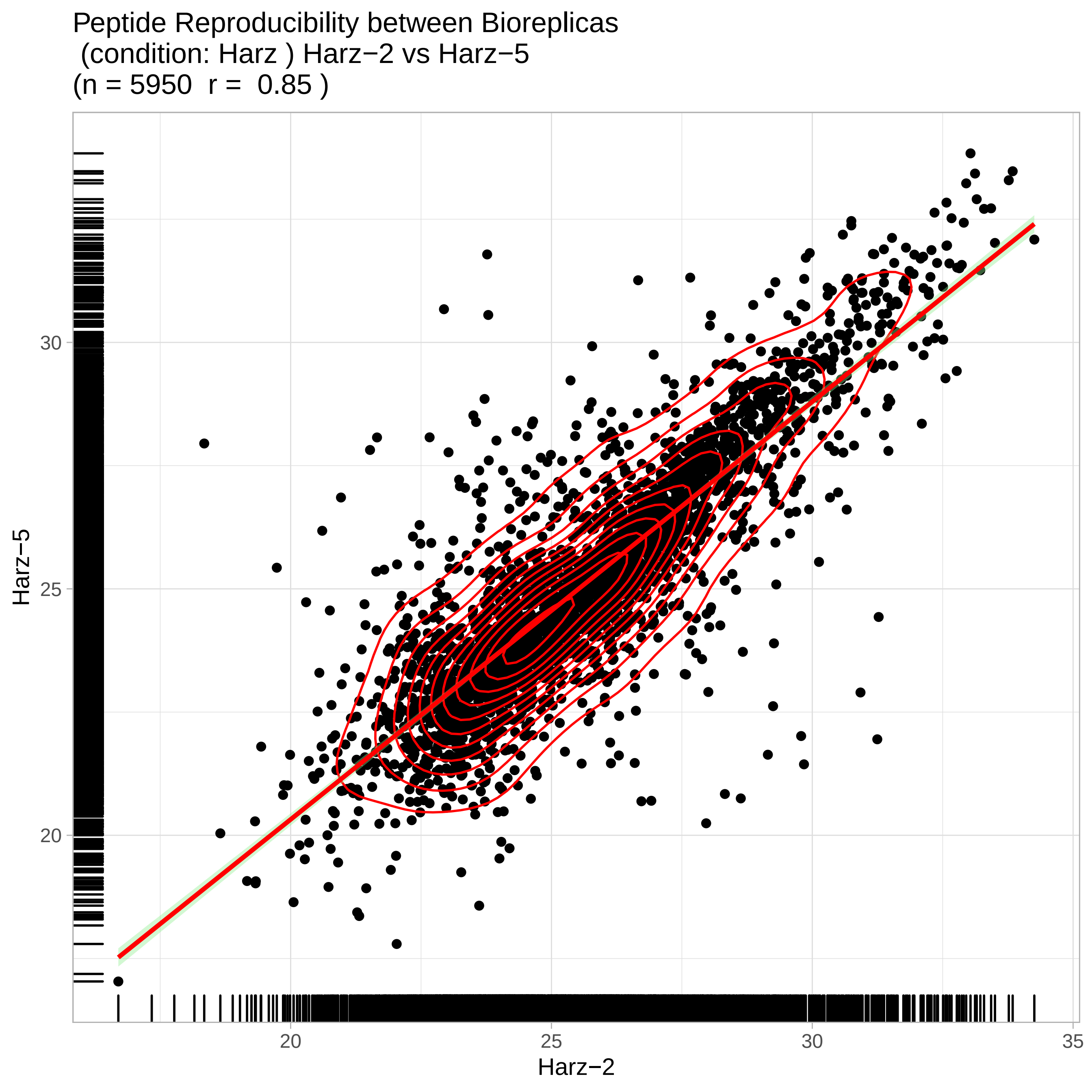
Peptide Reproducibility between Bioreplicas
(condition: Harz) Harz−2 vs Harz−5
(n = 5950 r = 0.85)
Harz−5
Harz−2
20
25
30
35

Peptide Reproducibility between Bioreplicas
(condition: Harz) Harz−2 vs Harz−6
(n = 5950 r = 0.85)

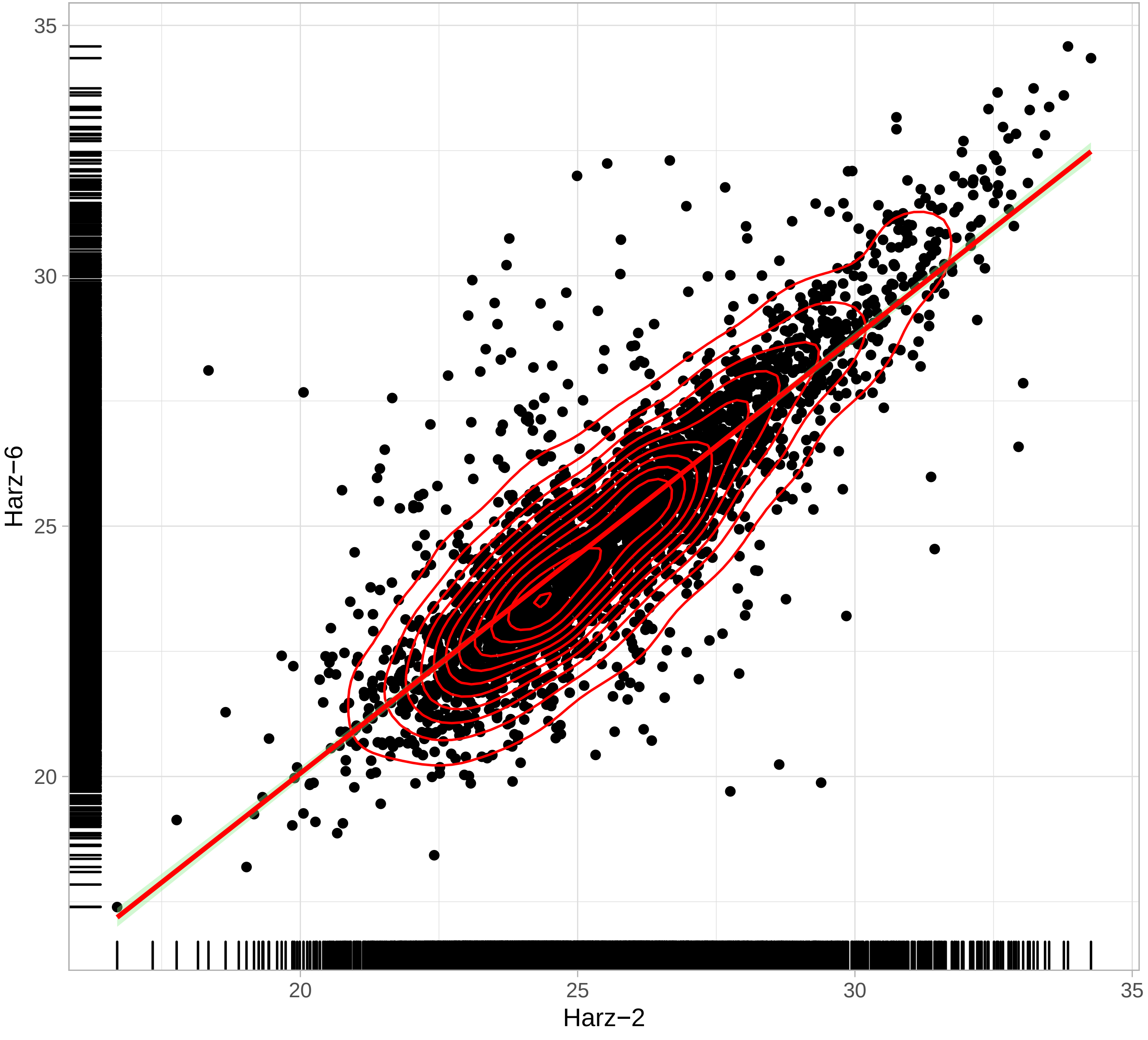

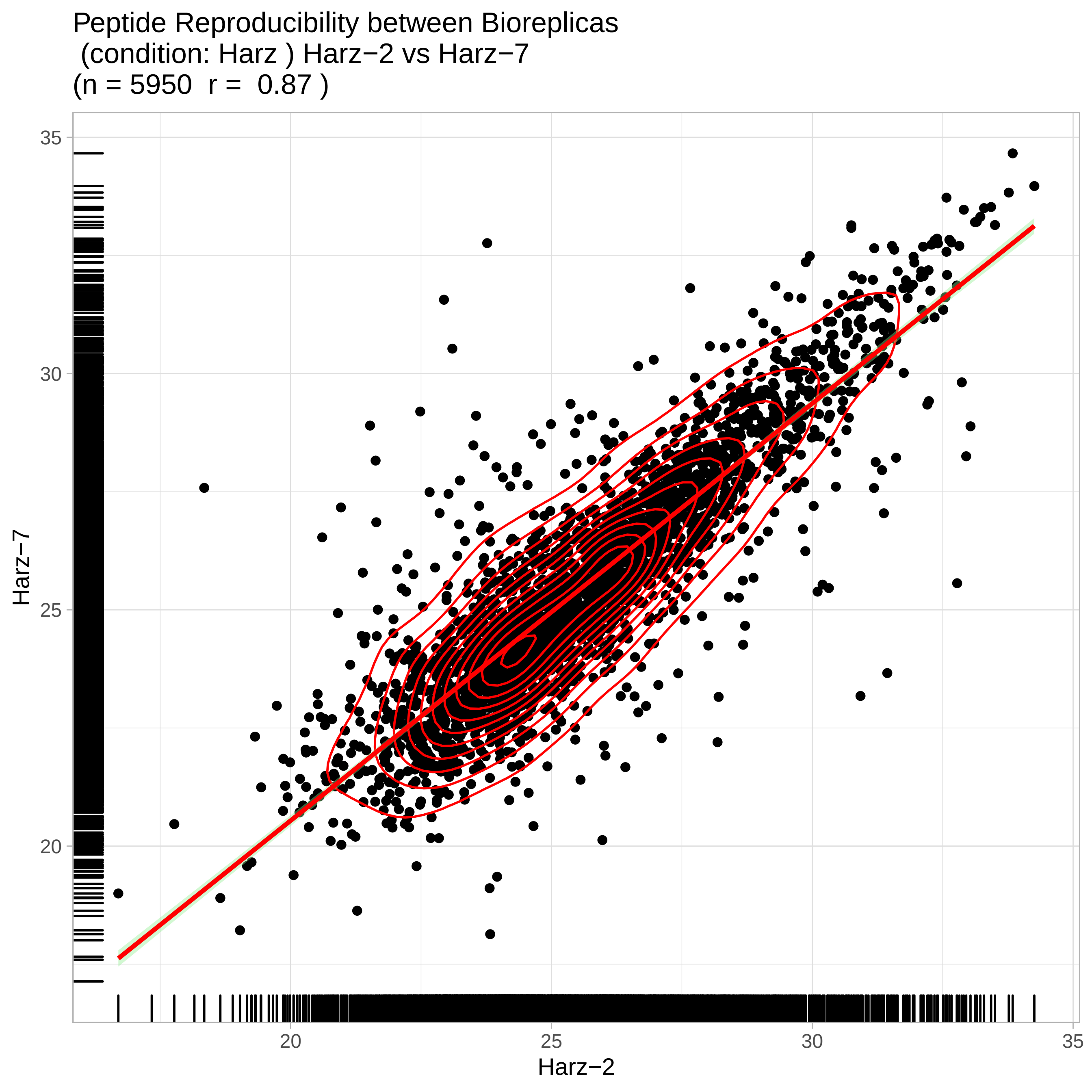
Peptide Reproducibility between Bioreplicas
(condition: Harz) Harz−2 vs Harz−7
(n = 5950 r = 0.87)
Harz−7
35
30
25
20
20
25
30
35
Harz−2

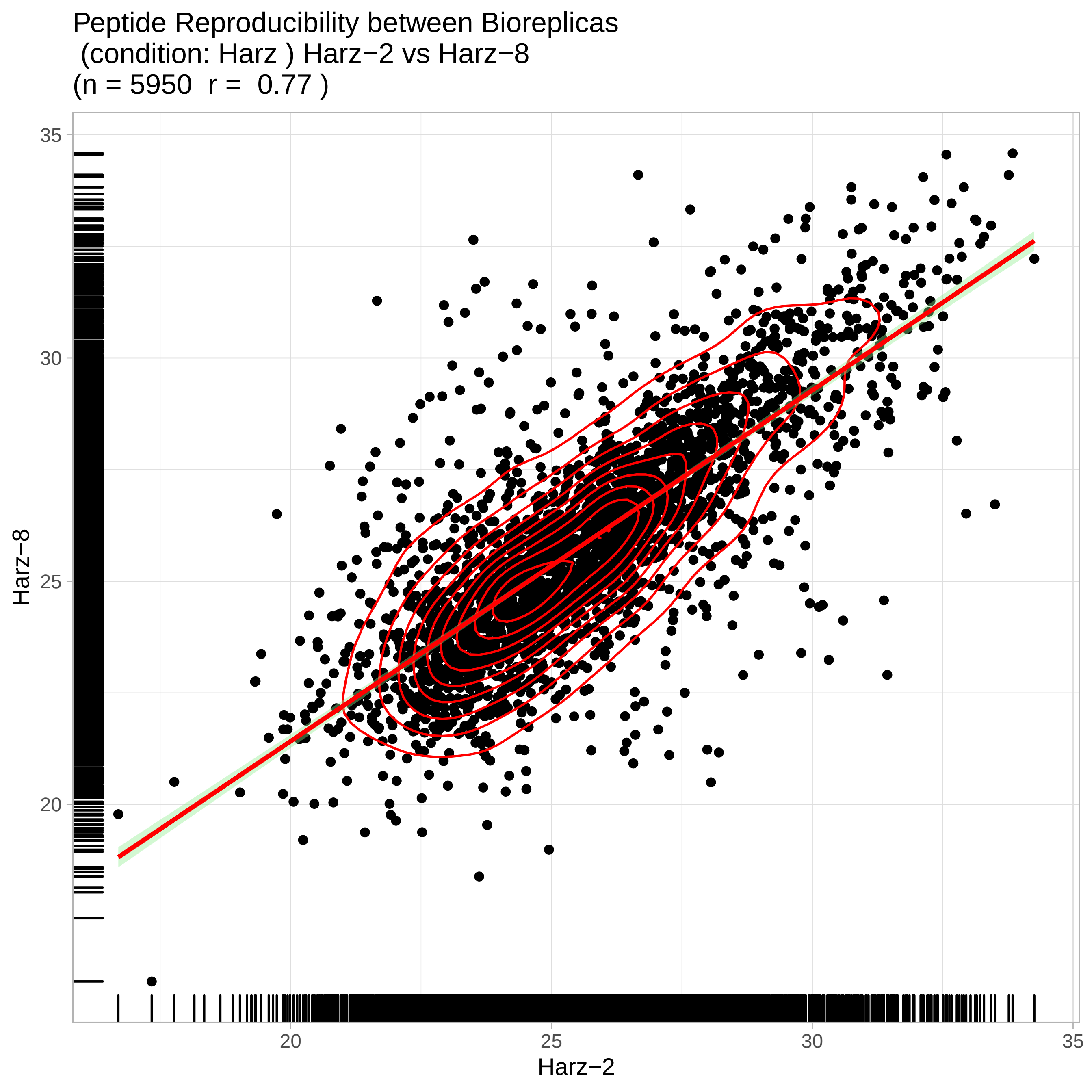

Peptide Reproducibility between Bioreplicas
(condition: Harz) Harz−2 vs Harz−8
(n = 5950 r = 0.77)
Harz−8
Harz−2
35
30
25
20
20
25
30
35

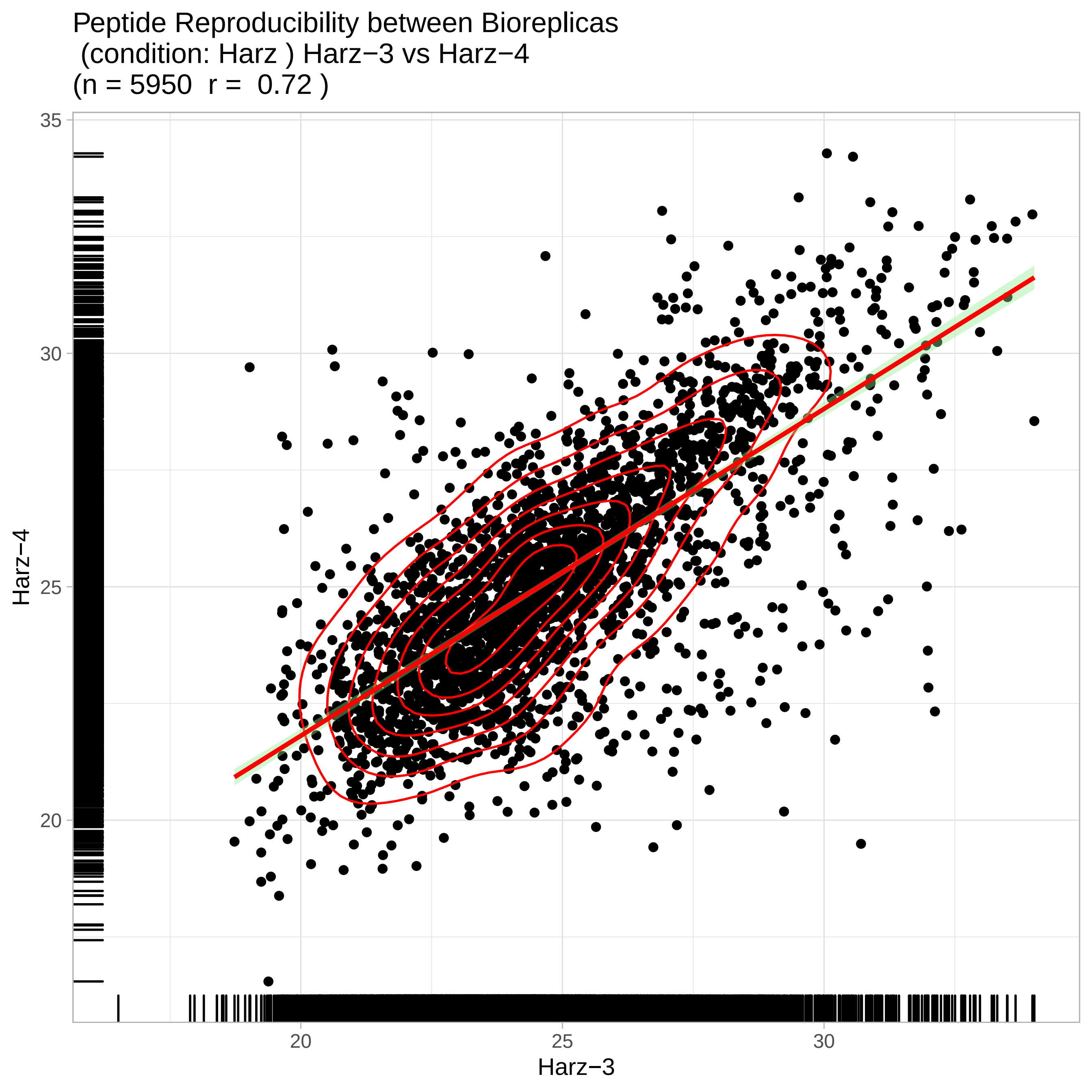
Peptide Reproducibility between Bioreplicas
(condition: Harz) Harz−3 vs Harz−4
(n = 5950 r = 0.72)
Harz−4
Harz−3
35
30
25
20
20
25
30

Peptide Reproducibility between Bioreplicas
 (condition: Harz) Harz−3 vs Harz−5
(n = 5950 r = 0.81)

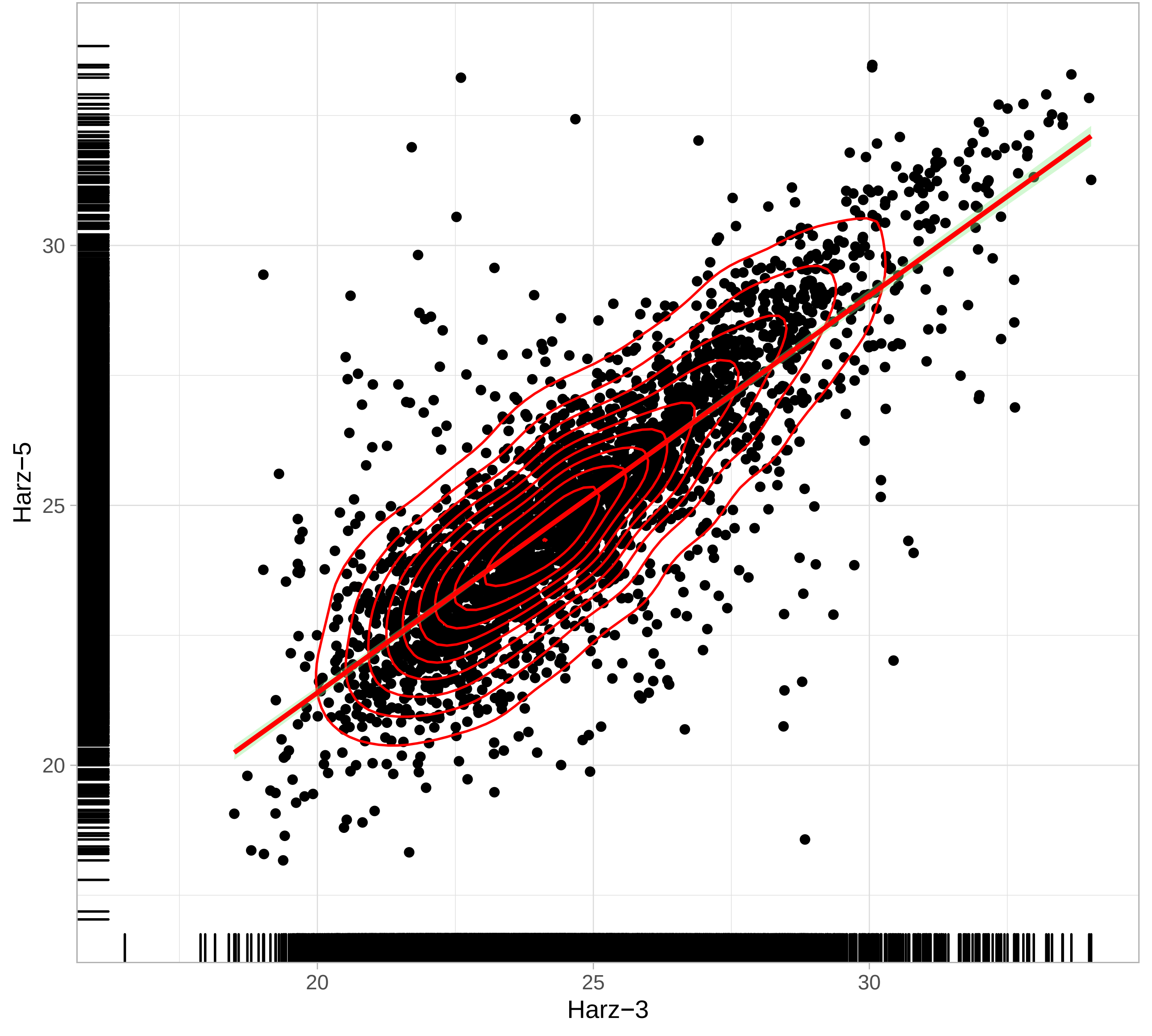

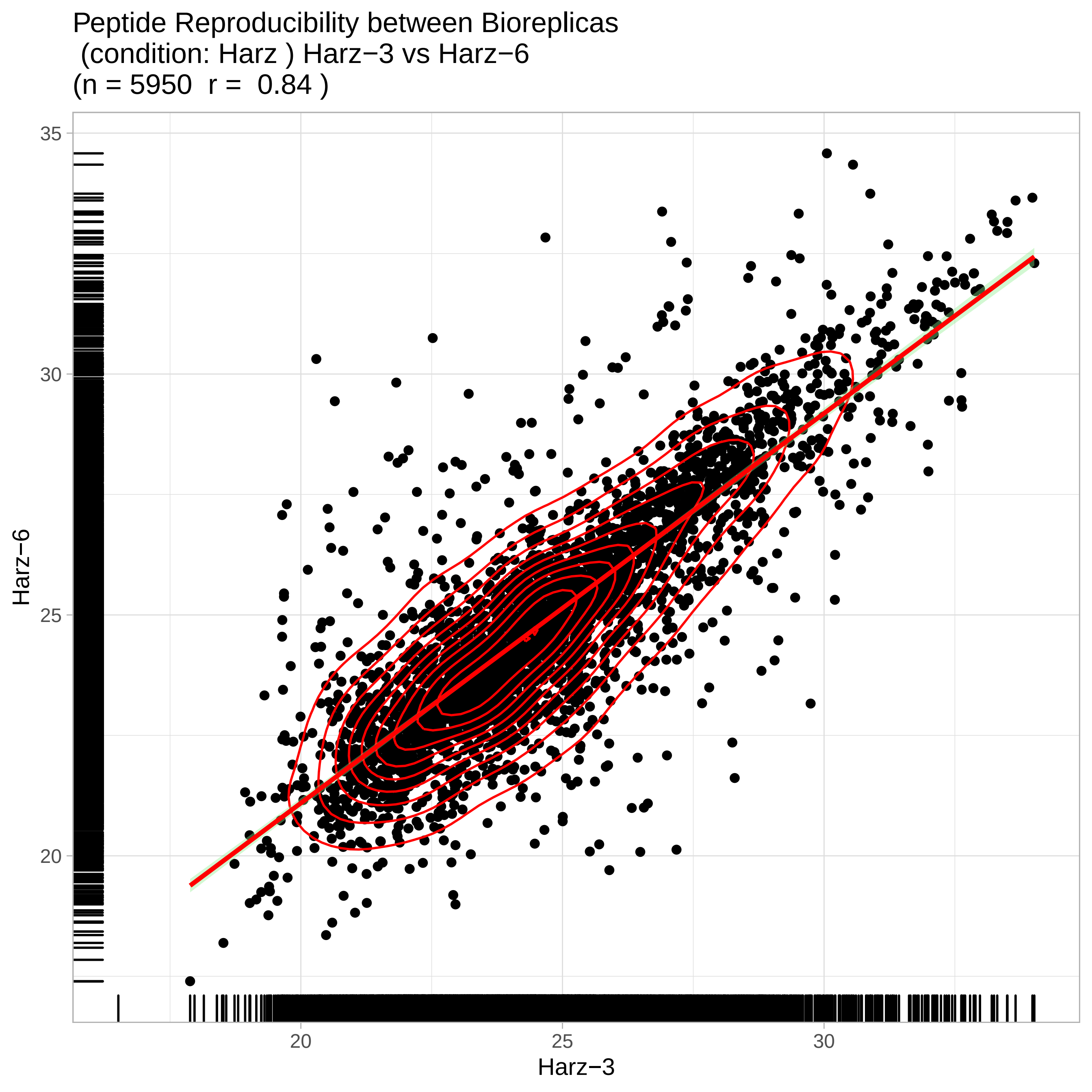

Peptide Reproducibility between Bioreplicas
(condition: Harz) Harz−3 vs Harz−6
(n = 5950 r = 0.84)
35
30
25
20
Harz−6
20
25
30
Harz−3

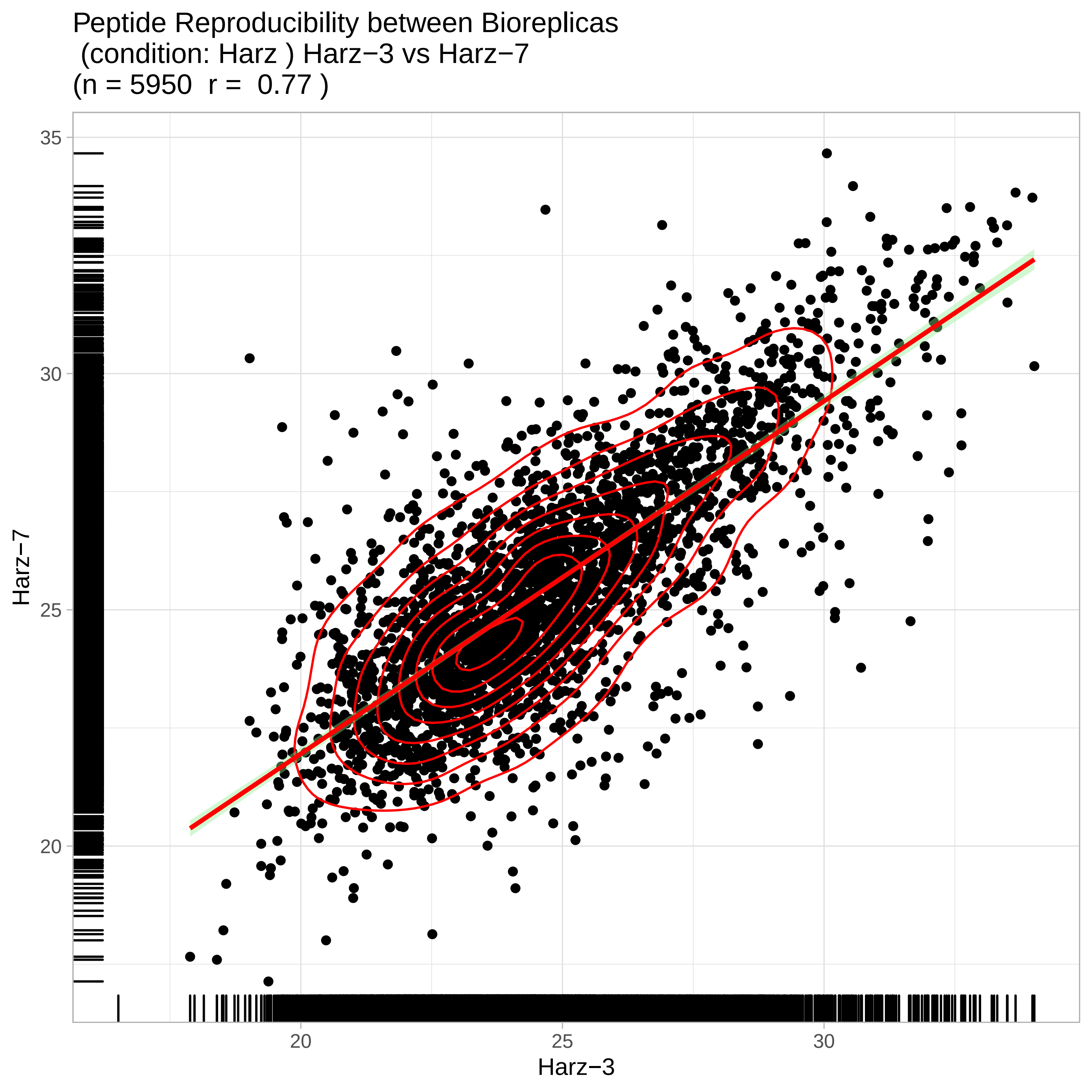
Peptide Reproducibility between Bioreplicas
(condition: Harz) Harz−3 vs Harz−7
(n = 5950 r = 0.77)
Harz−7
Harz−3
35
30
25
20
20
25
30

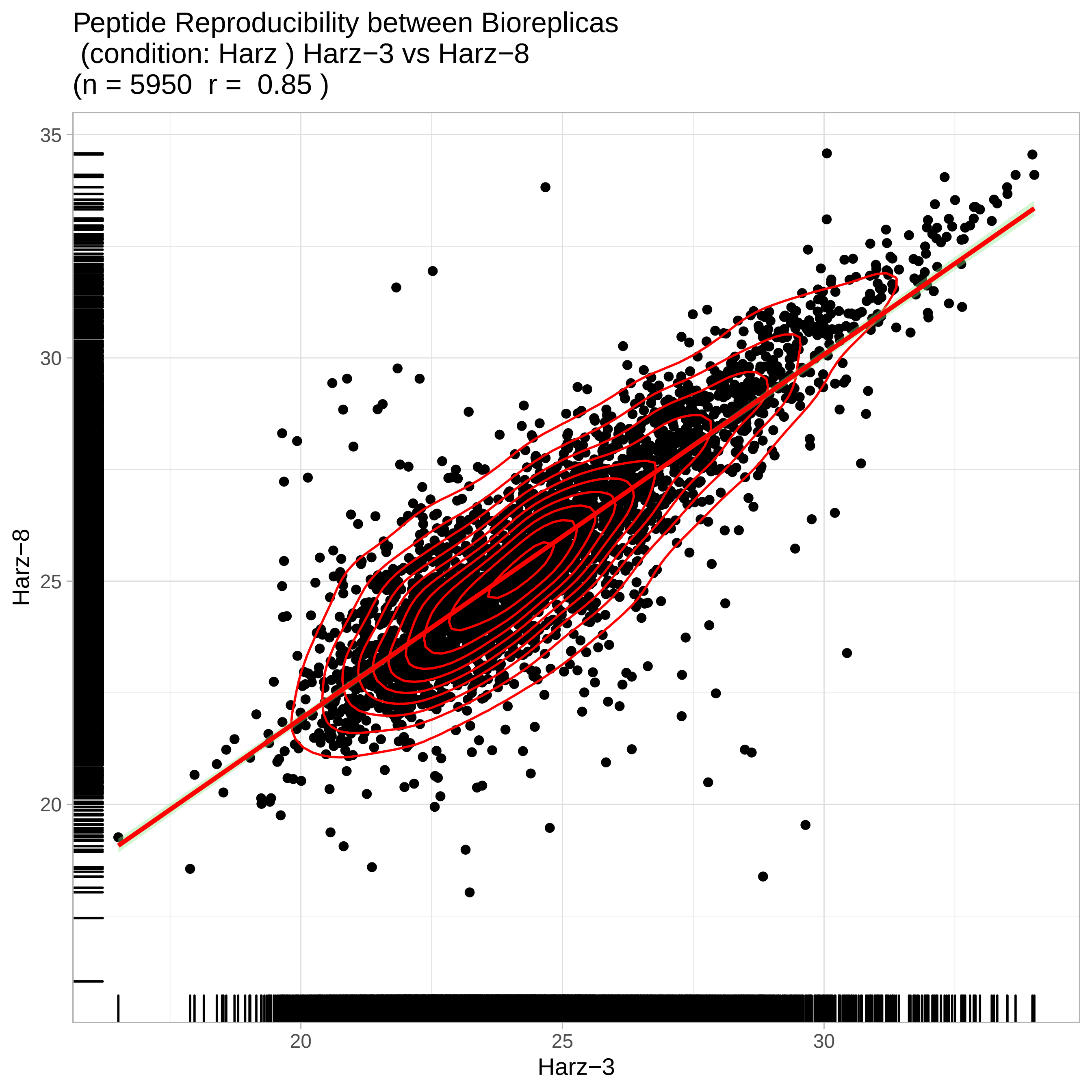
Peptide Reproducibility between Bioreplicas
(condition: Harz) Harz−3 vs Harz−8
(n = 5950 r = 0.85)
Harz−8
Harz−3
35
30
25
20
20
25
30

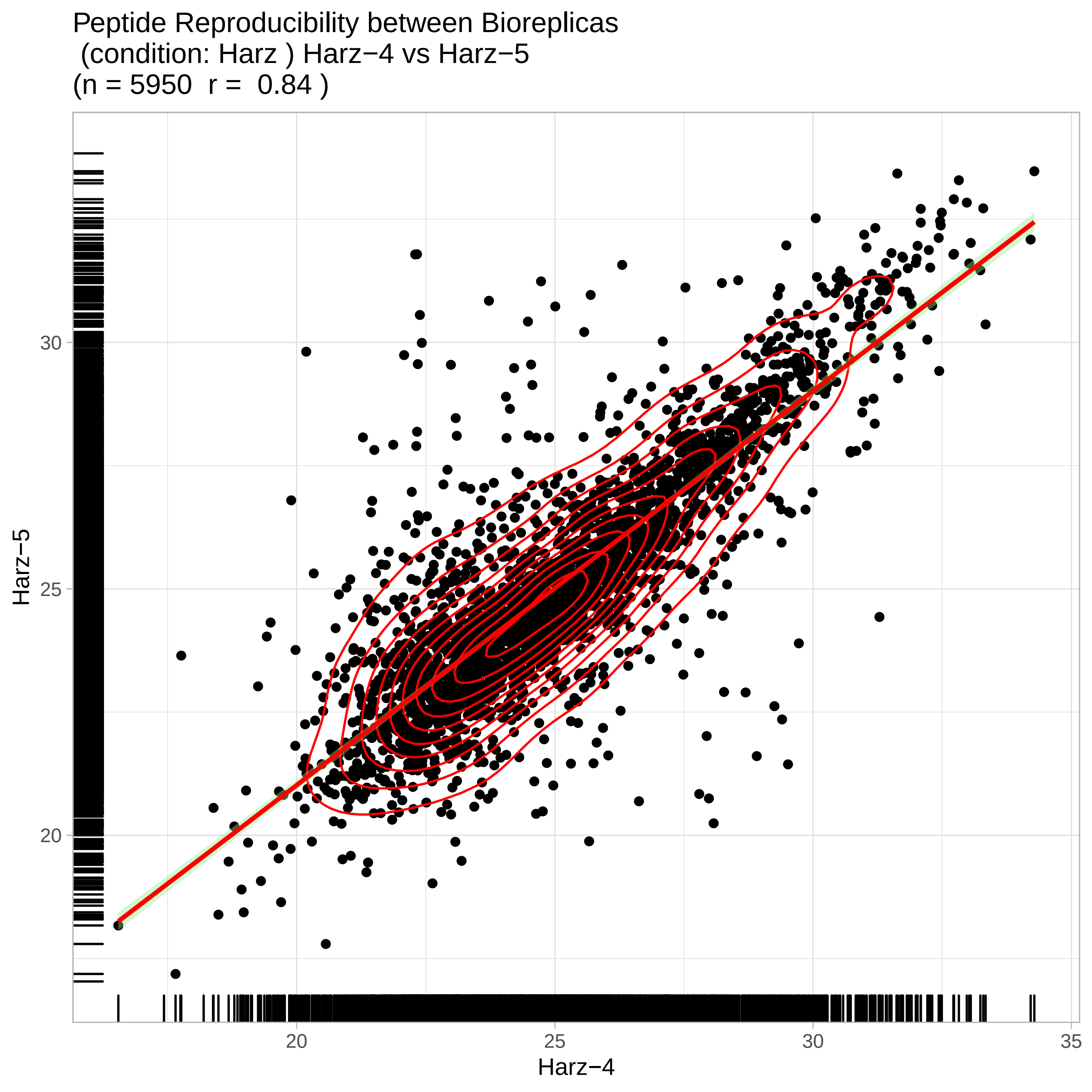
Peptide Reproducibility between Bioreplicas
(condition: Harz) Harz−4 vs Harz−5
(n = 5950 r = 0.84)
Harz−5
30
25
20
20
25
30
35
Harz−4

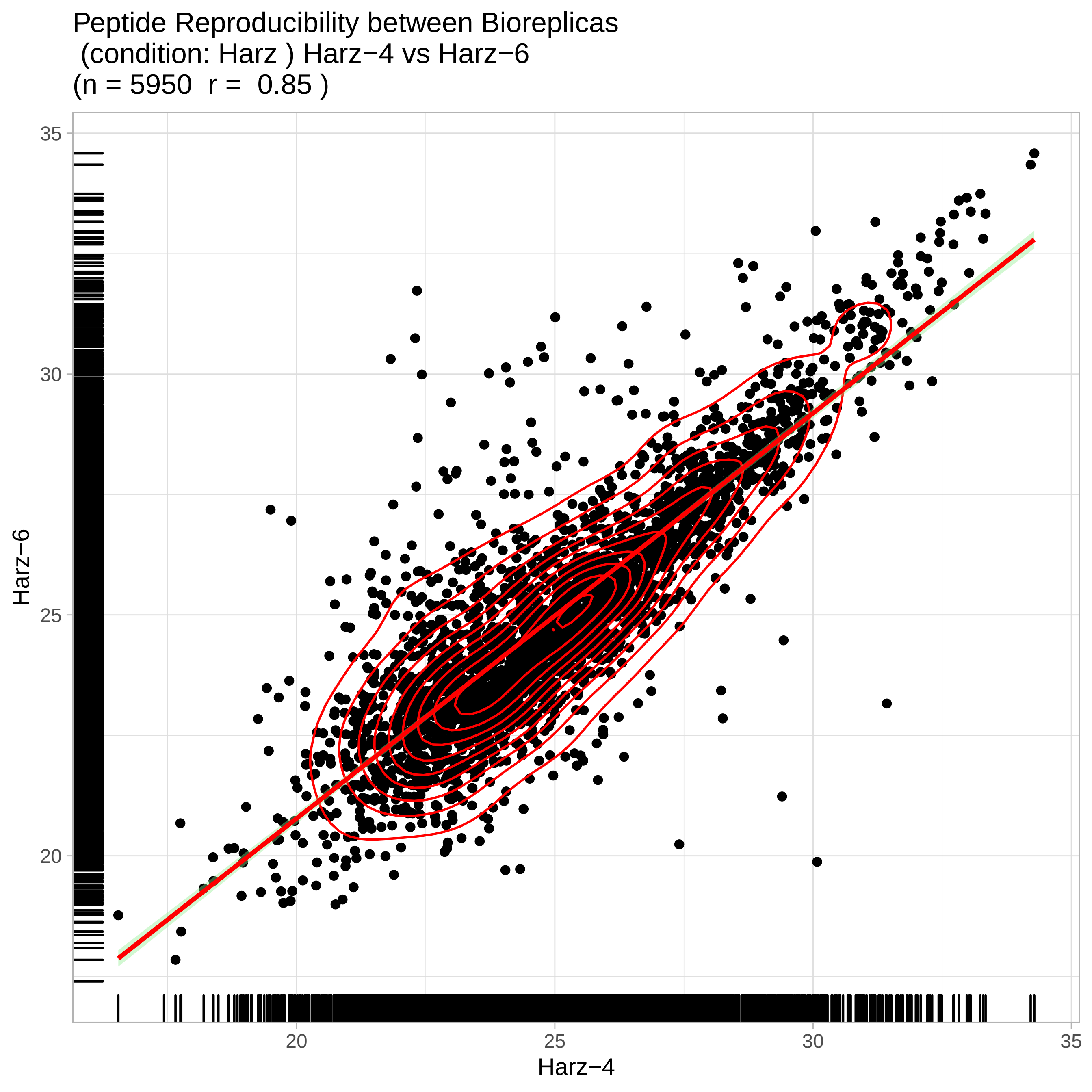

Peptide Reproducibility between Bioreplicas
(condition: Harz) Harz−4 vs Harz−6
(n = 5950 r = 0.85)
35
30
25
20
Harz−6
20
25
30
35
Harz−4

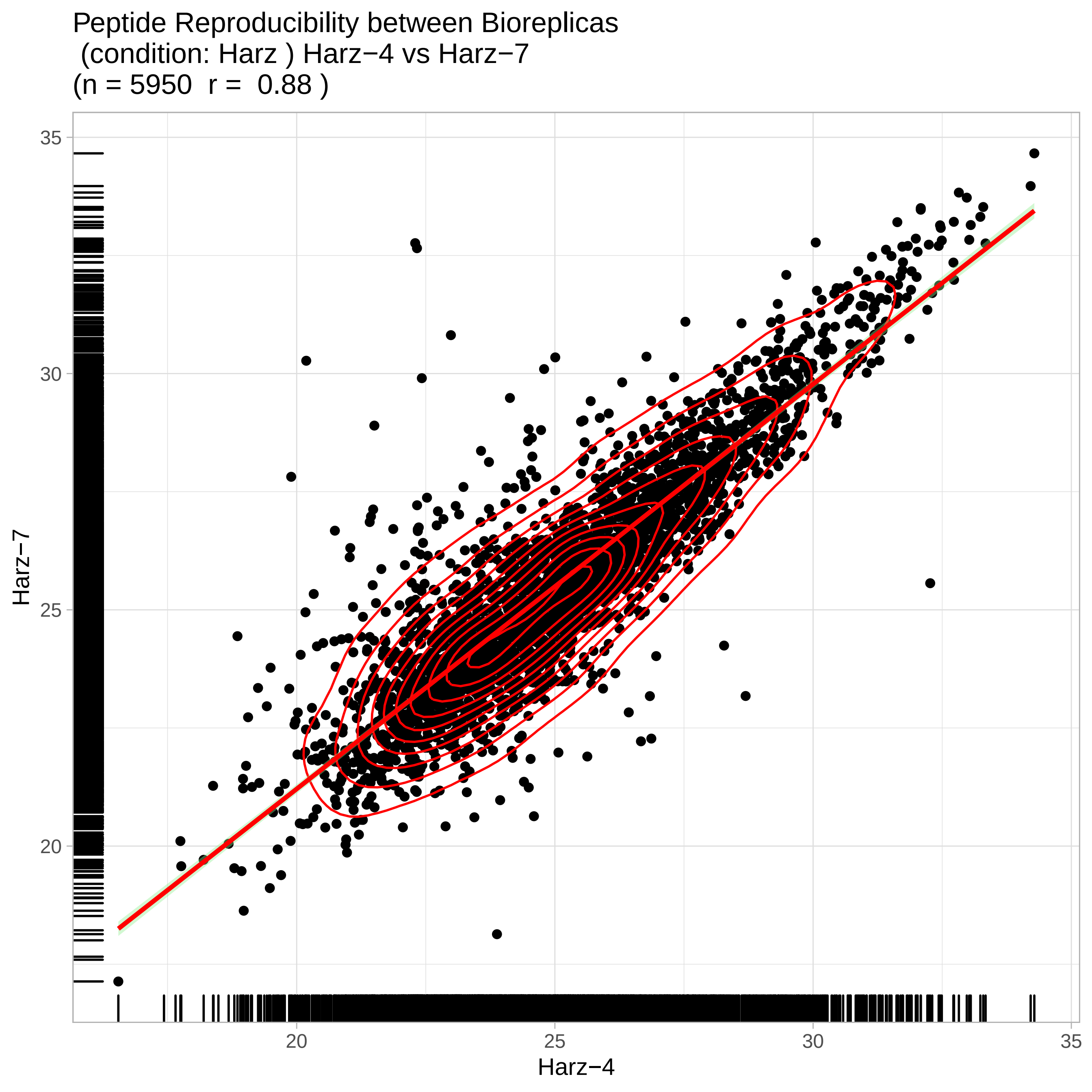
Peptide Reproducibility between Bioreplicas
(condition: Harz) Harz−4 vs Harz−7
(n = 5950 r = 0.88)
Harz−7
35
30
25
20
20
25
30
35
Harz−4

Peptide Reproducibility between Bioreplicas
(condition: Harz) Harz−4 vs Harz−8
(n = 5950 r = 0.79)

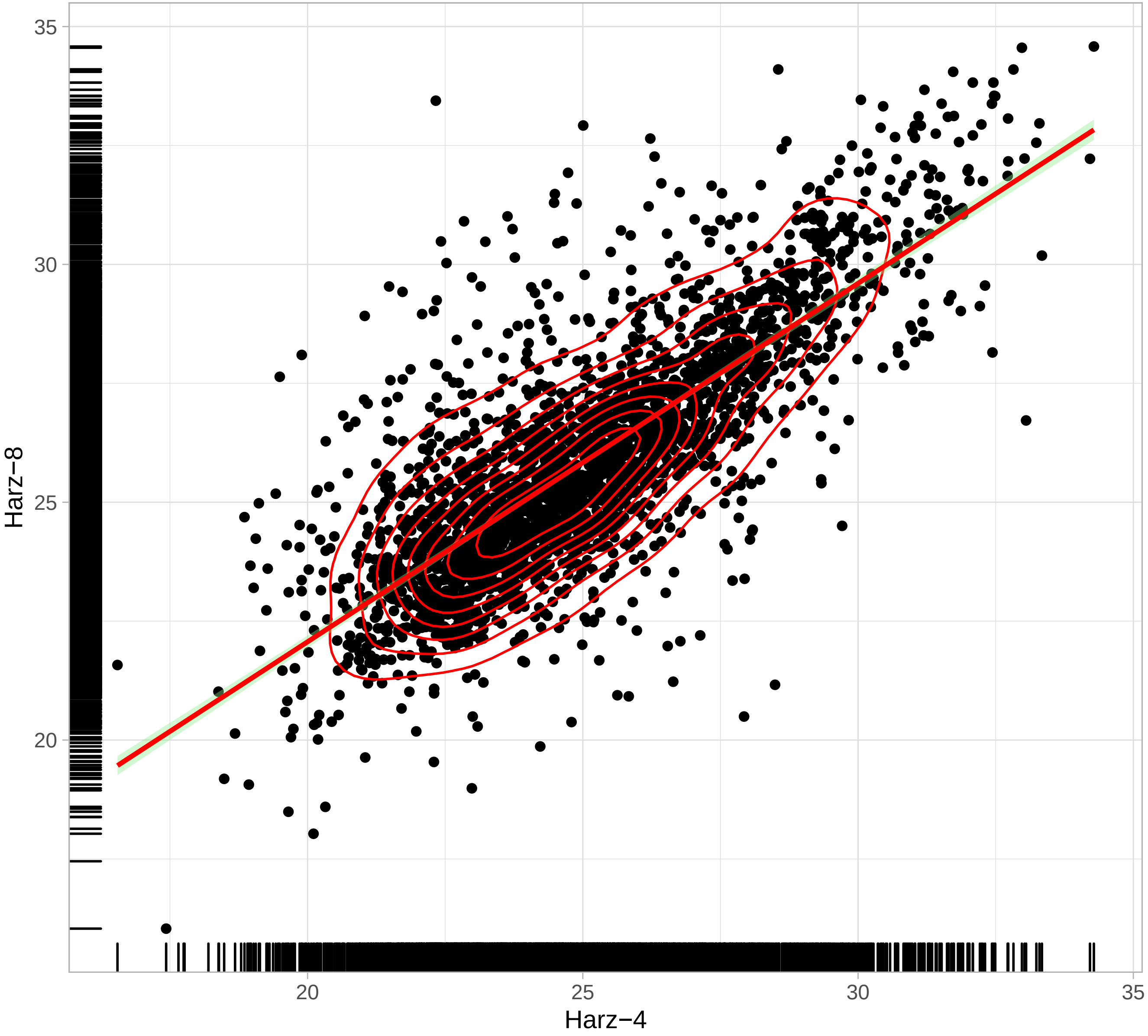

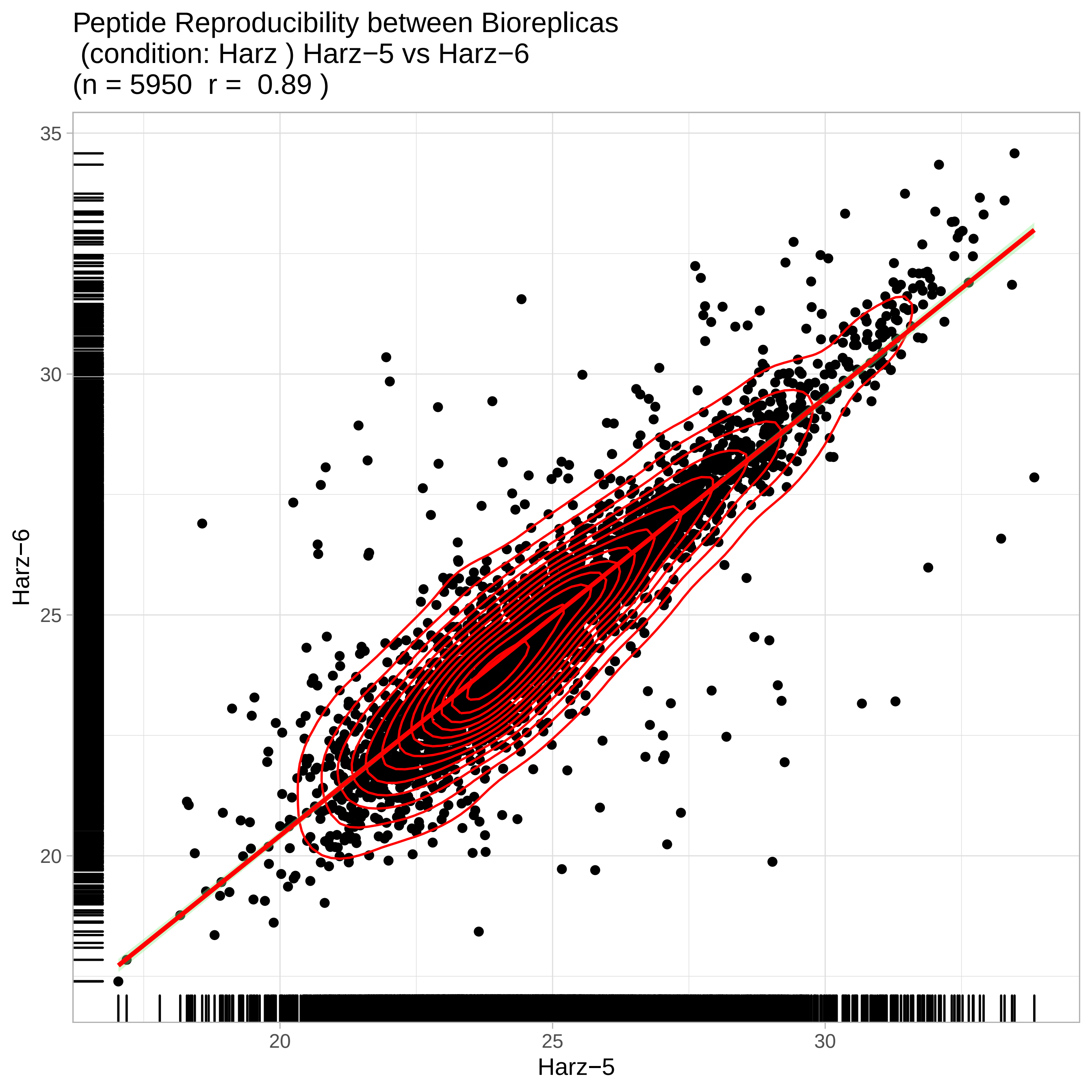

Peptide Reproducibility between Bioreplicas
(condition: Harz) Harz−5 vs Harz−6
(n = 5950 r = 0.89)
35
30
25
20
Harz−6
20
25
30
Harz−5

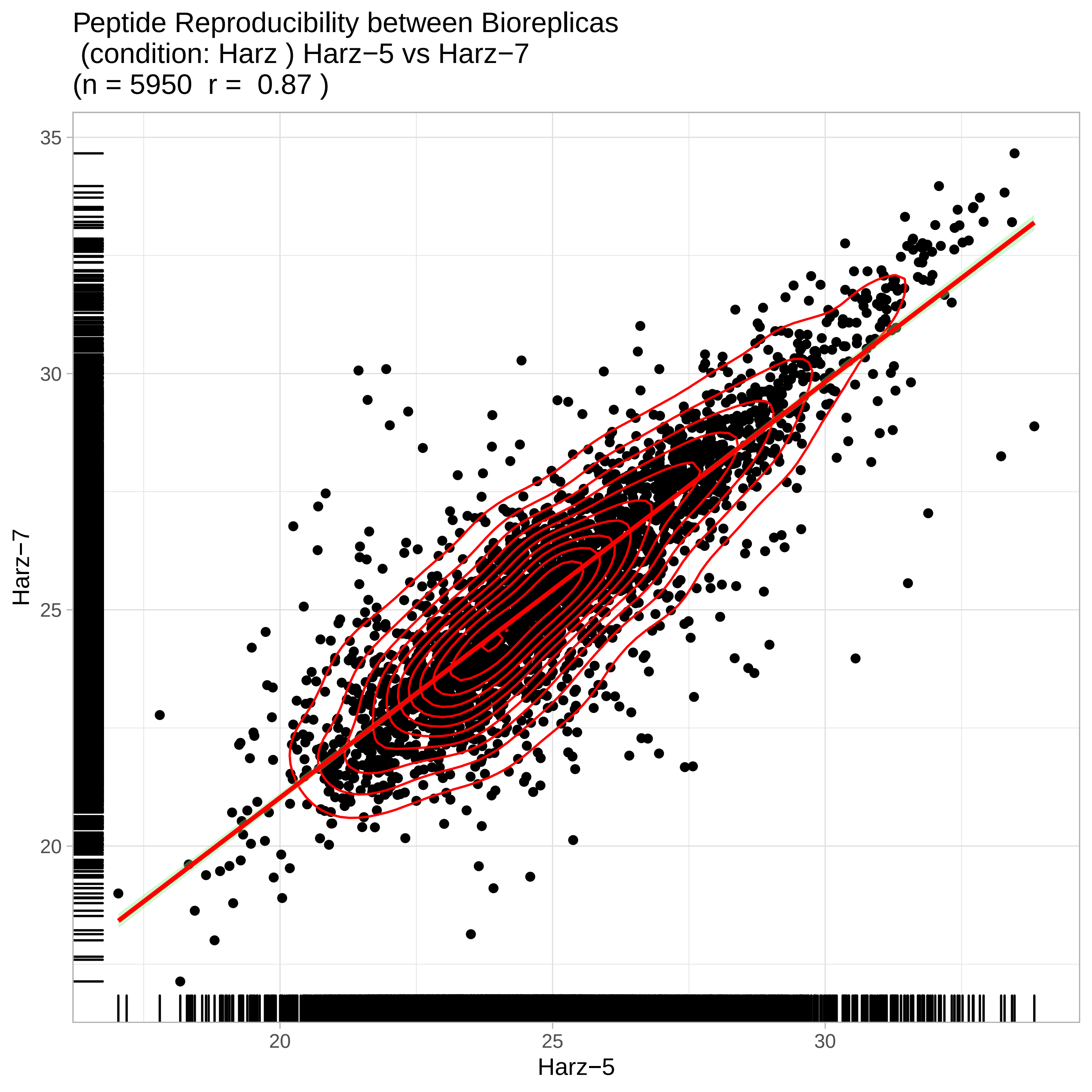

Peptide Reproducibility between Bioreplicas
(condition: Harz) Harz−5 vs Harz−7
(n = 5950 r = 0.87)
35
30
25
20
20
25
30
Harz−7
Harz−5

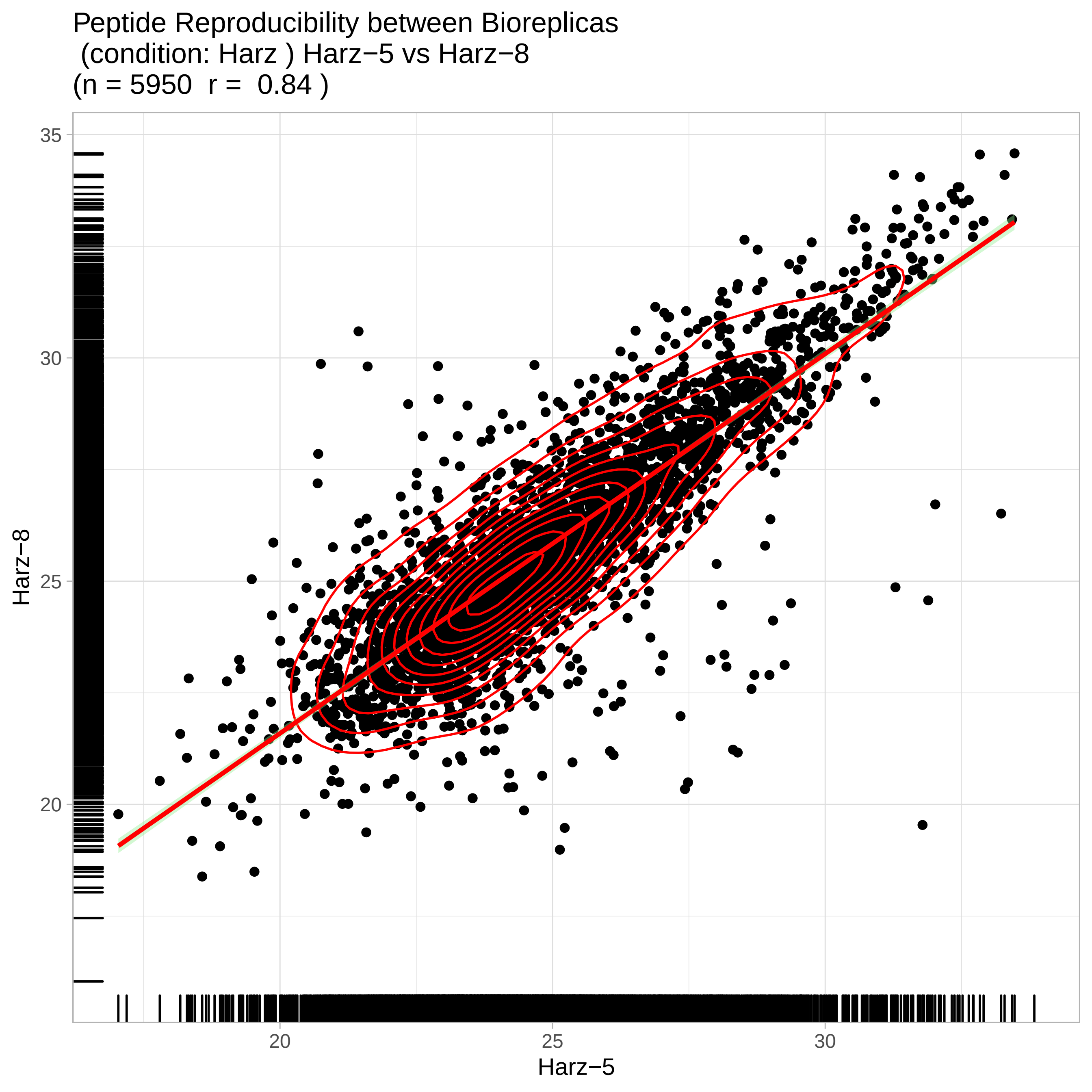

Peptide Reproducibility between Bioreplicas
(condition: Harz) Harz−5 vs Harz−8
(n = 5950 r = 0.84)
Harz−8
35
30
25
20
20
25
30
Harz−5

Peptide Reproducibility between Bioreplicas
(condition: Harz) Harz−6 vs Harz−7
(n = 5950 r = 0.89)

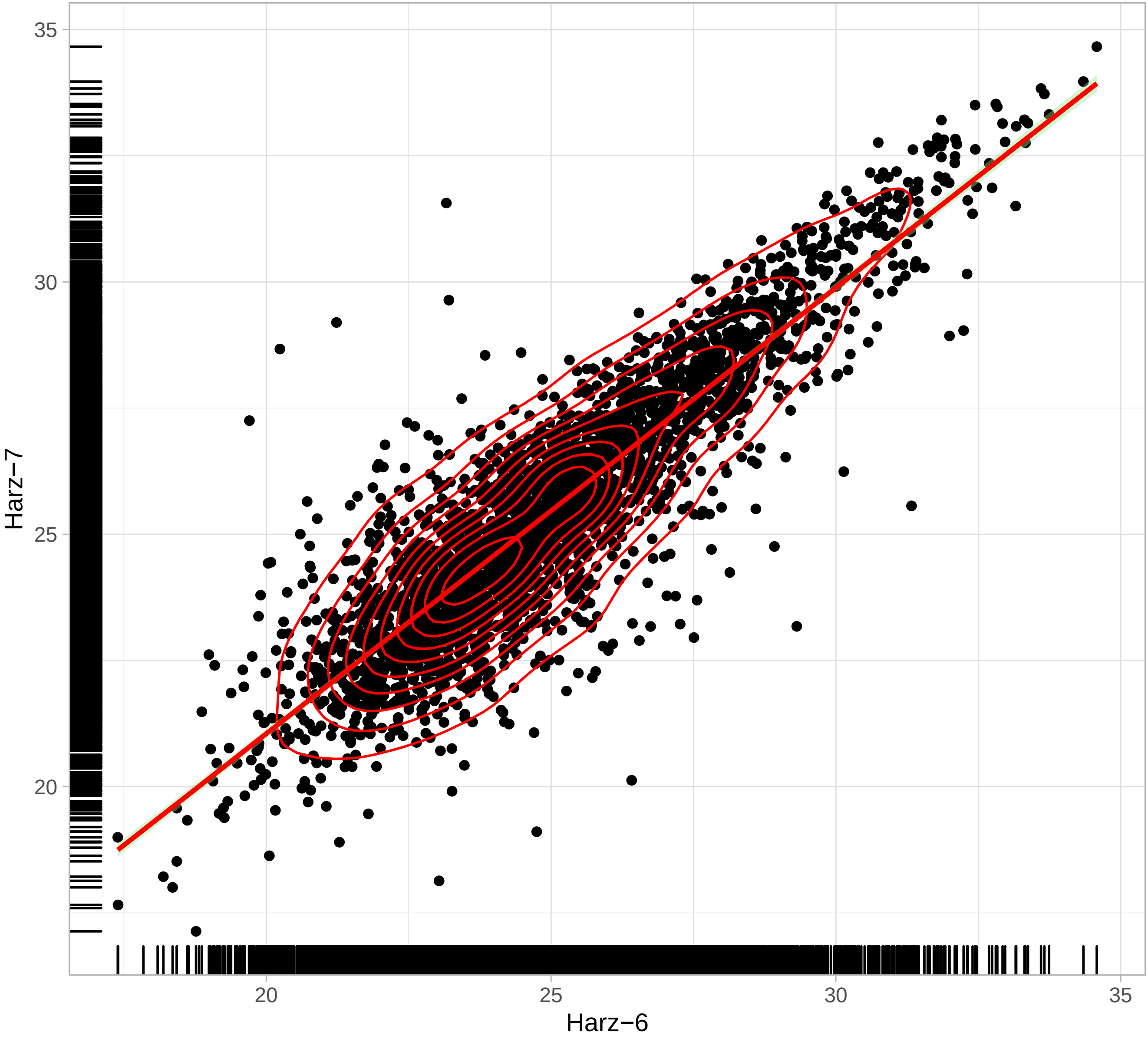

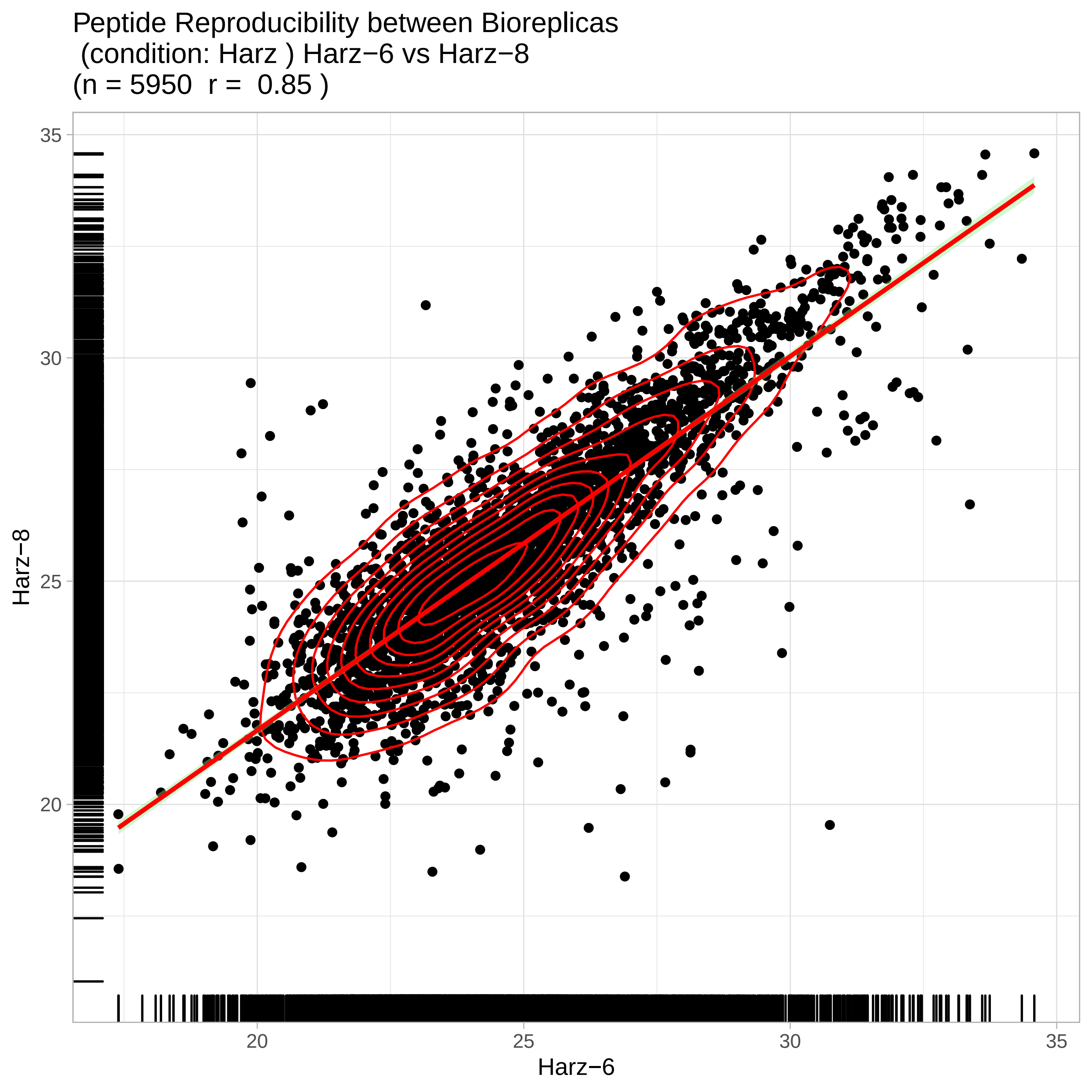
Peptide Reproducibility between Bioreplicas
(condition: Harz) Harz−6 vs Harz−8
(n = 5950 r = 0.85)
Harz−8
Harz−6
35
30
25
20
20
25
30
35

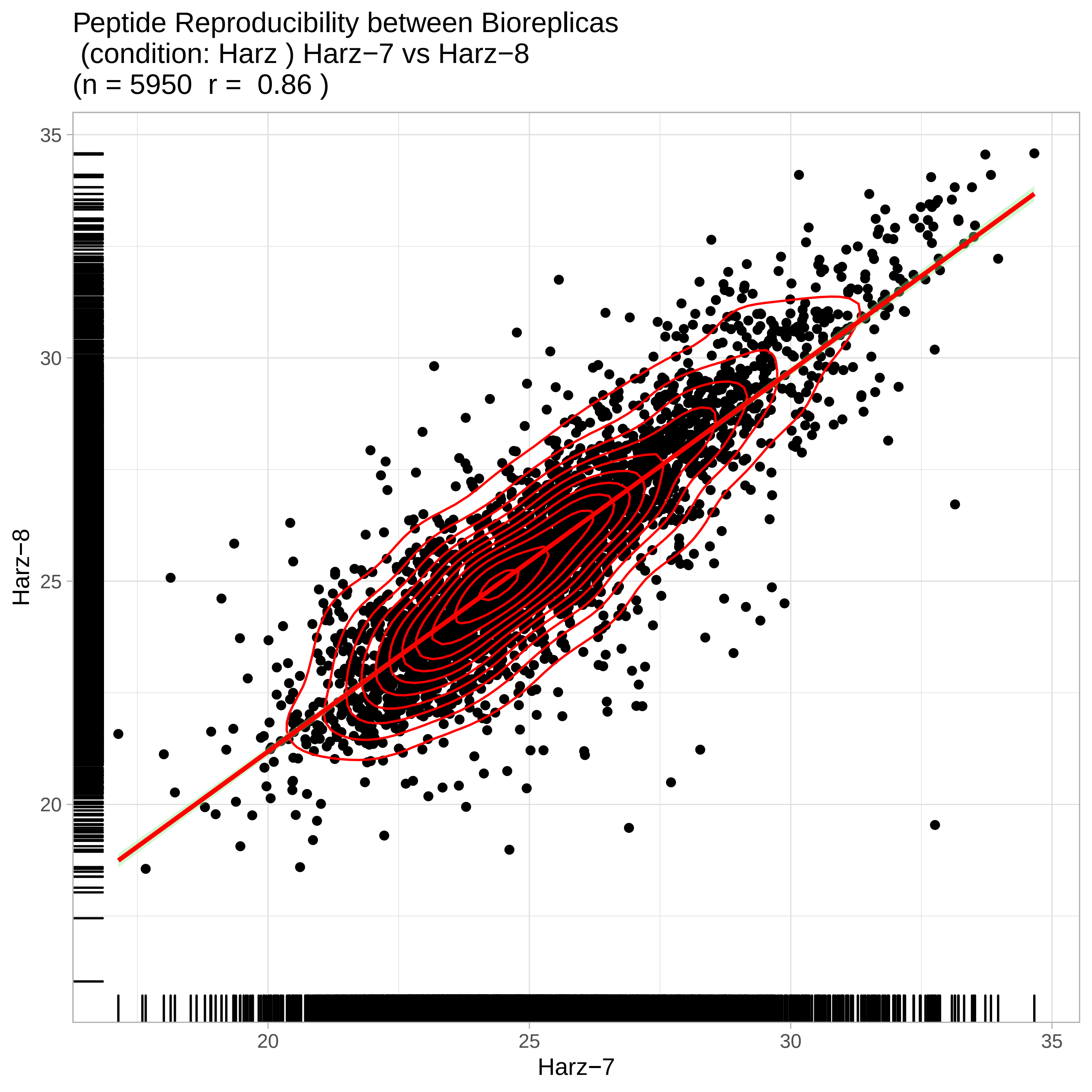
Peptide Reproducibility between Bioreplicas
(condition: Harz) Harz−7 vs Harz−8
(n = 5950 r = 0.86)
Harz−8
Harz−7
20
25
30
35

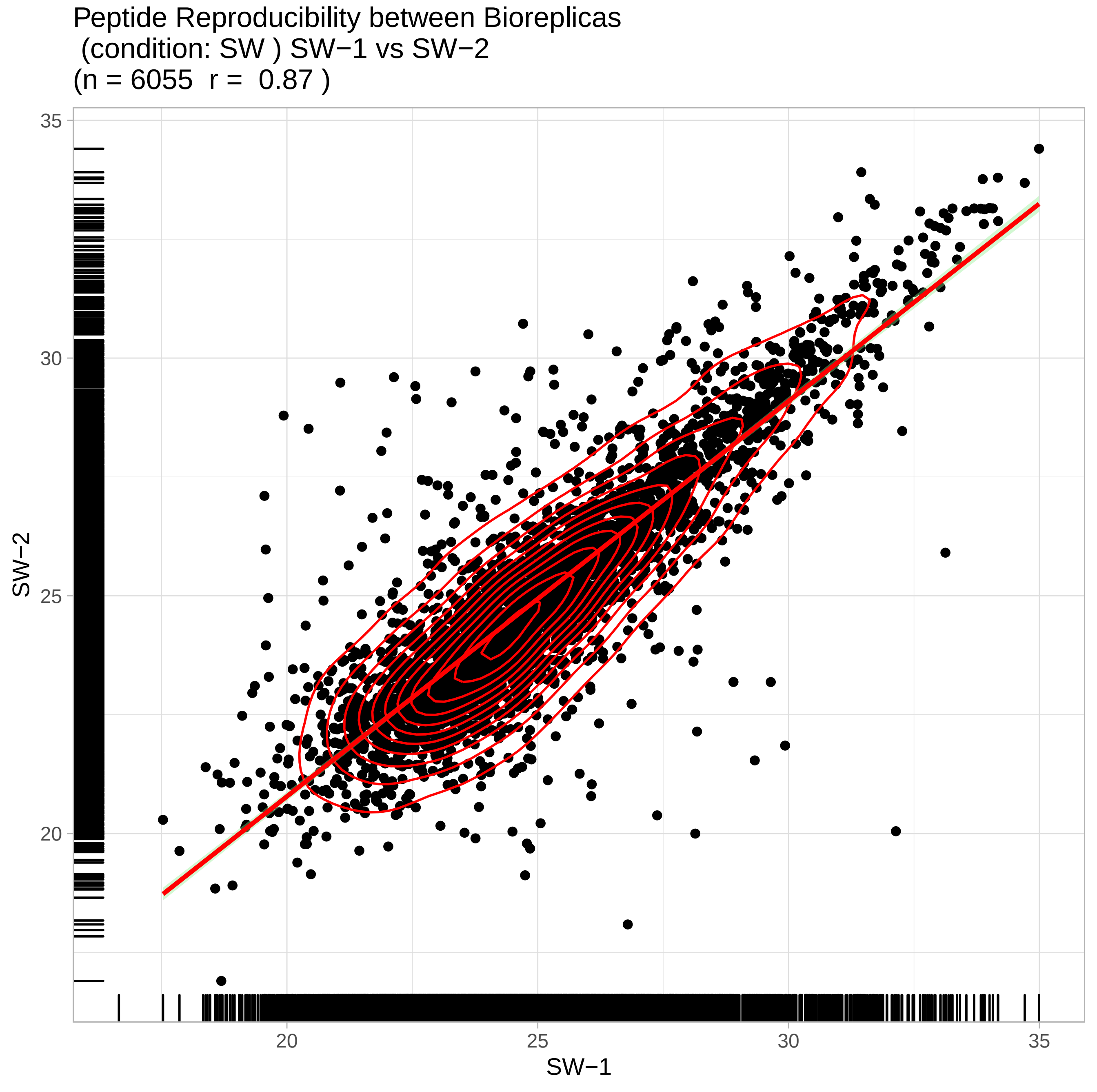

Peptide Reproducibility between Bioreplicas
(condition: SW) SW−1 vs SW−2
(n = 6055 r = 0.87)
35
30
25
20
SW−2
20
25
30
35
SW−1

Peptide Reproducibility between Bioreplicas
(condition: SW) SW−1 vs SW−3
(n = 6055 r = 0.86)

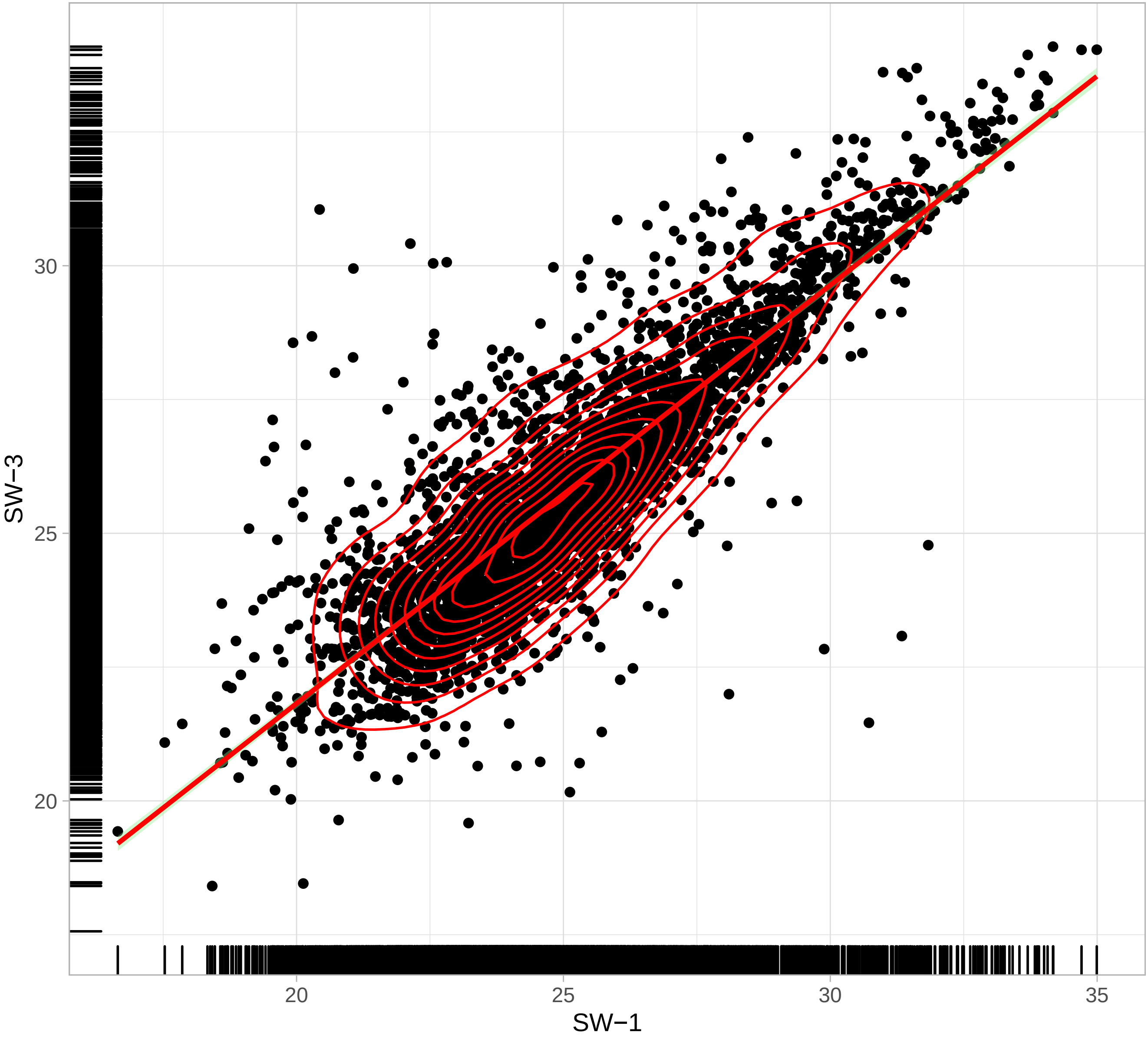

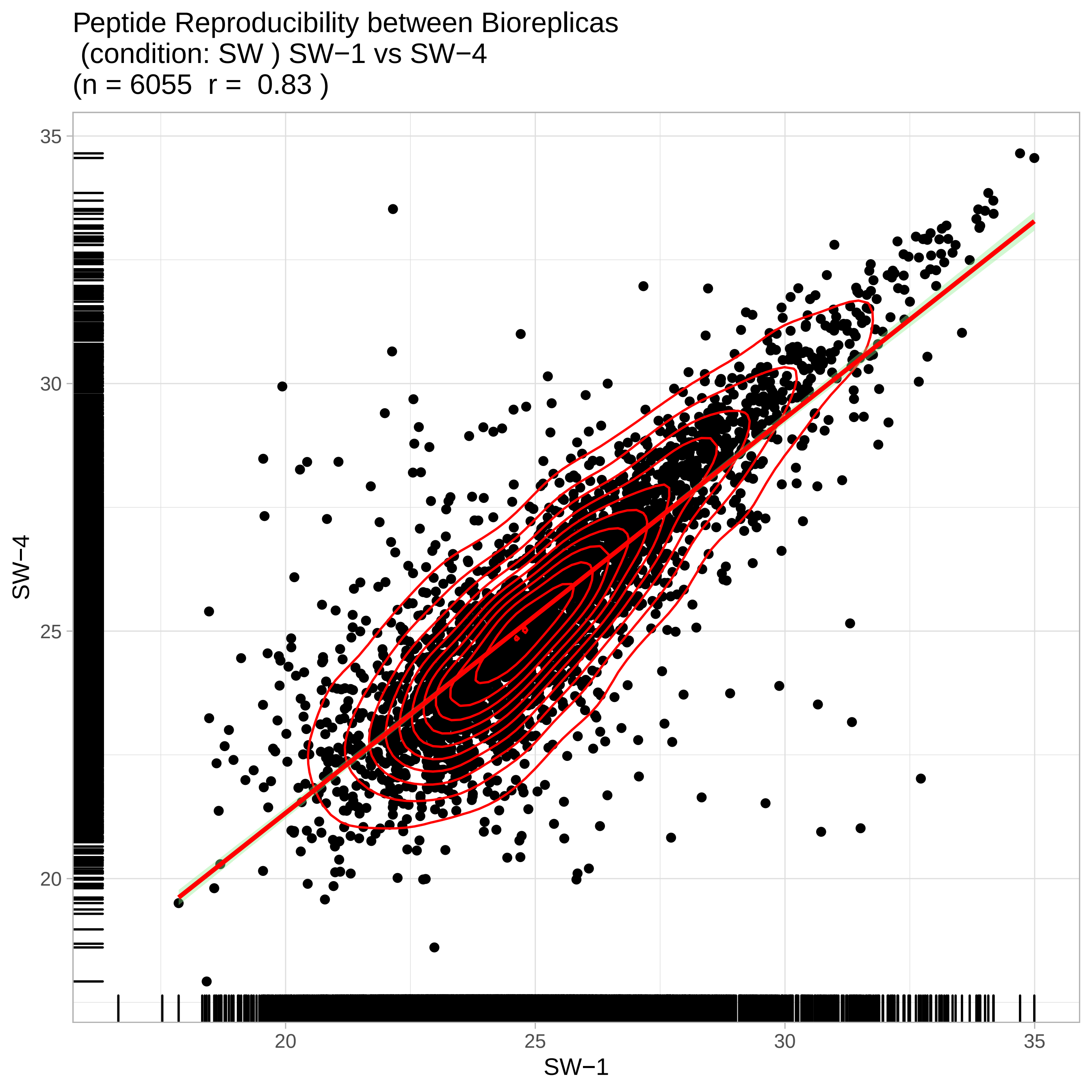
Peptide Reproducibility between Bioreplicas
(condition: SW) SW−1 vs SW−4
(n = 6055 r = 0.83)
SW−4
35
30
25
20
20
25
30
35
SW−1

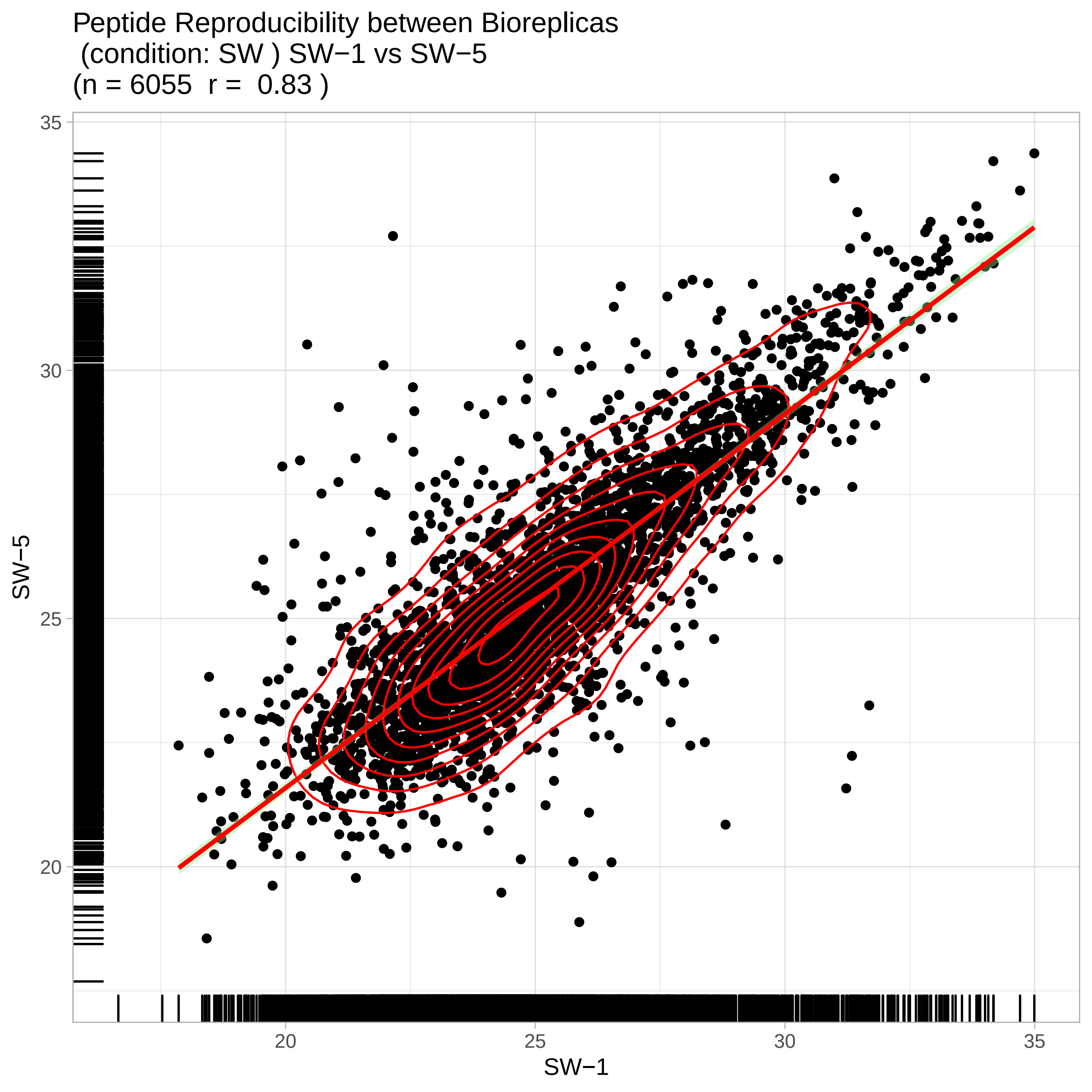

Peptide Reproducibility between Bioreplicas
(condition: SW) SW−1 vs SW−5
(n = 6055 r = 0.83)
35
30
25
20
SW−5
20
25
30
35
SW−1

Peptide Reproducibility between Bioreplicas
(condition: SW) SW−1 vs SW−6
(n = 6055 r = 0.79)

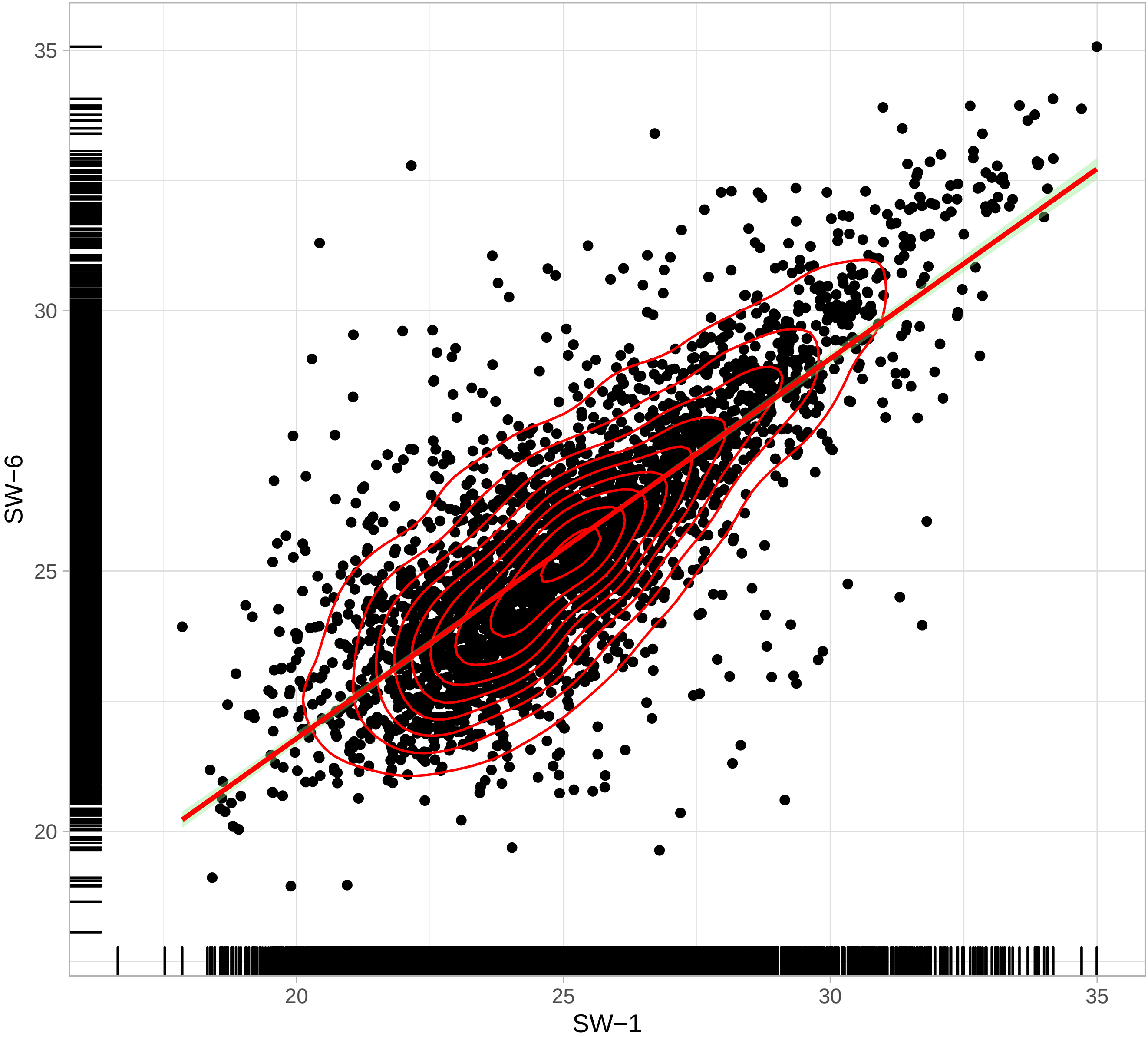

Peptide Reproducibility between Bioreplicas
(condition: SW) SW−1 vs SW−7
(n = 6055 r = 0.83)

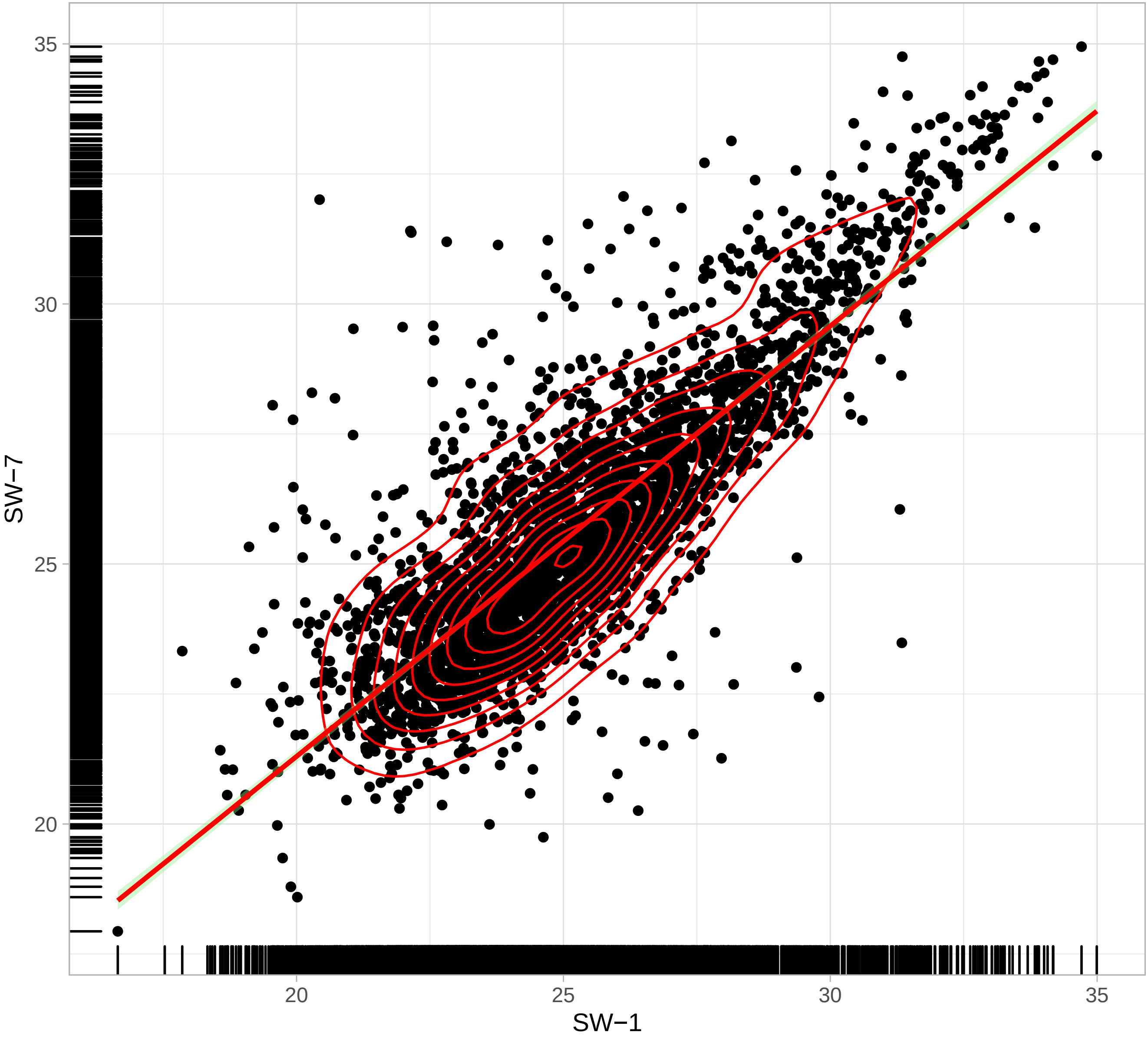

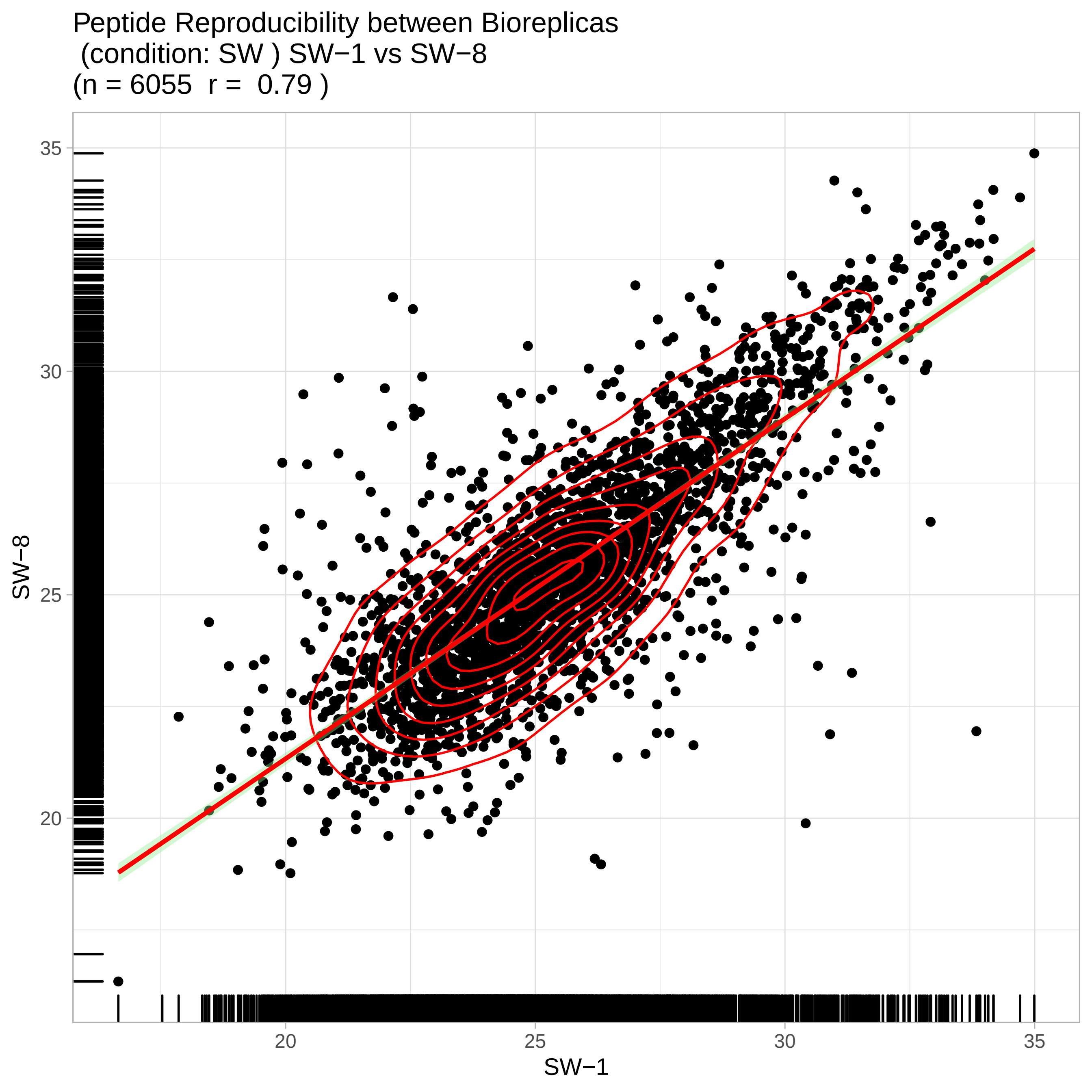
Peptide Reproducibility between Bioreplicas
(condition: SW) SW−1 vs SW−8
(n = 6055 r = 0.79)
SW−8
35
30
25
20
20
25
30
35
SW−1

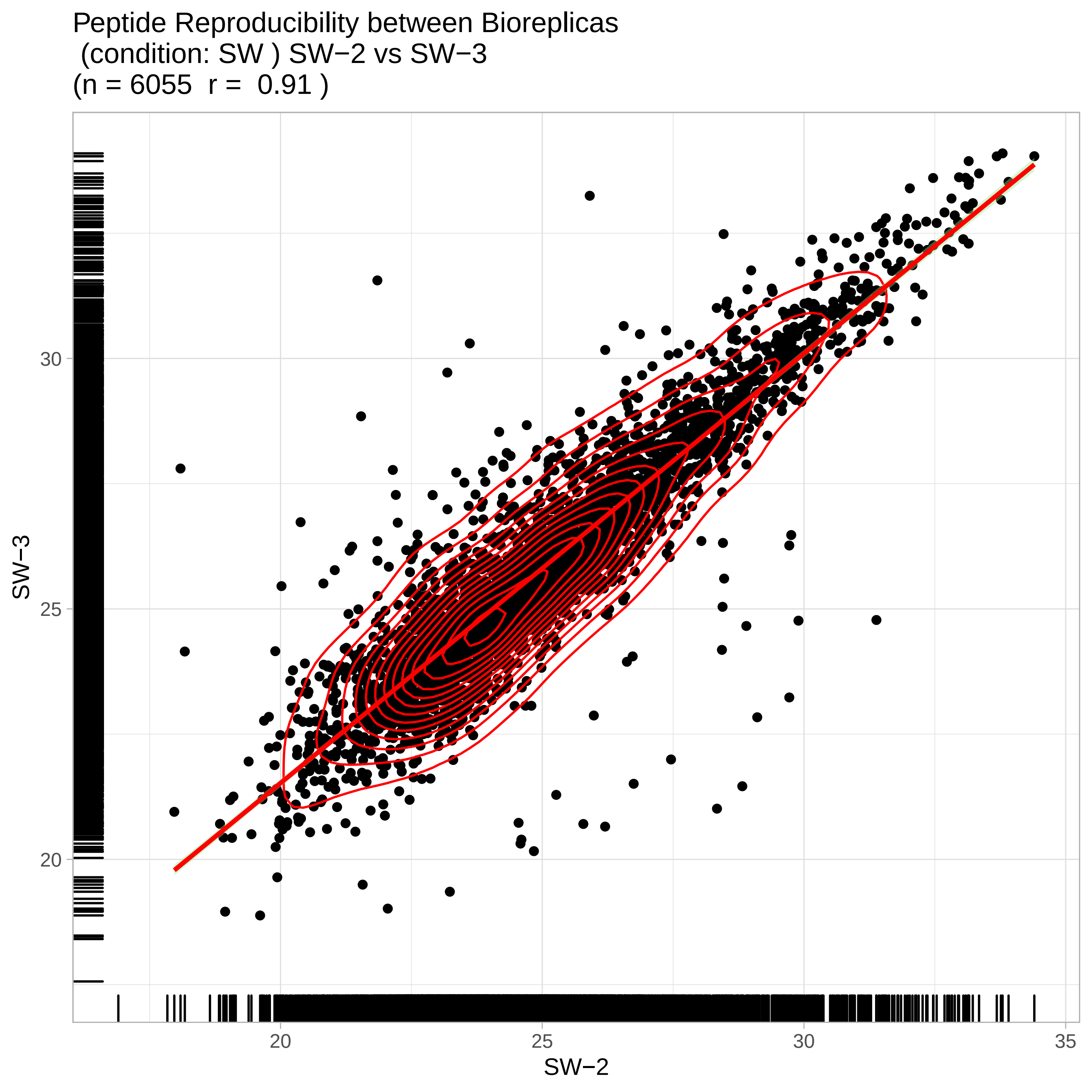

Peptide Reproducibility between Bioreplicas
(condition: SW) SW−2 vs SW−3
(n = 6055 r = 0.91)
SW−3
SW−2
20
25
30
35

Peptide Reproducibility between Bioreplicas
(condition: SW) SW−2 vs SW−4
(n = 6055 r = 0.85)

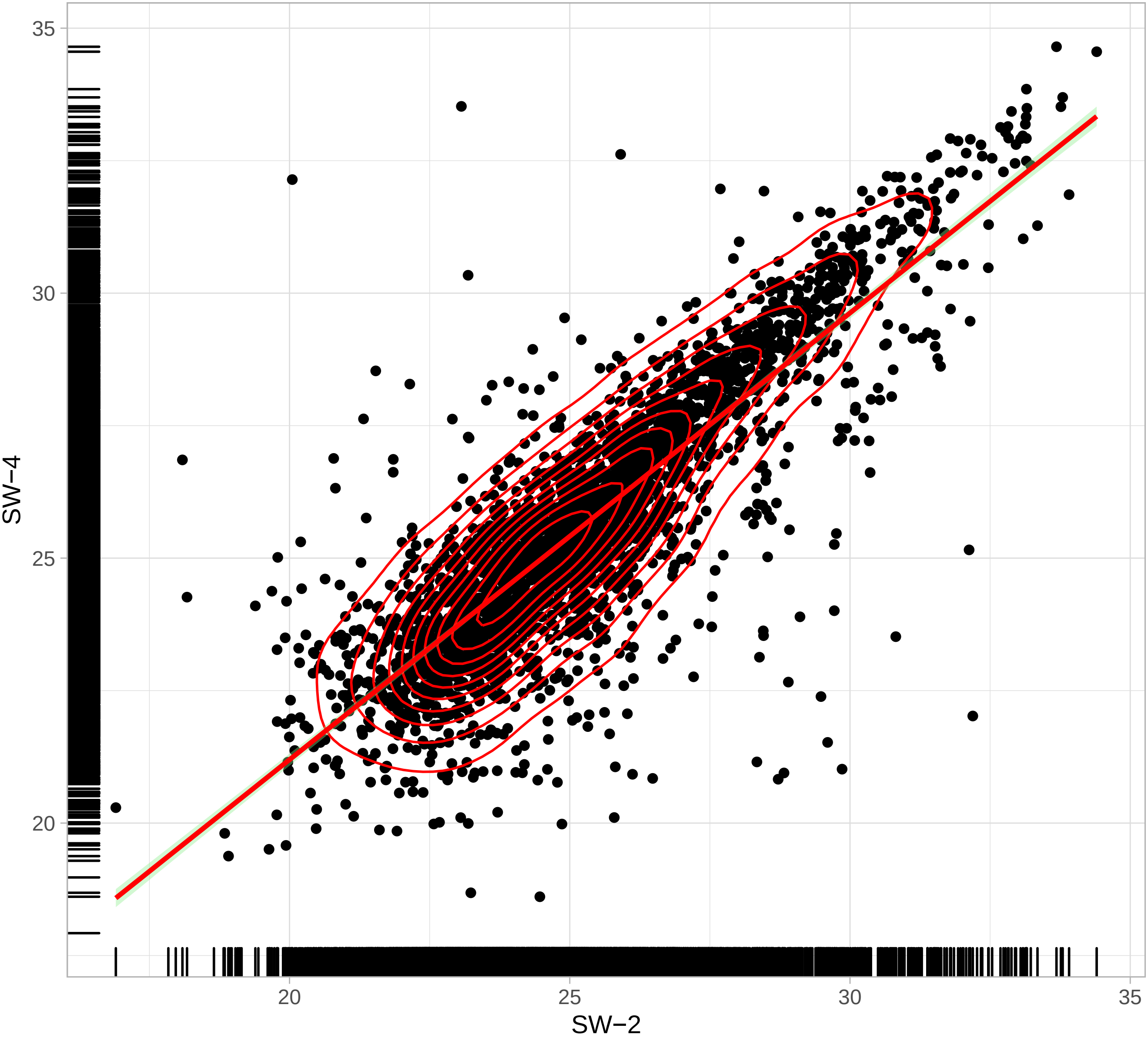

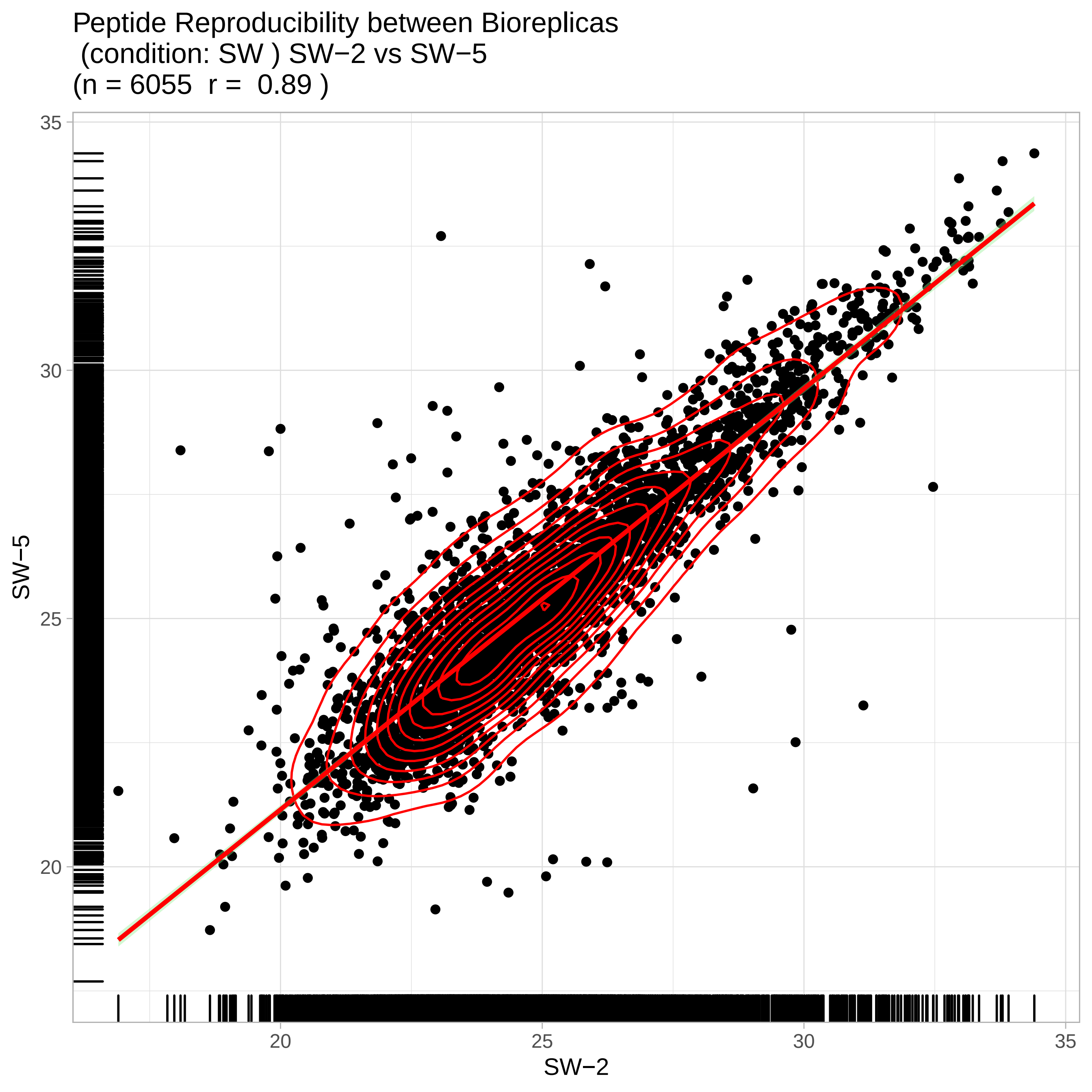
Peptide Reproducibility between Bioreplicas
(condition: SW) SW−2 vs SW−5
(n = 6055 r = 0.89)
35
30
25
20
SW−5
20
25
30
35
SW−2

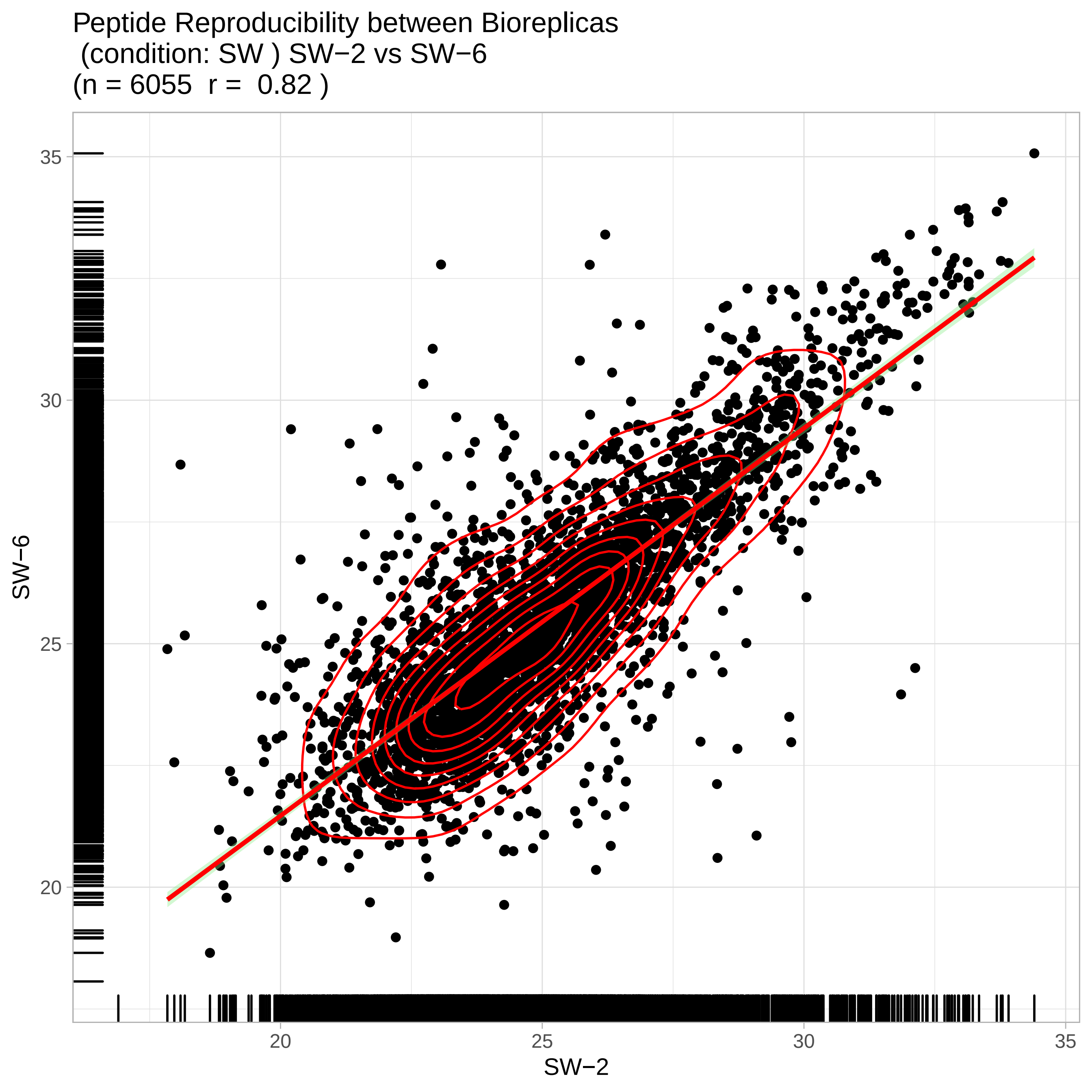
Peptide Reproducibility between Bioreplicas
(condition: SW) SW−2 vs SW−6
(n = 6055 r = 0.82)
SW−6
SW−2
35
30
25
20
20
25
30
35

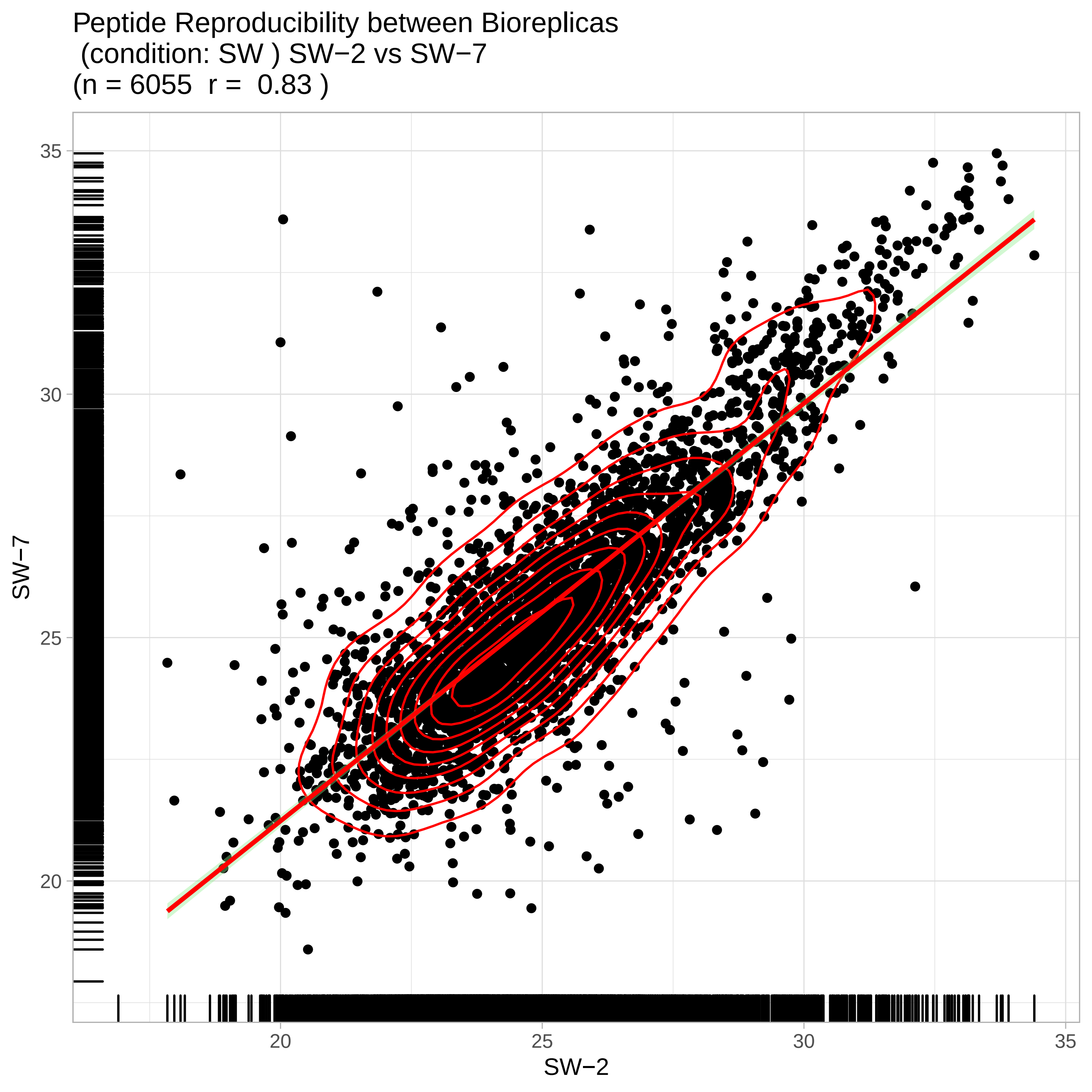
Peptide Reproducibility between Bioreplicas
(condition: SW) SW−2 vs SW−7
(n = 6055 r = 0.83)
SW−7
SW−2
35
30
25
20
20
25
30
35

Peptide Reproducibility between Bioreplicas
(condition: SW) SW−2 vs SW−8
(n = 6055 r = 0.85)

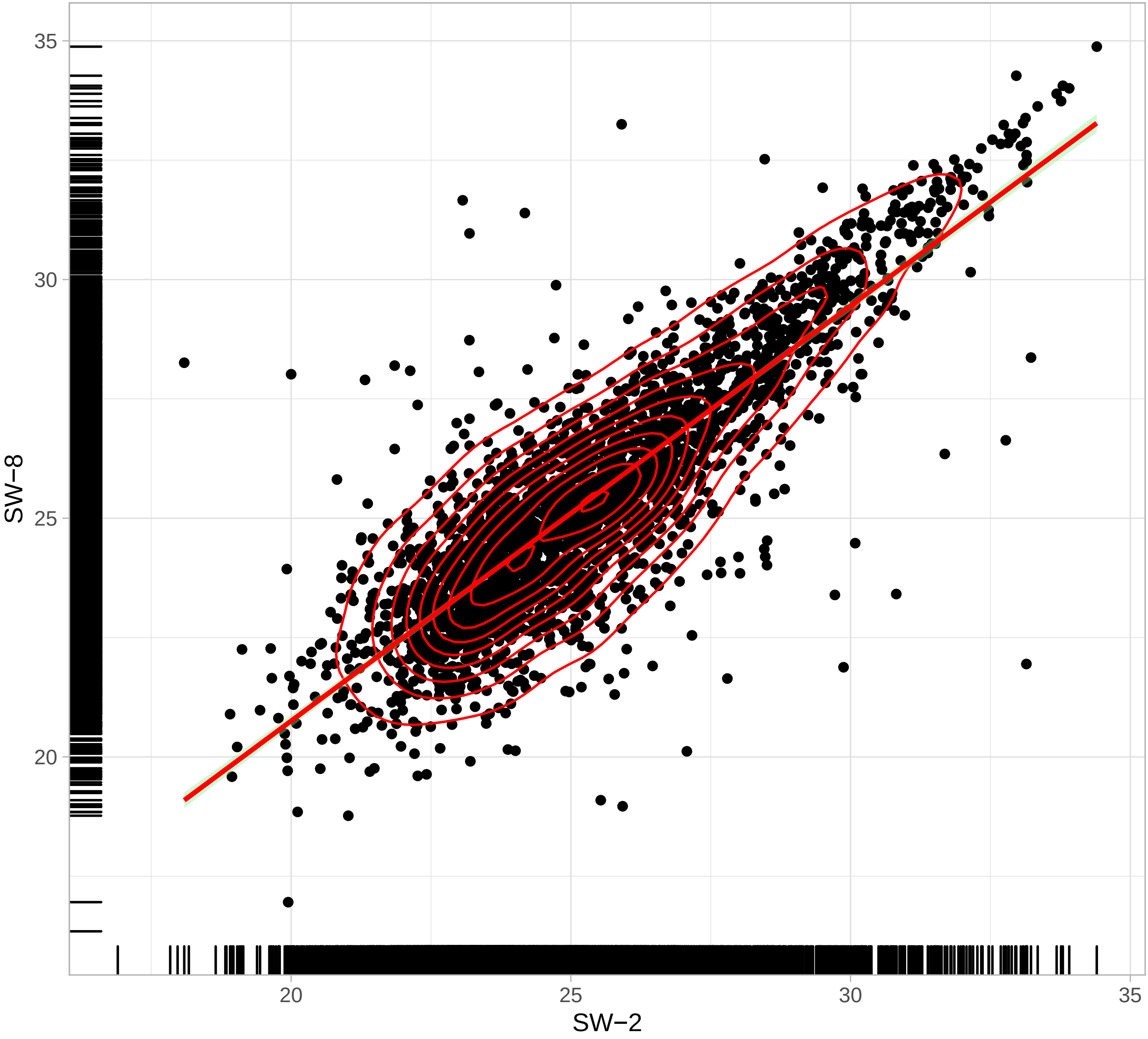

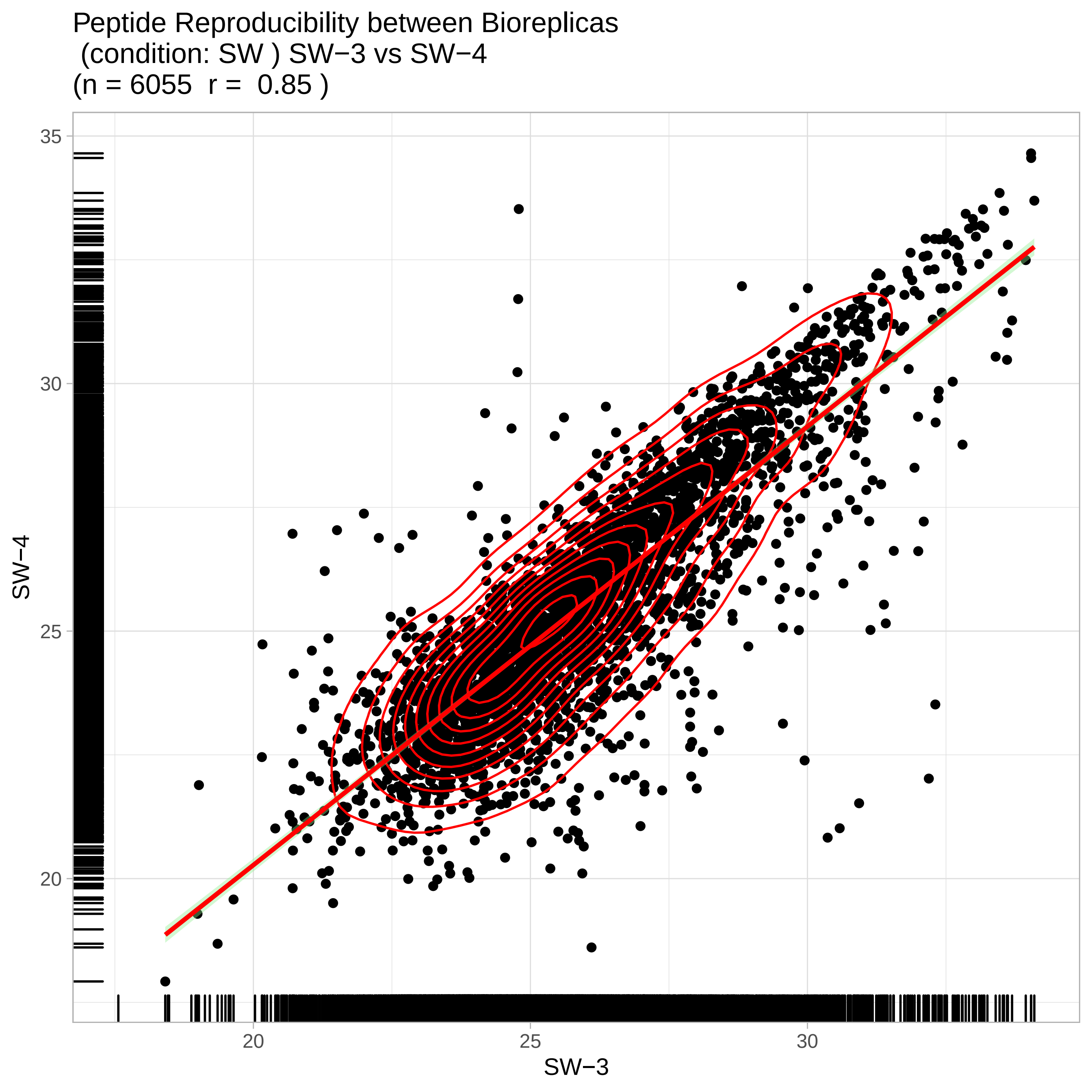

Peptide Reproducibility between Bioreplicas
(condition: SW) SW−3 vs SW−4
(n = 6055 r = 0.85)
35
30
25
20
SW−4
20
25
30
SW−3

Peptide Reproducibility between Bioreplicas
(condition: SW) SW−3 vs SW−5
(n = 6055 r = 0.91)

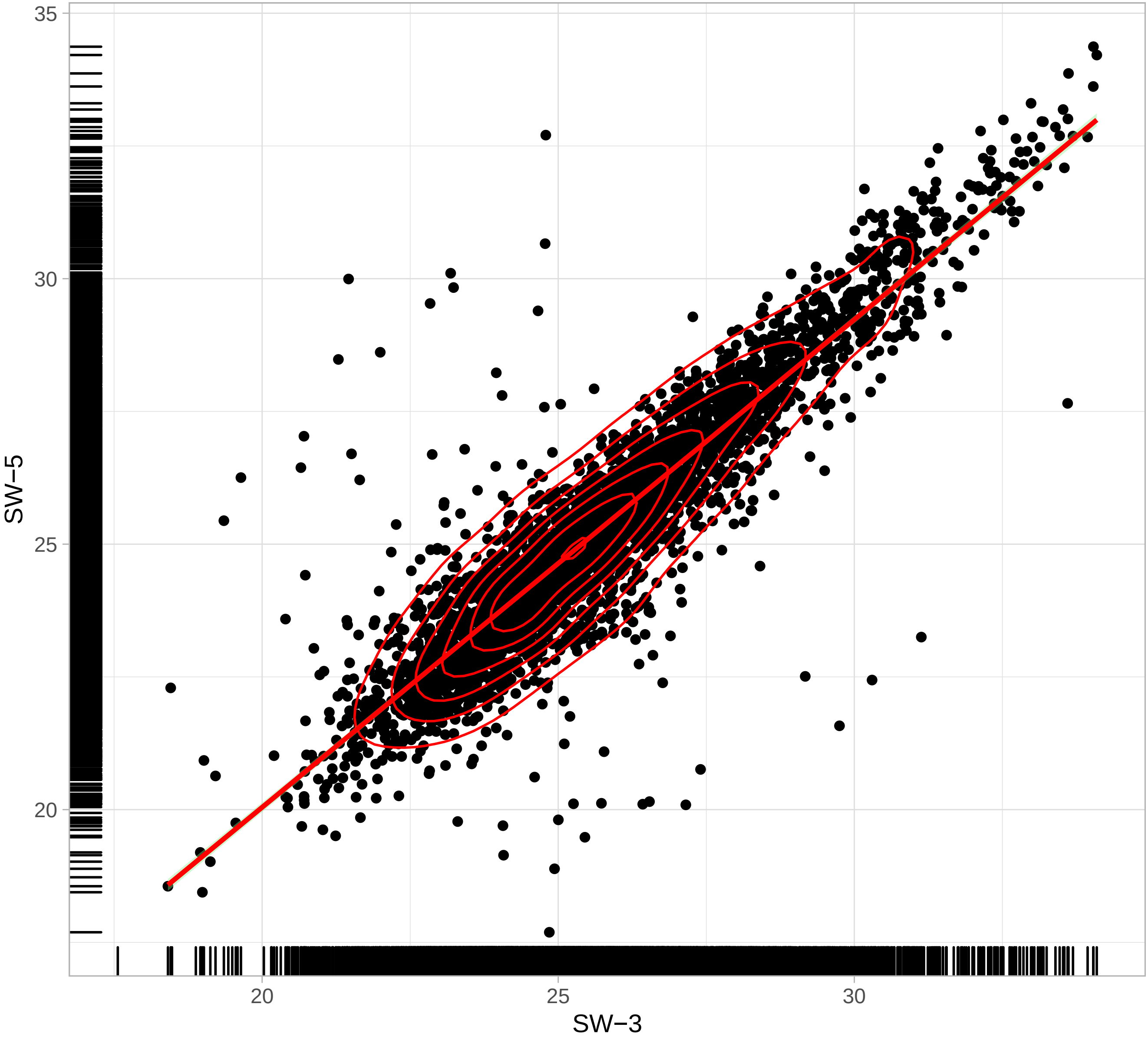

Peptide Reproducibility between Bioreplicas
(condition: SW) SW−3 vs SW−6
(n = 6055 r = 0.85)

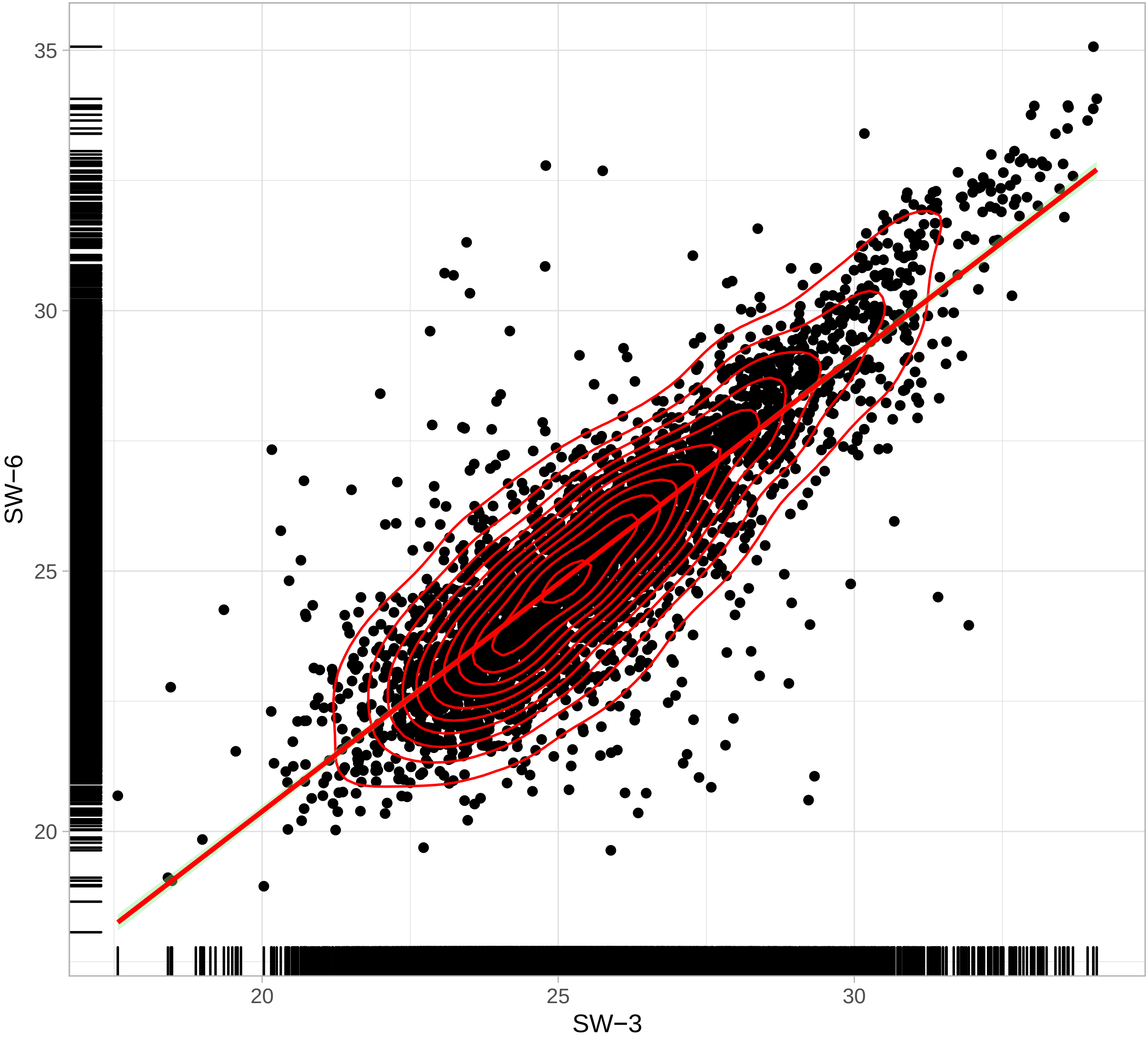

Peptide Reproducibility between Bioreplicas
(condition: SW) SW−3 vs SW−7
(n = 6055 r = 0.89)

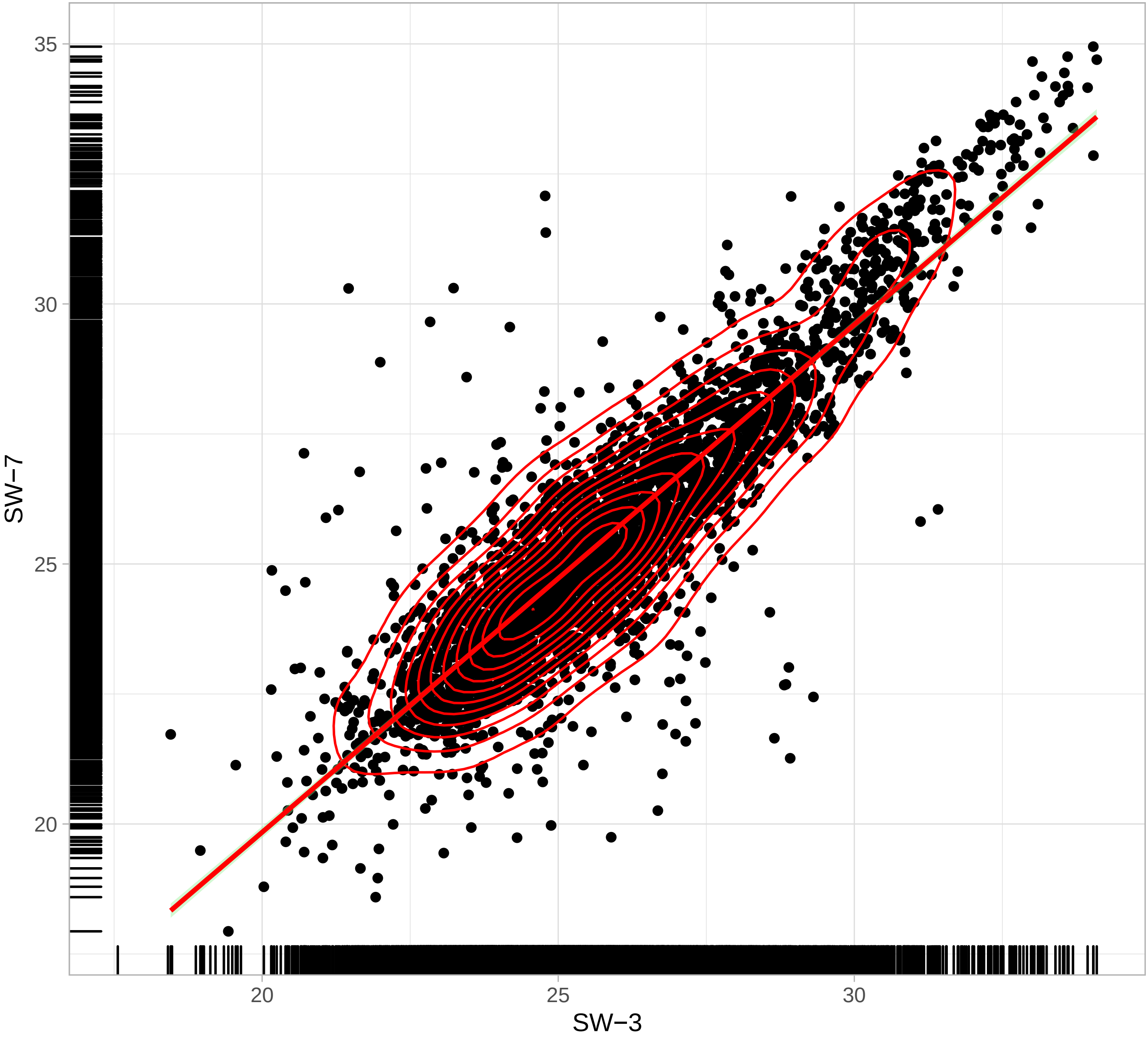

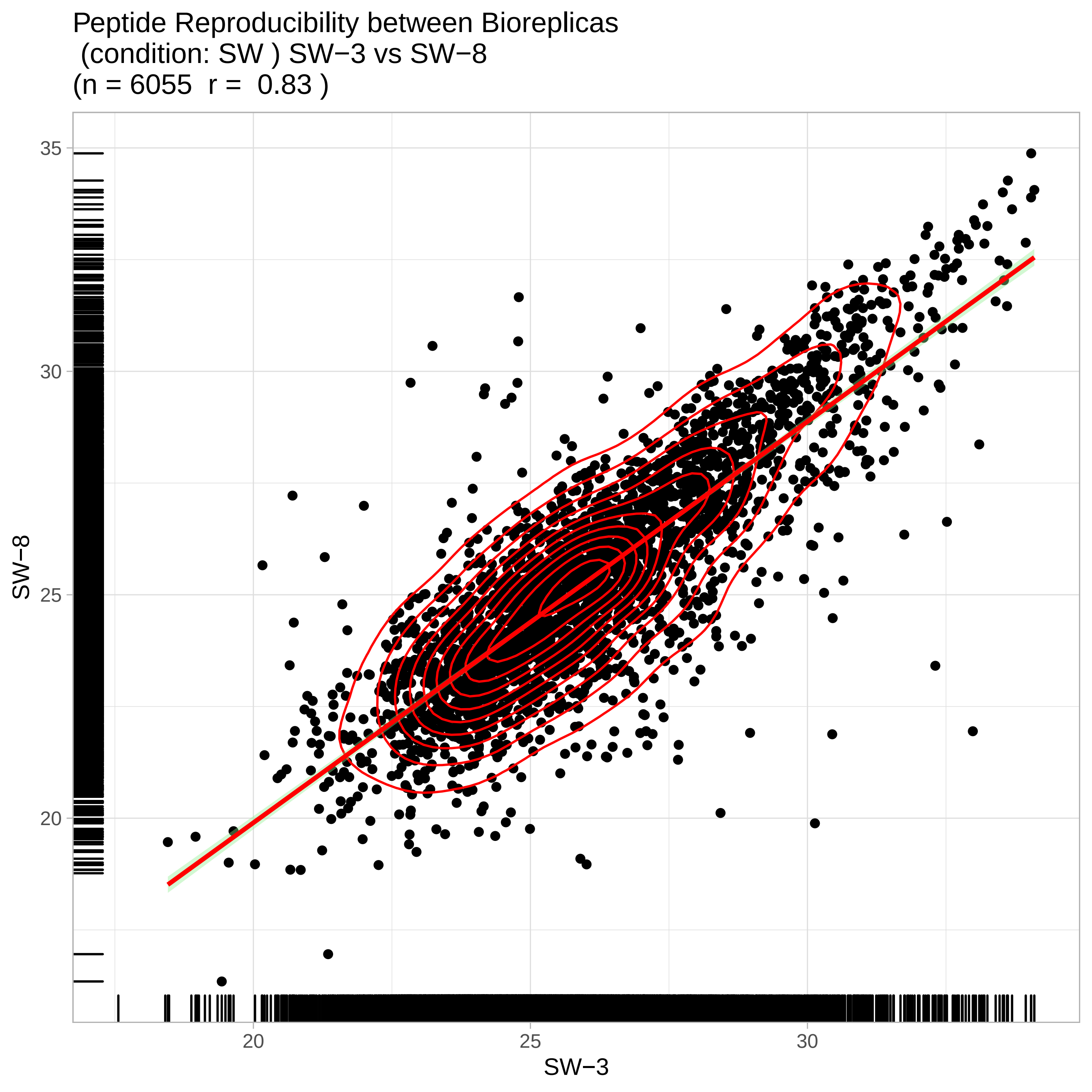

Peptide Reproducibility between Bioreplicas
(condition: SW) SW−3 vs SW−8
(n = 6055 r = 0.83)
35
30
25
20
SW−8
20
25
30
SW−3

Peptide Reproducibility between Bioreplicas
(condition: SW) SW−4 vs SW−5
(n = 6055 r = 0.83)

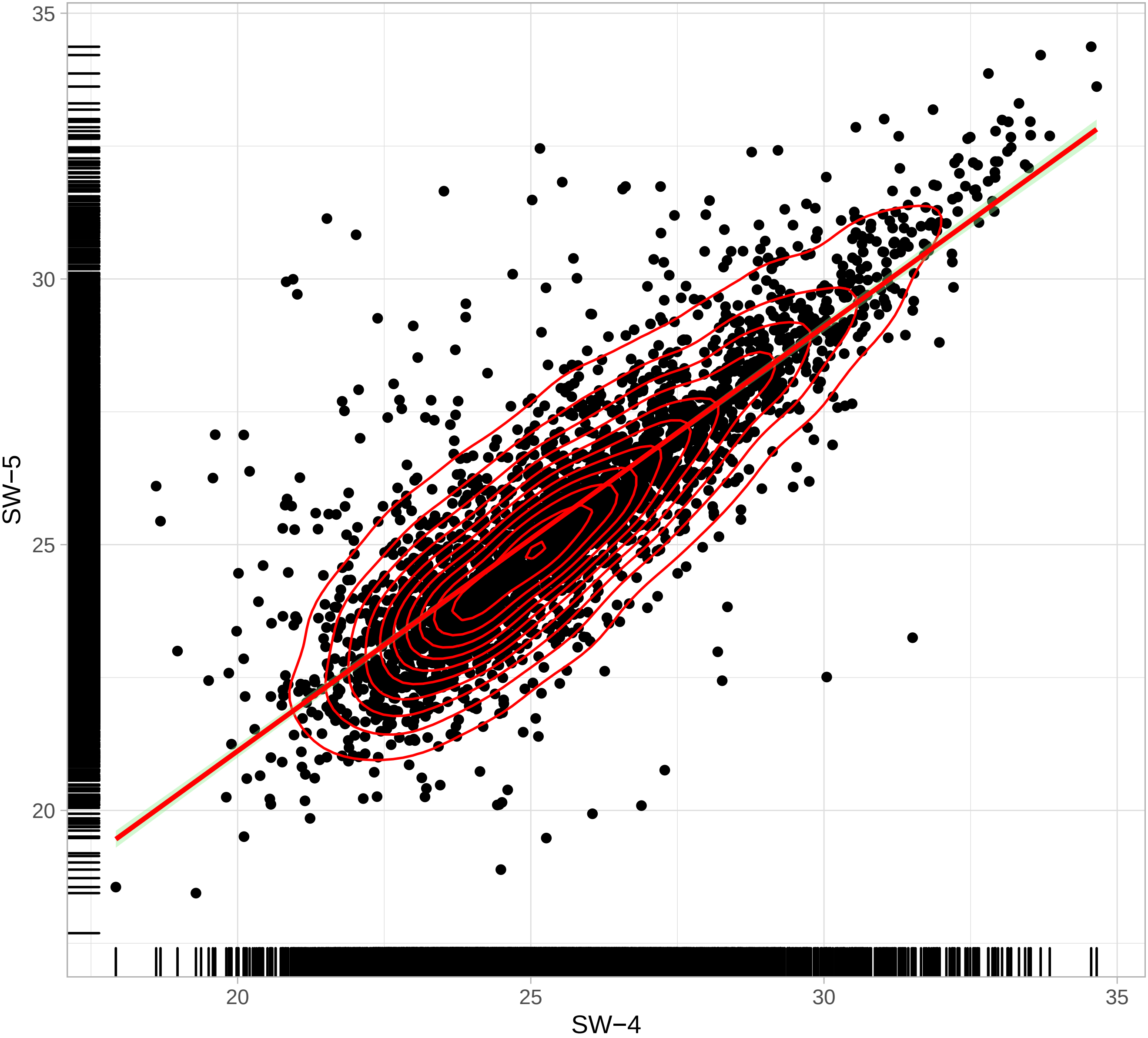

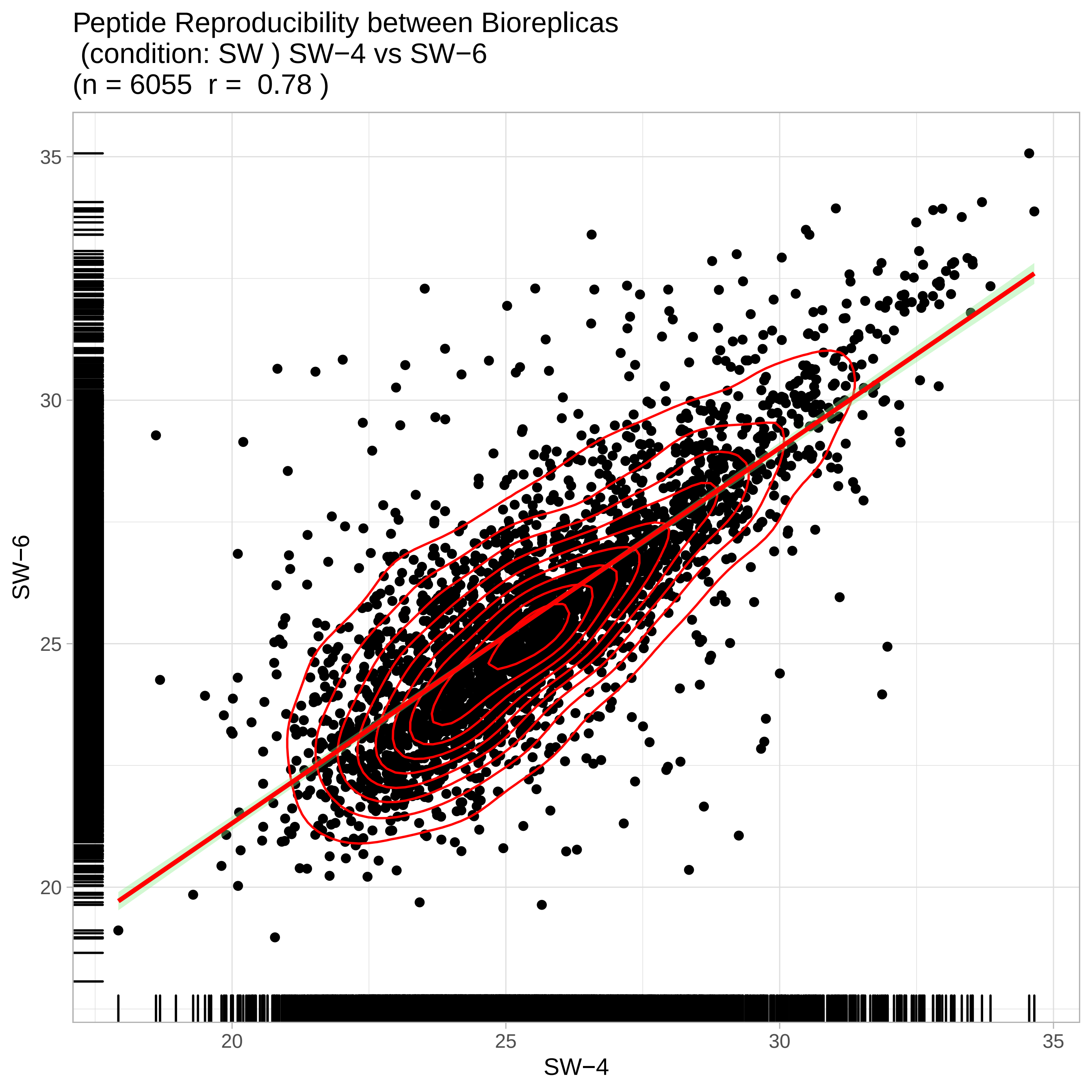

Peptide Reproducibility between Bioreplicas
(condition: SW) SW−4 vs SW−6
(n = 6055 r = 0.78)
SW−6
SW−4
35
30
25
20
20
25
30
35

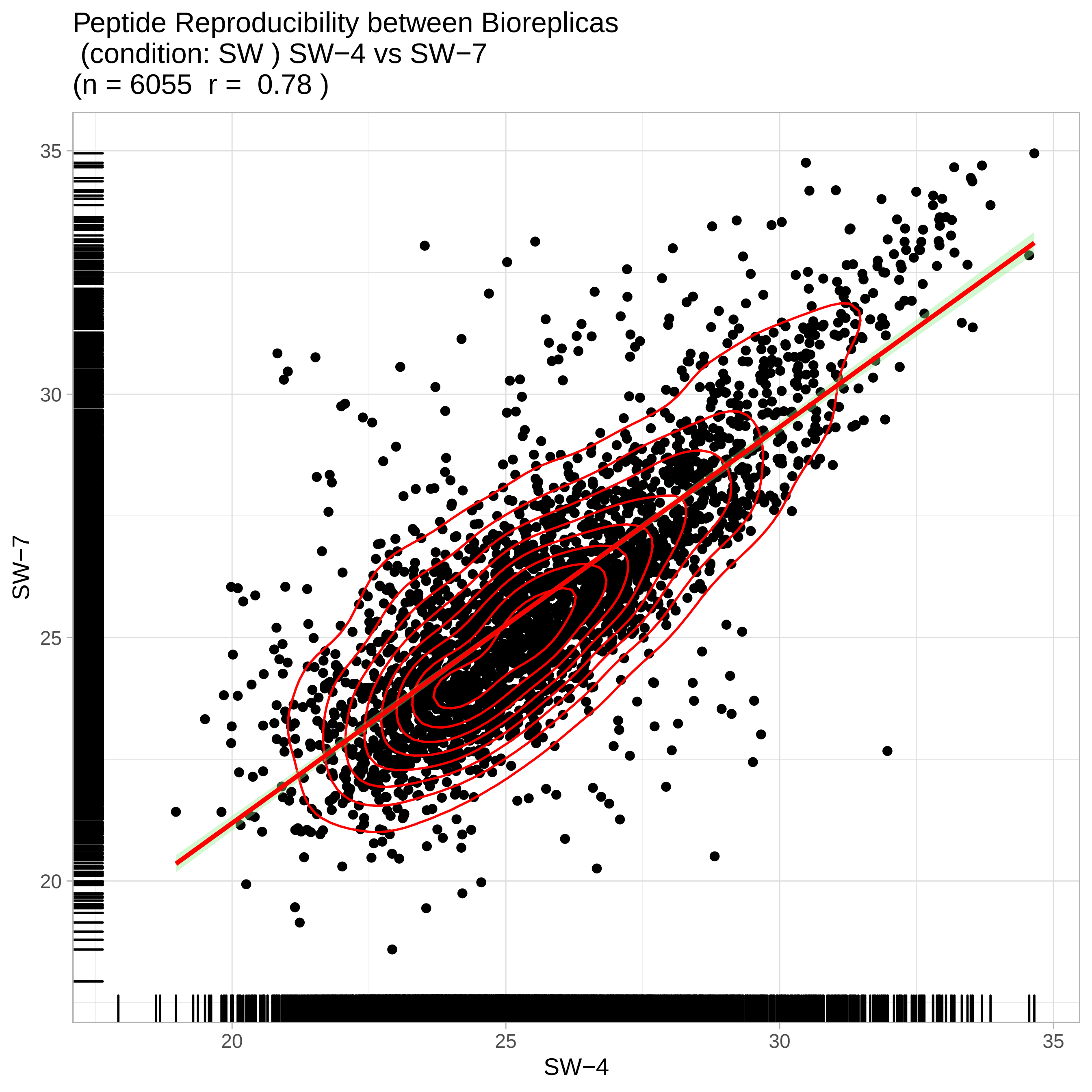
Peptide Reproducibility between Bioreplicas
(condition: SW) SW−4 vs SW−7
(n = 6055 r = 0.78)
SW−7
SW−4
35
30
25
20
20
25
30
35

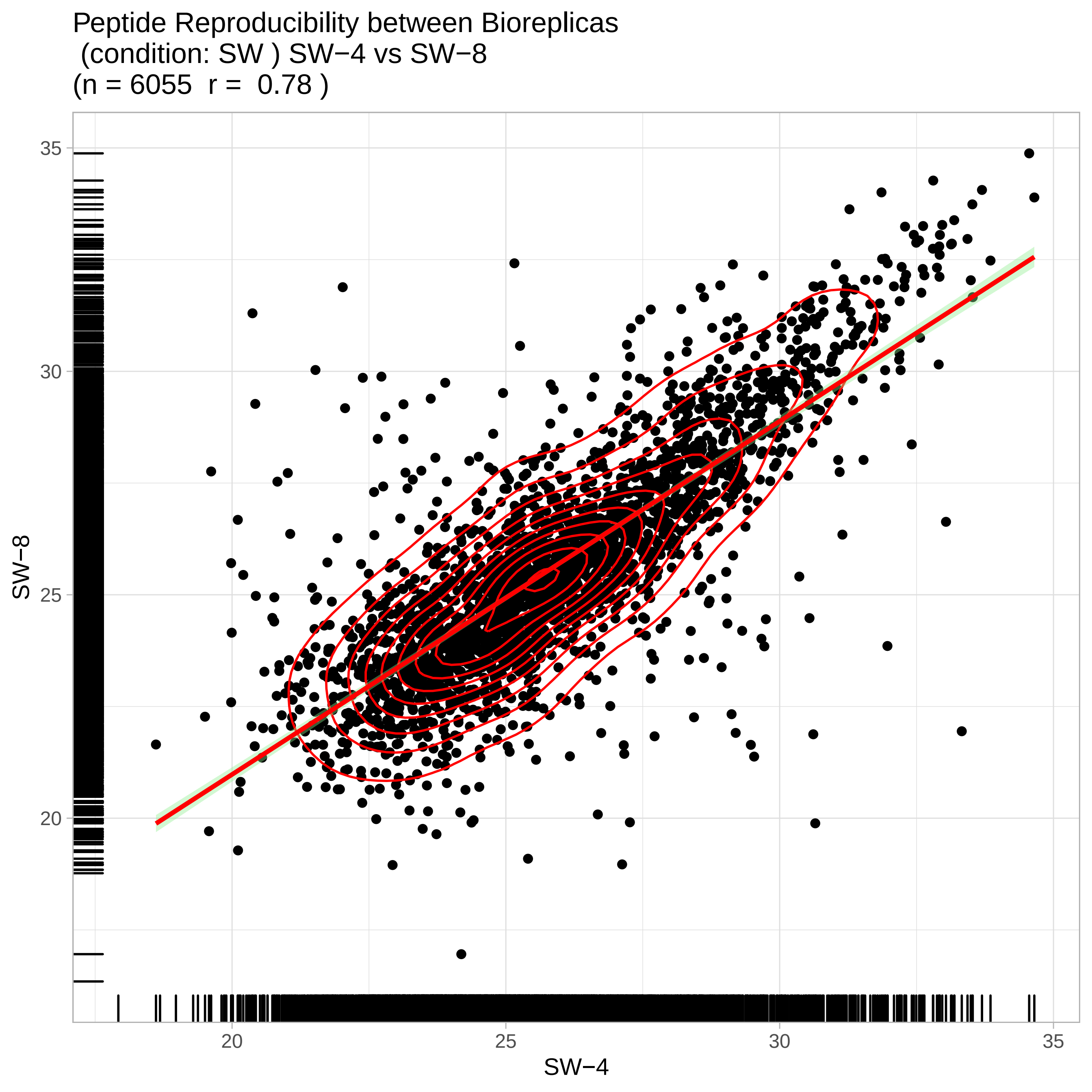
Peptide Reproducibility between Bioreplicas
(condition: SW) SW−4 vs SW−8
(n = 6055 r = 0.78)
SW−8
35
30
25
20
20
25
30
35
SW−4

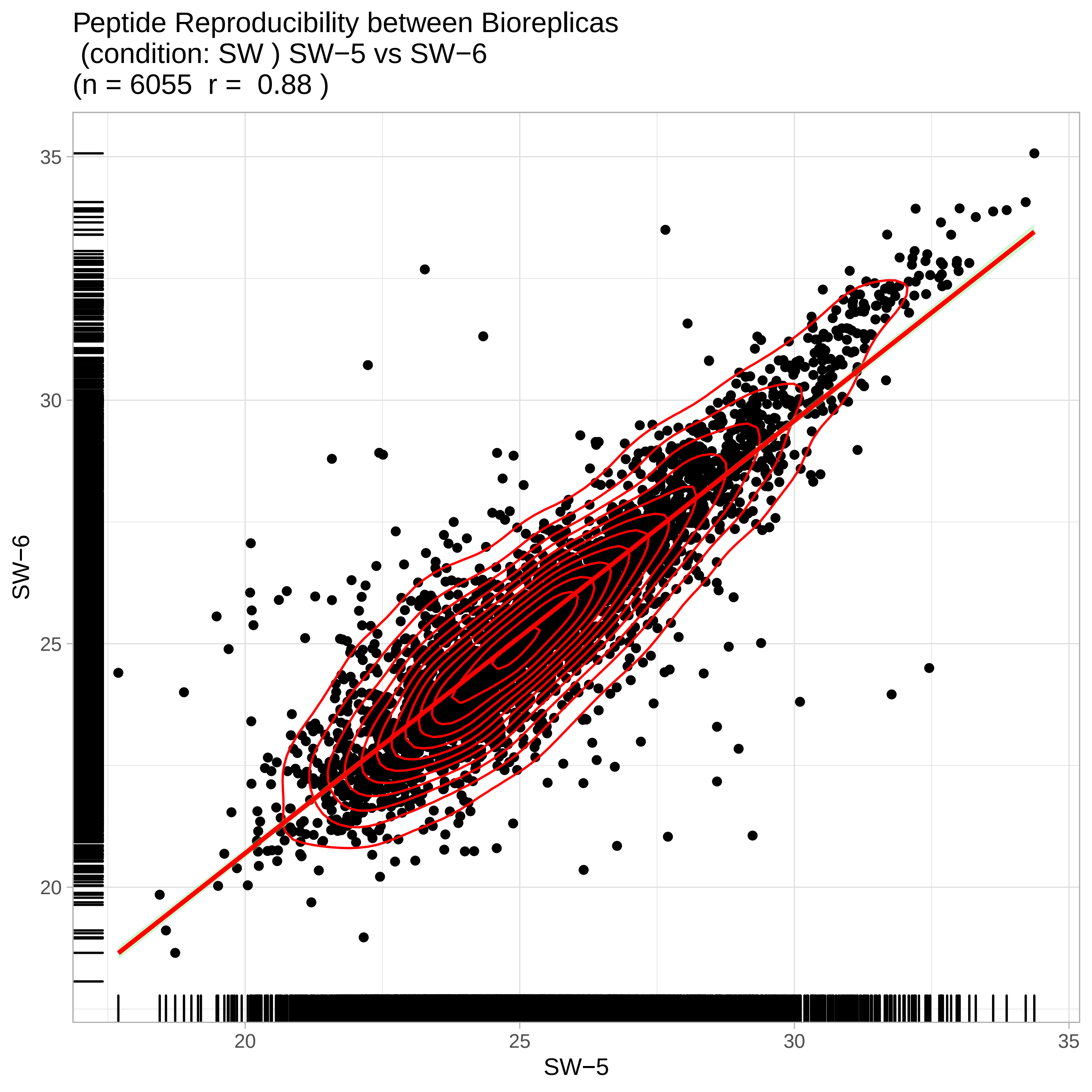

Peptide Reproducibility between Bioreplicas
(condition: SW) SW−5 vs SW−6
(n = 6055 r = 0.88)
35
30
25
20
SW−6
20
25
30
35
SW−5

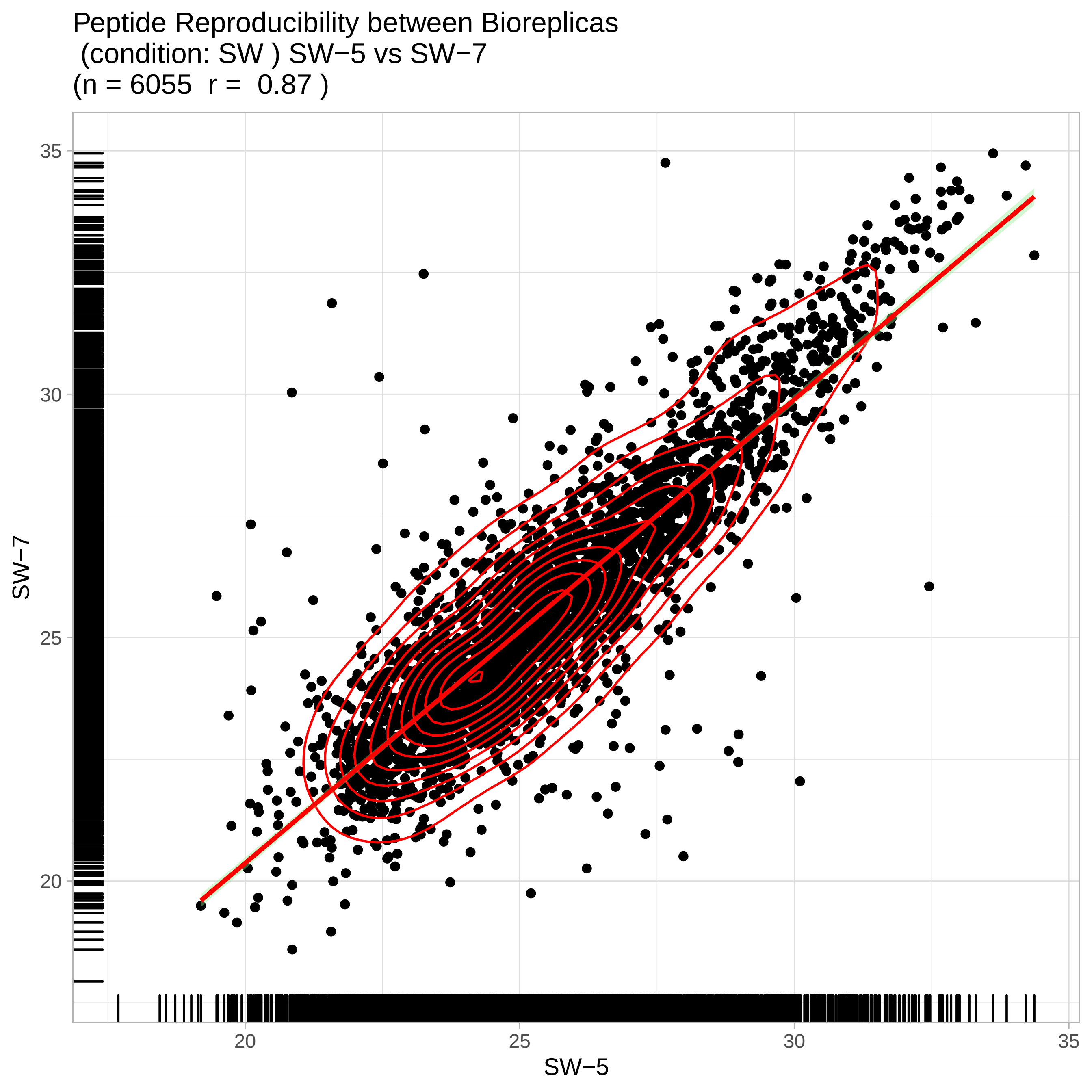
Peptide Reproducibility between Bioreplicas
(condition: SW) SW−5 vs SW−7
(n = 6055 r = 0.87)
35
30
25
20
SW−7
20
25
30
35
SW−5

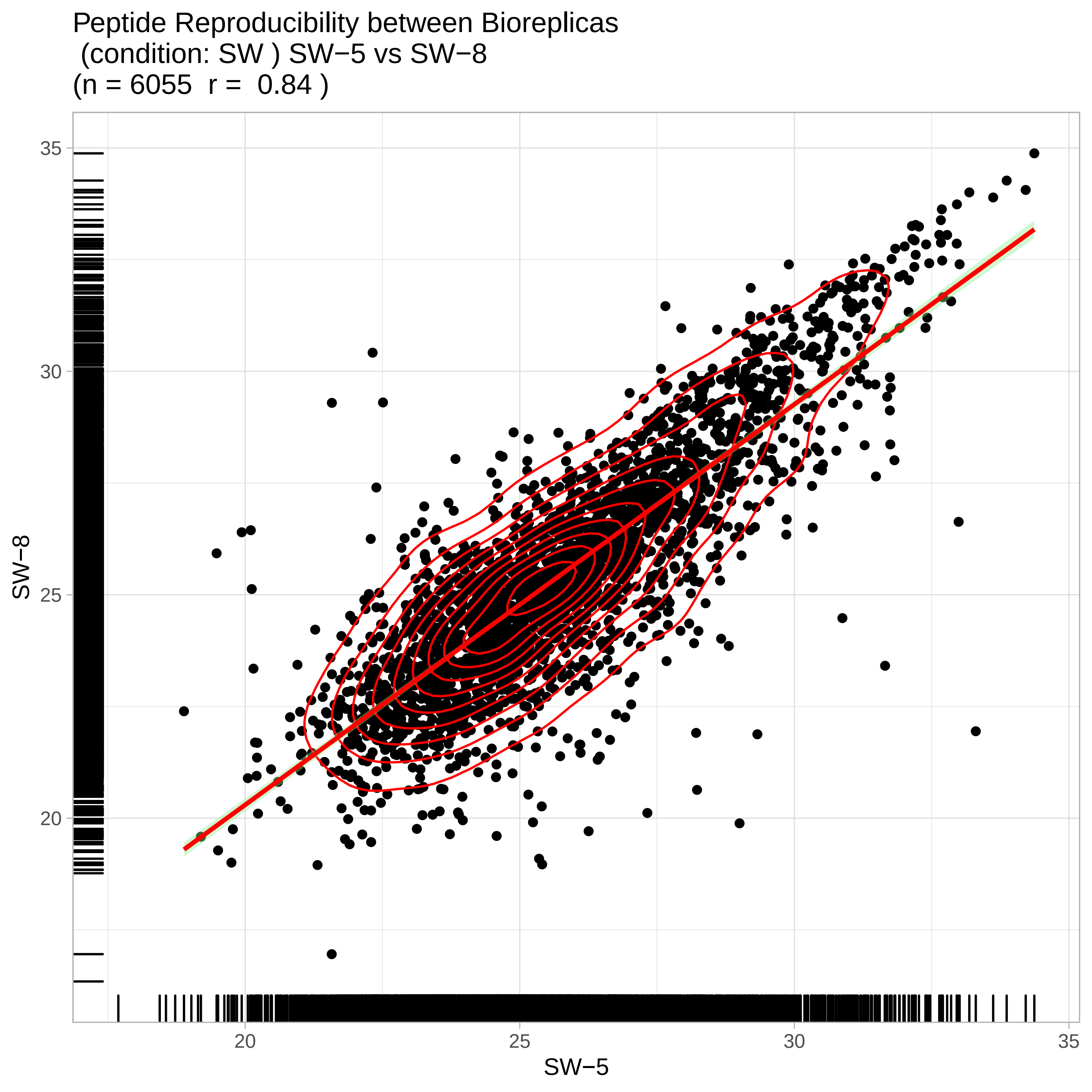

Peptide Reproducibility between Bioreplicas
(condition: SW) SW−5 vs SW−8
(n = 6055 r = 0.84)
SW−8
SW−5
35
30
25
20
20
25
30
35

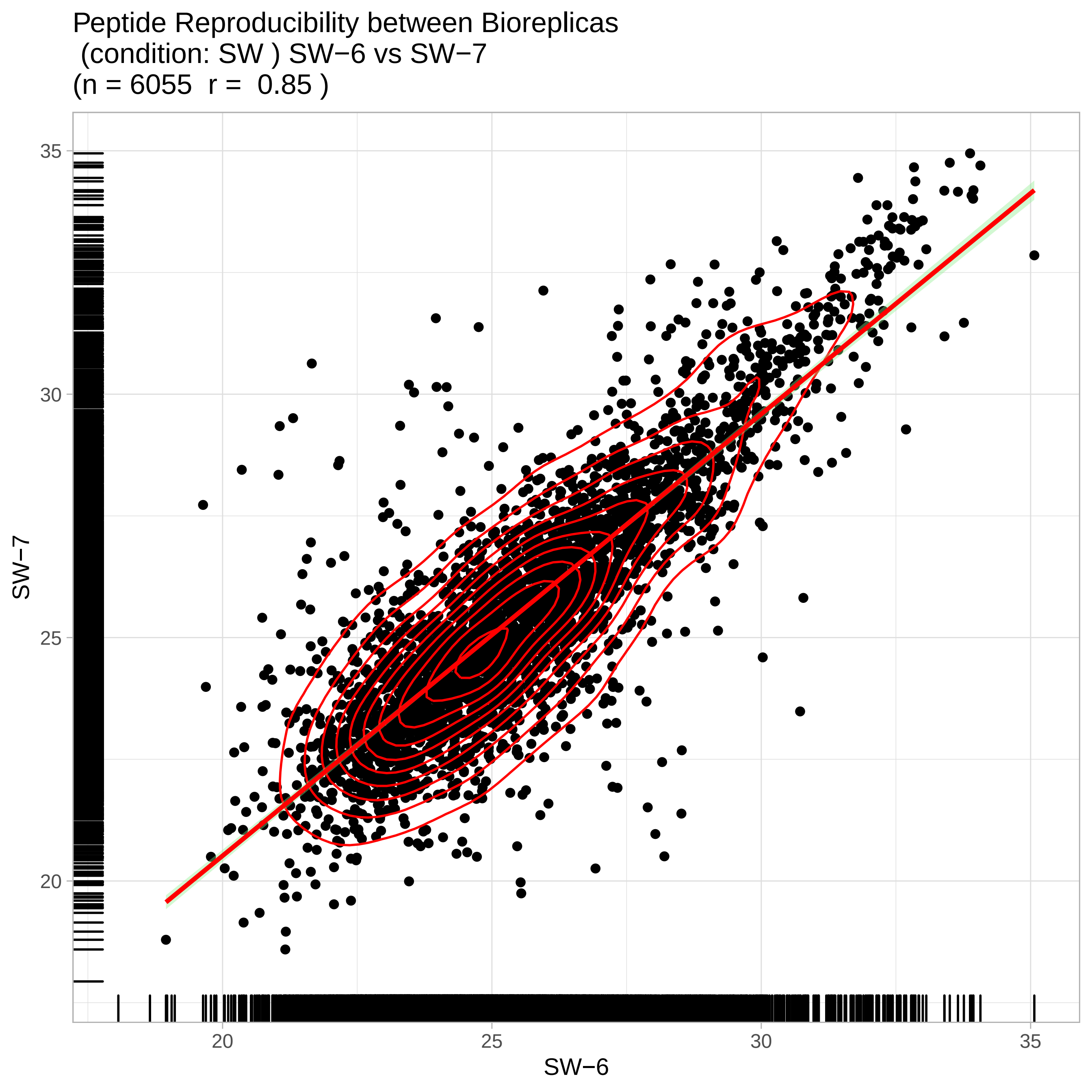
Peptide Reproducibility between Bioreplicas
(condition: SW) SW−6 vs SW−7
(n = 6055 r = 0.85)
SW−7
SW−6
20
25
30
35
20
25
30
35

Peptide Reproducibility between Bioreplicas
(condition: SW) SW−6 vs SW−8
(n = 6055 r = 0.84)

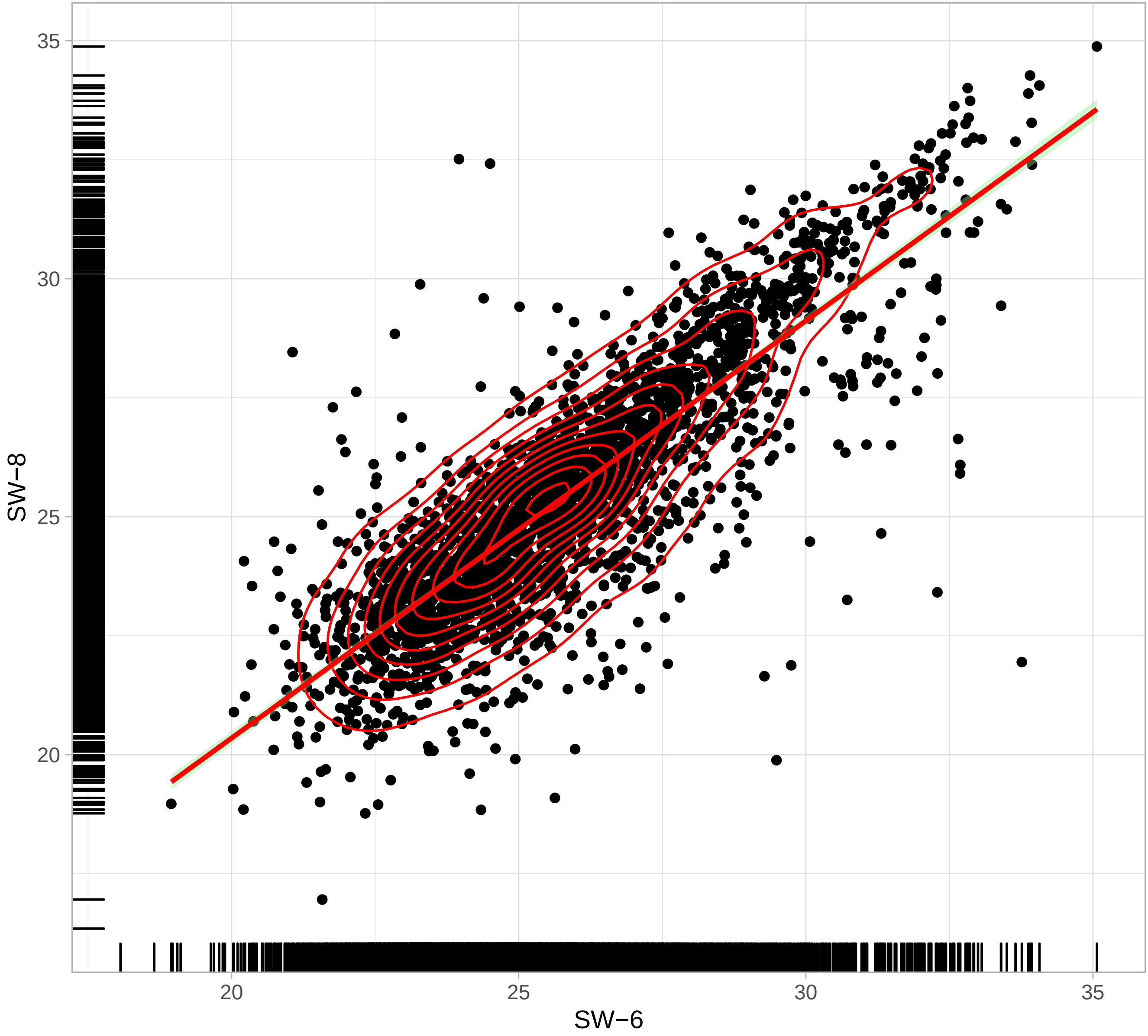

Peptide Reproducibility between Bioreplicas
(condition: SW) SW−7 vs SW−8
(n = 6055 r = 0.82)

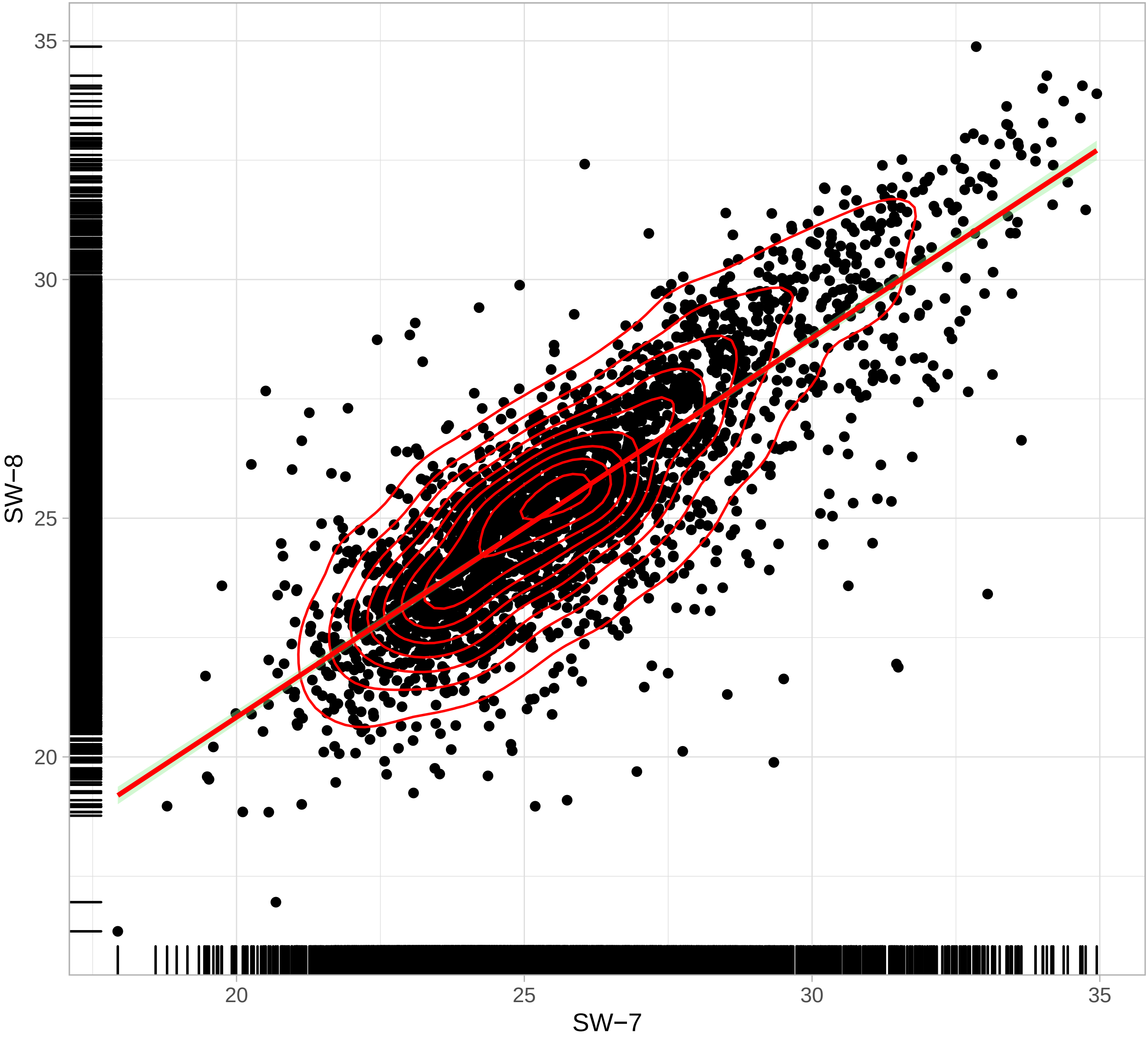

Supplement: Supplementary file 2 — Supplementary Information 2. [file 41598_2020_72569_MOESM2_ESM.zip › SI3_artMS_QC/qcPlots_evidence.qcplot.basicReproducibility.pdf]
