# Supplementary material for: Abiotic and past climatic conditions drive protein abundance variation among natural populations of the caddisfly Crunoecia irrorata
Source: Sci Rep. 2020 Sep 23;10:15538. doi: 10.1038/s41598-020-72569-4 (PMC7512004; doi:10.1038/s41598-020-72569-4)
Supplement: Supplementary file 2 — Supplementary Information 2. [file 41598_2020_72569_MOESM2_ESM.zip › SI3_artMS_QC/qcPlots_evidence.qcplot.intensityStats.pdf]

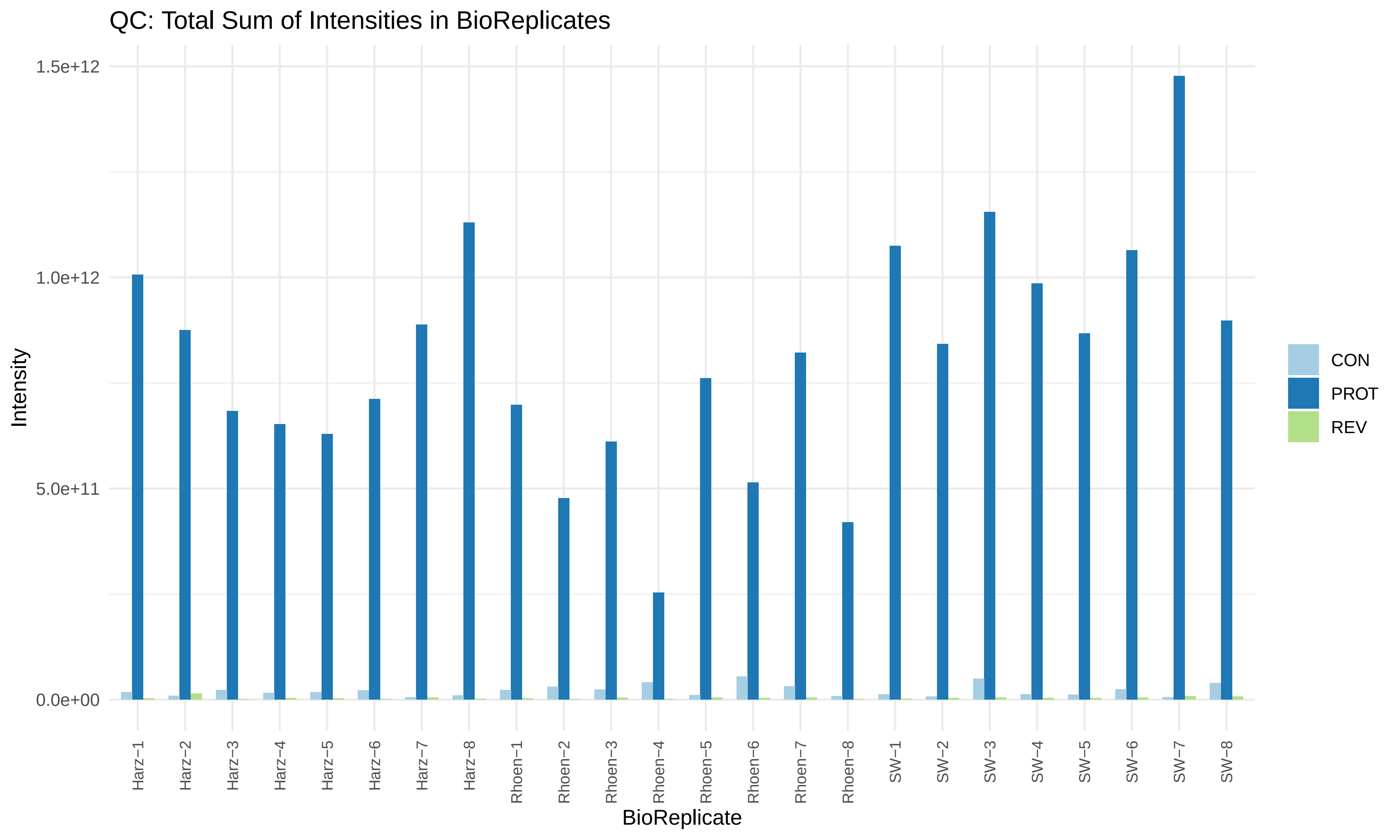
QC: Total Sum of Intensities in BioReplicates
Intensity
1.5e+12
1.0e+12
5.0e+11
0.0e+00
BioReplicate
Harz−1
Harz−2
Harz−3
Harz−4
Harz−5
Harz−6
Harz−7
Harz−8
Rhoen−1
Rhoen−2
Rhoen−3
Rhoen−4
Rhoen−5
Rhoen−6
Rhoen−7
Rhoen−8
SW−1
SW−2
SW−3
SW−4
SW−5
SW−6
SW−7
SW−8
CON
PROT
REV

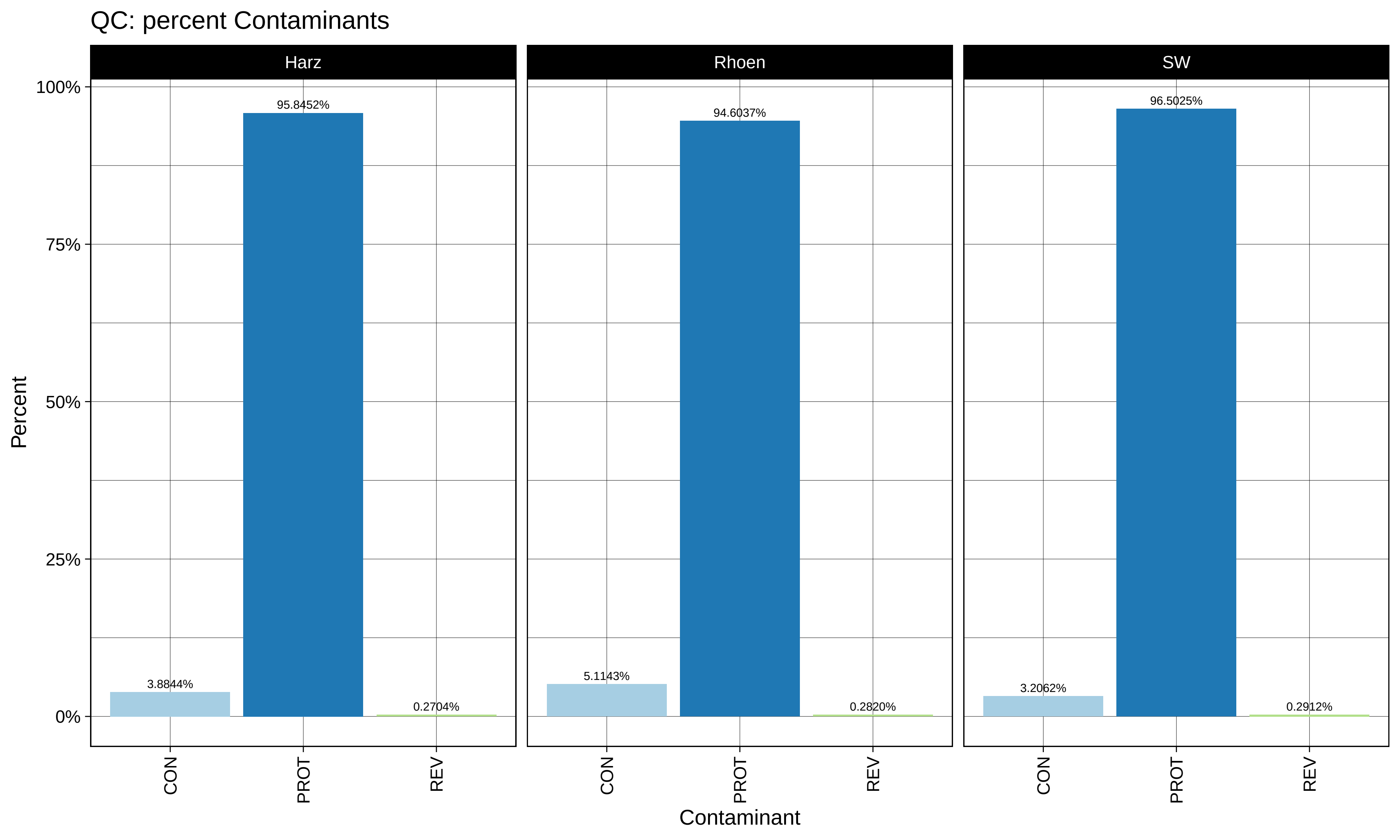

QC: percent Contaminants
Harz
Rhoen
SW
100%
75%
50%
25%
0%
Percent
95.8452%
3.8844%
0.2704%
94.6037%
5.1143%
0.2820%
96.5025%
3.2062%
0.2912%
CON
PROT
REV
Contaminant

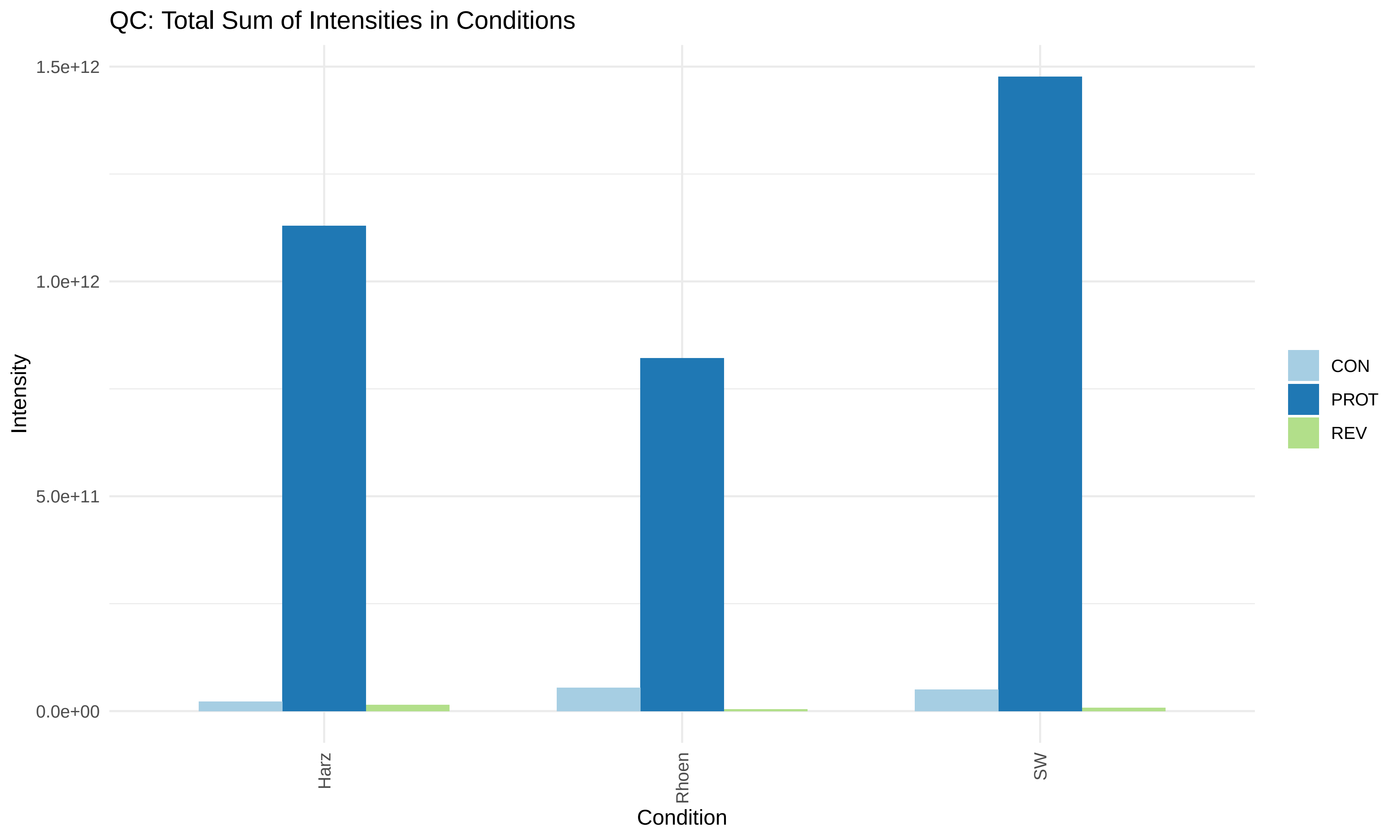
QC: Total Sum of Intensities in Conditions
Intensity
1.5e+12
1.0e+12
5.0e+11
0.0e+00
Harz
Rhoen
SW
Condition
CON
PROT
REV

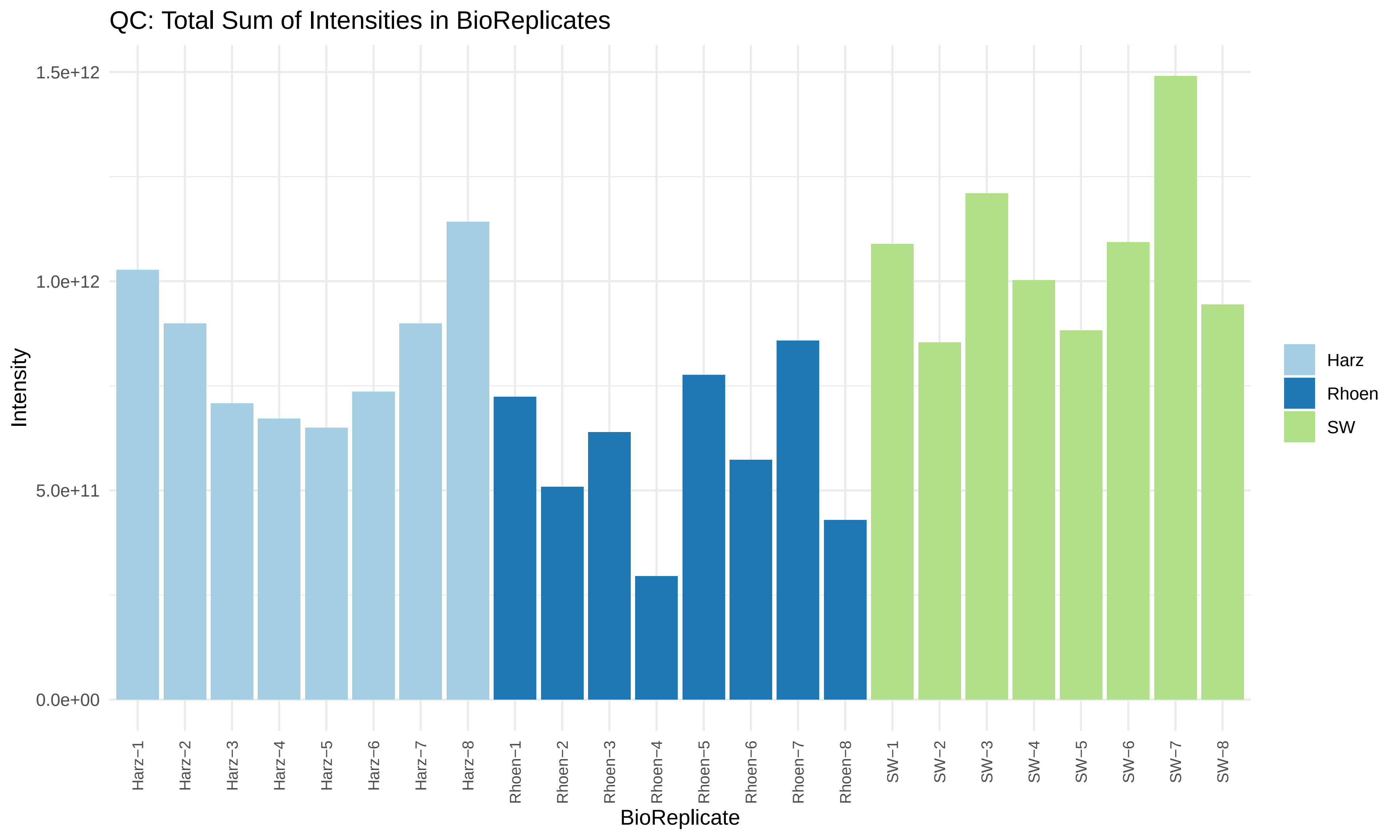
QC: Total Sum of Intensities in BioReplicates
Intensity
1.5e+12
1.0e+12
5.0e+11
0.0e+00
Harz−1
Harz−2
Harz−3
Harz−4
Harz−5
Harz−6
Harz−7
Harz−8
Rhoen−1
Rhoen−2
Rhoen−3
Rhoen−4
Rhoen−5
Rhoen−6
Rhoen−7
Rhoen−8
SW−1
SW−2
SW−3
SW−4
SW−5
SW−6
SW−7
SW−8
BioReplicate
Harz
Rhoen
SW

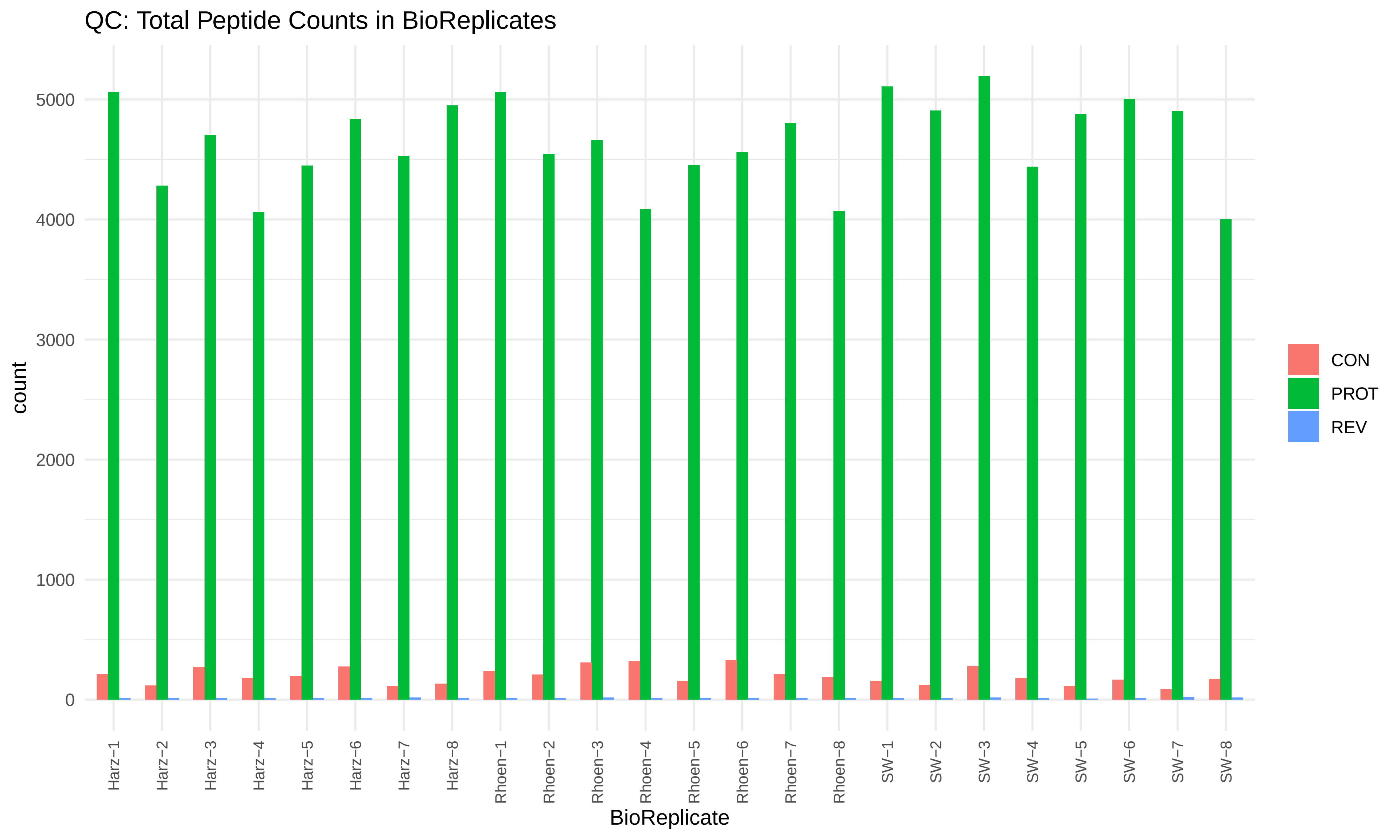
QC: Total Peptide Counts in BioReplicates
count
5000
4000
3000
2000
1000
0
Harz−1
Harz−2
Harz−3
Harz−4
Harz−5
Harz−6
Harz−7
Harz−8
Rhoen−1
Rhoen−2
Rhoen−3
Rhoen−4
Rhoen−5
Rhoen−6
Rhoen−7
Rhoen−8
SW−1
SW−2
SW−3
SW−4
SW−5
SW−6
SW−7
SW−8
BioReplicate
CON
PROT
REV

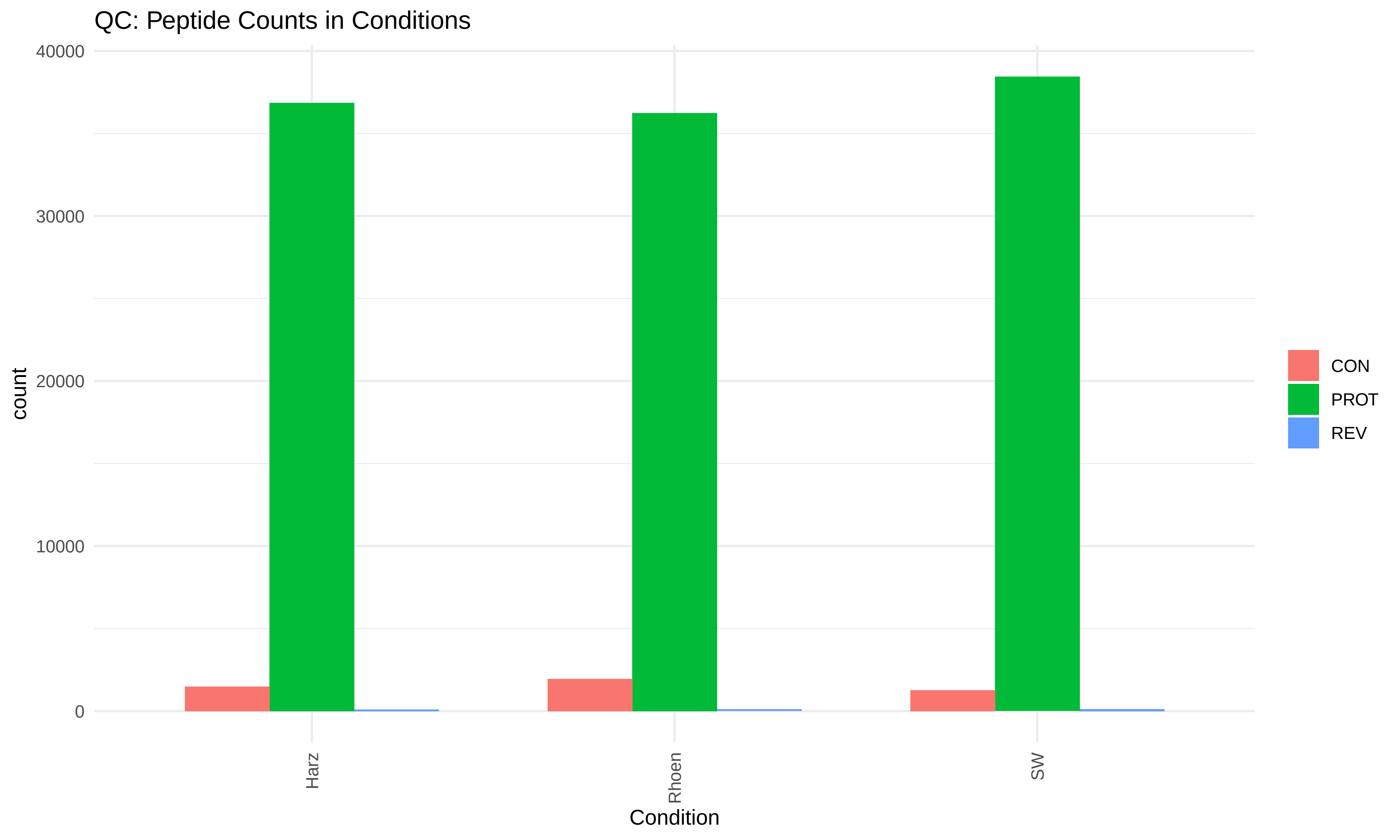
QC: Peptide Counts in Conditions
40000
30000
20000
10000
0
count
Harz
Rhoen
SW
Condition
CON
PROT
REV

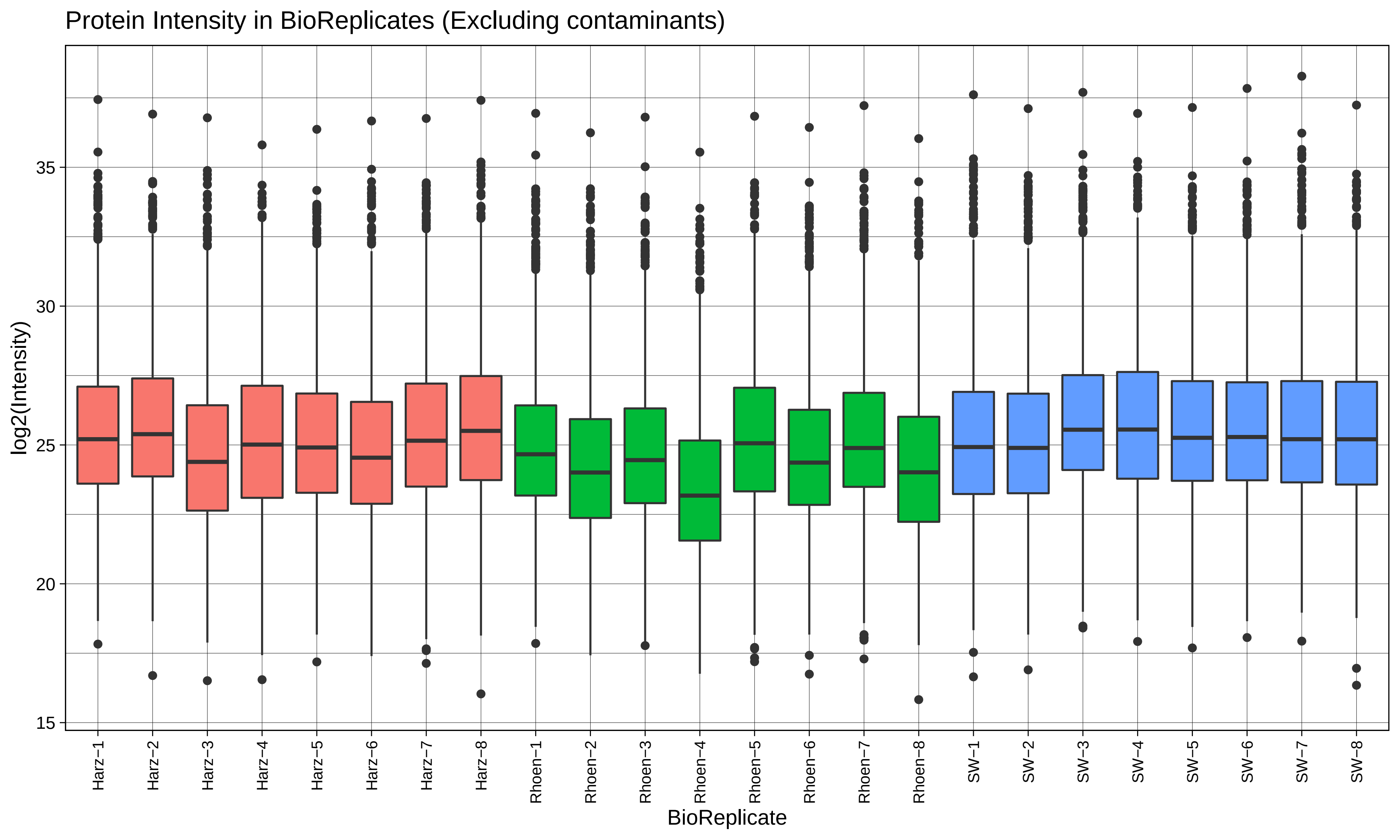

Protein Intensity in BioReplicates (Excluding contaminants)
log2(Intensity)
35
30
25
20
15
Harz−1
Harz−2
Harz−3
Harz−4
Harz−5
Harz−6
Harz−7
Harz−8
Rhoen−1
Rhoen−2
Rhoen−3
Rhoen−4
Rhoen−5
Rhoen−6
Rhoen−7
Rhoen−8
SW−1
SW−2
SW−3
SW−4
SW−5
SW−6
SW−7
SW−8
BioReplicate

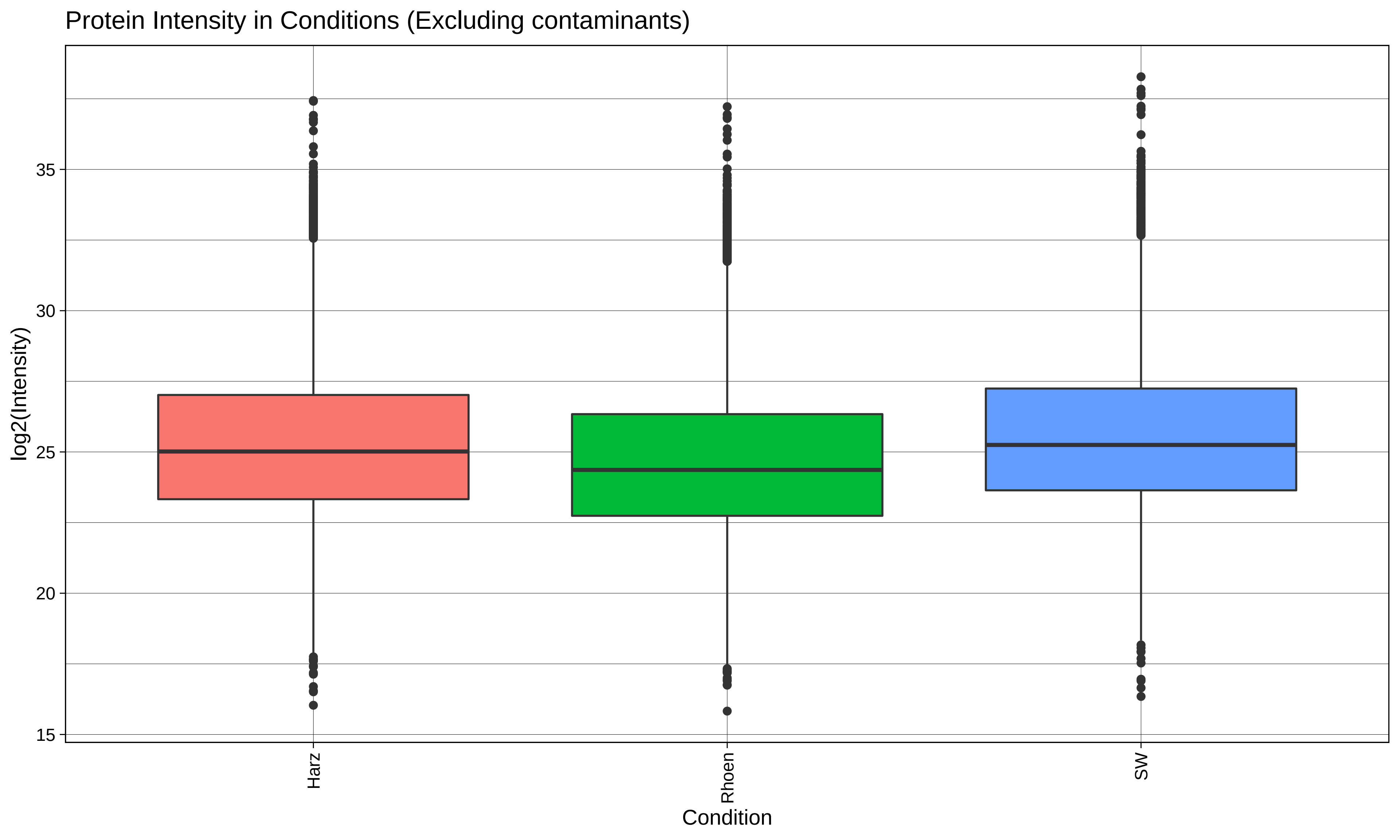

Protein Intensity in Conditions (Excluding contaminants)
log2(Intensity)
35
30
25
20
15
Harz
Rhoen
SW
Condition

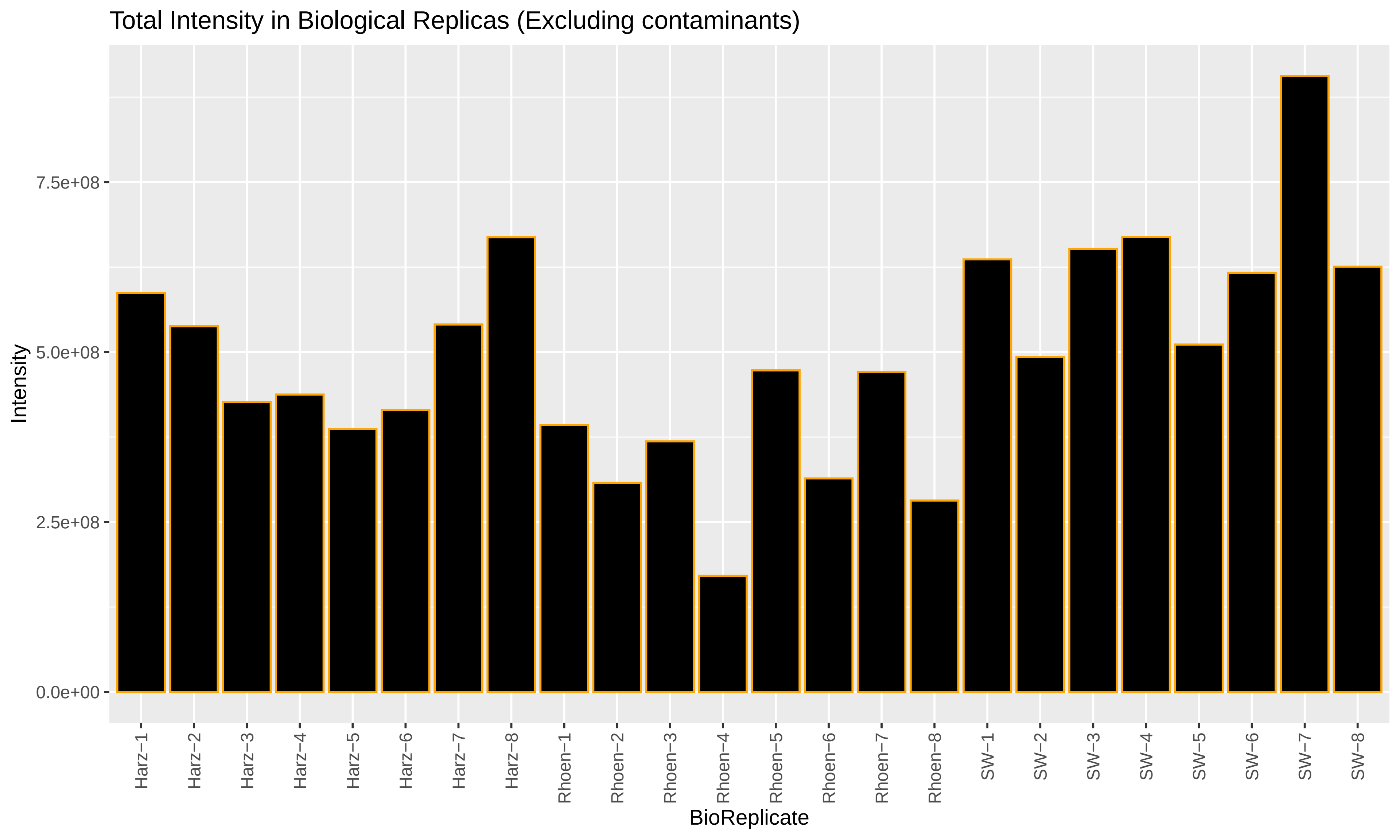

Total Intensity in Biological Replicas (Excluding contaminants)
Intensity
7.5e+08
5.0e+08
2.5e+08
0.0e+00
Harz−1
Harz−2
Harz−3
Harz−4
Harz−5
Harz−6
Harz−7
Harz−8
Rhoen−1
Rhoen−2
Rhoen−3
Rhoen−4
Rhoen−5
Rhoen−6
Rhoen−7
Rhoen−8
SW−1
SW−2
SW−3
SW−4
SW−5
SW−6
SW−7
SW−8
BioReplicate

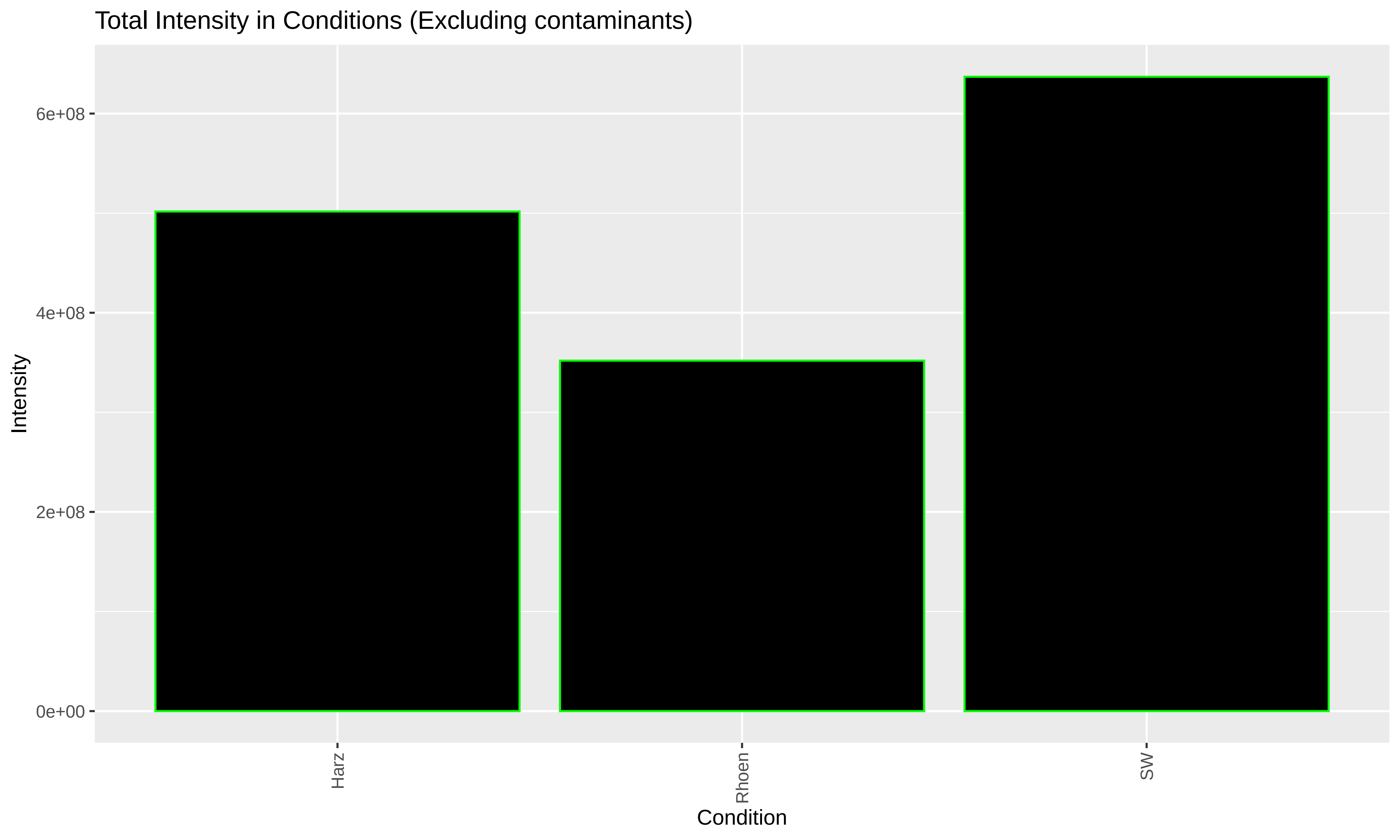
Total Intensity in Conditions (Excluding contaminants)
6e+08
4e+08
2e+08
0e+00
Intensity
Harz
Rhoen
SW
Condition

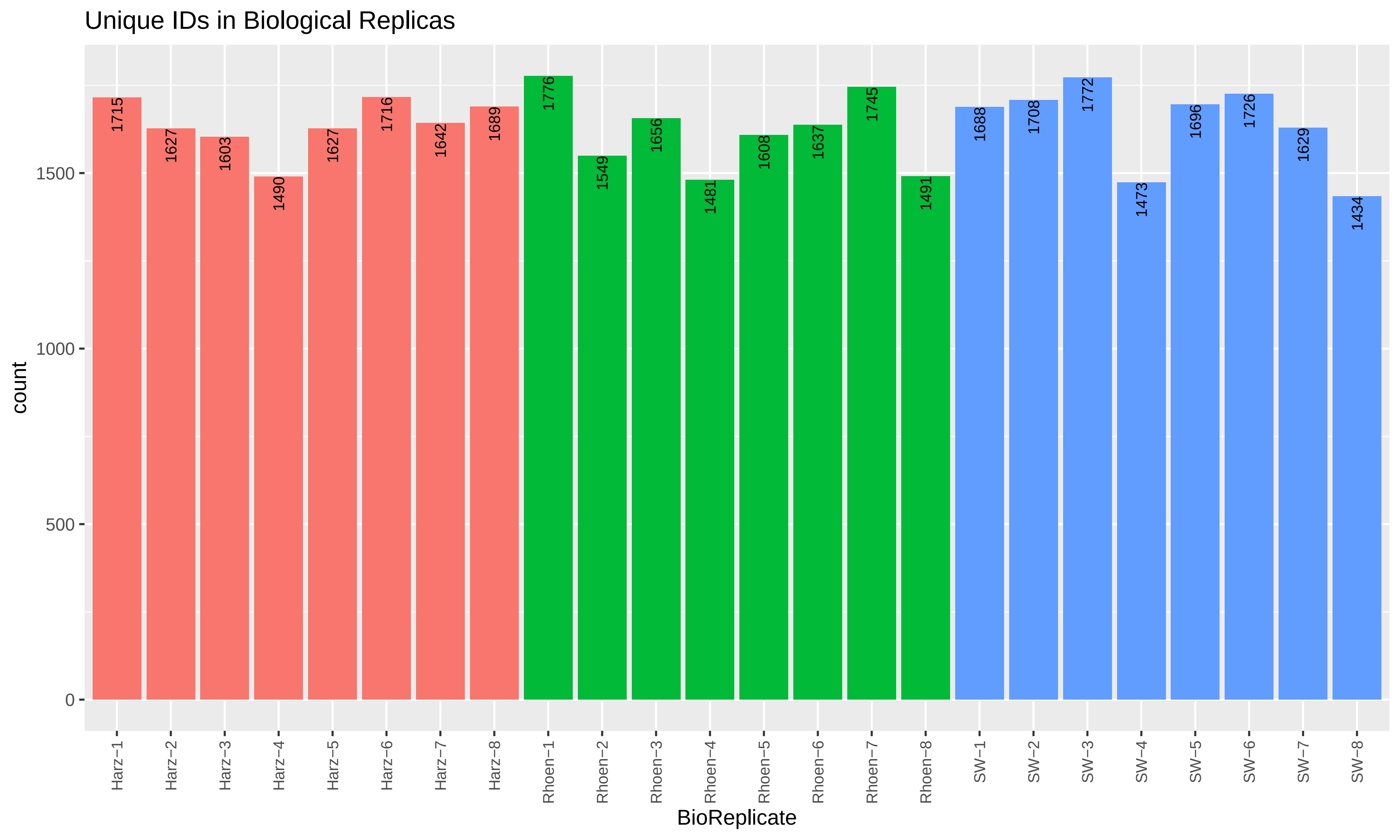

Unique IDs in Biological Replicas
count
BioReplicate
0
500
1000
1500
1715
1627
1603
1490
1627
1716
1642
1689
1776
1549
1656
1481
1608
1637
1745
1491
1688
1708
1772
1473
1696
1726
1629
1434
Harz−1
Harz−2
Harz−3
Harz−4
Harz−5
Harz−6
Harz−7
Harz−8
Rhoen−1
Rhoen−2
Rhoen−3
Rhoen−4
Rhoen−5
Rhoen−6
Rhoen−7
Rhoen−8
SW−1
SW−2
SW−3
SW−4
SW−5
SW−6
SW−7
SW−8

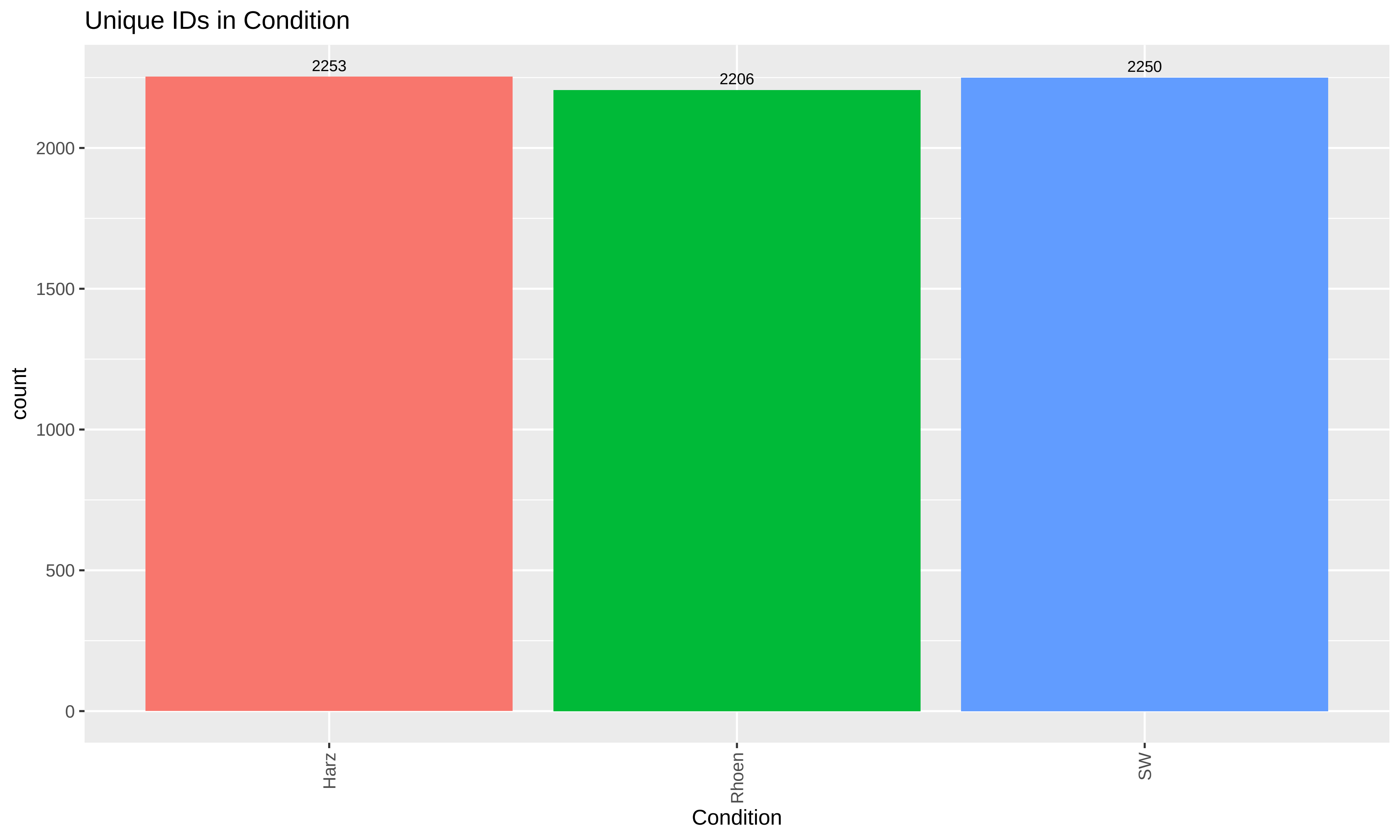
Unique IDs in Condition
count
Condition
Harz
Rhoen
SW
2253
2206
2250
0
500
1000
1500
2000
