# Supplementary material for: Abiotic and past climatic conditions drive protein abundance variation among natural populations of the caddisfly Crunoecia irrorata
Source: Sci Rep. 2020 Sep 23;10:15538. doi: 10.1038/s41598-020-72569-4 (PMC7512004; doi:10.1038/s41598-020-72569-4)

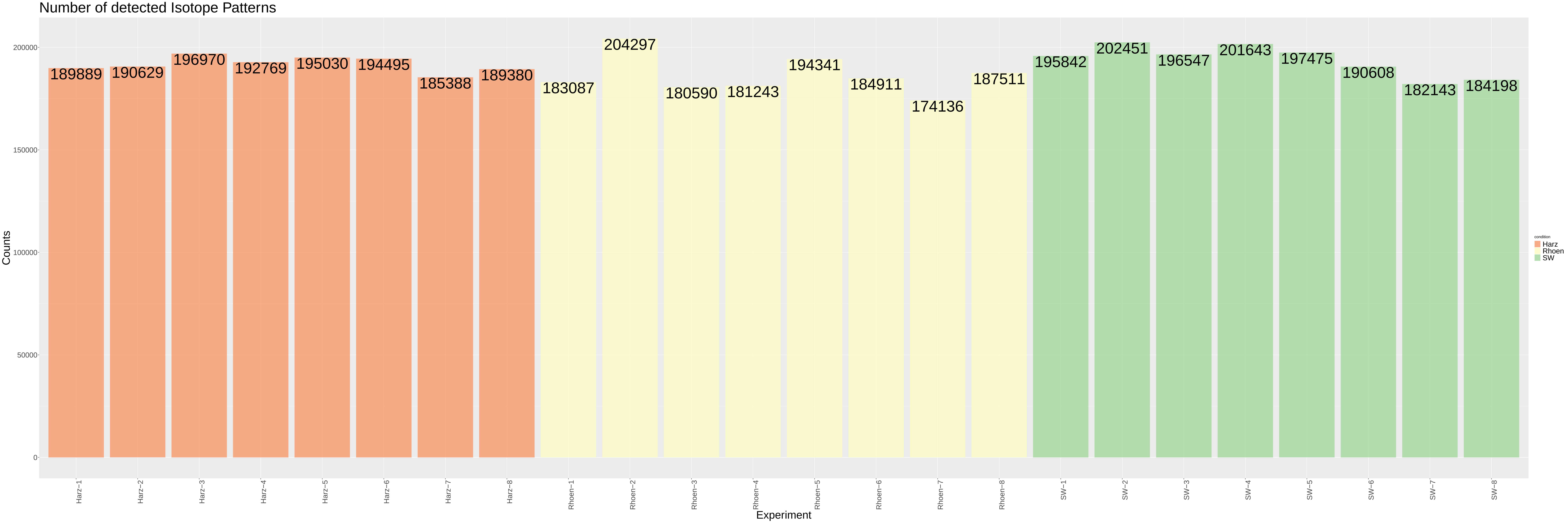
Number of detected Isotope Patterns
Counts
Experiment
0
50000
100000
150000
200000
189889
190629
196970
192769
195030
194495
185388
189380
183087
204297
180590
181243
194341
184911
174136
187511
195842
202451
196547
201643
197475
190608
182143
184198
Harz−1
Harz−2
Harz−3
Harz−4
Harz−5
Harz−6
Harz−7
Harz−8
Rhoen−1
Rhoen−2
Rhoen−3
Rhoen−4
Rhoen−5
Rhoen−6
Rhoen−7
Rhoen−8
SW−1
SW−2
SW−3
SW−4
SW−5
SW−6
SW−7
SW−8
condition
Harz
Rhoen
SW

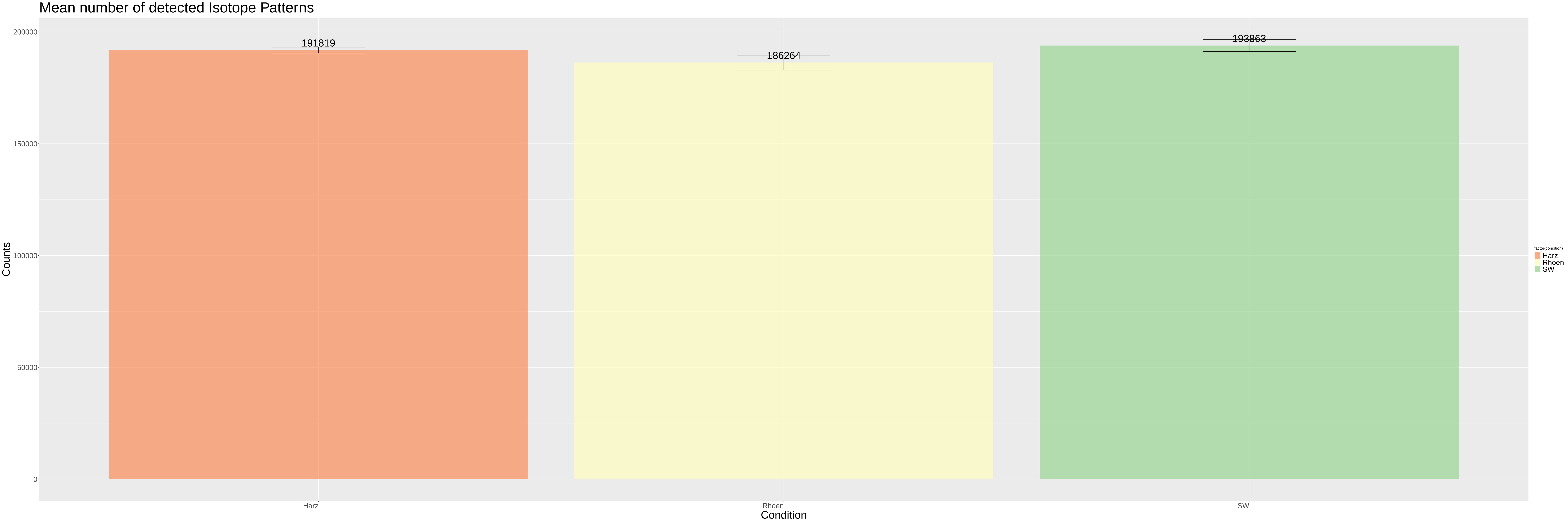
Mean number of detected Isotope Patterns
200000
150000
100000
50000
0
Counts
191819
186264
193863
Harz
Rhoen
SW
Condition
factor(condition)
Harz
Rhoen
SW

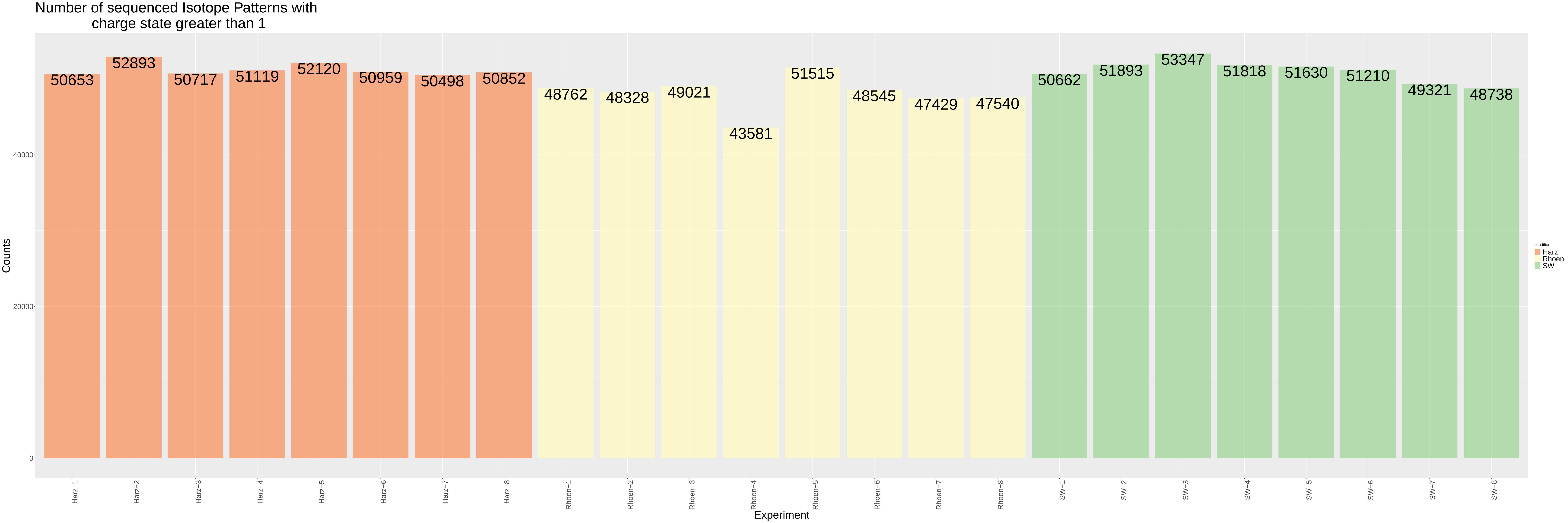
Number of sequenced Isotope Patterns with
charge state greater than 1
Counts
Experiment
40000
20000
0
50653
52893
50717
51119
52120
50959
50498
50852
48762
48328
49021
43581
51515
48545
47429
47540
50662
51893
53347
51818
51630
51210
49321
48738
Harz−1
Harz−2
Harz−3
Harz−4
Harz−5
Harz−6
Harz−7
Harz−8
Rhoen−1
Rhoen−2
Rhoen−3
Rhoen−4
Rhoen−5
Rhoen−6
Rhoen−7
Rhoen−8
SW−1
SW−2
SW−3
SW−4
SW−5
SW−6
SW−7
SW−8
condition
Harz
Rhoen
SW

Mean number of sequenced Isotope
Patterns with charge state greater than 1

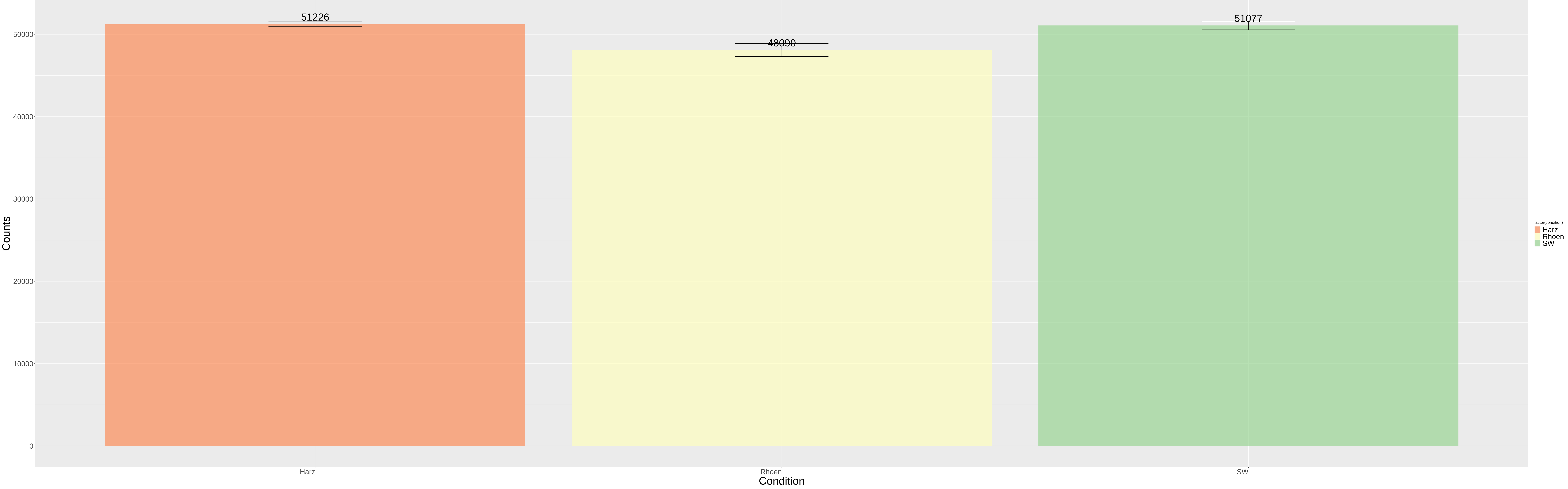

Supplement: Supplementary file 2 — Supplementary Information 2. [file 41598_2020_72569_MOESM2_ESM.zip › SI3_artMS_QC/QCSummary_ISOTOPE.pdf]
