# Supplementary material for: Abiotic and past climatic conditions drive protein abundance variation among natural populations of the caddisfly Crunoecia irrorata
Source: Sci Rep. 2020 Sep 23;10:15538. doi: 10.1038/s41598-020-72569-4 (PMC7512004; doi:10.1038/s41598-020-72569-4)

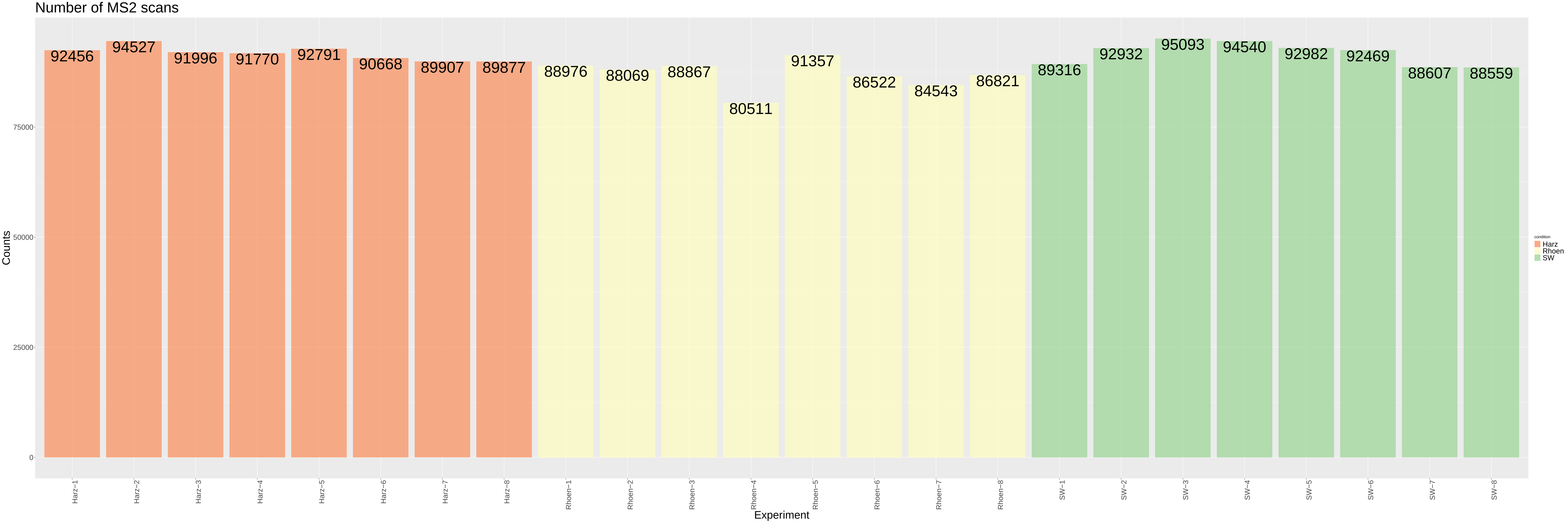

Number of MS2 scans
Counts
Experiment
0
25000
50000
75000
92456
94527
91996
91770
92791
90668
89907
89877
88976
88069
88867
80511
91357
86522
84543
86821
89316
92932
95093
94540
92982
92469
88607
88559
Harz−1
Harz−2
Harz−3
Harz−4
Harz−5
Harz−6
Harz−7
Harz−8
Rhoen−1
Rhoen−2
Rhoen−3
Rhoen−4
Rhoen−5
Rhoen−6
Rhoen−7
Rhoen−8
SW−1
SW−2
SW−3
SW−4
SW−5
SW−6
SW−7
SW−8
condition
Harz
Rhoen
SW

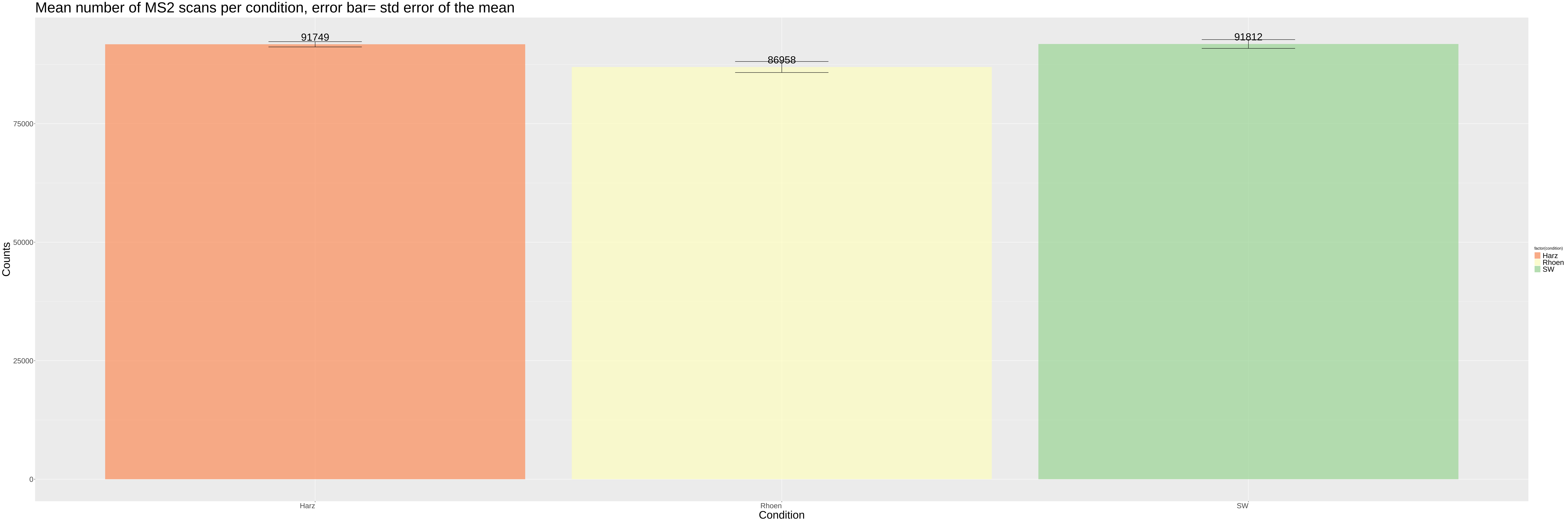
Mean number of MS2 scans per condition, error bar= std error of the mean
91749
86958
91812
Counts
75000
50000
25000
0
Harz
Rhoen
SW
Condition
factor(condition)
Harz
Rhoen
SW

# Number of MS1 and MS2 scans

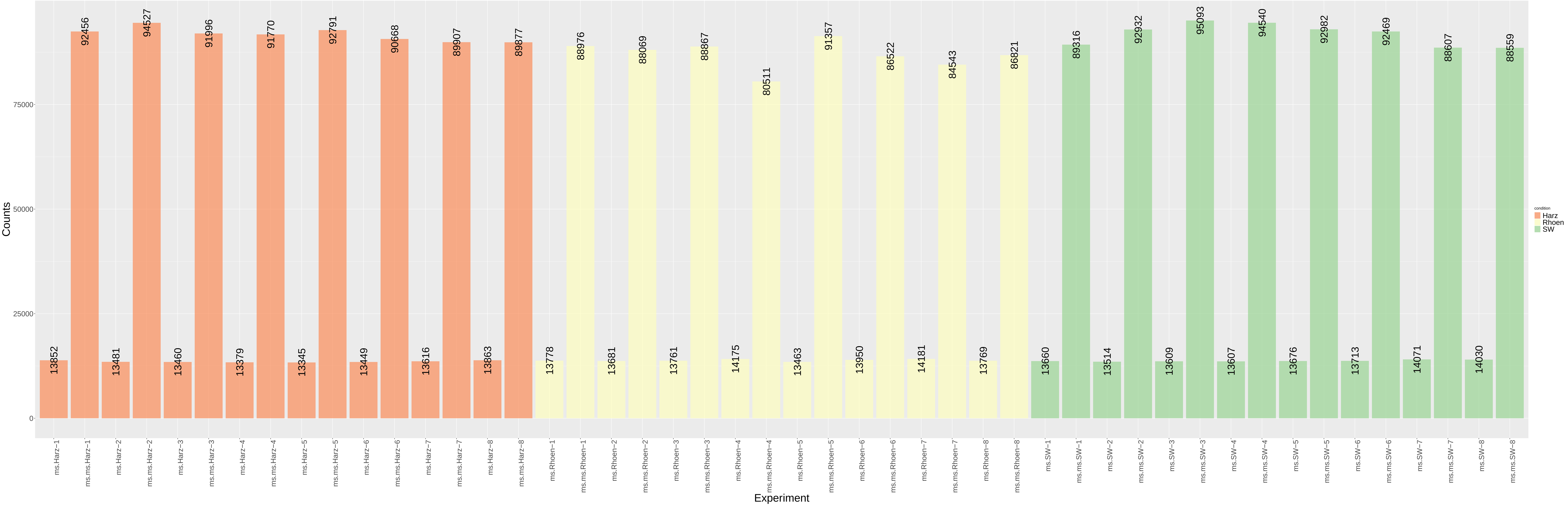

Supplement: Supplementary file 2 — Supplementary Information 2. [file 41598_2020_72569_MOESM2_ESM.zip › SI3_artMS_QC/QCSummary_MS2.pdf]
