# Supplementary figures and images for: Abiotic and past climatic conditions drive protein abundance variation among natural populations of the caddisfly Crunoecia irrorata
Source: Sci Rep. 2020 Sep 23;10:15538. doi: 10.1038/s41598-020-72569-4 (PMC7512004; doi:10.1038/s41598-020-72569-4)

Missing cleavage stats

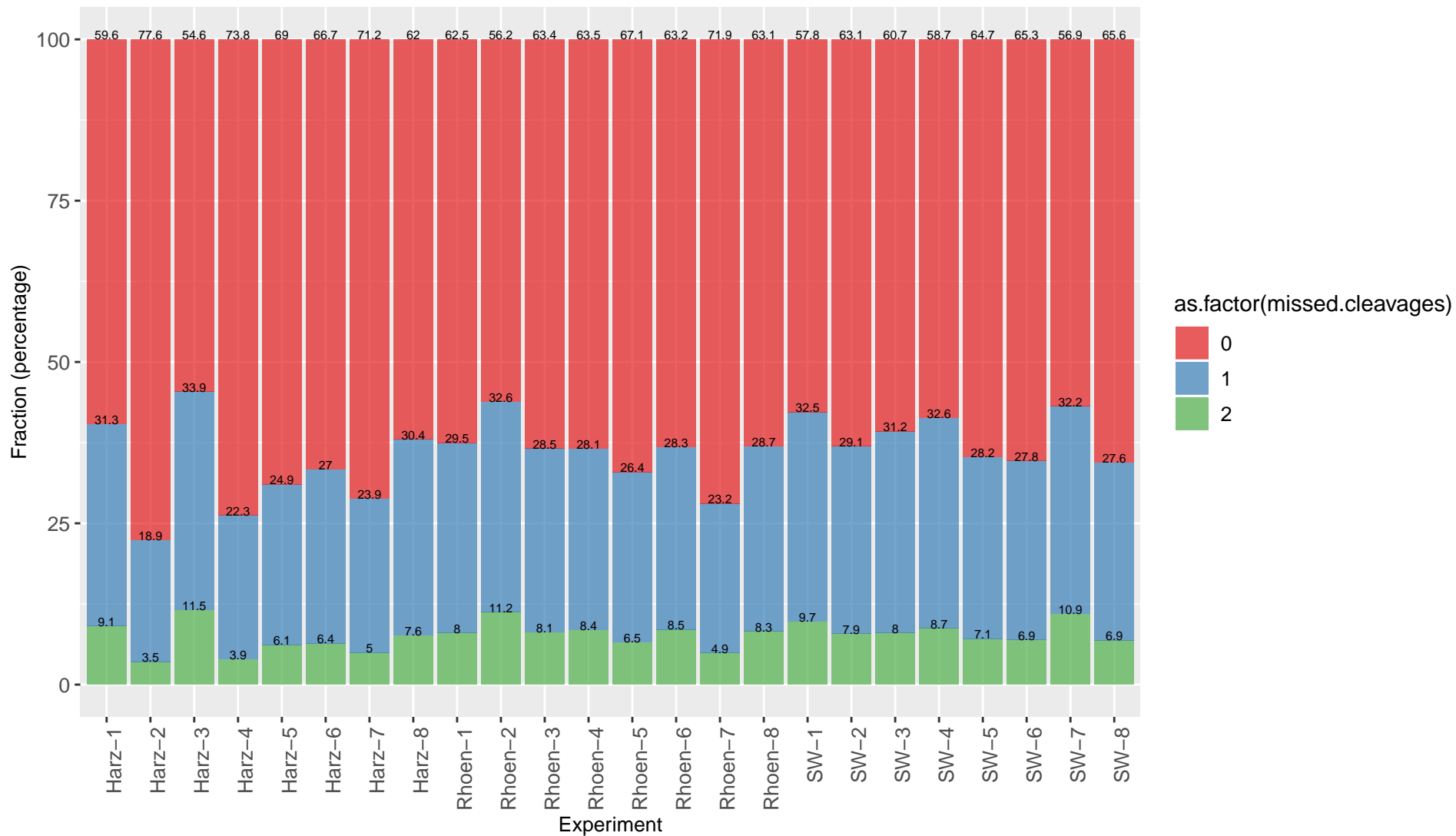

Percentage of peptides with at least 1 Methionine oxidized

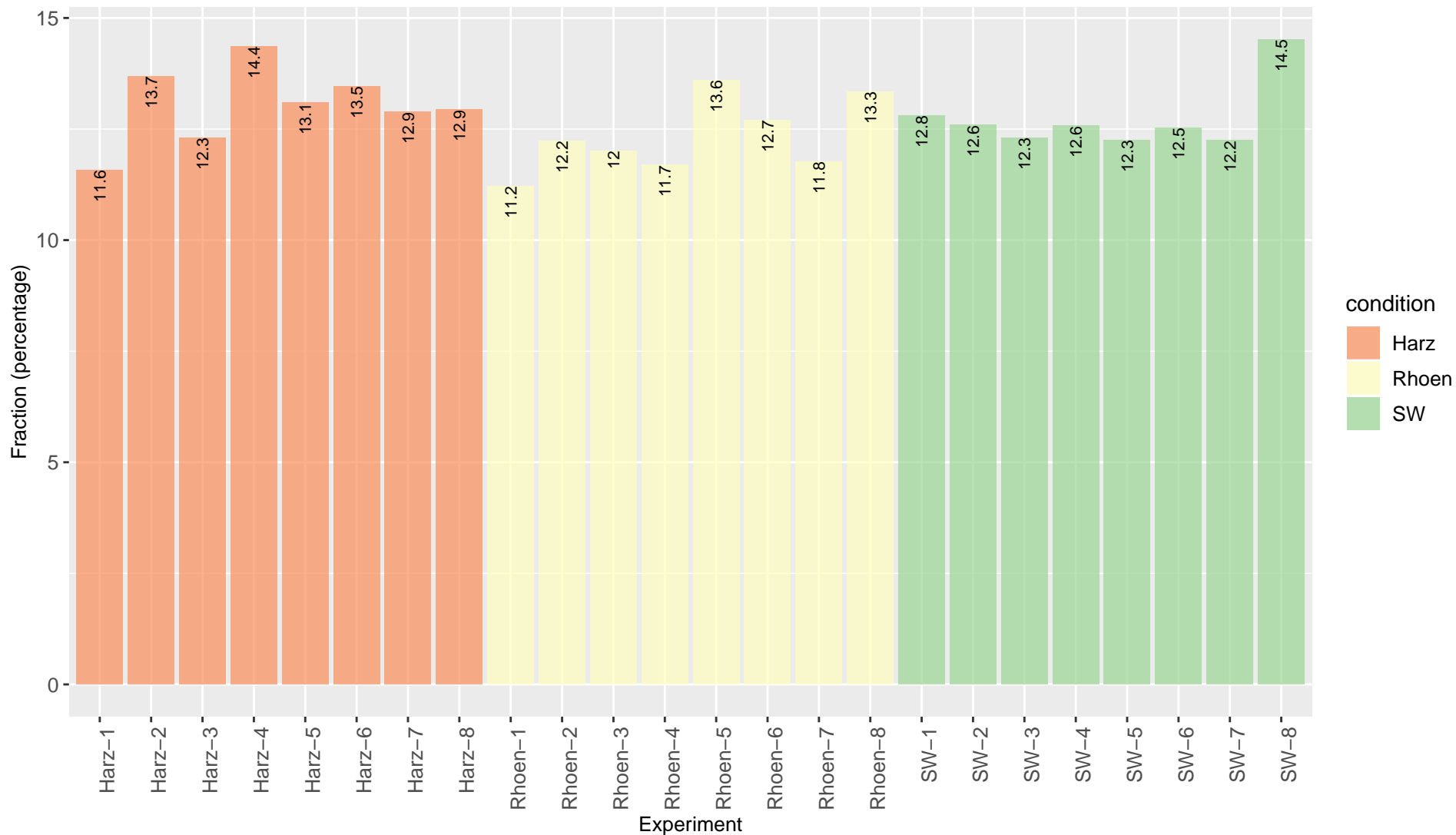

Supplement: Supplementary file 2 — Supplementary Information 2. [file 41598_2020_72569_MOESM2_ESM.zip › SI3_artMS_QC/QC-SamplePrep.pdf]

Precursor charge state distribution

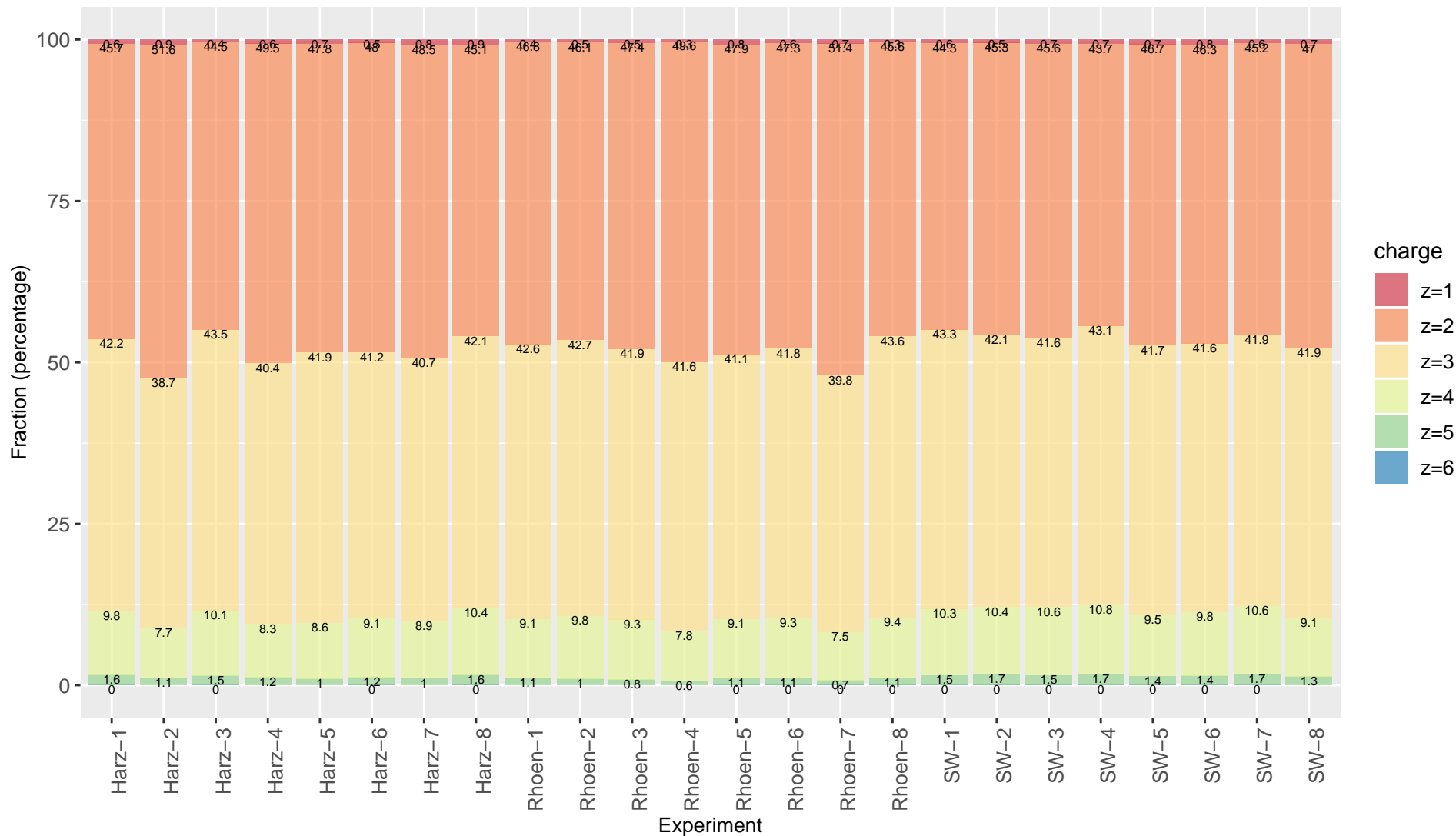

Supplement: Supplementary file 2 — Supplementary Information 2. [file 41598_2020_72569_MOESM2_ESM.zip › SI3_artMS_QC/QC_Plots_CHARGESTATE.pdf]

# Precursor mass error (in ppm) distribution

Global median mass error on the top

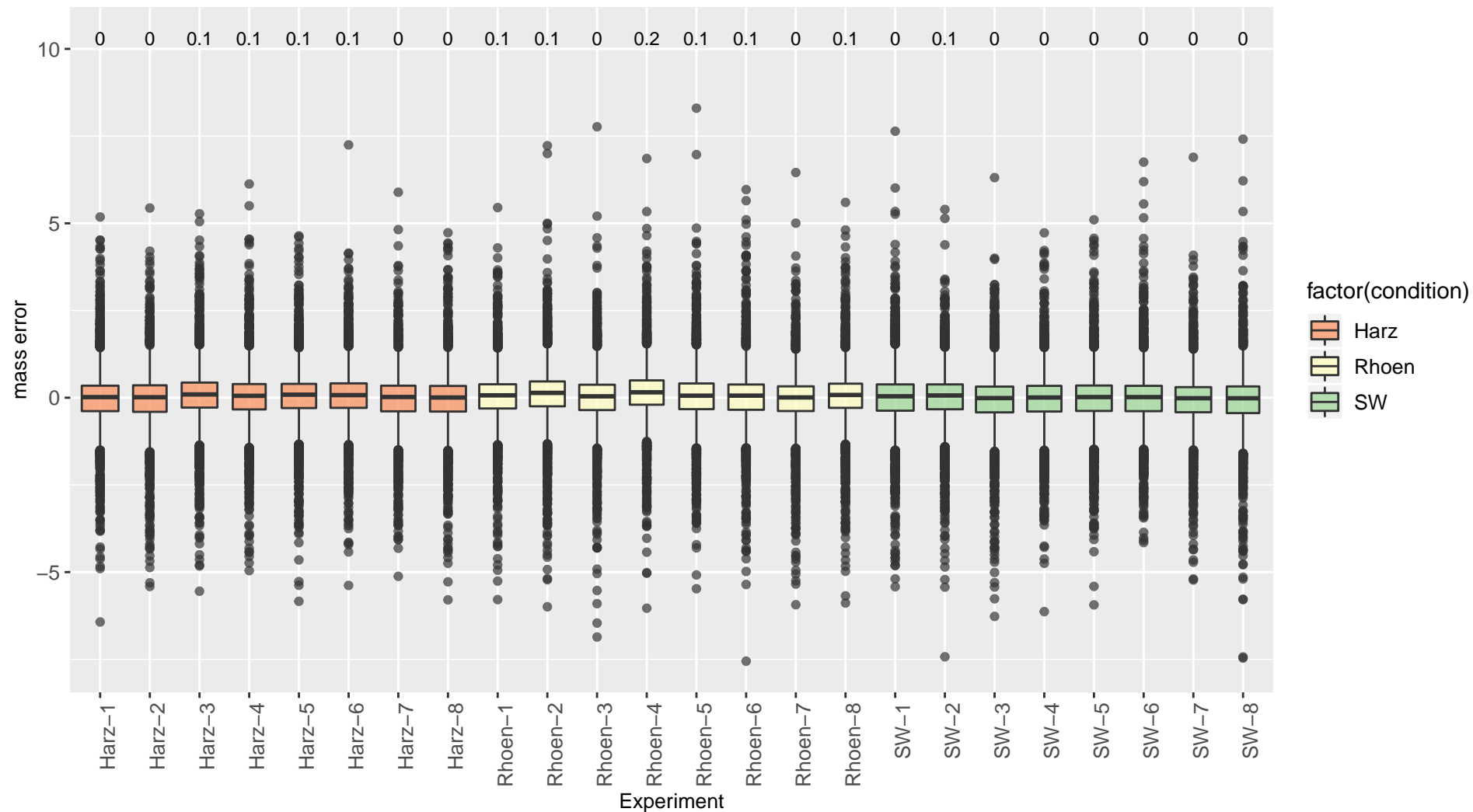

Supplement: Supplementary file 2 — Supplementary Information 2. [file 41598_2020_72569_MOESM2_ESM.zip › SI3_artMS_QC/QC_Plots_MASSERROR.pdf]

# Precursor mass-over-charge distribution

Global median  $m/z$  on the top

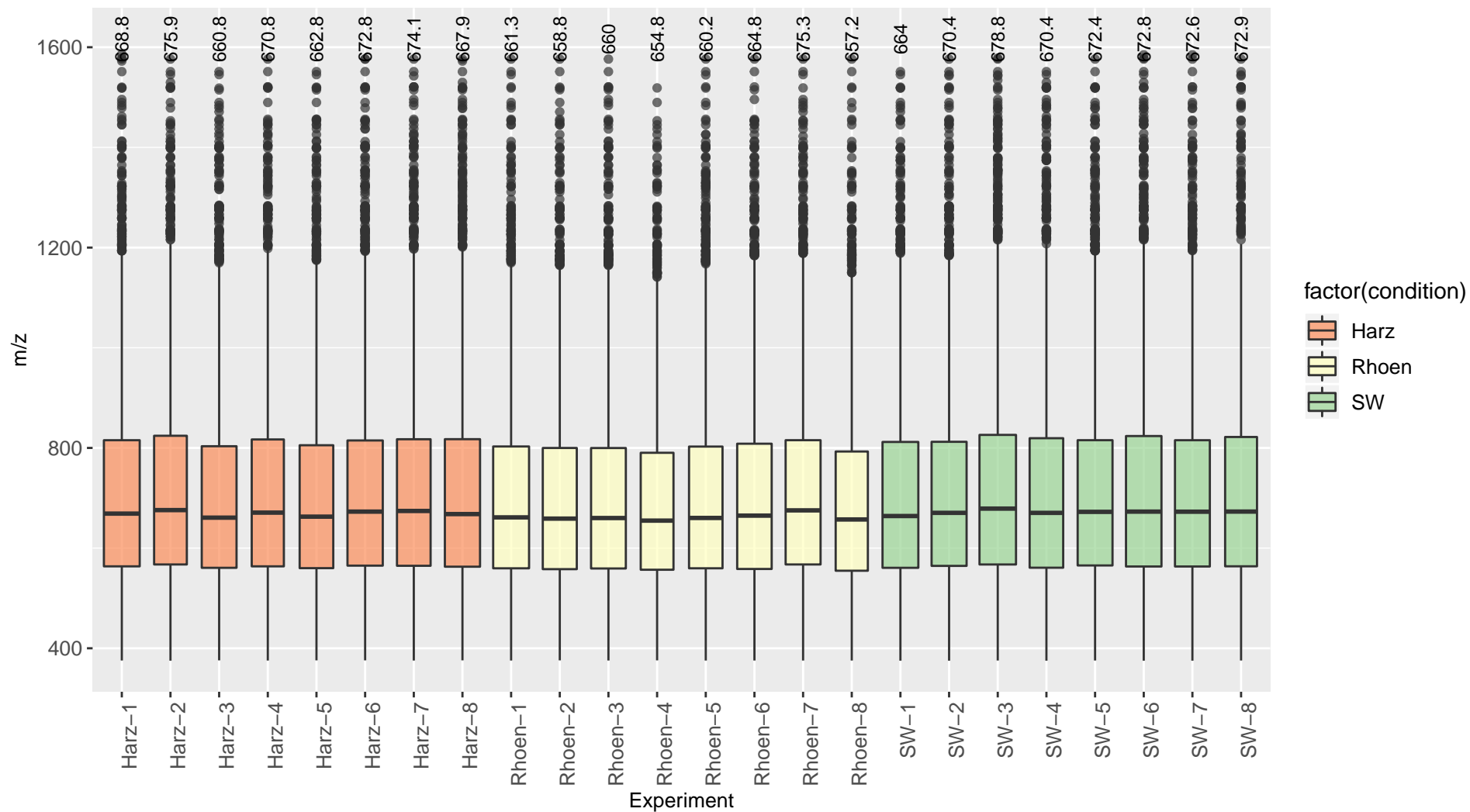

Supplement: Supplementary file 2 — Supplementary Information 2. [file 41598_2020_72569_MOESM2_ESM.zip › SI3_artMS_QC/QC_Plots_MZ.pdf]

Frequency of peptides detection

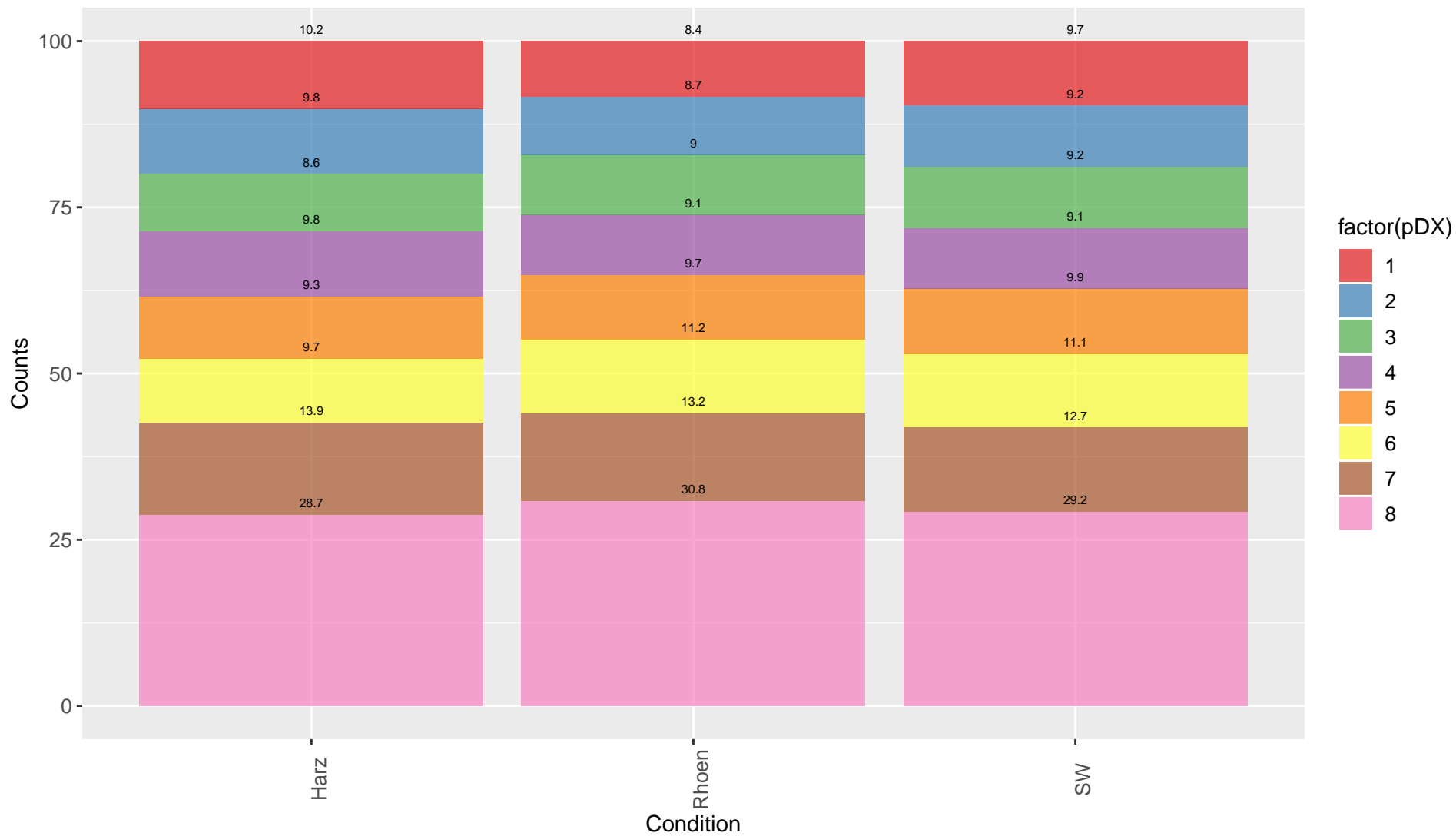

Supplement: Supplementary file 2 — Supplementary Information 2. [file 41598_2020_72569_MOESM2_ESM.zip › SI3_artMS_QC/QC_Plots_PepDetect.pdf]

Type of identification  
(MaxQuant type column)

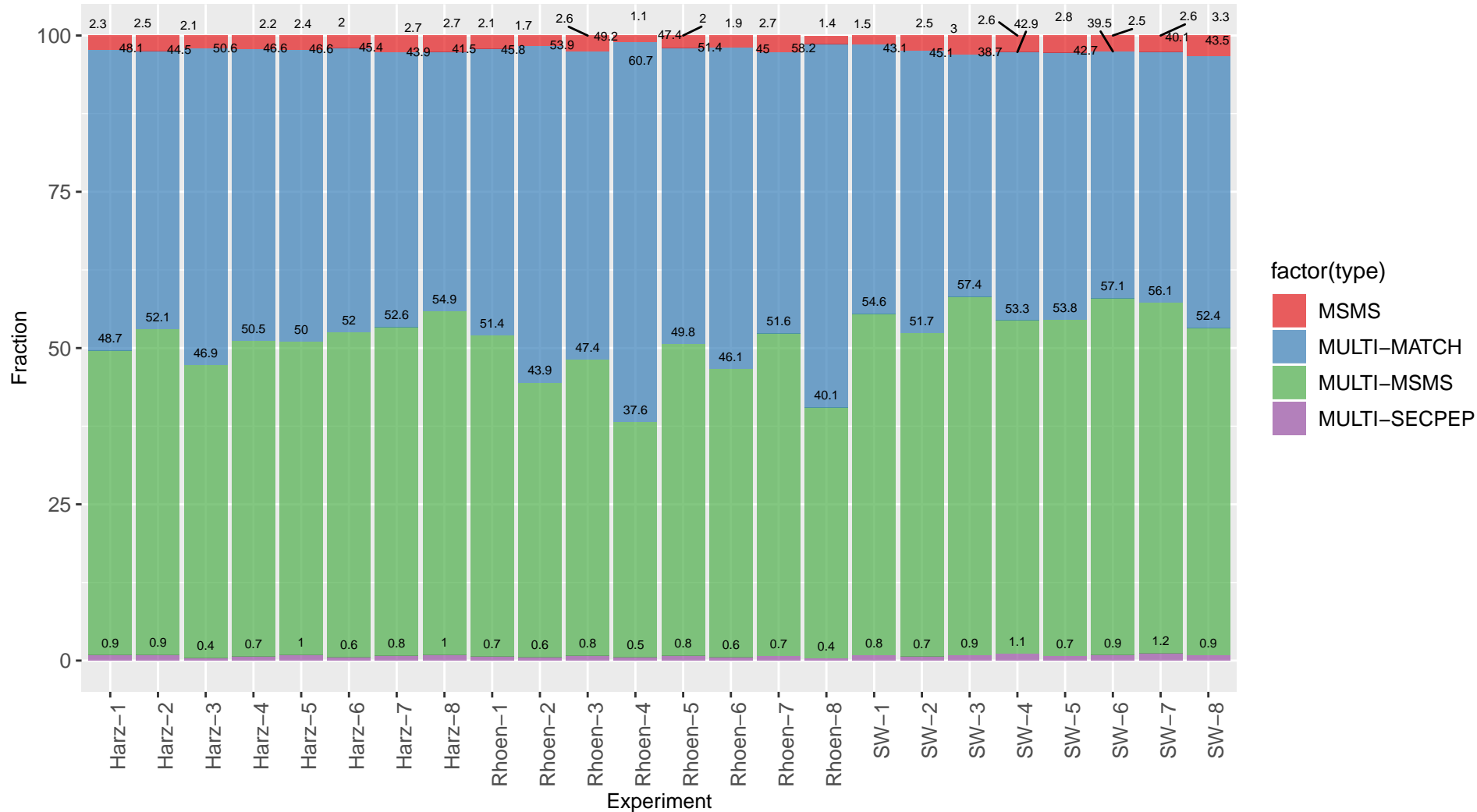

Supplement: Supplementary file 2 — Supplementary Information 2. [file 41598_2020_72569_MOESM2_ESM.zip › SI3_artMS_QC/QC_Plots_TYPE.pdf]

Matrix Correlation based on Protein Intensities

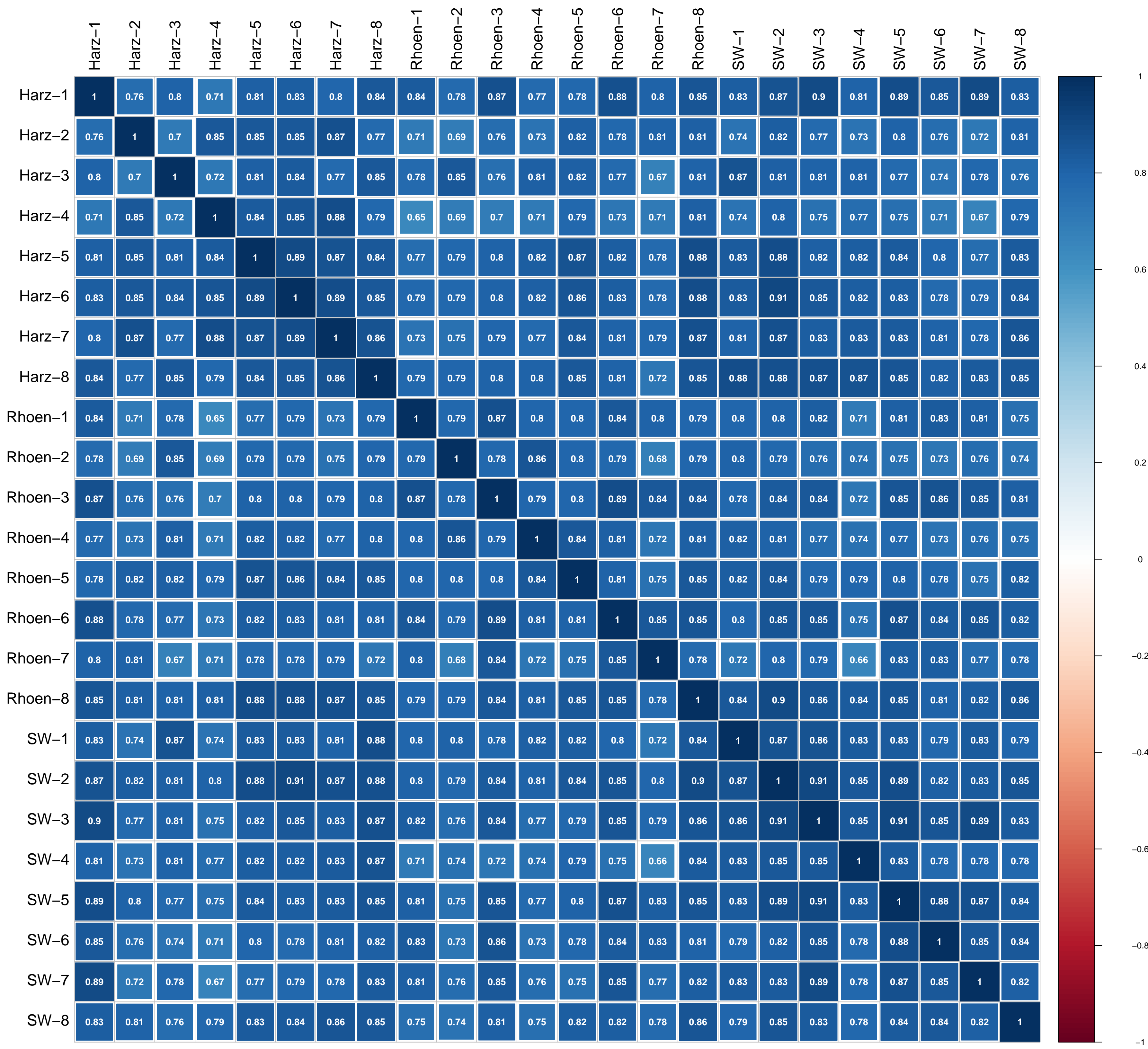

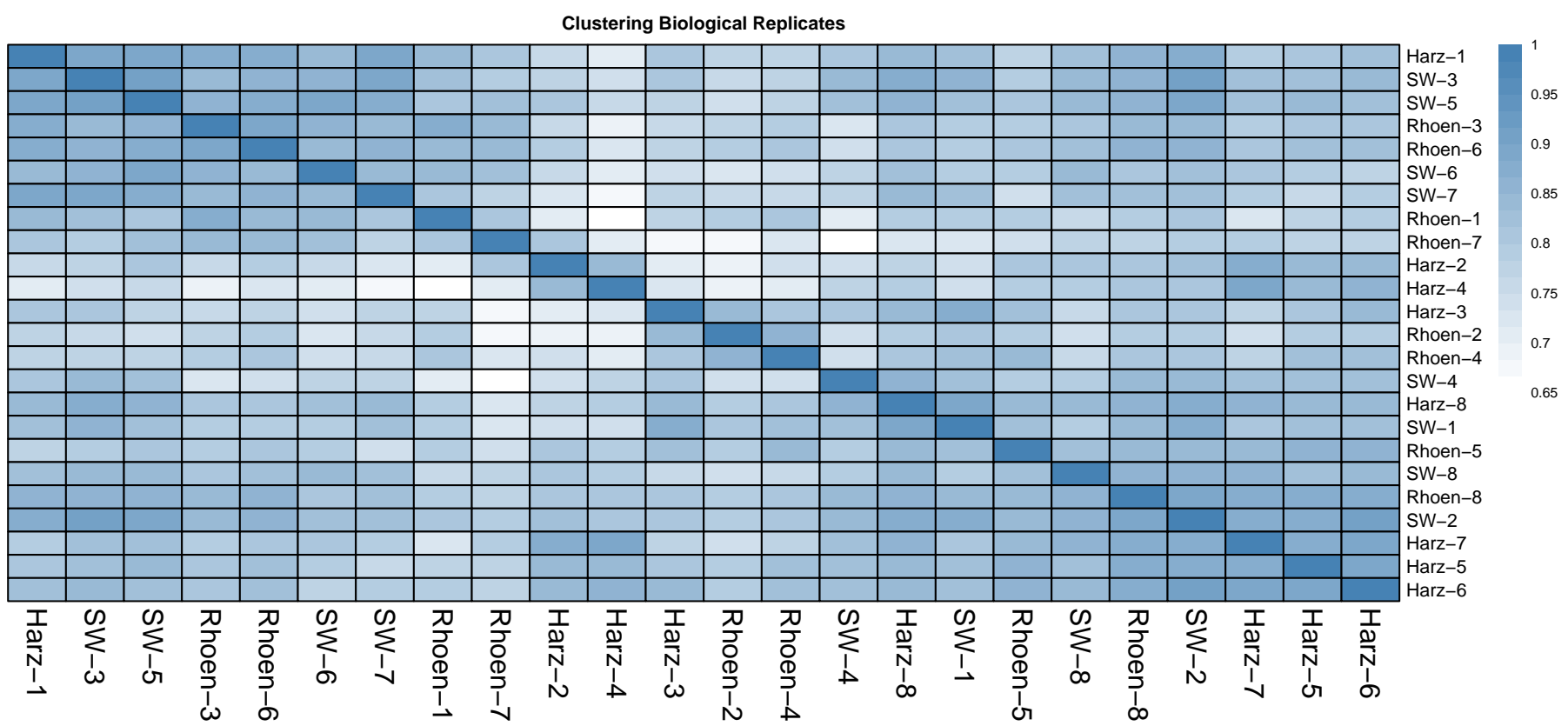

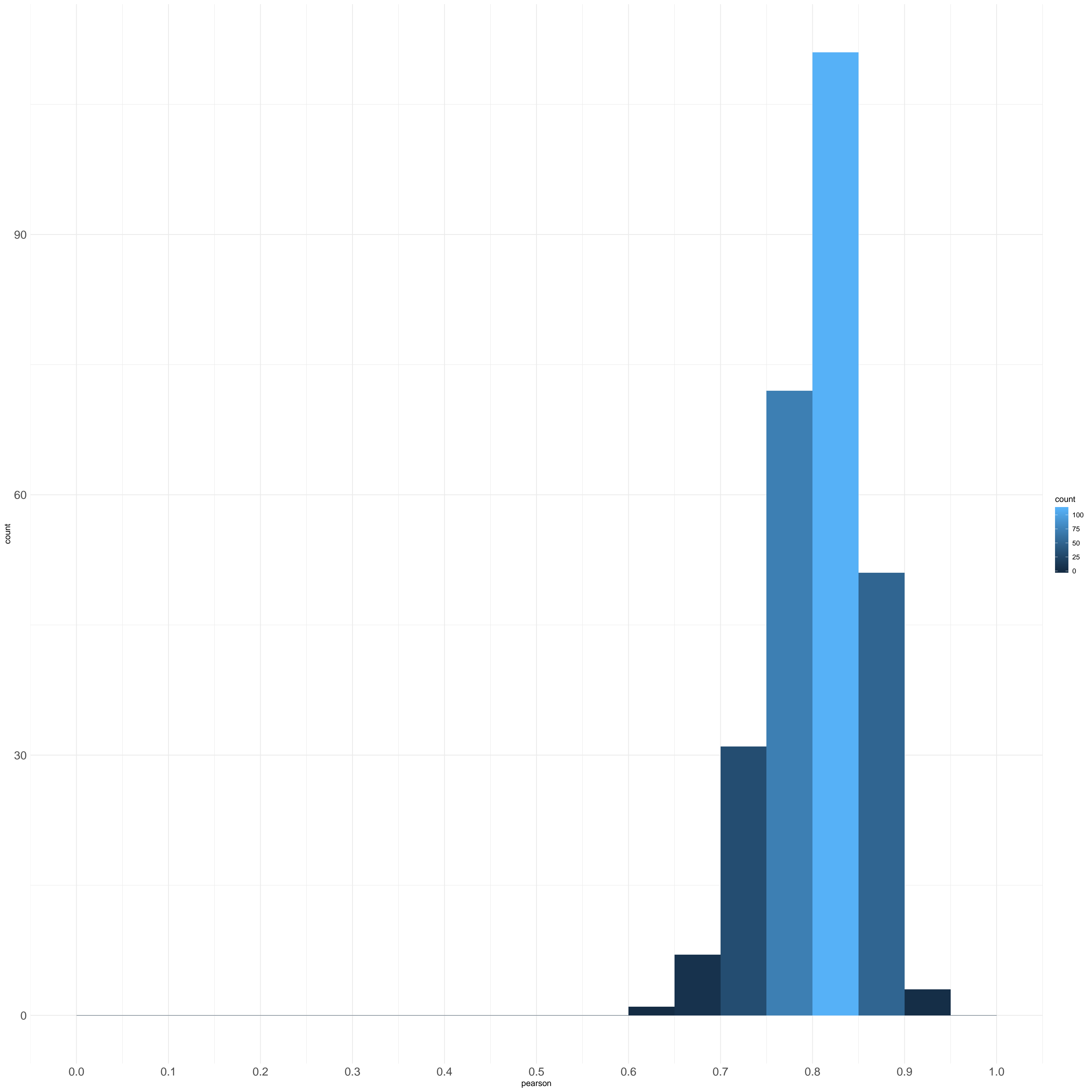

Supplement: Supplementary file 2 — Supplementary Information 2. [file 41598_2020_72569_MOESM2_ESM.zip › SI3_artMS_QC/qcPlots_evidence.qcplot.correlationMatrixBR.pdf]

Matrix Correlation based on protein intensities

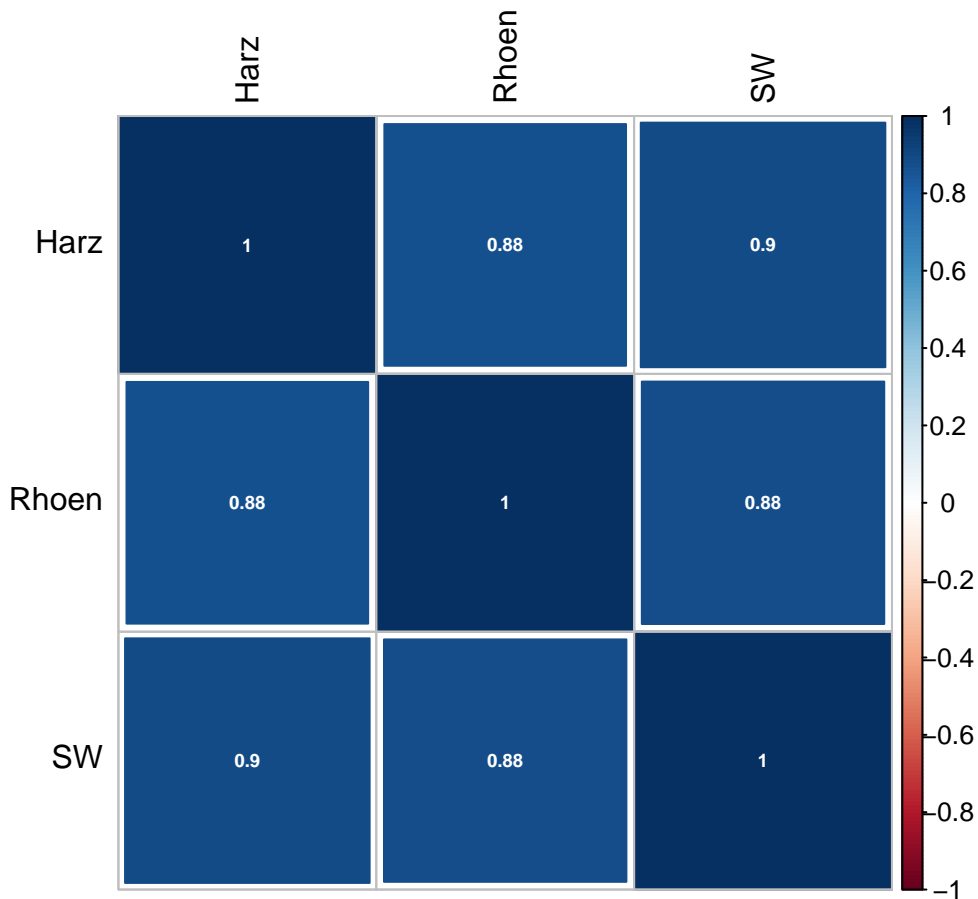

Clustering Conditions

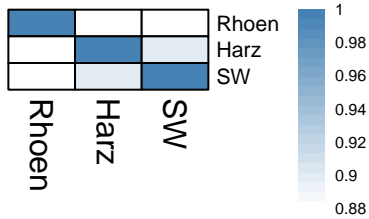

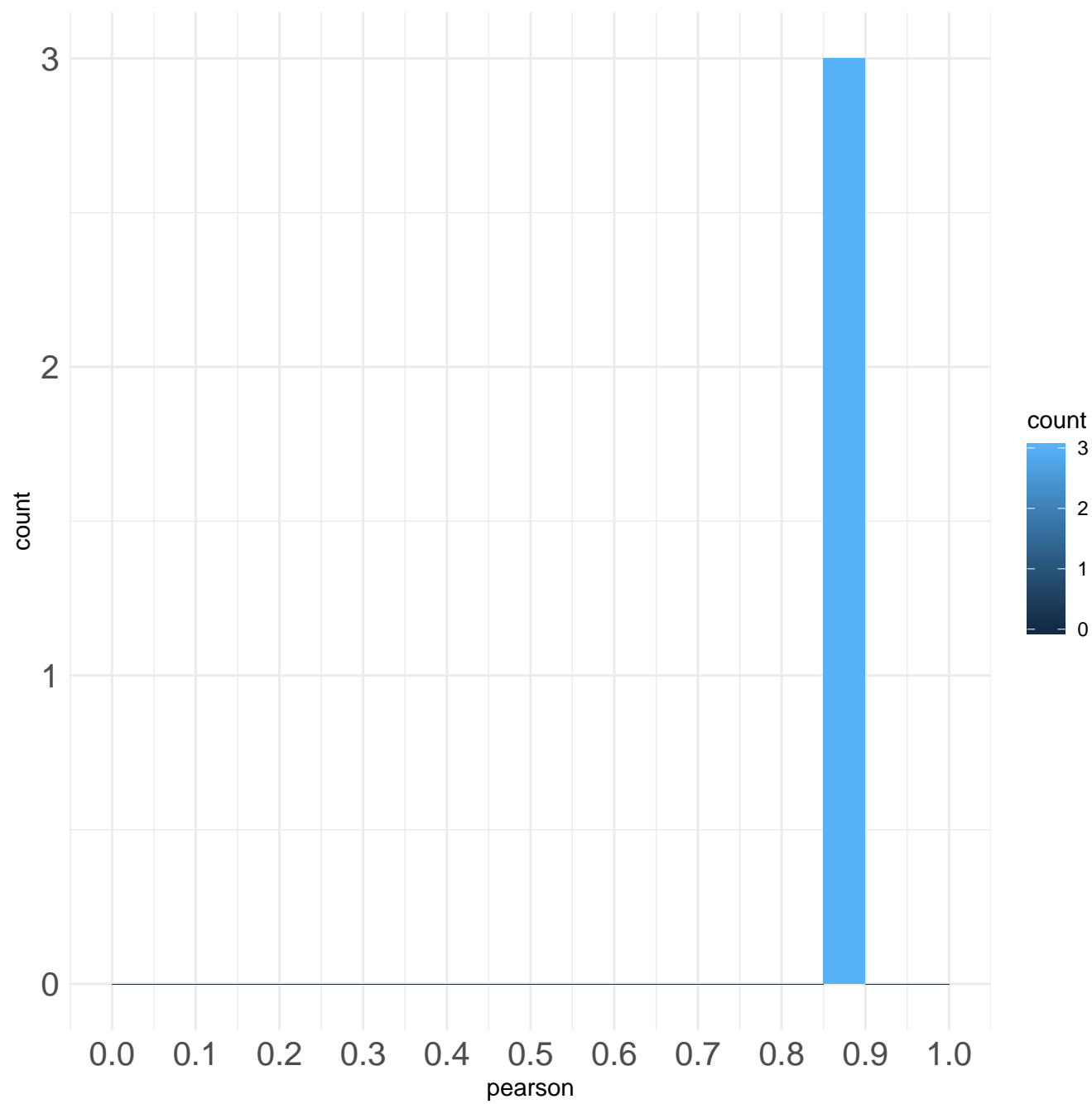

Supplement: Supplementary file 2 — Supplementary Information 2. [file 41598_2020_72569_MOESM2_ESM.zip › SI3_artMS_QC/qcPlots_evidence.qcplot.correlationMatrixConditions.pdf]

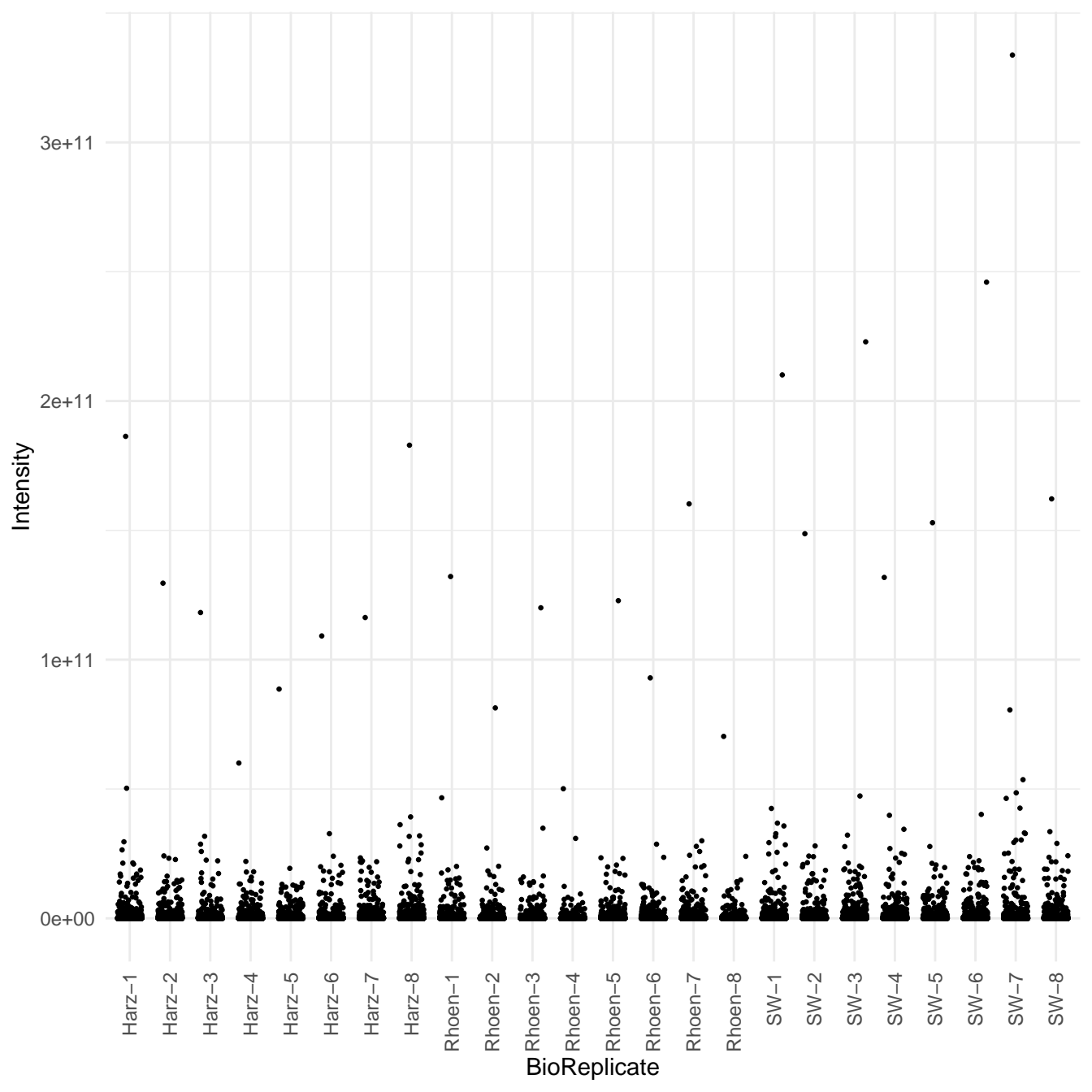

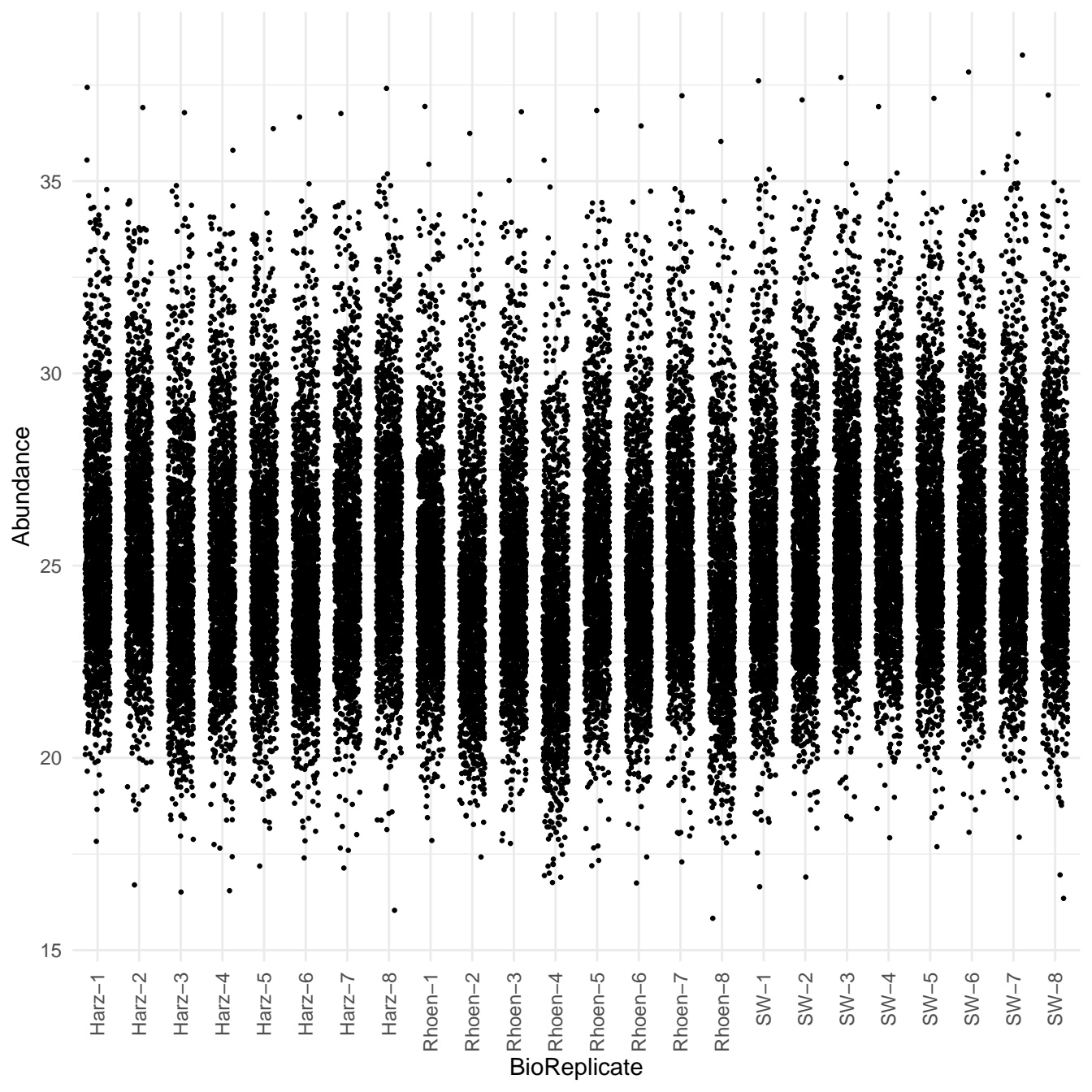

Supplement: Supplementary file 2 — Supplementary Information 2. [file 41598_2020_72569_MOESM2_ESM.zip › SI3_artMS_QC/qcPlots_evidence.qcplot.IntensityDistributions.pdf]

Number of MS1 scans

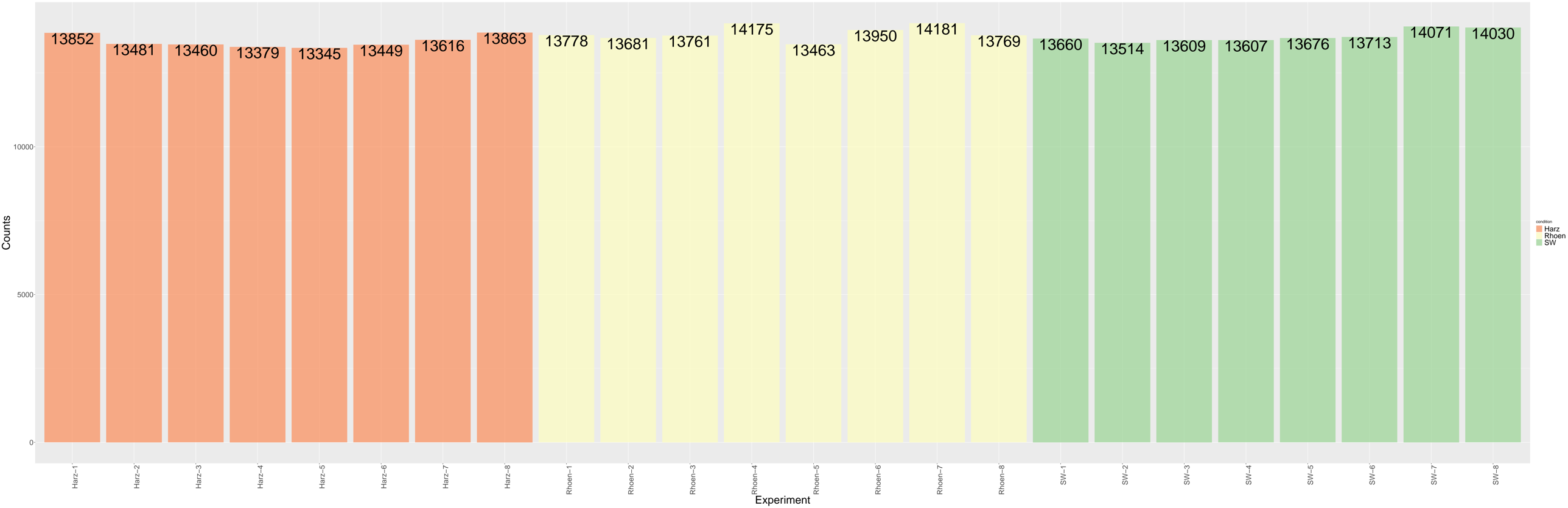

Mean number of MS1 scans per condition,  
error bar= std error of the mean

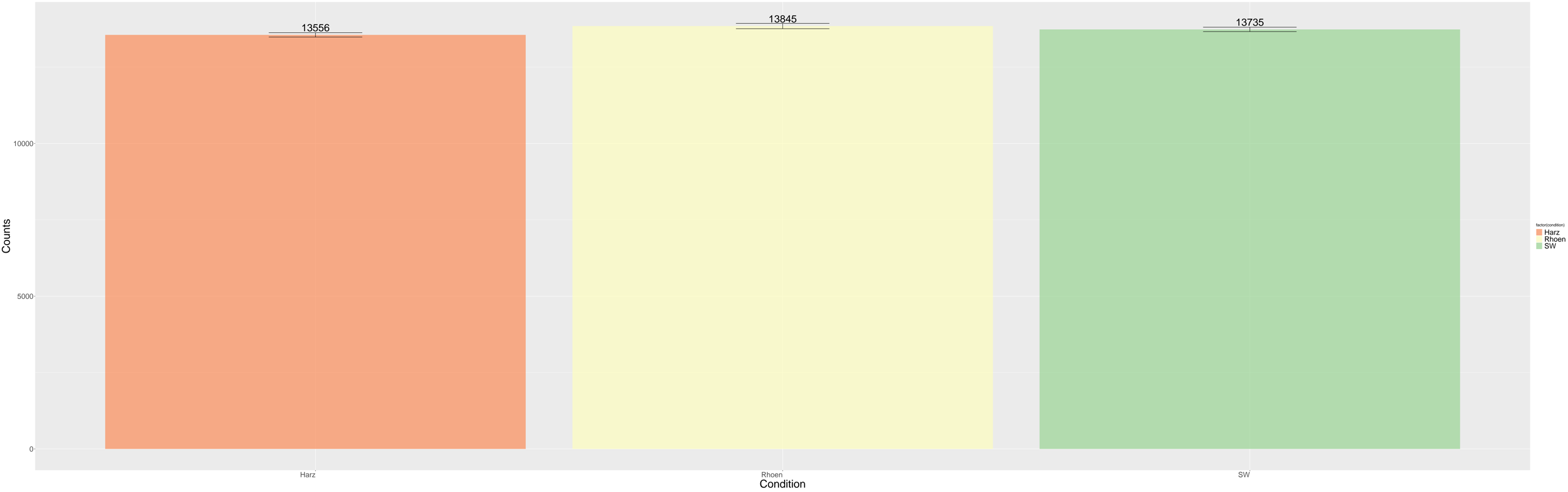

Supplement: Supplementary file 2 — Supplementary Information 2. [file 41598_2020_72569_MOESM2_ESM.zip › SI3_artMS_QC/QCSummary_MS1SCANS.pdf]

MS2 Identification rate

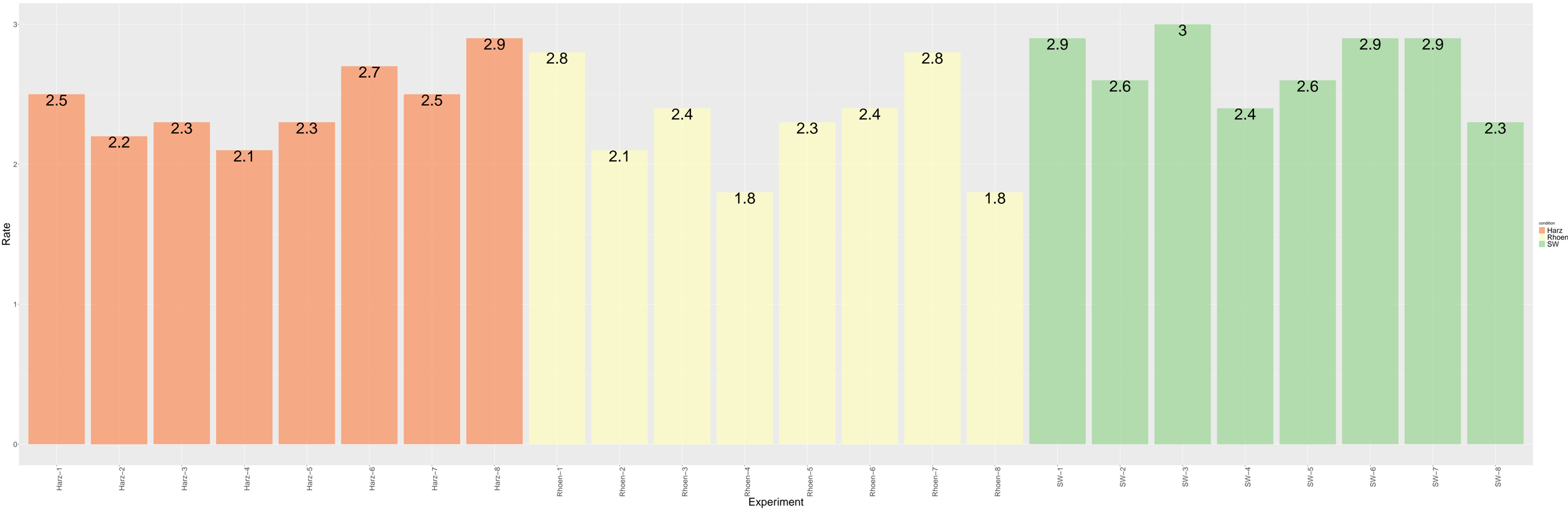

Mean MS2 Identification rate across bioreplicates and fractions

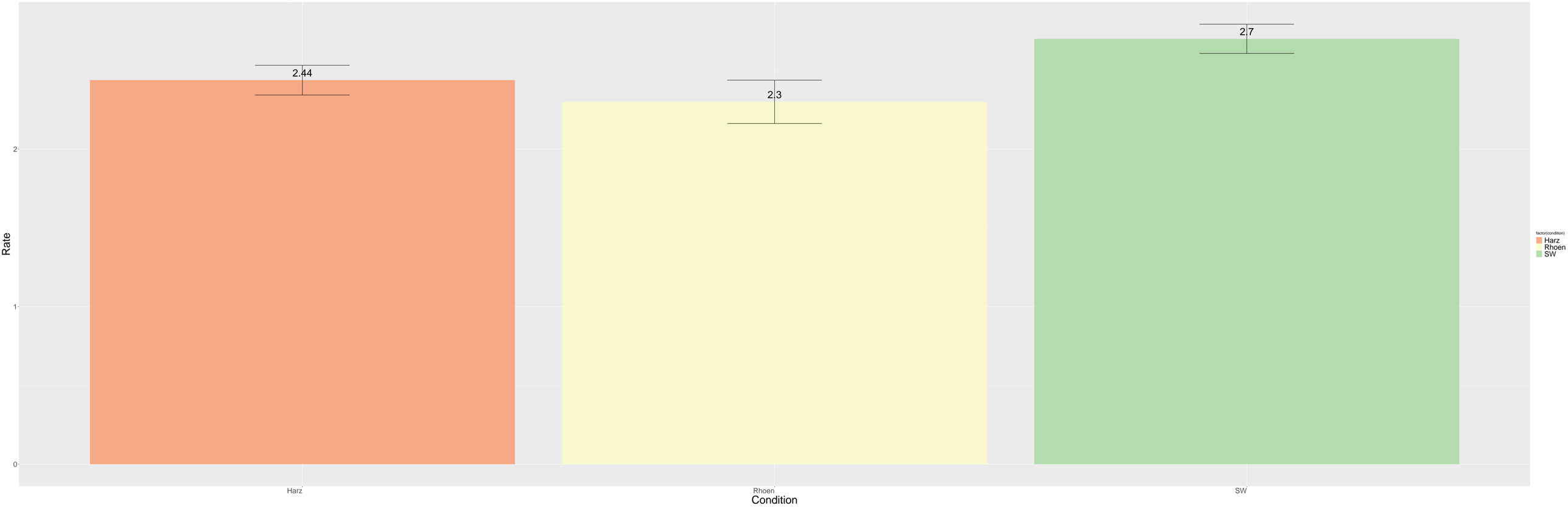

Supplement: Supplementary file 2 — Supplementary Information 2. [file 41598_2020_72569_MOESM2_ESM.zip › SI3_artMS_QC/QCSummary_MSMS.pdf]
