# Supplementary material for: Abiotic and past climatic conditions drive protein abundance variation among natural populations of the caddisfly Crunoecia irrorata
Source: Sci Rep. 2020 Sep 23;10:15538. doi: 10.1038/s41598-020-72569-4 (PMC7512004; doi:10.1038/s41598-020-72569-4)
Supplement: Supplementary file 3 — Supplementary Information 3. [file 41598_2020_72569_MOESM3_ESM.pdf]

Supporting Information File 1 for:

Abiotic and past climatic conditions drive protein abundance variation among natural populations of the caddisfly *Crunoecia irrorata*

Joshua Niklas Ebner[1*], Danilo Ritz[2], and Stefanie von Fumetti[1]

Affiliations:
[1]Spring Ecology Research Group, Department of Environmental Sciences, University of Basel, Basel, Switzerland
[2]Proteomics Core Facility, Biozentrum, University of Basel, Basel, Switzerland

***Correspondence:** joshuaebner@unibas.ch

Contents:

Supplemental Tables

Supplementary Table S1 | Information on sampled freshwater springs (code (by the Authors, does not reflect official coding), elevation, sampling date, region, latitude and longitude (decimal values))

Supplementary Table S2 | Peptide and protein concentration measurements of population-wide samples subjected to LC-MS/MS analysis

Supplemental Figures

Supplementary Figure S1 | Dotplots of mean values of all abiotic measurements

Supplementary Figure S2 | Cor.test output of pairwise comparisons of abiotic variables

Supplementary Figure S3 | NAs of MaxQuant LFQ

Supplementary Figure S4 | nMDS stress plot

Supplementary Figure S5 | Statistics and results of the WGCNA analysis

Supplementary Figure S6 | Statistics and results of the $WGCNA_{BC}$ analysis

Supplementary Figure S7 | Capsule width and protein ID overlap

Supplementary Figure S8 | Scatterplot and linear regression of protein abundance distance and geographic distance between springs

Supplementary Figure S9 | Scatterplot and linear regression of environmental distance and geographic distance between springs

Supplementary Figure S10 | Boxplots and significance results of Kruskal-Wallis pairwise comparisons of 4 abiotic variables between sampling regions

Supplementary Figure S11 | Reaction norms of 33 proteins in relation to elevation of springs

Supplementary Figure S12 | Pathways related to changes in aqueous pH

Supplementary Figure S13 | Pfam family frequency in modules “green” and “yellow” of the WGCNA

Supplementary Figure S14 | Exemplary reaction norms (polynomial regression) of population-wide protein biomarker abundances (y-axis) in relation to spring altitude and temperature (x-axis)

Supplementary Figure S15 | Heat-shock protein reaction norms

Supplementary Figure S16 | Pfam family frequencies in reaction norm proteins

Supplementary Figure S17 | Overview of $WGCNA_{BC}$ modules, combining COG family frequencies and eigengene-BioClim correlations for each identified module.

Supplementary Figure S18 | Overview of WGCNA modules, combining COG family frequencies and eigengene-abiotic variable correlations for each identified module.

Supplementary Figure S19 | Statistics and results of the $WGCNA_{DAP}$ analysis.

Supplementary Figure S20 | COG family distribution of differentially abundant proteins (DAPs) between sampling regions identified via LIMMA.

Supplementary Figure S21 | COG family distribution of proteins with positive eigenvector loadings in nMDS axis 2

Supplementary Table S1 | Code = Spring- (population)-codes, Elev. = elevation (m.a.s.l), Sample Date = Sampling of springs (identifying individuals and storing them in RNAlater, measuring of abiotic variables and noting coordinates) occurred between 07:00 and 18:00 on all sampling days), Regions = One of the three regions in which springs were probed (Rhoen = Rhoen Biosphere Reserve, Harz = Harz National Park, BF = "Black Forest"), Lat. & Long. = latitudes and longitudes in decimal values for each sampled spring as recorded with the "GPS Coordinates" App for Android, referenced with a *e*Trex Summit® HC handheld GPS (Garmin).

Code	Elev.	Sample Date	Regions	Lat. (Decimal)	Long. (Decimal)
R1	370	29.07.2019	Rhoen	50.613586	9.764174
R2	570	29.07.2019	Rhoen	50.5993729	9.99908903
R3	600	29.07.2019	Rhoen	50.5889192	10.0041492
R4	818	29.07.2019	Rhoen	50.4678952	10.0136548
R5	815	29.07.2019	Rhoen	50.4658716	10.0054564
R6	730	29.07.2019	Rhoen	50.5035052	9.92957296
R7	606	29.07.2019	Rhoen	50.517887	9.932069
R8	275	30.07.2019	Rhoen	50.6096721	9.64546653
R9	340	30.07.2019	Rhoen	50.5993789	9.66936553
H1	332	31.07.2019	Harz	51.637179	10.406294
H2	390	31.07.2019	Harz	51.70213	10.343042
H3	764	01.08.2019	Harz	51.767917	10.610061
H4	696	01.08.2019	Harz	51.767861	10.612028
H5	482	01.08.2019	Harz	51.742708	10.675715
H6	517	01.08.2019	Harz	51.742801	10.675805
H7	601	02.08.2019	Harz	51.726299	10.564328
H8	597	02.08.2019	Harz	51.643169	10.696474
H9	577	02.08.2019	Harz	51.647887	10.741779
SW1	912	19.08.2019	BF	47.652422	7.975453
SW2	912	19.08.2019	BF	47.652422	7.975456
SW3	967	19.08.2019	BF	47.652778	7.973611
SW4	346	19.08.2019	BF	47.597678	7.840197
SW5	498	20.08.2019	BF	47.662372	7.901392
SW6	460	20.08.2019	BF	47.65695	7.907233
SW7	511	21.08.2019	BF	47.655839	7.767608
SW8	589	21.08.2019	BF	47.658853	7.770014
SW9	598	21.08.2019	BF	47.659061	7.7702

Supplementary Table S2 | Sample number, spring code, protein concentrations (µg/µl) peptide concentrations (µg/µl) and amount of protein used for trypsin digestion (µg/µl) of pooled larvae samples (A – X).

Sample number specification	Spring Code	Protein concentration (µg/µl)	Peptide concentration (µg/µl)	Amount of protein used for digestion with trypsin (µg/µl)
A	R1	0.42458	1.451	~50 µg
B	R2	0.925299	0.551	~50 µg
C	R3	0.427037	1.973	~50 µg
D	R5	1.72706	0.496	~50 µg
E	R6	0.563432	1.697	~50 µg
F	R7	0.997794	0.553	~50 µg
G	R8	1.70433	0.755	~50 µg
H	R9	0.215078	1.908	~50 µg
I	H1	0.271602	1.381	~50 µg
J	H2	1.085652	1.636	~50 µg
K	H3	0.217535	0.951	~50 µg
L	H4	0.773546	1.155	~50 µg
M	H5	0.540086	0.946	~50 µg
N	H6	0.522882	1.551	~50 µg
O	H7	0.542543	1.671	~50 µg
P	H9	0.258084	1.583	~50 µg
Q	SW1	0.213849	1.123	~50 µg
R	SW2	0.301707	1.553	~50 µg
S	SW3	0.186818	1.38	~50 µg
T	SW4	0.11862	1.409	~50 µg
U	SW5	0.280818	1.283	~50 µg
V	SW6	0.287573	1.197	~50 µg
W	SW7	0.414752	2.027	~50 µg
X	SW8	0.288806	0.51	~50 µg

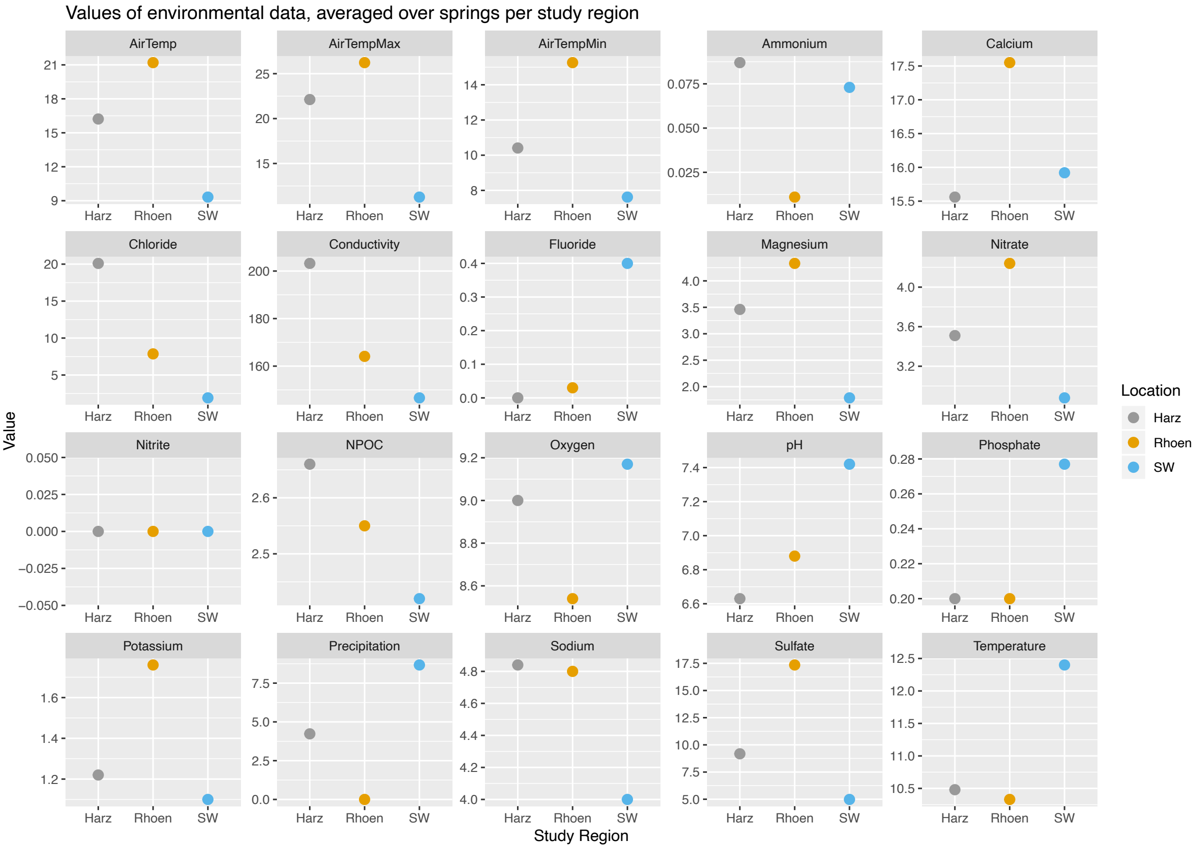

Supplementary Figure S1 | Dotplots of mean values of all abiotic measurements (*n* = 9 per region).

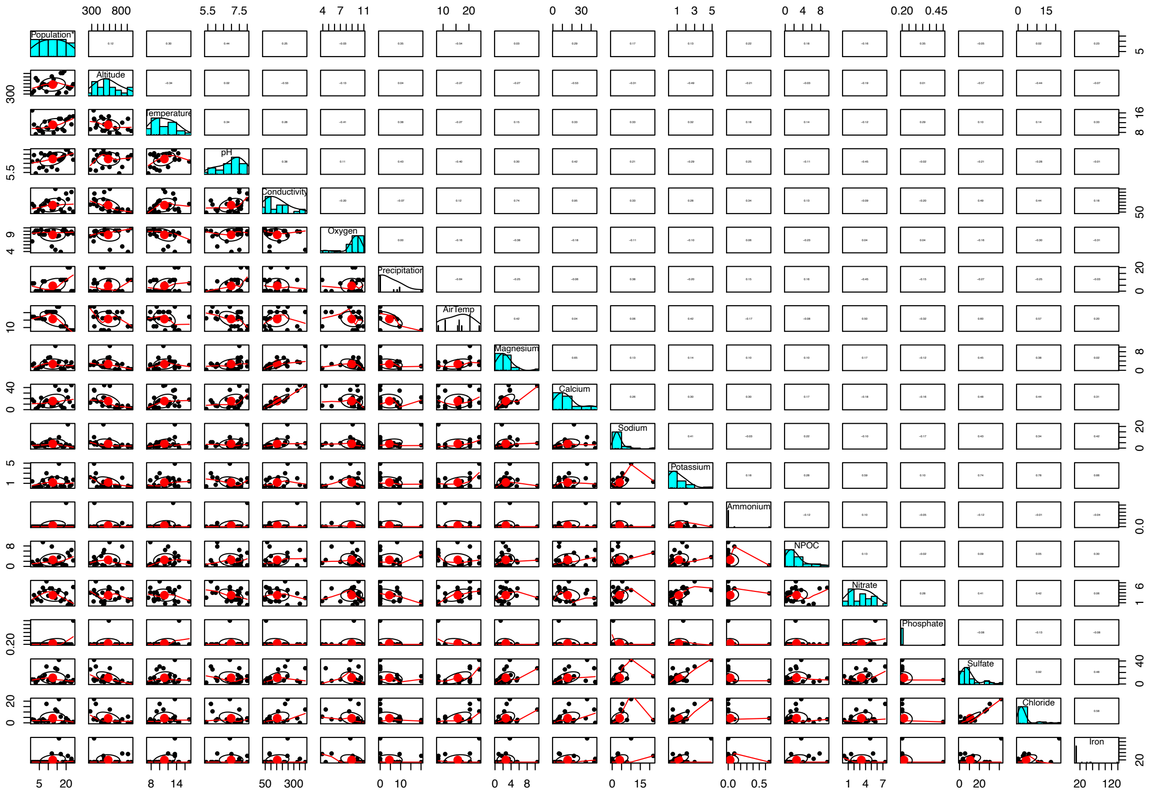

Supplementary Figure S2 | Cor.test output of pairwise comparisons of abiotic variables showing scatterplots and Pearson's correlation values. Of strongly

correlating (>0.7) variables, one was chosen to be included in analyses (for a reduced set of abiotic variables included in statistical analyses, see **Supplementary Information File 2**).

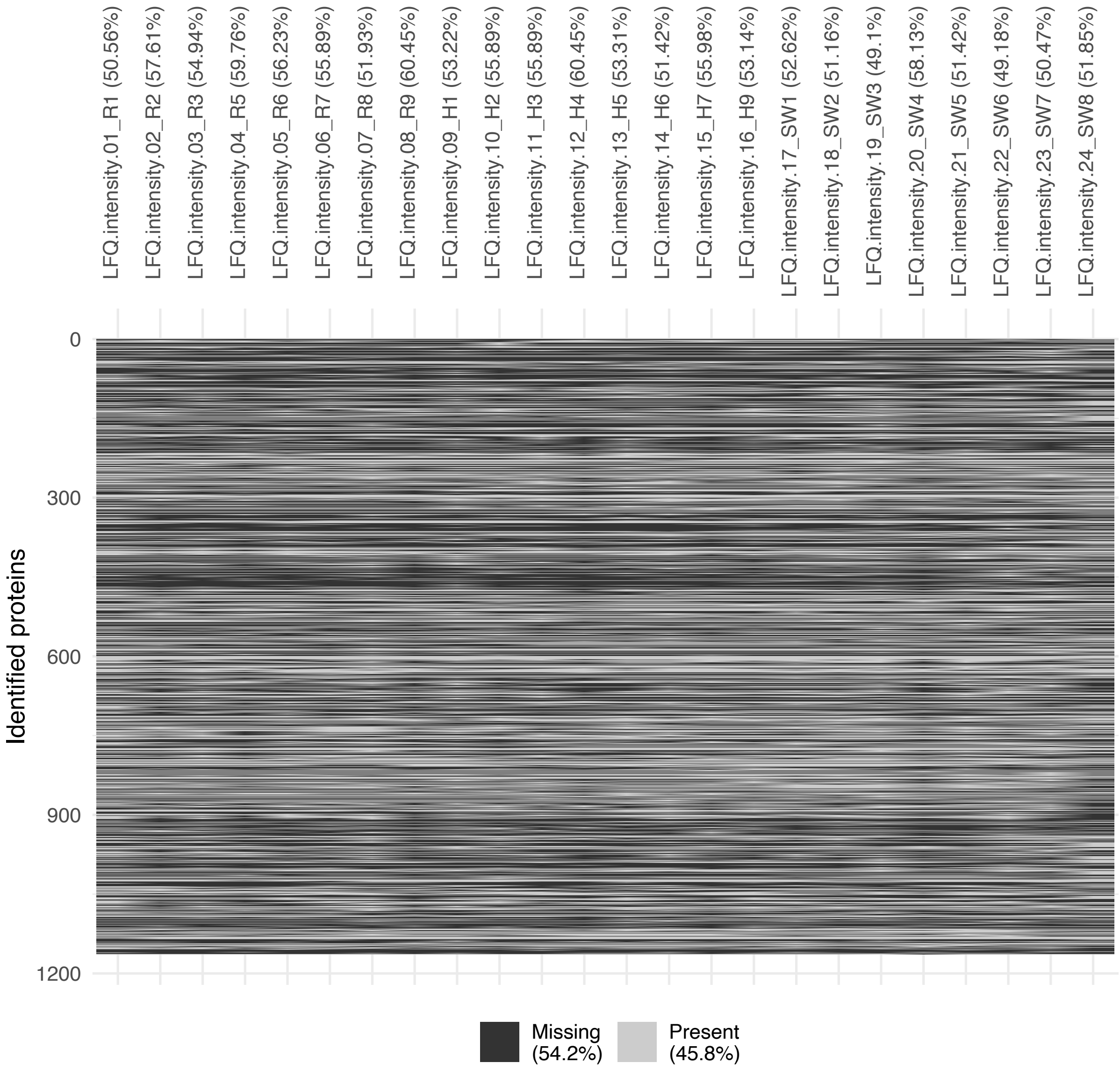

Supplementary Figure S3 | Number of missing values (NAs) of label-free quantification (LFQ) values in the MaxQuant output. Plot was generated using the vis_miss function of package visdat v.0.5.3 [1].

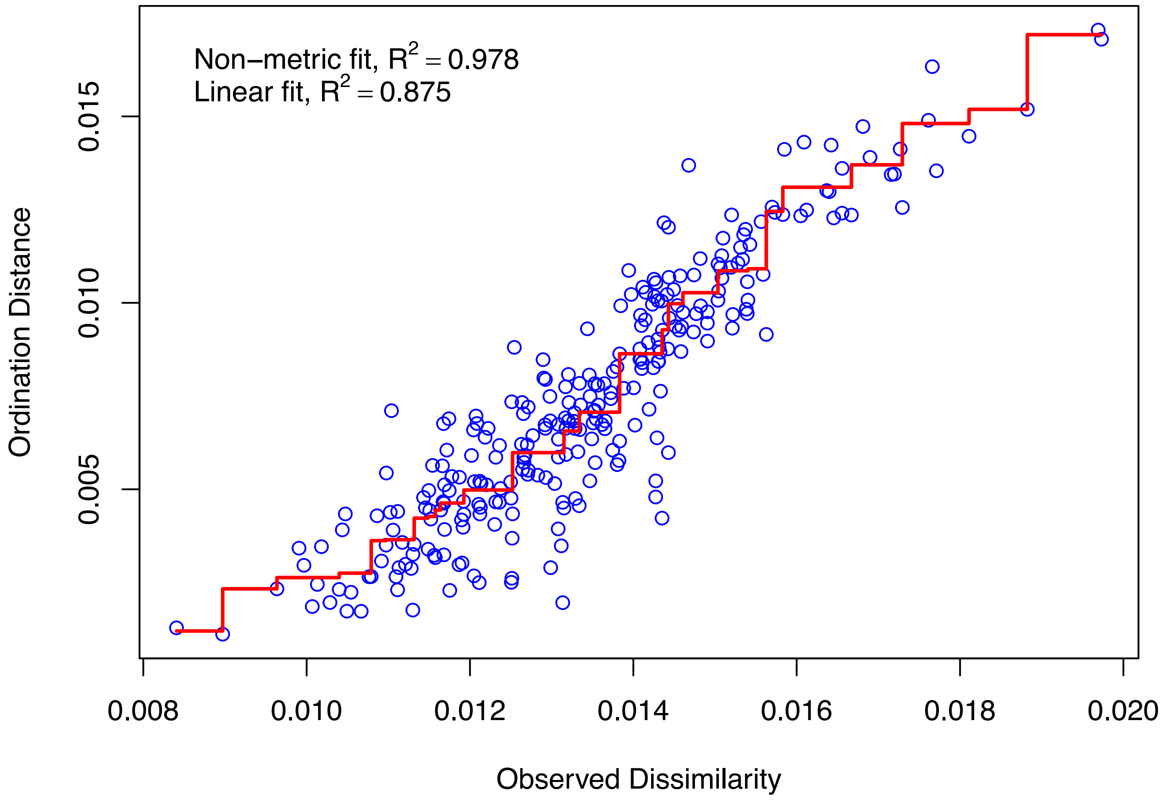

Supplementary Figure S4 | nMDS stress plot.

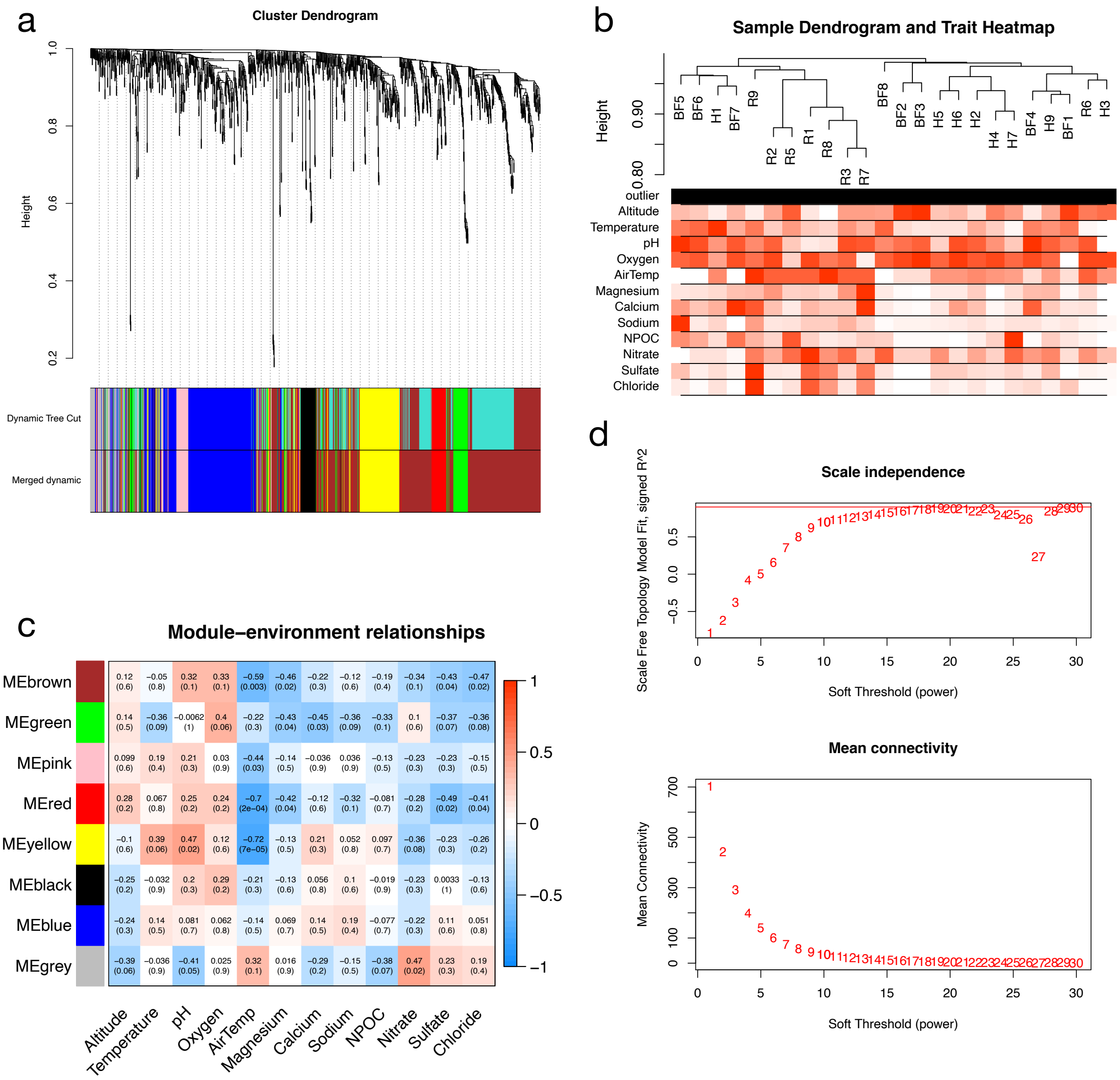

Supplementary Figure S5 | Statistics and results of the WGCNA analysis. (a) Clustering of proteins with dissimilarity based on topological overlap, together with assigned module colors (Dynamic Tree Cut; modules with more than *n* = 20 proteins). Merged dynamic below shows concatenated modules of Dynamic Tree Cut modules that showed a correlation of > 0.80, representing the modules used in the further analysis. **(b)** Sample dendogram and abiotic heatmap showing no outliers in the data. **(c)** Module-Environment relationships considering all 24 populations and 12 abiotic variables. **(d)** Analysis of global network topology for various soft-thresholding powers using protein abundance data of all 24 *C. irrorata* populations. Upper panel shows the scale-free fit index (y-axis) as a function of the soft-thresholding power (x-axis). The lower panel displays the mean connectivity (degree, y-axis) as a function of the soft-thresholding power (x-axis). We chose the power 18 for the global analysis, which is the lowest power for which the scale-free topology fit index reaches 0.90 (red cut-off line in left panel.

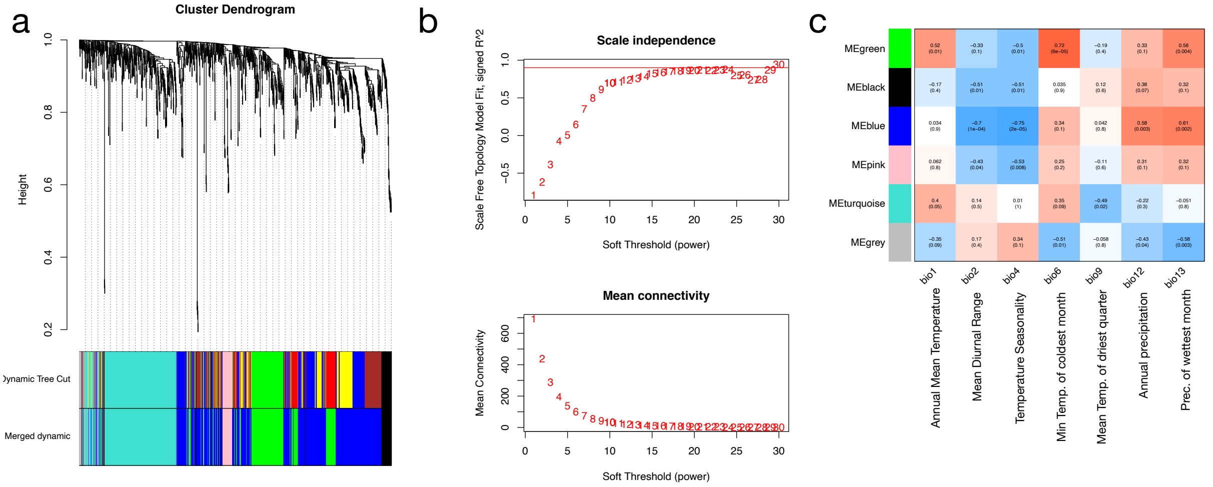

Supplementary Figure S6 | Statistics and results of the WGCNA$_{BC}$ analysis. (a) Clustering of proteins with dissimilarity based on topological overlap, together with assigned module colors (Dynamic Tree Cut; modules with more than *n* = 20 proteins). Merged dynamic below shows concatenated modules of Dynamic Tree Cut modules that showed a correlation of > 0.80, representing the modules used in the further analysis. **(b)** Analysis of global network topology for various soft-thresholding powers using protein abundance data of all 24 *C. irrorata* populations. Upper panel shows the scale-free fit index (y-axis) as a function of the soft-thresholding power (x-axis). The lower panel displays the mean connectivity (degree, y-axis) as a function of the soft-thresholding power (x-axis). We chose the power 18 for the BioClim analysis, which is the lowest power for which the scale-free topology fit index reaches 0.90 (red cut-off line in left panel; same as for the *in situ* abiotic variable WGCNA). (**c**) Module-BioClim relationships considering all 24 populations and 7 selected BioClim variables.

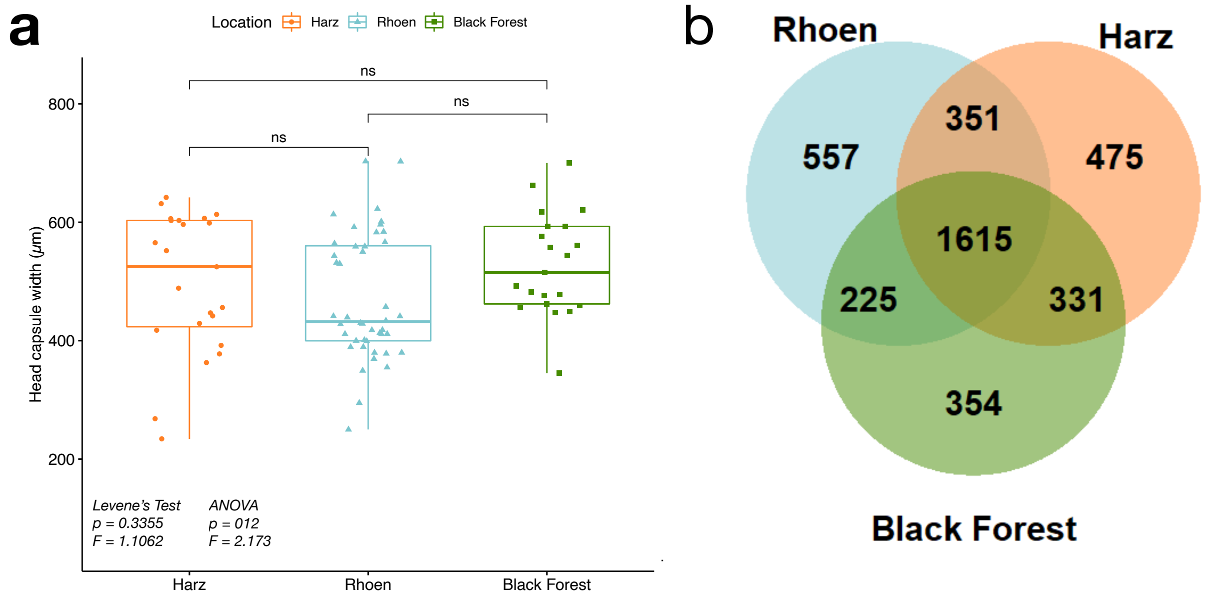

Supplementary Figure S7 | (**a**) Boxplots of head capsule widths of randomly sampled individuals (*n* = 88) and significance results of pairwise comparison

between sampling regions. (**b**) Venn diagram of number of identified majority protein IDs among sampling regions.

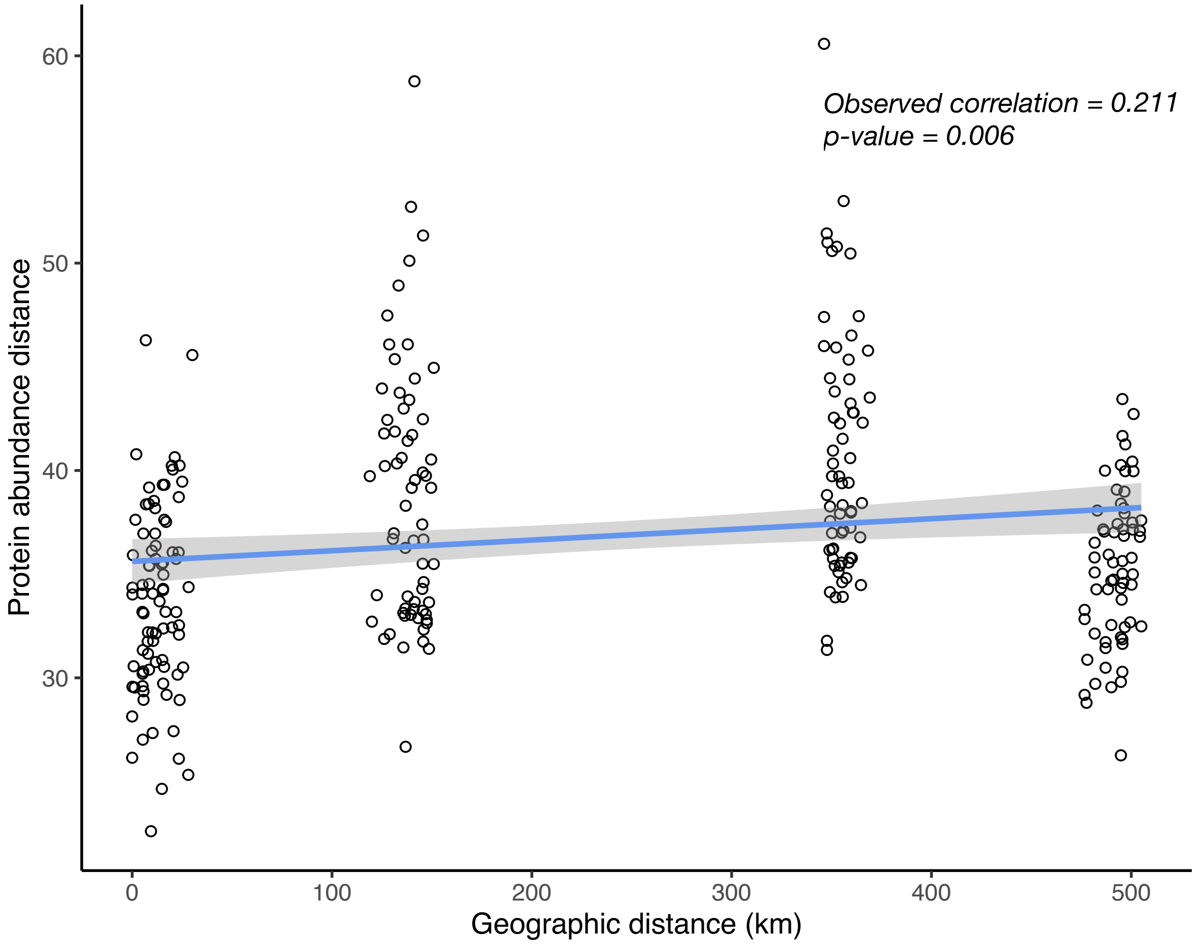

Supplementary Figure S8 | Scatterplot and linear regression of protein abundance distance (Euclidean) between springs (y-axis) and geographic distance (km) between springs. Mantel test statistics are given in the top right corner (observed correlation and *p*-value).

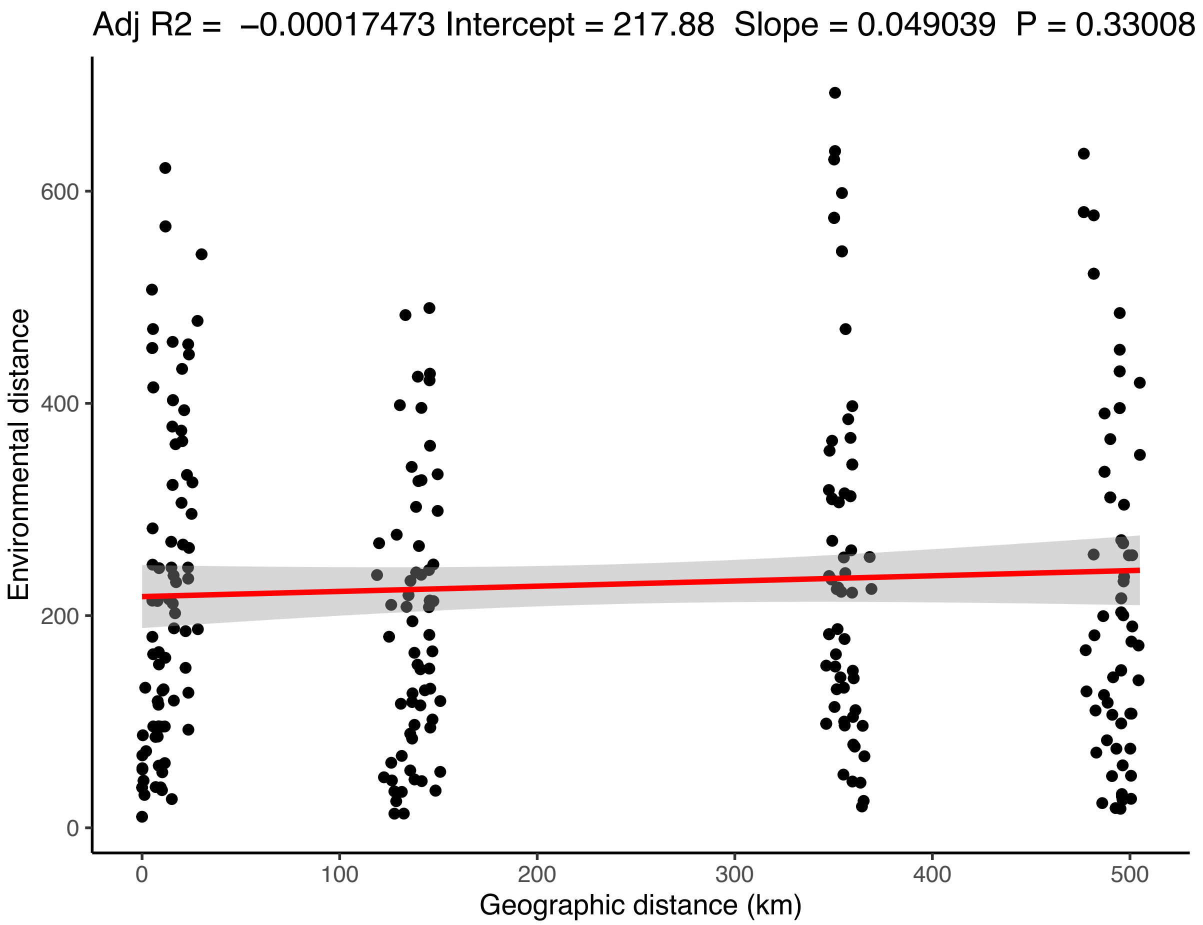

Supplementary Figure S9 | Scatterplot and linear regression of environmental distance (Euclidean) between springs (y-axis; based on 11 abiotic variables) and geographic distance (km) between springs. Adjusted R^2, intercept, slope and significance of the linear model are given on top of the scatterplot.

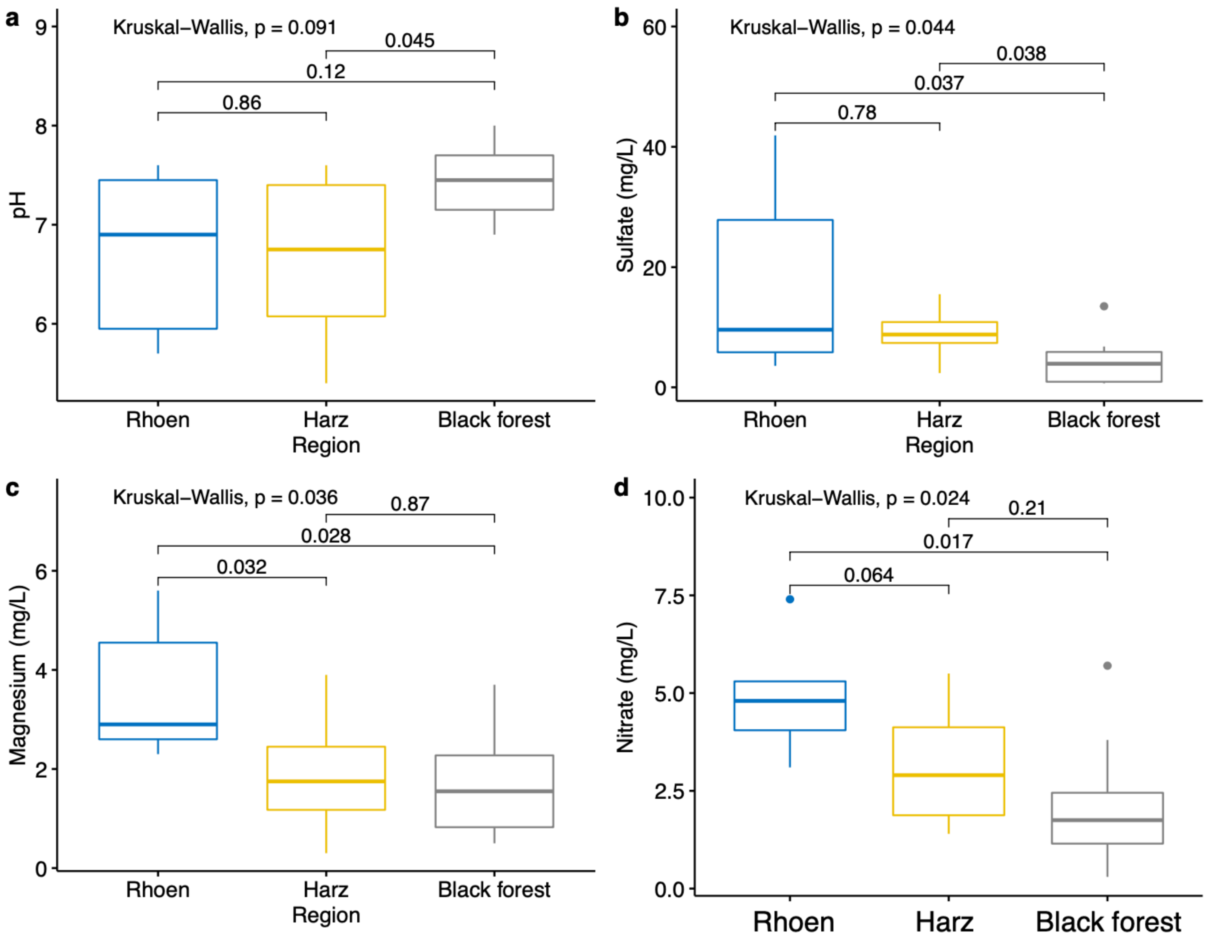

Supplementary Figure S10 | Boxplots and significance results of Kruskal-Wallis pairwise comparisons of 4 abiotic variables between sampling regions. (**a**) pH. (**b**) Sulfate. (**c**) Magnesium. (**d**) Nitrate.

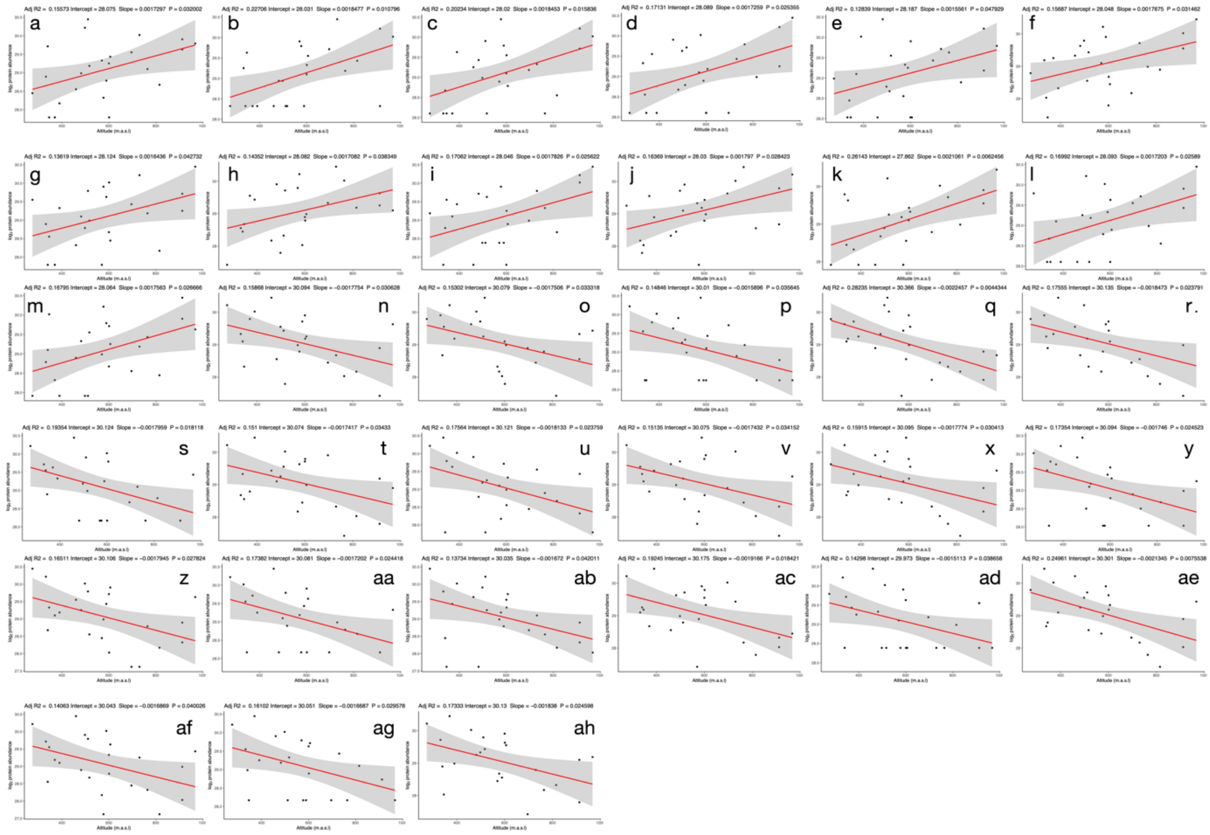

Supplementary Figure S11 | Reaction norms of 33 proteins in relation to elevation (m.a.s.l) of springs. Of these, 13 show significantly increased abundances with increasing altitude (**a-m**) and 20 proteins significantly decreasing abundances with increasing altitude (**n-ah**).

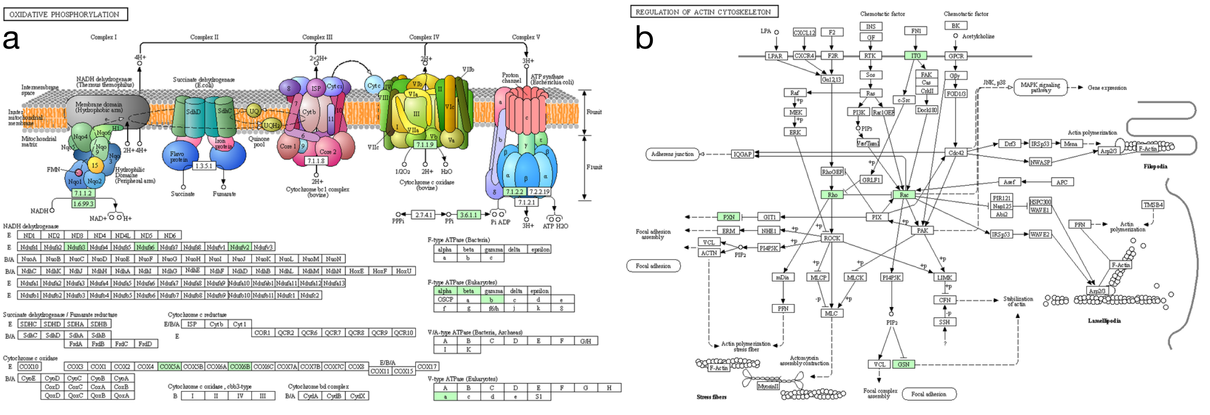

Supplementary Figure S12 | Pathways related to changes in aqueous pH. Mapping of member proteins of the "yellow" module (n_{yellow} = 110; 82.71 % annotated) to (**a**) oxidative phosphorylation (*n* = 15 proteins (13,65 %)) and (**b**) regulation of actin cytoskeleton (*n* = 9 proteins (8,19 %)) pathways. Green boxes represent KEGG nodes present in the module. Pathways were visualized using KEGG mapper (http:///www.genome.jp/ keg/mapper)[2–45].

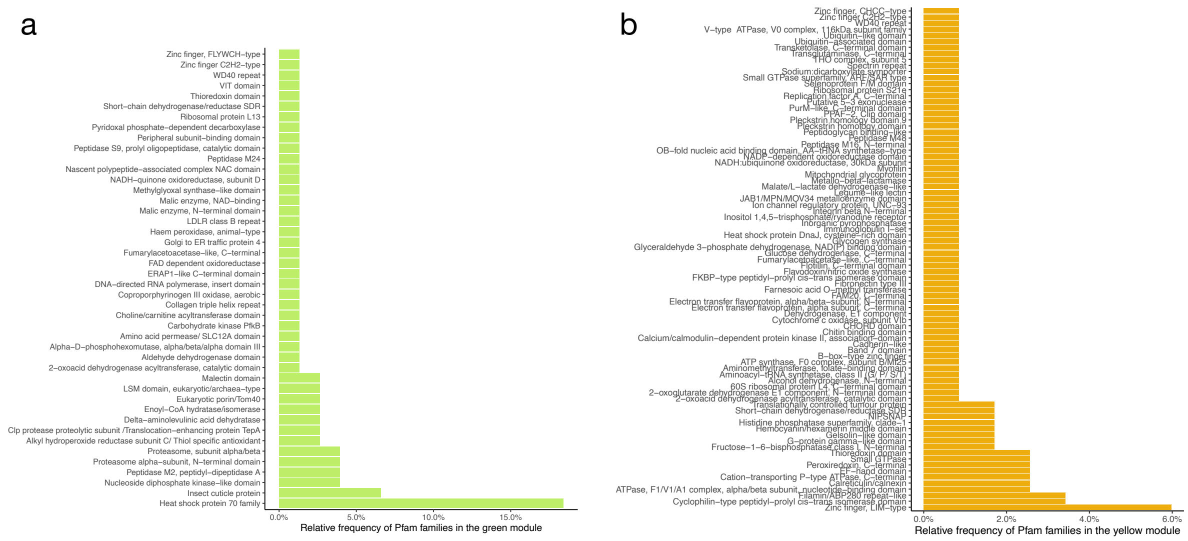

Supplementary Figure S13 | Pfam family frequency in modules (**a**) "green" and (**b**) "yellow".

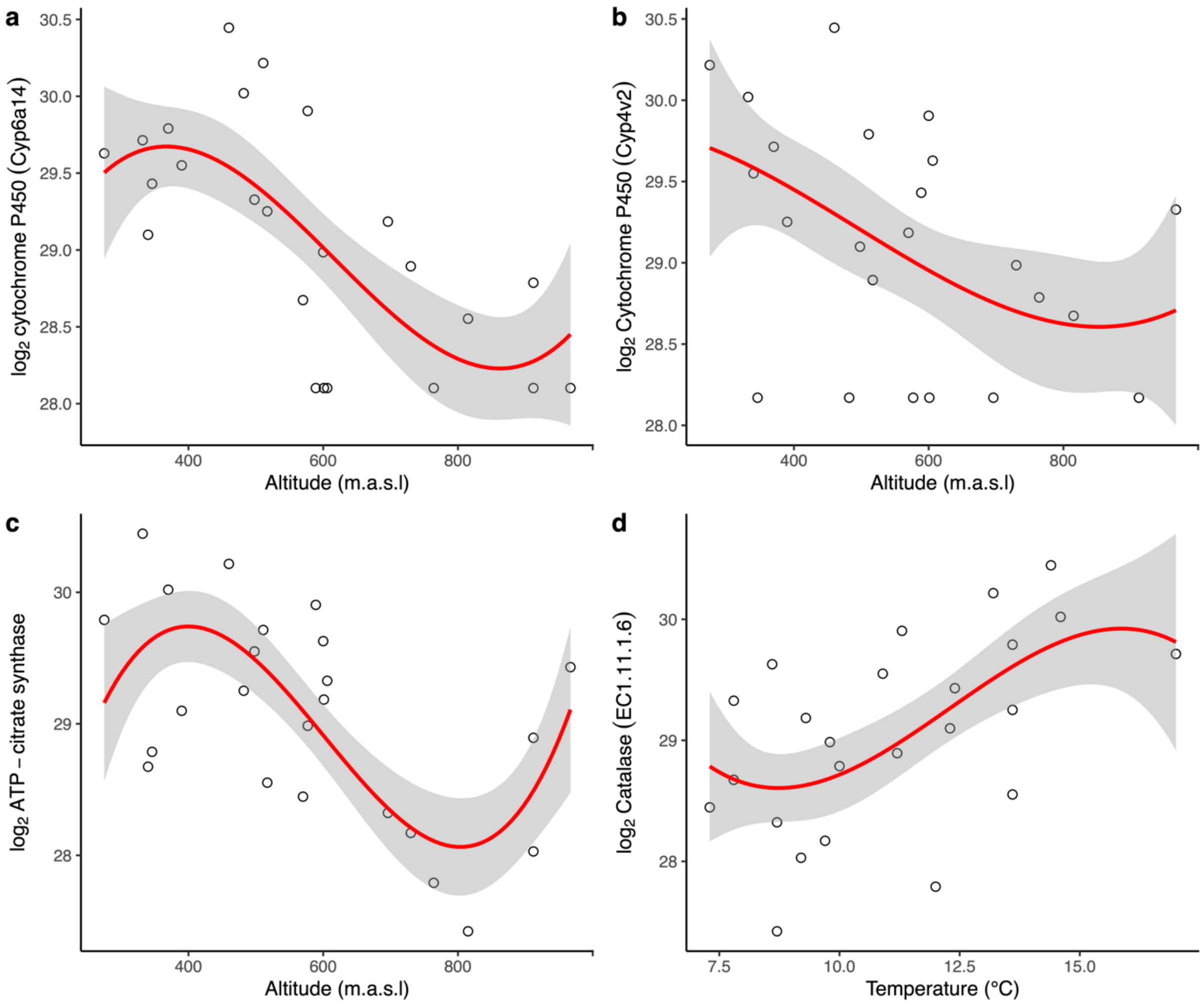

Supplementary Figure S14 | Exemplary reaction norms (polynomial regression) of population-wide protein biomarker abundances (y-axis) in relation to spring altitude and temperature (x-axis). (**a**) Probable cytochrome P450 (*Cyp6a2*) (**b**) Cytochrome P450 4C1 (Cyp4c1). (**c**) ATP-citrate synthase. (**d**) Catalase. Shown are

the fit line (red line) and 95% confidence interval (light grey area). RMSE: Root Mean Square Error; R^2: correlation coefficient; *p*: level of statistical significance of the model.

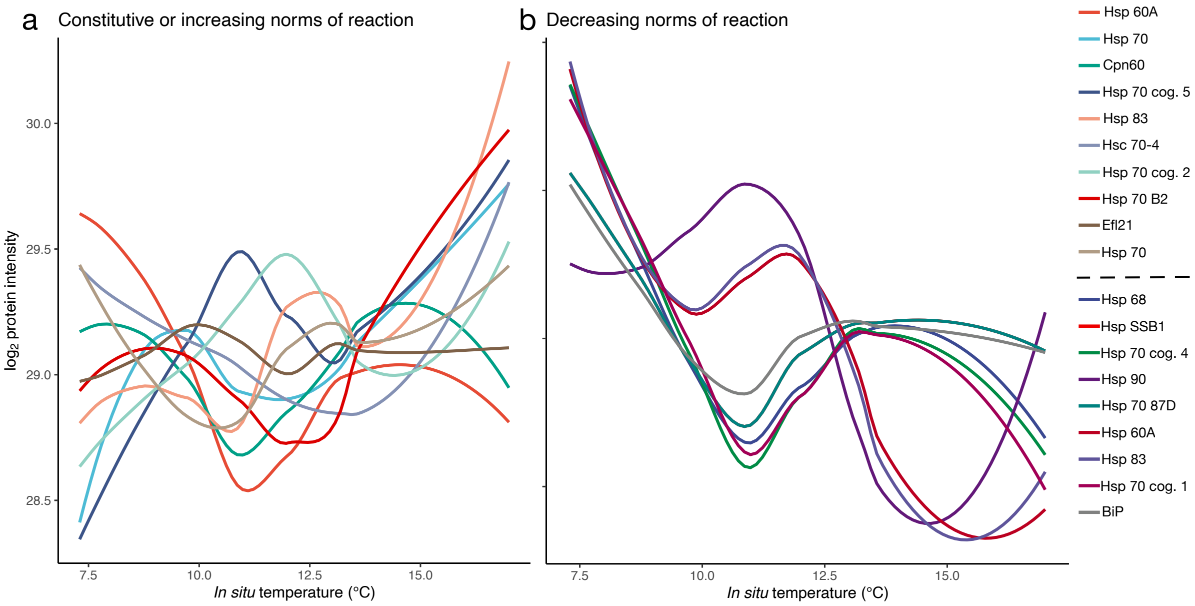

Supplementary Figure S15 | Heat-shock protein reaction norms. (a) Constitutive or increasing norms of reaction with increasing spring *in situ* temperatures. **(b)** Decreasing norms of reaction with increasing spring *in situ* temperatures.

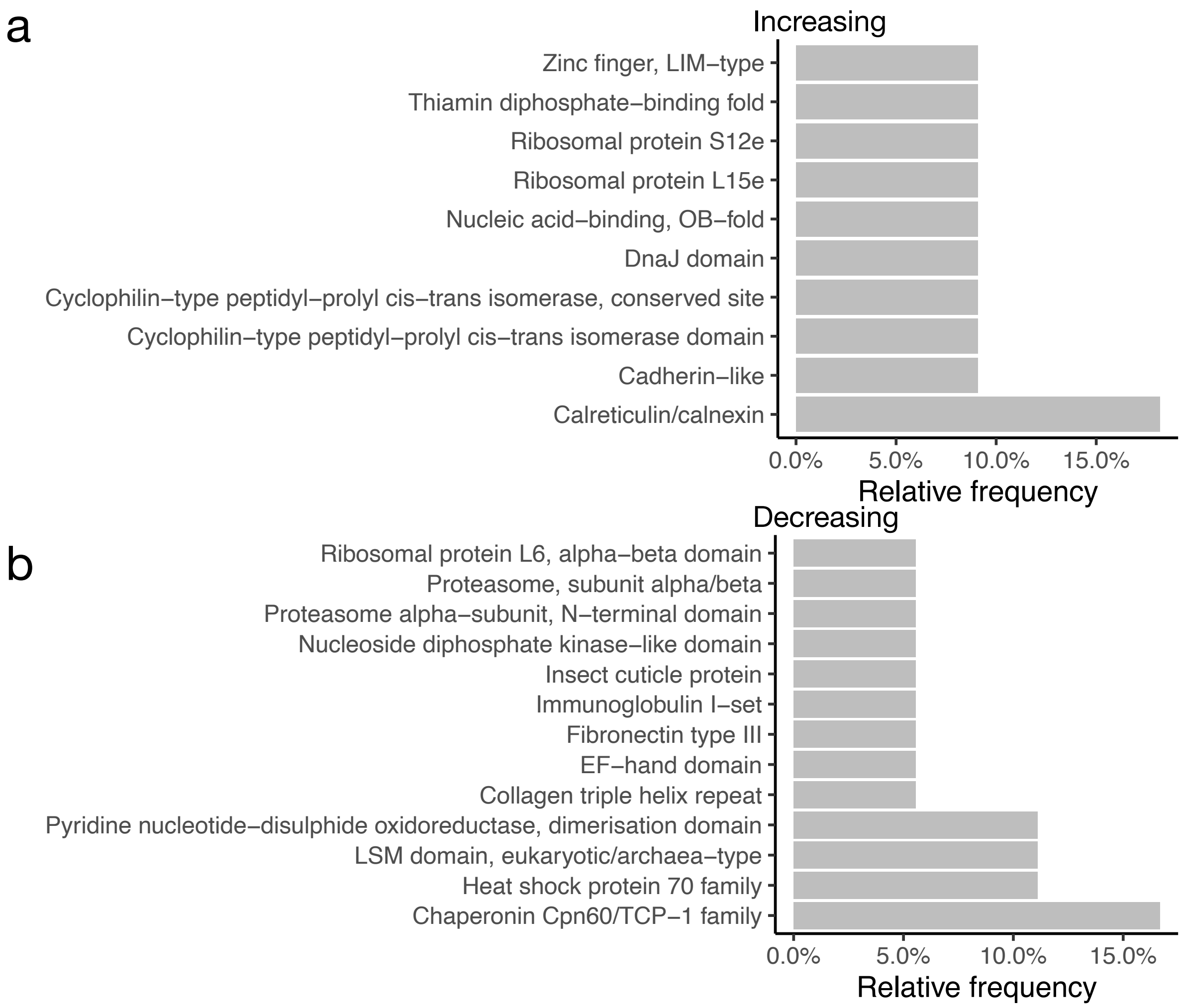

Supplementary Figure S16 | Pfam family frequencies in reaction norm proteins. (**a**) increasing in abundance with increasing in situ temperature (°C) of springs (n = 29) and (**b**) decreasing in abundance with increasing in situ temperature (°C) of springs (n = 33).

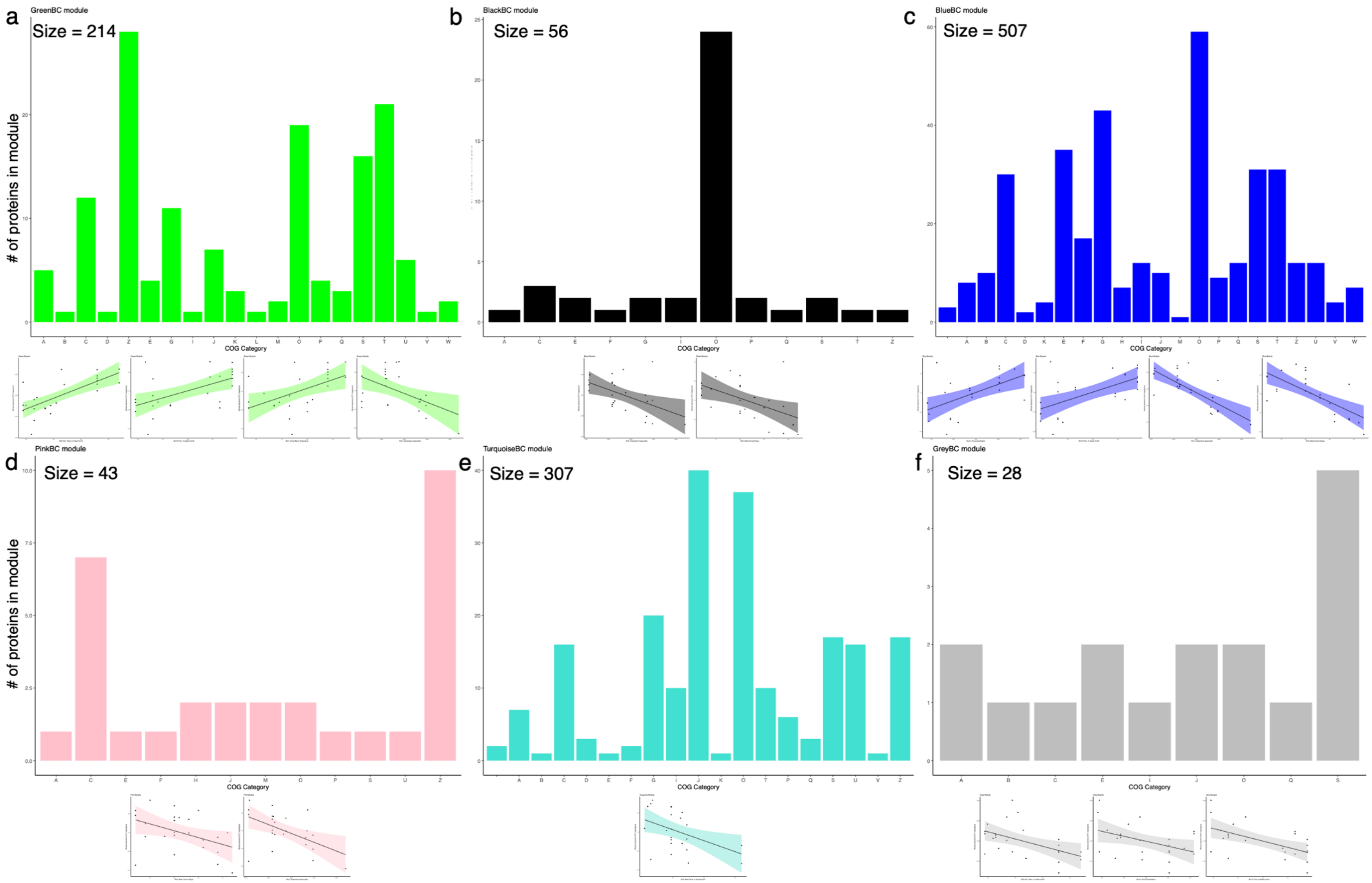
a
GreenBC module
Size = 214
# of proteins in module
COG Category
b
BlackBC module
Size = 56
COG Category
c
BlueBC module
Size = 507
COG Category
d
PinkBC module
Size = 43
# of proteins in module
COG Category
e
TurquoiseBC module
Size = 307
COG Category
f
GreyBC module
Size = 28
COG Category

Supplementary Figure S17 | Overview of WGCNA$_{BC}$ modules. (**a**) COG family distribution of proteins belonging to the "green$_{BC}$" module. One-letter abbreviations are identical to Fig. 2 in the main manuscript. Scatterplots underneath portray the "green$_{BC}$" module eigengene expression positively associated with BioClim Bio6 (min. temp. of coldest month), Bio13 (prec. of wettest month), Bio1 (annual mean temperature) and Bio4 (temperature seasonality) variables. (**b**) COG family distribution of proteins belonging to the "black$_{BC}$" module. One-letter abbreviations are identical to Fig. 2 in the main manuscript. Scatterplots underneath portray the "black$_{BC}$" module eigengene expression positively associated with BioClim Bio4 (temperature seasonality) and Bio2 (mean diurnal range) variables. (**c**) COG family distribution of proteins belonging to the "blue$_{BC}$" module. One-letter abbreviations are identical to Fig. 2 in the main manuscript. Scatterplots underneath portray the "blue$_{BC}$" module eigengene expression positively associated with BioClim Bio12 (annual precipitation), Bio13 (prec. of wettest month), Bio4 (temperature seasonality) and Bio2 (mean diurnal range) variables. (**d**) COG family distribution of proteins belonging to the "pink$_{BC}$" module. One-letter abbreviations are identical to Fig. 2 in the main manuscript. Scatterplots underneath portray the "pink$_{BC}$" module eigengene expression positively associated with BioClim Bio2 (mean diurnal range) and Bio4 (temperature seasonality) variables. (**e**) COG family distribution of proteins belonging to the "turquoise$_{BC}$" module. One-letter abbreviations are identical to Fig. 2 in the main manuscript. Scatterplots underneath portray the "turquoise$_{BC}$" module eigengene expression positively associated with BioClim Bio9 (mean temp. of driest quarter) variable. (**f**) COG family distribution of proteins belonging to the "grey$_{BC}$" module. One-letter abbreviations are identical to Fig. 2 in the main manuscript. Scatterplots underneath portray the "grey$_{BC}$" module eigengene expression positively associated with BioClim Bio6 (min. temp. of coldest month) and Bio12 (annual precipitation) variables.

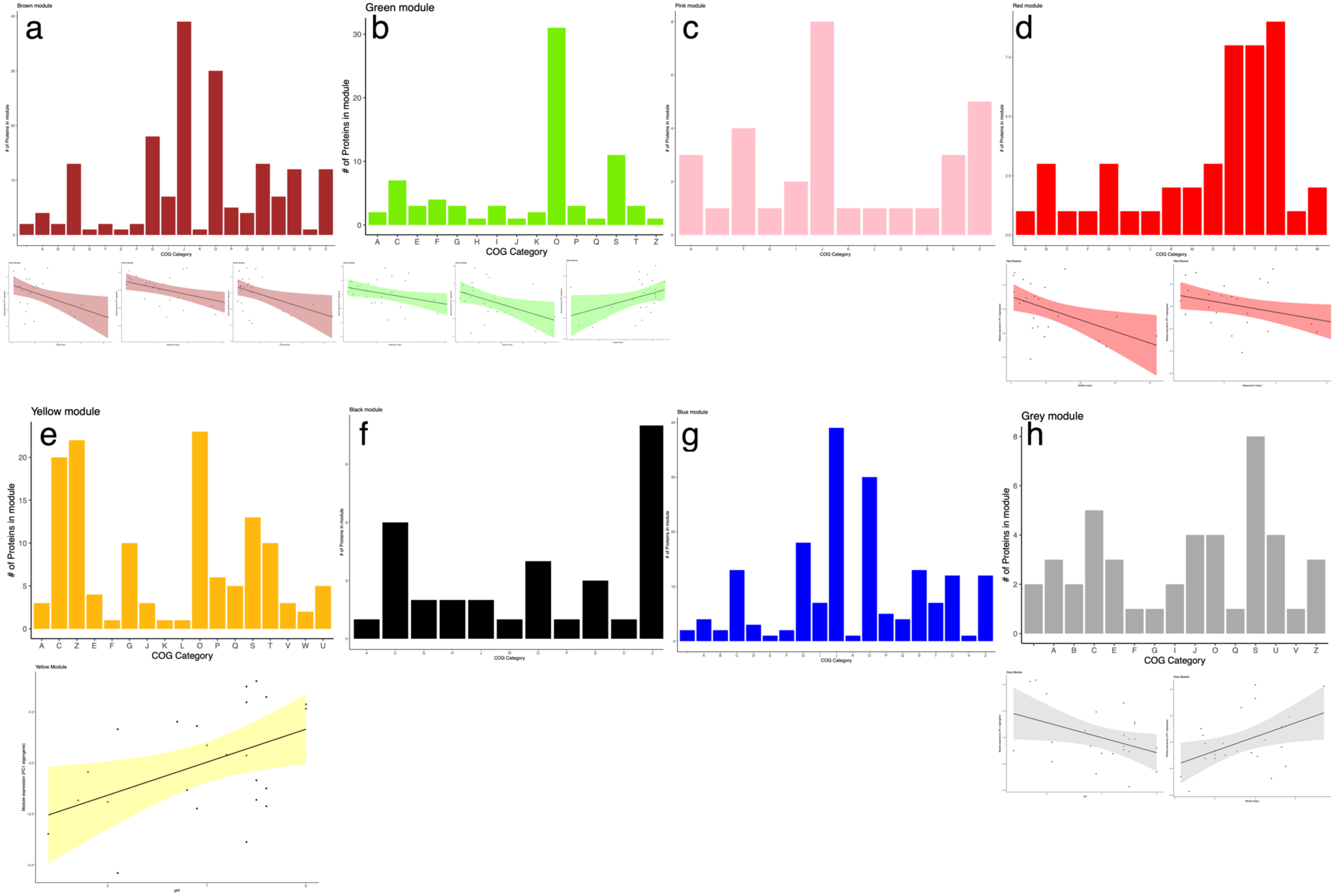
Brown module
a
# of Proteins in module
COG Category
Green module
b
# of Proteins in module
0
10
20
30
A C E F G H I J K O P Q S T Z
COG Category
Pink module
c
# of Proteins in module
COG Category
Red module
d
# of Proteins in module
COG Category
Red Module
Yellow module
e
# of Proteins in module
0
5
10
15
20
A C Z E F G J K L O P Q S T V W U
COG Category
Yellow Module
Module expression (PC1 eigengene)
pH
Black module
f
# of Proteins in module
COG Category
Blue module
g
# of Proteins in module
COG Category
Grey module
h
# of Proteins in module
0
2
4
6
8
A B C E F G I J O Q S U V Z
COG Category
Grey Module

Supplementary Figure S18 | Overview of WGCNA modules. (**a**) COG family distribution of proteins belonging to the “brown” module. One-letter abbreviations are identical to Fig. 2 in the main manuscript. Scatterplots underneath portray the “brown” module eigengene expression negatively associated with sulfate, magnesium and chloride abiotic variables. (**b**) COG family distribution of proteins belonging to the “green” module. One-letter abbreviations are identical to Fig. 2 in the main manuscript. Scatterplots underneath portray the “green” module eigengene expression negatively associated with magnesium and calcium and positively associated with oxygen. (**c**) COG family distribution of proteins belonging to the “pink” module. The eigengene expression of the “pink” module was not associated with any measured abiotic variables. (**d**) COG family distribution of proteins belonging to the “red” module. One-letter abbreviations are identical to Fig. 2 in the main manuscript. Scatterplots underneath portray the “red” module eigengene expression negatively associated with sulfate and magnesium abiotic variables. (**e**) COG family distribution of proteins belonging to the “yellow” module. One-letter abbreviations are identical to Fig. 2 in the main manuscript. Scatterplots underneath portray the “yellow” module eigengene expression positively associated with the pH abiotic variable. (**f**) COG family distribution of proteins belonging to the “black” module. One-letter abbreviations are identical to Fig. 2 in the main manuscript. The eigengene expression of the “black” module was not associated with any measured abiotic variables. (**g**) COG family distribution of proteins belonging to the “blue” module. The eigengene expression of the “blue” module was not associated with any measured abiotic variables. (**h**) COG family distribution of proteins belonging to the “grey” module. Scatterplots underneath portray the “grey” module eigengene expression negatively associated with pH and positively associated with nitrate abiotic variables.

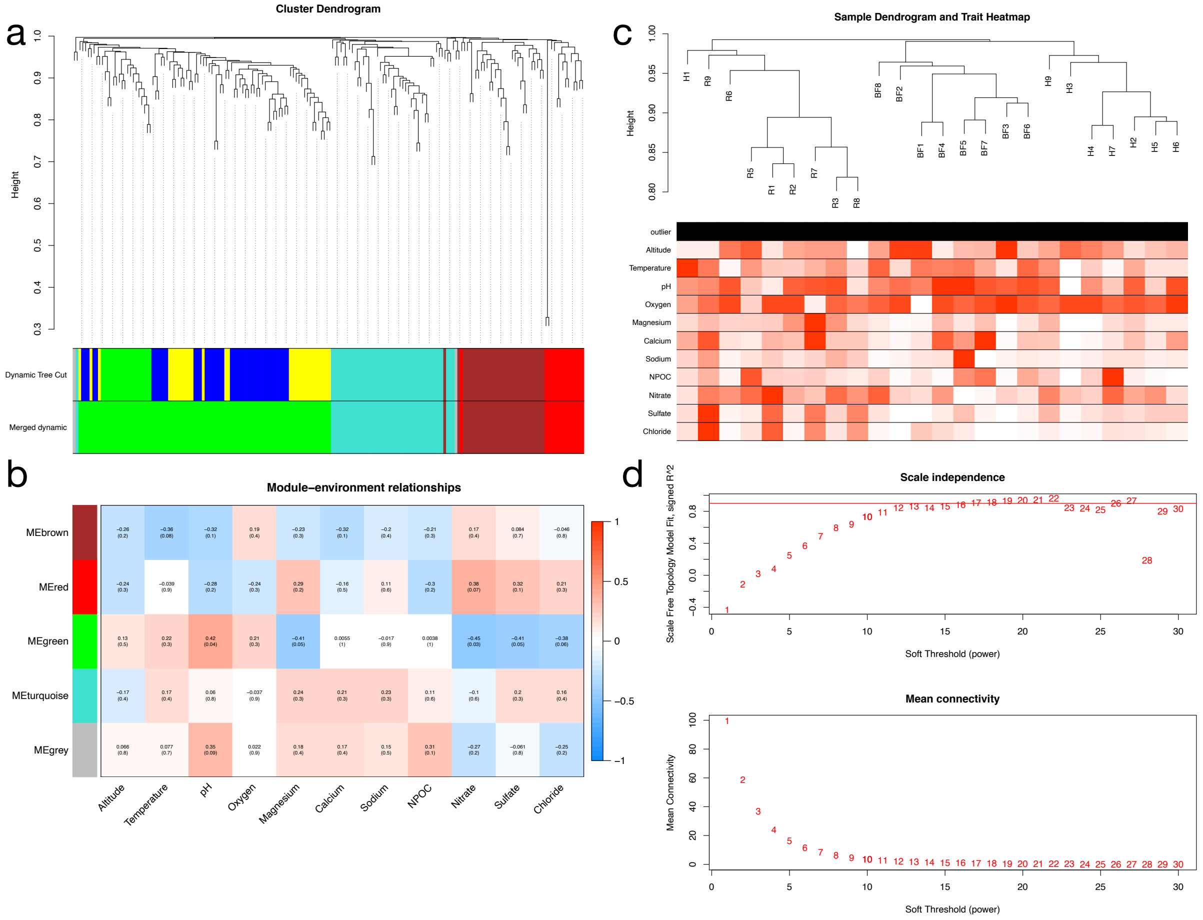

Supplementary Figure S19 | Statistics and results of the WGCNA$_{DAP}$ analysis. (a) Clustering of proteins with dissimilarity based on topological overlap, together with assigned module colors (Dynamic Tree Cut; modules with more than *n* = 10 proteins). Merged dynamic below shows concatenated modules of Dynamic Tree Cut modules that showed a correlation of > 0.50, representing the modules used in the further analysis. (**b**) Module-Environment relationships considering all 24 populations and 11 abiotic variables. **(c)** Sample dendogram and abiotic heatmap showing no outliers in the data. **(d)** Analysis of global network topology for various soft-thresholding powers using protein abundance data of all 24 *C. irrorata* populations. Upper panel shows the scale-free fit index (y-axis) as a function of the soft-thresholding power (x-axis). The lower panel displays the mean connectivity (degree, y-axis) as a function of the soft-thresholding power (x-axis). We chose the power 12 for the DAP analysis, which is the lowest power for which the scale-free topology fit index reaches 0.90.

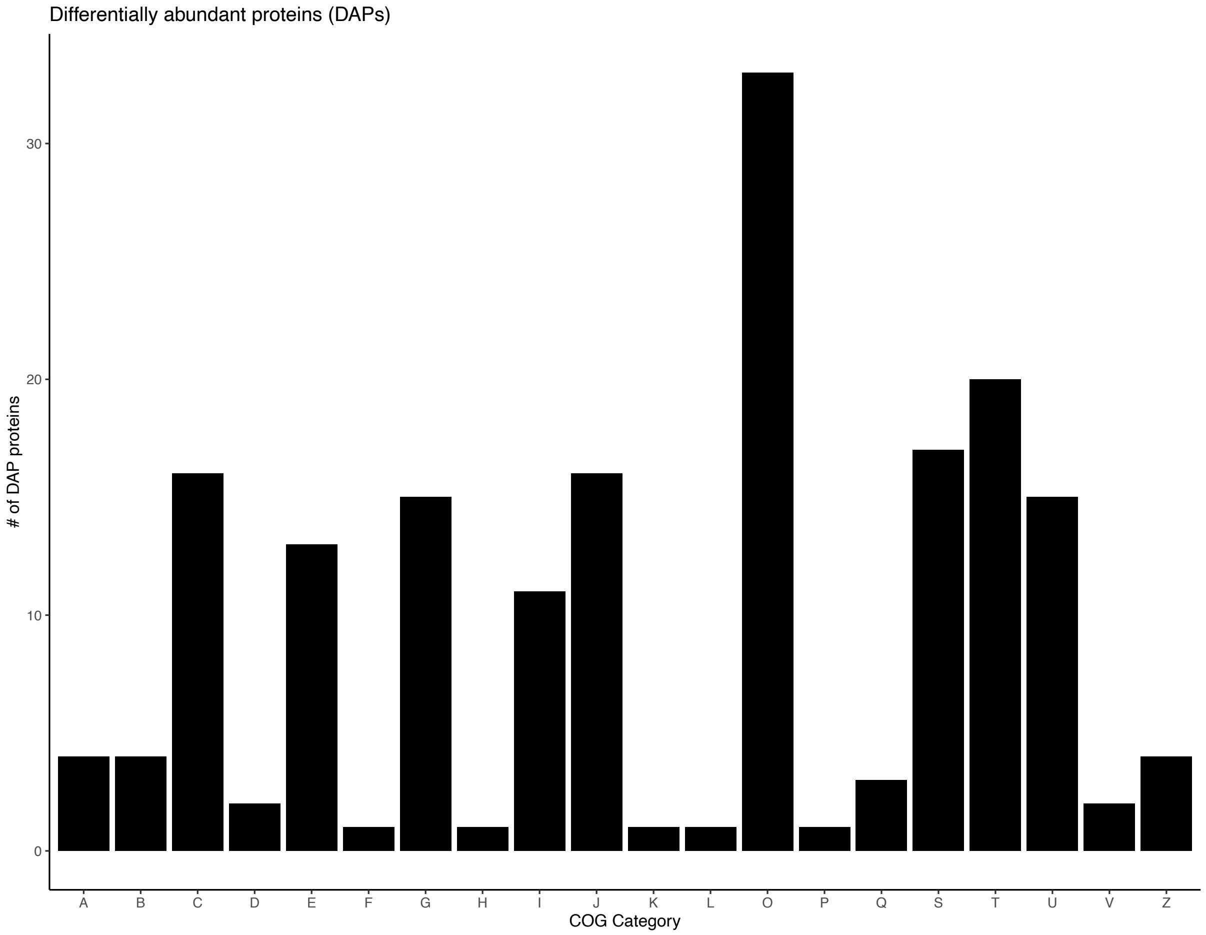

Supplementary Figure S20 | COG family distribution of differentially abundant proteins (DAPs) between sampling regions identified via LIMMA. One-letter abbreviations are identical to Fig. 2 in the main manuscript.

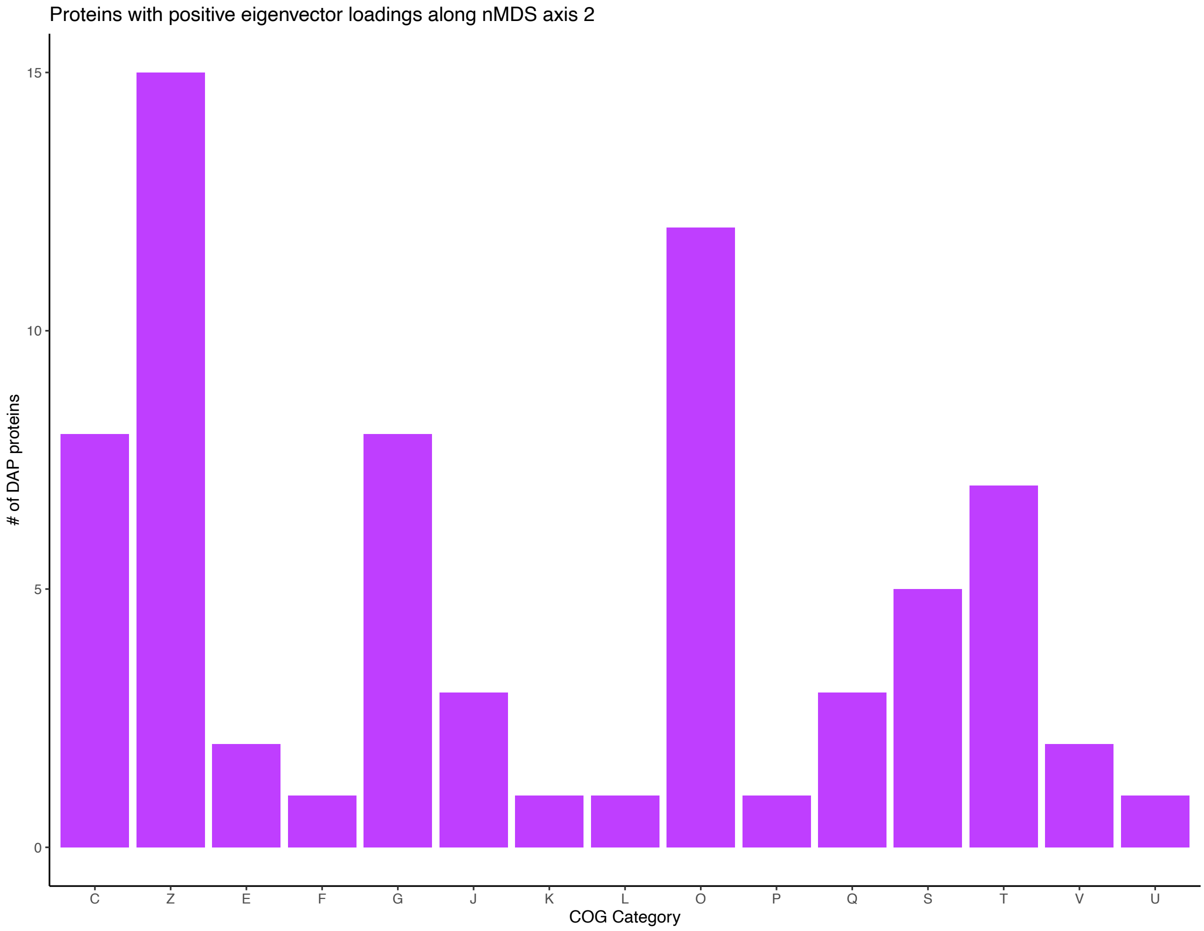

Supplementary Figure S21 | COG family distribution of proteins with positive eigenvector loadings in nMDS axis 2, separating sampling regions according tot he nMDS solution. One-letter abbreviations are identical to Fig. 2 in the main manuscript.

References

1. Tierney, N. Preliminary Visualisation of Data [R package visdat version 0.5.3]. *2019* (2019).
2. Kanehisa, M. & Goto, S. KEGG: kyoto encyclopedia of genes and genomes. *Nucleic Acids Res.* **28**, 27–30 (2000).
3. Kanehisa, M., Sato, Y., Furumichi, M., Morishima, K. & Tanabe, M. New approach for understanding genome variations in KEGG. *Nucleic Acids Res.* **47**, D590–D595 (2019).
4. Kanehisa, M. Toward understanding the origin and evolution of cellular organisms. *Protein Sci. Publ. Protein Soc.* **28**, 1947–1951 (2019).
5. Kanehisa, M. & Sato, Y. KEGG Mapper for inferring cellular functions from protein sequences. *Protein Sci. Publ. Protein Soc.* **29**, 28–35 (2020).
